# Hydrogen Transfer-Mediated Multicomponent Reaction for Direct Synthesis of Quinazolines by a Naphthyridine-Based Iridium Catalyst

**DOI:** 10.1016/j.isci.2020.101003

**Published:** 2020-03-21

**Authors:** Zhenda Tan, Zhongxin Fu, Jian Yang, Yang Wu, Liang Cao, Huanfeng Jiang, Juan Li, Min Zhang

**Affiliations:** 1 Key Lab of Functional Molecular Engineering of Guangdong Province, School of Chemistry and Chemical Engineering, South China University of Technology, Guangzhou, Guangdong, China; 2 Department of Chemistry, Jinan University, Huangpu Road West 601, Guangzhou, Guangdong 510632, P. R. China

**Keywords:** Inorganic Chemistry, Molecular Inorganic Chemistry, Catalysis

## Abstract

Selective linkage of renewable alcohols and ammonia into functional products would not only eliminate the prepreparation steps to generate active amino agents but also help in the conservation of our finite fossil carbon resources and contribute to the reduction of CO_2_ emission. Herein the development of a novel 2-(4-methoxyphenyl)-1,8-naphthyridine-based iridium (III) complex is reported, which exhibits excellent catalytic performance toward a new hydrogen transfer-mediated annulation reaction of 2-nitrobenzylic alcohols with alcohols and ammonia. The catalytic transformation proceeds with the striking features of good substrate and functional group compatibility, high step and atom efficiency, no need for additional reductants, and liberation of H_2_O as the sole by-product, which endows a new platform for direct access to valuable quinazolines. Mechanistic investigations suggest that the non-coordinated N-atom in the ligand serves as a side arm to significantly promote the condensation process by hydrogen bonding.

## Highlights

• Use of abundant ammonia and alcohols
• Good substrate and functional group compatibility
• New naphthyridine-based Ir catalyst
• Strategy merging hydrogen transfer and annulation

## Graphical Abstract

**Figure:**
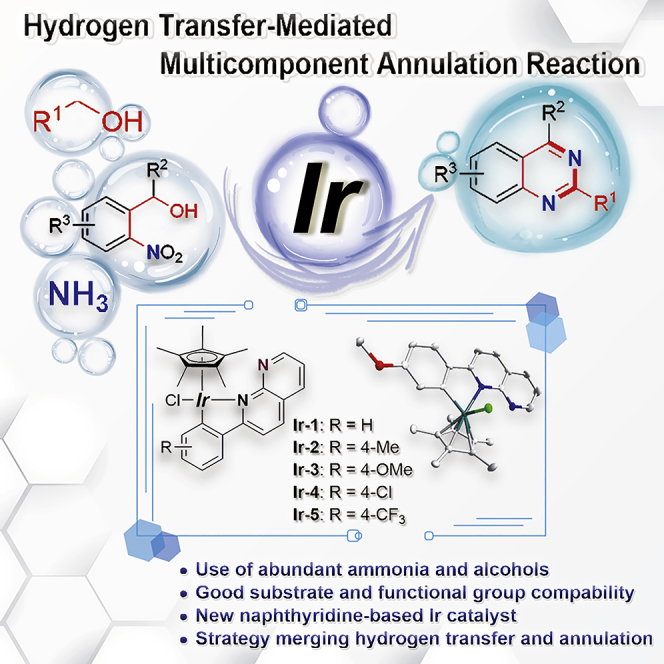

## Introduction

Mass mining and consumption of fossil resources have resulted in a call for the development of new catalytic transformations, enabling production of functional chemicals from renewable resources with high step and atom efficiency (Goldemberg, 2007, Michlik. and Kempe, 2013a, Michlik and Kempe, 2013b, Kozlowski and Davis, 2013). Among various alternative feedstocks, alcohols are a category of oxidized hydrocarbons that can be extensively derived from biomass including abundantly available lignocellulose via degradation (Zakzeski et al., 2010, Sun et al., 2018, Vispute et al., 2010). N-heteroarenes represent a class of highly important compounds, and they have been extensively employed for the development of valuable products, such as bioactive molecules, pharmaceuticals, agrochemicals, dyes, ligands, sensors, and materials (Boyarskiy et al., 2016, Preshlock et al., 2016, Bandini, 2011). Consequently, the linkage of alcohols into N-heteroaromatic frameworks is of high importance, as it not only helps in the conservation of our finite fossil carbon resources but also contributes to the reduction of CO_2_ emission.

**Scheme 1:**
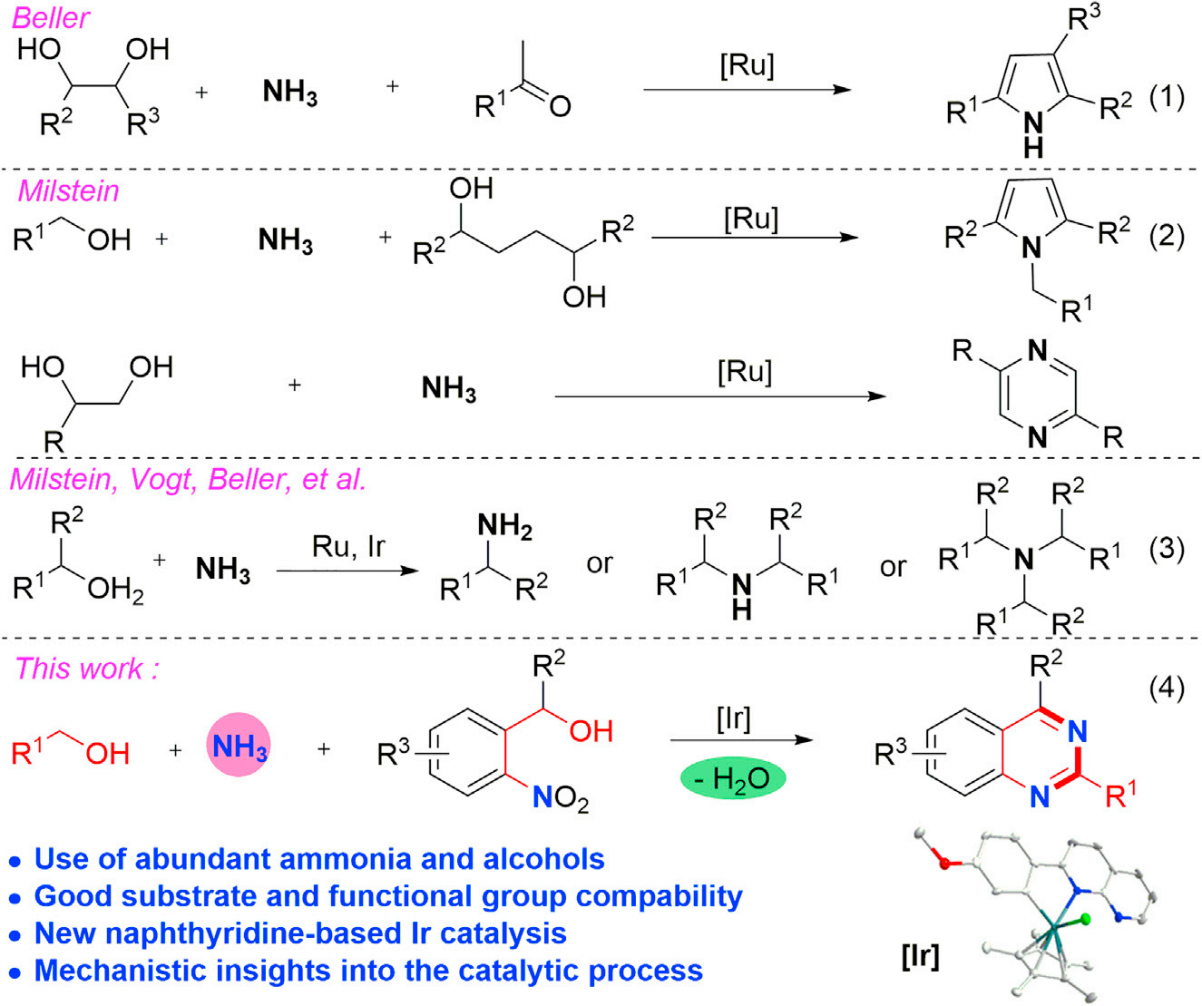
Alcohols and Ammonia Utilized for the Synthesis of N-Heteroarene and Amine

Over the past decade, the strategy of acceptorless dehydrogenative coupling (ADC) proceeded to renew the construction of N-heteroarenes. In this strategy, dehydrogenation is involved in the activation of alcohols via *in situ* formation of carbonyl intermediates, and H_2_ and/or H_2_O are generally generated as the by-products. Since 2013, significant progress has been made in this regard by the groups of Milstein (Srimani et al., 2013a, Srimani et al., 2013b, Daw et al., 2016, Daw et al., 2017), Kempe (Michlik. and Kempe, 2013a, Michlik and Kempe, 2013b, Deibl et al., 2015, Hille et al., 2014, Hille et al., 2017, Deibl and Kempe, 2017, Kallmeier et al., 2017), Beller (Zhang et al., 2013a, Zhang et al., 2013b), Kirchner (Mastalir et al., 2016), and others (Pan et al., 2016, Xu et al., 2017, Elangovan et al., 2015, Chen et al., 2014). However, it is important to note that these transformations mainly rely on the utilization of specific amines, whereas the synthesis of N-heteroarenes by combining alcohols with ammonia, an abundant and renewable nitrogen source, has been rarely explored, although the related transformations would eliminate prepreparation steps to generate active amino agents, and result in high step and atom efficiency. For instance, the Beller group has reported a Ru-catalyzed synthesis of pyrroles from ammonia, vicinal diols, and ketones (Scheme 1, Equation 1) (Zhang et al., 2013a, Zhang et al., 2013b). Milstein and the co-workers have presented a synthesis of pyrroles and pyrazines from alcohols and ammonia (Scheme 1, Equation 2) (Daw et al., 2018).

In recent years, the so-called hydrogen-borrowing reaction has emerged as an appealing tool in achieving the alkylation of amines (Wang et al., 2014, Xiao et al., 2019, Kaloglu et al., 2016, Elangovan et al., 2016) and activated carbon nucleophiles (Blank and Kempe, 2010, Elangovan et al., 2015, Deibl and Kempe, 2016, Peña-López et al., 2016). Interestingly, the synthesis of various alkylamines from alcohols and ammonia has also been nicely demonstrated (Scheme 1, Equation 3) (Ye et al., 2014, Pingen et al., 2010, Imm et al., 2010, Imm et al., 2011, Gunanathan and Milstein, 2008, Yamaguchi et al., 2008, Kawahara et al., 2010). In such transformations, the alcohols serve as both the hydrogen suppliers and coupling agents. So, there is no need for external reductants such as high-press H_2_ gas. Despite these significant advances, the construction of functional N-heteroarenes involving alcohols and ammonia feedstocks through hydrogen autotransfer as a substrate-activating strategy remains a new subject to be explored. However, such a concept would encounter the challenges of difficult proton exchanges and selectivity control, as well as catalyst deactivation by the lone pair of electrons on the nitrogen of excess ammonia (Klinkenberg and Hartwig, 2011).

Among various N-heteroarenes, quinazolines constitute a class of structurally unique compounds, which have been found to exhibit diverse biological and therapeutic activities (Parhi et al., 2013, Ugale and Bari, 2014, Juvale et al., 2013, Ple et al., 2004), and have been extensively applied for the discovery of various functional products (Zhao et al., 2013, Zhang et al., 2011). However, the existing approaches for accessing such compounds generally require preinstalled reactants (Lin et al., 2014, Malakar et al., 2012, Portela-Cubillo et al., 2008, Yan et al., 2012, Zhang et al., 2010). In this context, the search for direct synthesis of quinazolines from easily available substrates, preferably abundant and sustainable ones, would be of great significance. Enlightened by our recent work on the synthesis and functionalization of N-heterocycles (Chen et al., 2017b, Chen et al., 2018a, Chen et al., 2018b, Chen et al., 2017a, Liang et al., 2018, Liang et al., 2019, Xie et al., 2017, Xie et al., 2018, Xie et al., 2019), we wish herein to present, for the first time, a synthesis of quinazolines from 2-nitrobenzyl alcohols (Rajendran et al., 2015, Pasnoori et al., 2014), alcohols, and ammonia by a new iridium complex featuring a 2-(4-methoxyphenyl)-1,8-naphthyridyl ligand. In such a transformation, the hydrogen generated from dehydrogenation of alcohols and dehydroaromatization process is utilized for substrate activation through transfer hydrogenation (TH) of the nitro group, and there is no need for addition of external reductants.

## Results and Discussion

We initiated our investigations by choosing the synthesis of quinazoline **3aa** from *o*-nitrobenzene methanol **1a**, alcohol **2a**, and ammonia as a model reaction. First, we tested the combinations of several metal catalysts (i.e., Ru, Mn, Co, Fe, and Ni) with various phosphine ligands such as Xantphos, DPPE, DPPB, DPPP, Binap-dp, DPEphos, and Xphos (see Table S1), the privileged catalyst systems employed for the ADC and hydrogen-borrowing reactions. However, the low yields of product (<10%) disclosed that they were not suitable systems for the current synthetic purpose. When complex [IrCp∗Cl_2_]_2_ was employed, 15% yield of **3aa** was obtained. A further optimization of other reaction parameters involving solvents, bases, and temperatures (Table S2) slightly improved the yield to 18% by using *t*-BuONa as the base at 140°C. Enlightened by our recent synthesis of naphthyridines (Chen et al., 2017a, Chen et al., 2017b, Chen et al., 2018a, Chen et al., 2018b, Xiong et al., 2016), we believed that such compounds might serve as a class of useful N-ligands with tunable coordination modes, and the preparation of a suitable naphthyridyl Ir-complex might offer a solution to obtain the desirable catalytic efficiency. Thus, we prepared 9-cyclometalated iridium complexes, involving 8-naphthyridyl (**Ir-1**−**Ir-8**) and 1,2-phenylpyridyl (**Ir-9**) ones. Then, their catalytic performance toward the model reaction was evaluated (Table 1, entries 1–9). In comparison, complexes bearing a 1,8-naphthyridyl ligand (entries 1–7) exhibited appealing activity, and **Ir-3** (as confirmed by single-crystal X-ray diffraction, CCDC: 1848110, for detail, see Figure S101 and Tables S5–S10) was shown to be a preferred candidate, whereas complex with a 1,5-naphthyridyl or 2-pheynlpyridyl ligand only resulted in low product yield (entries 8–9). The results imply that the N-atom at position 8 in 1,8-naphthyridyl ligands plays a crucial role in affording a satisfactory product yield. Further optimization showed that the presence of iridium is essential in affording the product (entry 10), and the gaseous ammonia is relatively superior to other nitrogen sources (entries 11–15). A decrease of base amount to 30% resulted in a diminished yield (entry 16), and 40% *t*-BuONa was sufficient for the reaction (entry 17). The time-conversion profile at 2, 4, 8, and 16 h showed that the satisfactory product yield is due to the catalyst robustness (entry 18). Based on the results, the optimal (standard) conditions are as indicated in entry 17 of Table 1.

**Table 1:** Screening of Optimal Reaction Conditions


Entry	Catalyst	NH_3_ Source	Yields of 3aa^a^^,^^b^
1	**Ir-1**	NH_4_OAc	72
2	**Ir-2**	NH_4_OAc	75
3	**Ir-3**	NH_4_OAc	82
4	**Ir-4**	NH_4_OAc	61
5	**Ir-5**	NH_4_OAc	67
6	**Ir-6**	NH_4_OAc	71
7	**Ir-7**	NH_4_OAc	68
8	**Ir-8**	NH_4_OAc	15
9	**Ir-9**	NH_4_OAc	21
10	–	NH_4_OAc	–
11	**Ir-3**	NH_4_Cl	5
12	**Ir-3**	HCOONH_4_	Trace
13	**Ir-3**	NH_3_⋅H_2_O	Trace
14	**Ir-3**	(NH_4_)_2_SO_4_	22
15	**Ir-3**	NH_3_ (g)	88^c^
16	**Ir-3**	NH_3_ (g)	81^c^^,^^d^
17	**Ir-3**	NH_3_ (g)	88^c^^,^^e^
18	**Ir-3**	NH_3_ (g)	(12, 40, 65, 84)^f^

Also see Figure S101, Tables S5−S10 and Data S3.

a Unless otherwise stated, the reaction was performed with **1a** (0.5 mmol), **2a** (0.5 mmol), Ir (1 mol %), *t*-BuONa (50 mol %), NH_3_ sources (1.0 mmol) in toluene (1.5 mL) for 24 h under Ar protection.

b Gas chromatography yields with the use of hexadecane as an internal standard.

c 4 bar of NH_3_.

d *t*-BuONa (30 mol %).

e *t*-BuONa (40 mol %).

f Conversions for 2, 4, 8, and 16 h.

**Scheme 2:**
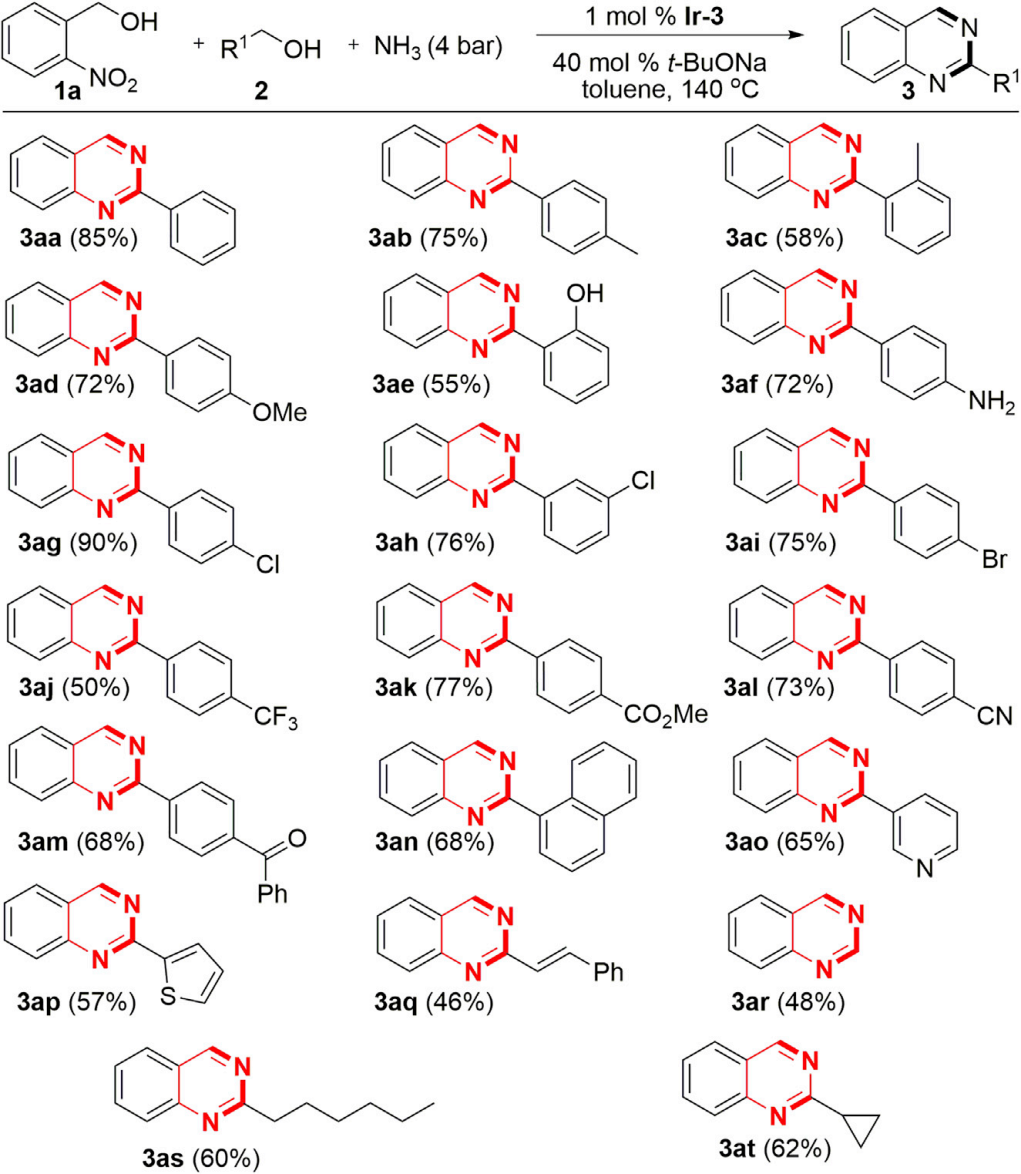
Variation of Alcohols Also see Scheme S1, Figures S1−S60 and Data S3.

With the optimal reaction conditions established, we then examined the generality of the synthetic protocol. (2-nitrophenyl)methanol **1a** was further employed to couple with various primary alcohols (**2a**−**2t**, Scheme S1) and ammonia. As illustrated in Scheme 2, all the reactions proceeded smoothly and furnished the desired quinazolines in moderate to excellent yields upon isolation (Scheme 2, **3ab**−**3at**). Apart from the alkyl-substituted benzyl alcohols, other functional groups such as −OMe, −OH, −NH_2_, −Cl, −Br, −CF_3_, −CO_2_Me, −COPh, −CN, and −C=C− are well tolerated in the transformation. The retention of these functionalities offers the potential for the elaboration of complex molecules via further chemical transformations. Moreover, except for the strong electron-withdrawing group −CF_3_, the electronic property of these substituents has little influence on the reaction, whereas the relatively lower product yields using ortho-substituted benzyl alcohols might relate to the steric hindrance (**3ac** and **3ae**). Furthermore, heteroaryl methanols (**2o** and **2p**) were also amenable to the transformation and resulted in the 2-heteroaryl-substituted quinazolines (**3ao** and **3ap**) in good yields, and the obtained products have the potential to be applied as hemilabile bidentate ligands in organometallic chemistry and catalysis. Interestingly, cinnamyl alcohol **2q** underwent smooth hydrogen transfer-mediated annulation, affording the 2-alkenyl quinazoline **3aq** in 46% yield. The relatively low product yield is due to partial formation of 2-alkyl quinazoline via reduction of the alkenyl group. The relatively low product yield of **3aq** is due to the partial formation of 2-alkyl quinazoline via reduction of the alkenyl group. Aliphatic alcohols, such as methanol (**2r**), heptan-1-ol (**2s**), and cyclopropyl carbinol (**2t**), were efficiently transformed into the 2-non-substituted and 2-alkyl quinazolines (**3ar**, **3as,** and **3at**) in moderate yields.

**Scheme 3:**
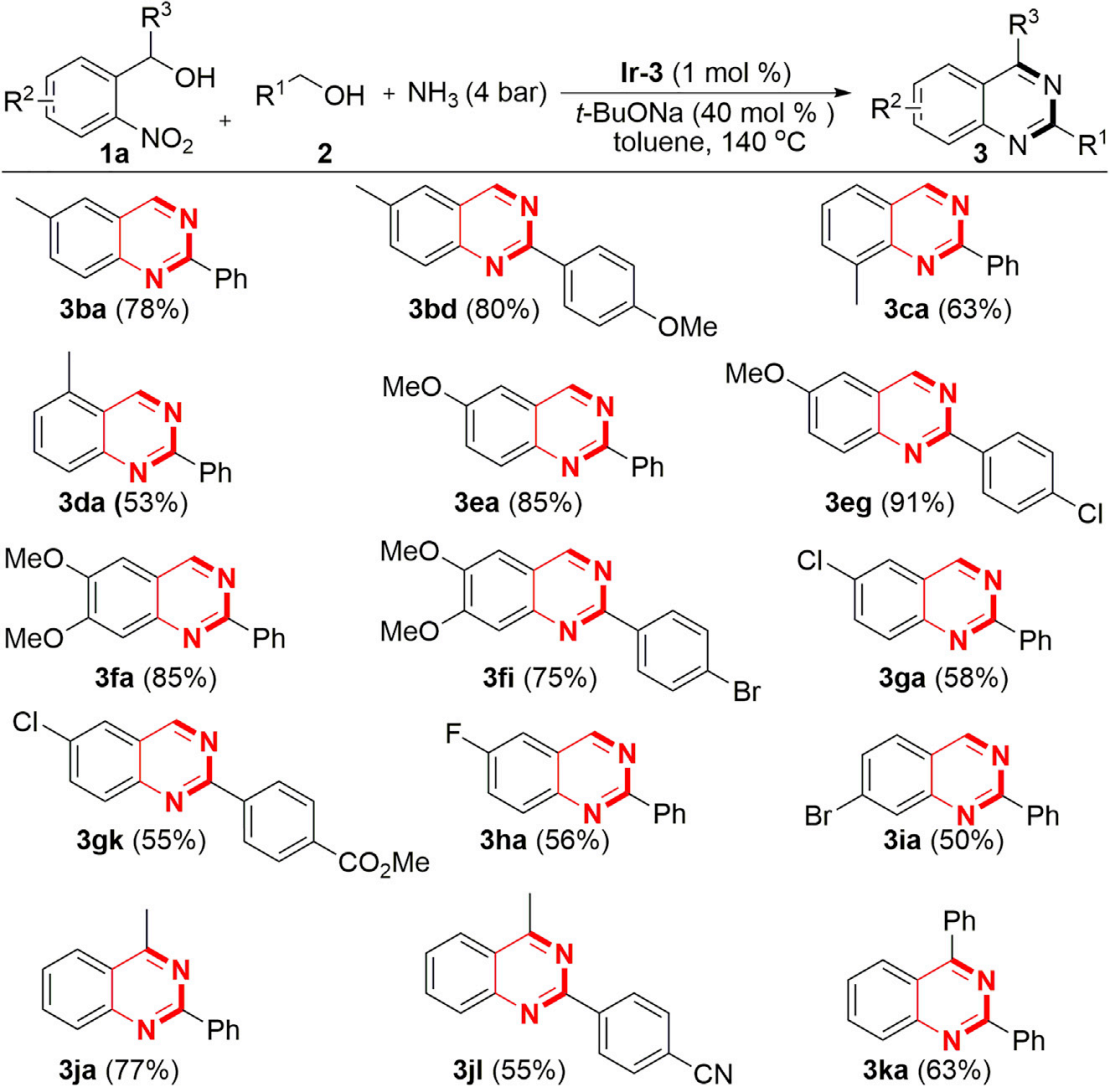
Variation of *o*-Nitroaryl Alcohols Also see Scheme S1, Figures S61−S91 and Data S3.

Subsequently, we turned our attention to the transformation of different 2-nitrobenzyl alcohols **1**. First, various related substrates (**1b**−**1i**) in combination with different primary alcohols **2** and NH_3_ were tested. As shown in Scheme 3, all the reactions smoothly delivered the multi-substituted quinazolines in moderate to excellent isolated yields. The electronic property of the substituents on the aryl ring of substrates **1** significantly influenced the product yields. In general, 2-nitrobenzyl alcohols **1** with electron-donating groups afforded the products in higher yields (**3ba**–**3ca** and **3ea**–**3fi**) than with electron-deficient ones (**3ga**–**3ia**). This phenomenon is rationalized as the catalyst has better stability toward the electron-rich aniline intermediates, arising from the TH of nitro group of substrates **1**. Gratifyingly, secondary alcohols, such as **1j** and **1k**, also underwent smooth annulation to give the 2,4-disubstituted quinazolines in good yields (**3ja**, **3jl**, and **3ka**). Similar to the results described in Scheme 2, a wide array of functionalities such as −Me, −OMe, −F, −Cl, −Br, −CN, −Ph, and ester are well tolerated in the transformation (Schemes 2 and 3). Noteworthy, the halogen groups did not undergo hydrodehalogenation, showing that the developed catalytic system exhibits unique chemoselectivity.

**Scheme 4:**
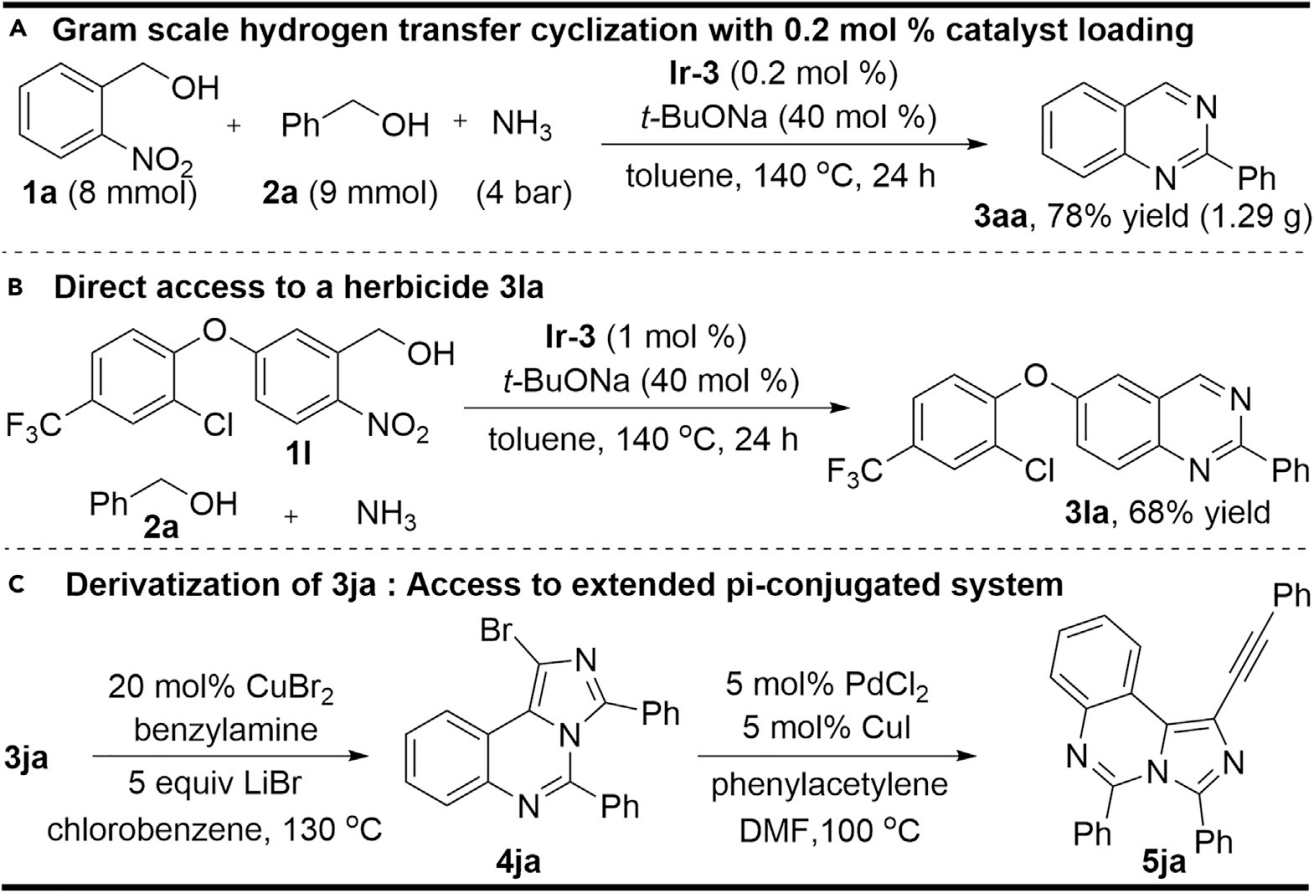
The Synthetic Utility of the Developed Chemistry Also see Scheme S1 and S3, Figures S92−S97 and Data S3.

To demonstrate the significance and practicality of the developed synthetic methodology, a gram-scale synthesis of compound **3aa** could be achieved by performing the reaction with 8 mmol of **1a** and 9 mmol of benzyl alcohol **2a**, which still afforded a good isolated product yield (78%) even with lower catalyst loading (Scheme 4, Equation a, 0.2 mol%). Furthermore, compound **3la**, a key ingredient used as a herbicide with the activity on Toll-like receptors, **20** could be prepared through the reduction of commercially available acifluorfen acid to 2-nitrobenzyl alcohol **1l** (Scheme S3) followed by the annulation reaction of **1l** with alcohol **2a** and ammonia (Equation b), and such a synthesis is far superior to the reported multi-step synthetic protocol (Mc Gowan et al., 2012, Munro and Bit, 1987, Sumida et al., 1995). Moreover, the extended π-conjugated system like compound **5ja** was successfully prepared by the halocyclization (Tan et al., 2016) of compound **3ja** and further Sonogashira coupling (Equation c), which offers a valuable basis for further development of optoelectronic materials.

**Figure 1:**
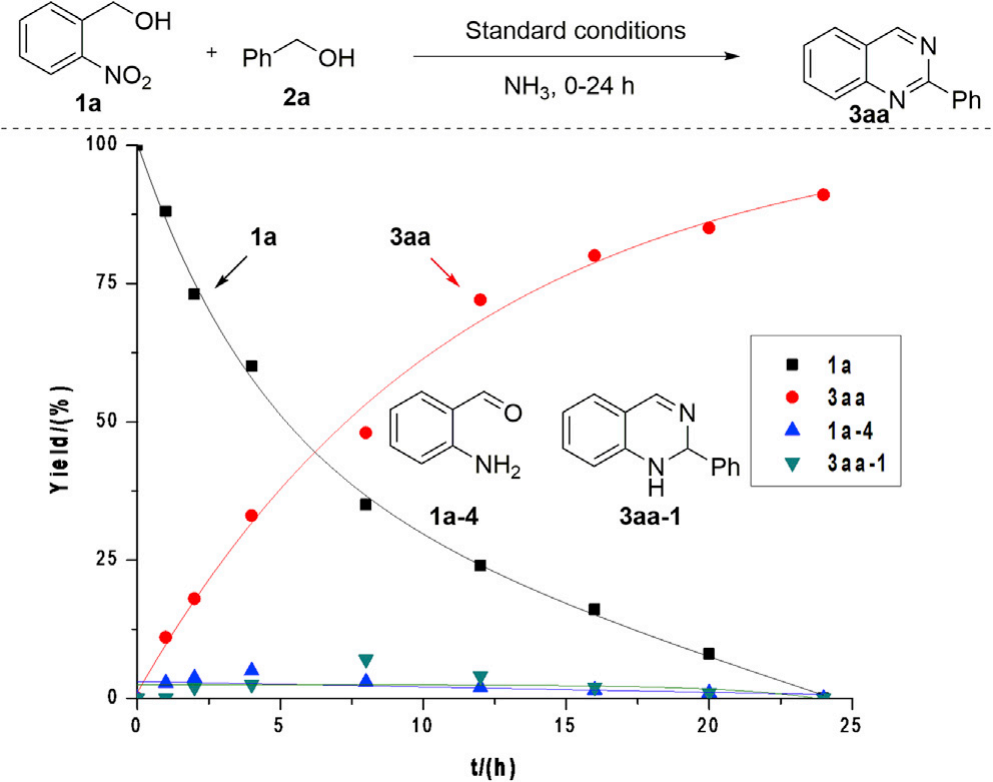
Time-Concentration Profile of the Model Reaction

To gain mechanistic insights into the catalytic transformation, a time-concentration profile of the model reaction is depicted in Figure 1 (also see Data S1). Substrates **1a** and **2a** with ammonia were converted into **3aa** in maximum yield within 24 h. 2-Aminobenzaldehyde **1a-4** and 1,2-dihydroquinazoline **3aa-1** were observed during the reaction, but they were consumed up after completion of the reaction (Figure 1). The subjection of compound **1a-4** with benzaldehyde **2a-1** and NH_3_ or direct treatment of **3aa-1** under the standard conditions afforded product **3aa** in almost quantitative yields (see Equations 1 and 2 of Scheme S2, also see Data S1). These results support the fact that compounds **1a-4**, **2a-1,** and **3aa-1** are the reaction intermediates. Furthermore, both the iridium catalyst and base play crucial roles in the dehydrogenation of **3aa-1** to product **3aa** (Equation 2). An iridium hydride complex (**Ir-H**) was obtained from the reaction of equimolar **Ir-3** and benzyl alcohol, which can efficiently catalyze the reaction to afford **3aa**, showing that **Ir-H** as a catalytic species is involved in the reaction (Equations 3 and 4, Scheme S2, also see Data S2 and Figure S98).

**Figure 2:**
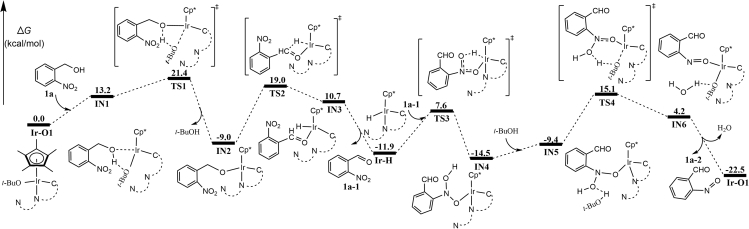
Calculated Energy Profiles for First TH *o*-Nitrobenzene methanol **1a** → 2-nitrosobenzaldehyde **1a-2**. Values shown are relative free energies in kcal/mol. Also see Tables S3 and S4 and Data S4.

With the above-mentioned preliminary experimental evidence in hand, the mechanism was further scrutinized by density functional theory calculations (geometry optimizations using B3LYP and single-point energy calculations using M06). For details, see Figures S99 and S100, Tables S3 and S4, Schemes S4–S8, and Data S4. The calculated free energy profile for the first TH (first TH) of **1a** to 2-nitrosobenzaldehyde **1a-2** is shown in Figure 2. Initially, the anion exchange between **Ir-3** and *t*-BuONa generates the alkoxy complex **Ir-O1**. One of the arms in 1,8-naphthyridyl ligand of **Ir-O1** dissociates, allowing the Ir center to coordinate with the hydroxyl group of **1a**. O–H bond cleavage occurs via transition state **TS1** with an energy barrier of 21.4 kcal/mol to give Ir-alkoxide complex **IN2**, which then undergoes β-hydride elimination by overcoming an energy barrier of 28.0 kcal/mol (**TS2**) relative to **IN2**, and generates complex **Ir-H** and *o*-nitrobenzaldehyde **1a-1**. The nitro group of **1a-1** further acts as a sacrificial hydrogen acceptor of **Ir-H** through two transition states **TS3** and **TS4**. Finally, 2-nitrosobenzaldehyde **1a-2** is generated with the regeneration of **Ir-O1**. In addition, the base-promoted intramolecular Meerwein-Ponndorf-Verley-Oppenauer-type transfer hydrogenation (MPV-O TH) is calculated to have an energy barrier of 33.1 kcal/mol (see Scheme S4), which is 3.5 kcal/mol higher than the overall barrier of the pathway shown in Figure 2. Thus, the MPV-O TH pathway is kinetically unfavorable.

**Figure 3:**
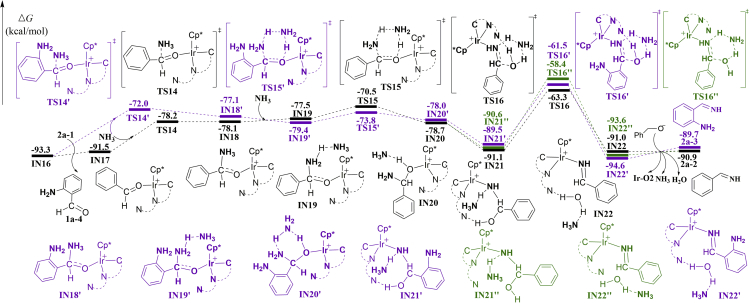
Calculated Energy Profiles for Coupling of Alcohol with Ammonia Black line for benzaldehyde **2a-1** and purple line for 2-aminobenzaldehyde **1a-4**. The dehydration without the assistance of the non-coordinated N-atom in the ligand is shown in green line. Values shown are relative free energies in kcal/mol. Also see Tables S3 and S4 and Data S4.

The calculated free energy profiles for the second TH of 2-nitrosobenzaldehyde **1a-2** to 2-(hydroxyamino)benzaldehyde **1a-3** and the third TH of **1a-3** to 2-aminobenzaldehyde **1a-4** are shown in Figures S99 and S100 (also see Tables S3 and S4, Schemes S5–S8 and Data S4). In consideration that both 2-aminobenzaldehyde **1a-4** and benzaldehyde **2a-1** can condense with ammonia, two plausible pathways toward the formation of imines were investigated. For the reaction of **2a-1** and ammonia (black line in Figure 3), ammonia approaches benzaldehyde through the C–N bond linkage (**TS14**) giving **IN18**. The TH of the ammonia using other ammonia as the proton-transferring shuttle then takes place via **TS15** and leads to **IN20**. The calculated free energy barrier of transition state **TS15** is 22.8 kcal/mol relative to **IN16**. After rearranging to more stable **IN21** featuring two hydrogen bonds, the dehydration occurs via **TS16**, giving the imine complex **IN22**. Meanwhile, we performed calculations for the dehydration without the hydrogen-bonding between the OH group and the non-coordinated N-atom in the ligand (green line in Figure 3). The calculated free energy of transition state **TS16″** is −58.4 kcal/mol, which is higher than that of **TS16**. Therefore, the non-coordinated N-atom in the 1,8-naphthyridyl ligand plays a crucial role in the reaction, as it serves as a side-arm to significantly promote the dehydration by hydrogen bonding. An alkoxyl anion ligand rebounds to Ir center to give imine **2a-2** with regeneration of the **Ir-O2** catalyst. The reaction of **1a-4** and ammonia (purple line in Figure 3) follows similar mechanisms to those for **2a-1**. The relevant mechanistic details are therefore not discussed again, for simplicity. The highest energy point for the reaction of **1a-4** and ammonia is **TS16′**, which is energetically less favorable by 1.8 kcal/mol compared with that of **TS16** for the reaction of **2a-1** and ammonia. Therefore, from a kinetic point of view, the reaction of **1a-4** and ammonia is less kinetically favorable.

**Scheme 5:**
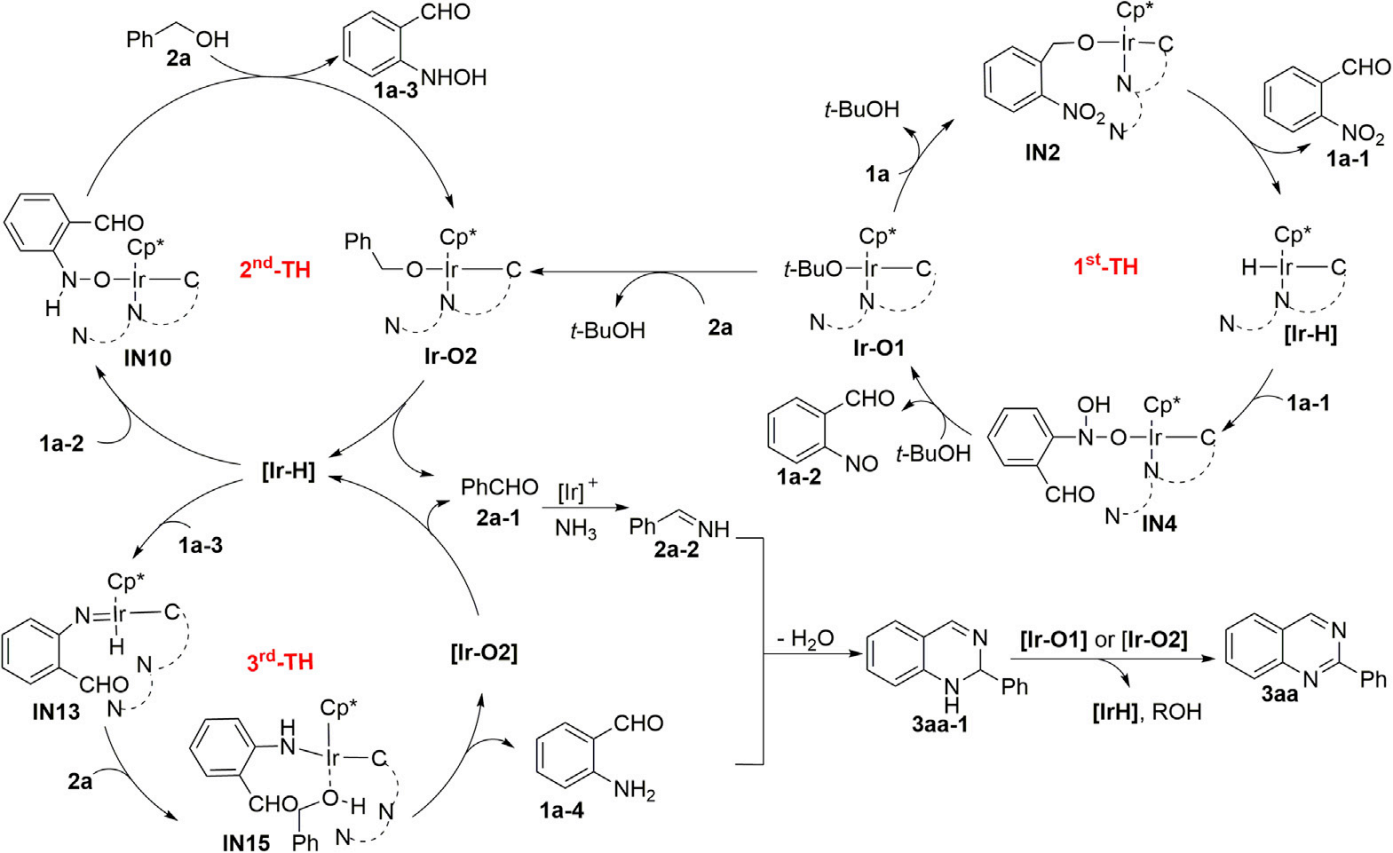
Plausible Reaction Pathway Also see Data S1 and S2 and Figure S98.

Based on all the above-mentioned findings, a plausible reaction pathway for the formation of product **3aa** is illustrated in Scheme 5. In the first TH process, the Ir-catalyzed dehydrogenation of **1a** via alkoxy anion exchange of **Ir-O1** with **1a** gives **IN2**, which is followed by β-H elimination to form the 2-nitrobenzaldehye **1a-1** and the **Ir–H** species. The successive TH to the nitro group of **1a-1** and *t*-BuOH-assisted dehydration forms 2-nitrosobenzaldehyde **1a-2** and regenerates the **Ir-O1** species. In the second TH process, the anion exchange of **Ir-O1** with **2a** gives a benzyloxy complex **Ir-O2**. The subsequent β-H elimination of **Ir-O2** followed by TH to the nitroso group and alcoholysis with **2a** delivers 2-(hydroxyamino)benzaldehyde **1a-3** and regenerates complex **Ir-O2**, respectively. In the third TH process, the **Ir-H** and benzaldehyde **2a-1** are generated via β-H elimination of **Ir-O2**. Subsequently, the Ir-promoted dehydration of **1a-3** forms a nitrene complex **IN13**, and the TH using **2a** as the proton-transferring shuttle generates 2-aminobenzaldehyde **1a-4** (Qu et al., 2014, Hou et al., 2017). Next, the successive formation of imine **2a-2** via the condensation of benzaldehyde **2a-1** with NH_3_ and the cyclization between **2a-2** and **1a-4** affords the dihydroquinazoline **3aa-1**. Finally, the iridium alkoxy complex-catalyzed dehydroaromatization of **3aa-1** gives rise to product **3aa**, and the *in situ*-generated **Ir-H** and alcohol further take part in the TH of the nitro group.

### Conclusion

In summary, we have prepared a series of cyclometalated iridium complexes. Among them, **Ir-3** featuring a 2-(4-methoxyphenyl)-1,8-naphthyridyl ligand exhibits the best catalytic performance toward the hydrogen transfer-mediated annulation of 2-nitrobenzyl alcohols with readily available alcohols and ammonia, which allows direct synthesis of a wide array of valuable quinazolines. Mechanistic investigation suggests that the non-coordinated N-atom in the ligand serves as a side arm to significantly promote the condensation step by hydrogen bonding. The catalytic transformation proceeds with the striking features of good substrate and functional group compatibility, liberation of H_2_O as the sole by-product, high atom and step efficiency, and no need for additional reductants. The developed chemistry paves the avenues for further development of hydrogen transfer-mediated coupling reactions by design of catalysts bearing N-side arm ligands.

## Methods

All methods can be found in the accompanying Transparent Methods supplemental file.

## Data and Software Availability

The crystallography data have been deposited at the Cambridge Crystallographic Data Center (CCDC) under accession number CCDC: 1848110 (**Ir-3**) and can be obtained free of charge from www.ccdc.cam.ac.uk/getstructures.

## Supplemental Information

Document S1. Transparent Methods, Figures S1–S101, Tables S1–S10, Schemes S1–S8, and Data S1–S4
