# Supplementary material for: Hydrogen Transfer-Mediated Multicomponent Reaction for Direct Synthesis of Quinazolines by a Naphthyridine-Based Iridium Catalyst
Source: iScience. 2020 Mar 21;23(4):101003. doi: 10.1016/j.isci.2020.101003 (PMC7150509; doi:10.1016/j.isci.2020.101003)
Supplement: Document S1. Transparent Methods, Figures S1–S101, Tables S1–S10, Schemes S1–S8, and Data S1–S4 [file mmc1.pdf]

iScience, Volume *23*

Supplemental Information

Hydrogen Transfer-Mediated Multicomponent Reaction for Direct Synthesis of Quinazolines by a Naphthyridine-Based Iridium Catalyst

Zhenda Tan, Zhongxin Fu, Jian Yang, Yang Wu, Liang Cao, Huanfeng Jiang, Juan Li, and Min Zhang

Copies of product NMR spectra

Figure S1. ^{1}H-NMR (400 MHz, $CDCl_3$) spectrum of **Ir-1**, related to **Table 1**.

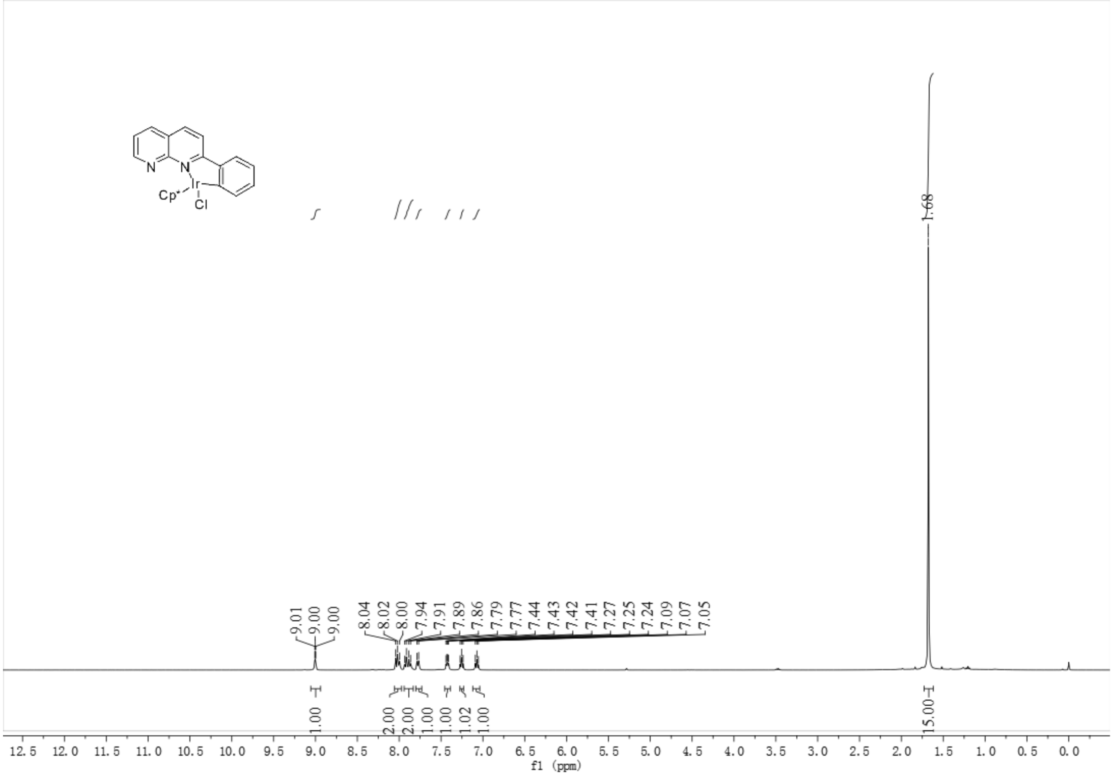

Figure S2. ^{13}C-NMR (100 MHz, $CDCl_3$) spectrum of **Ir-1**, related to **Table 1**.

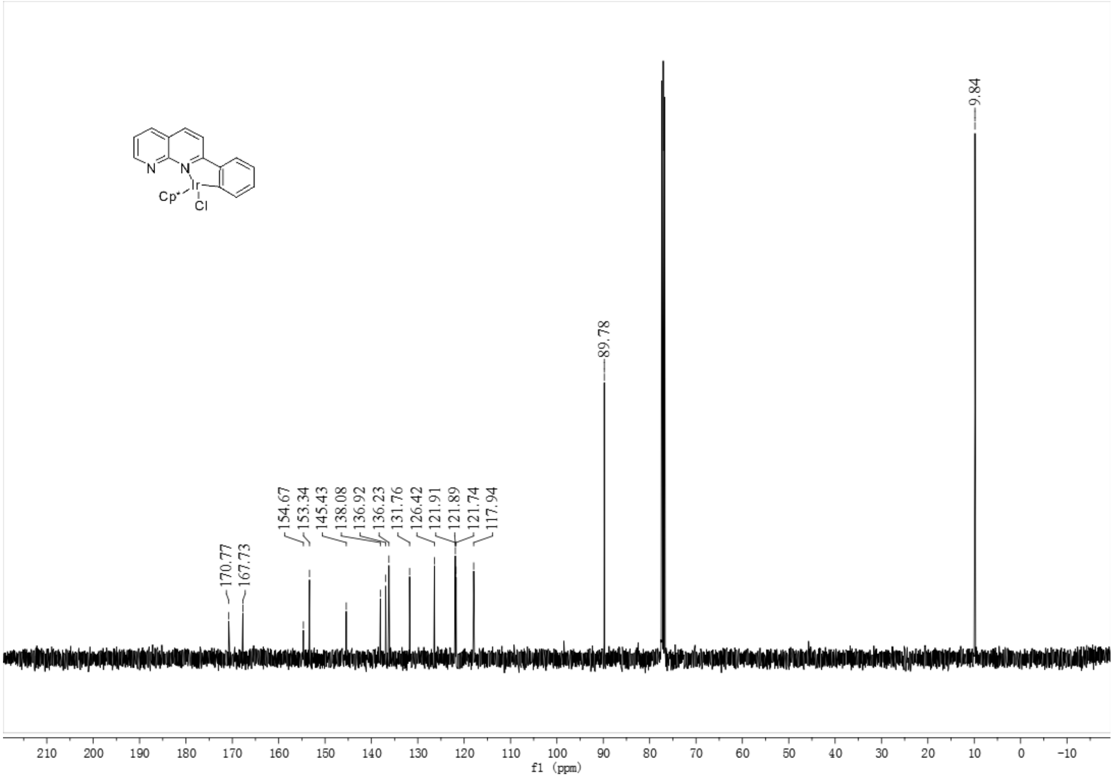

Figure S3. ^{1}H-NMR (400 MHz, $CDCl_3$) spectrum of **Ir-2**, related to **Table 1**.

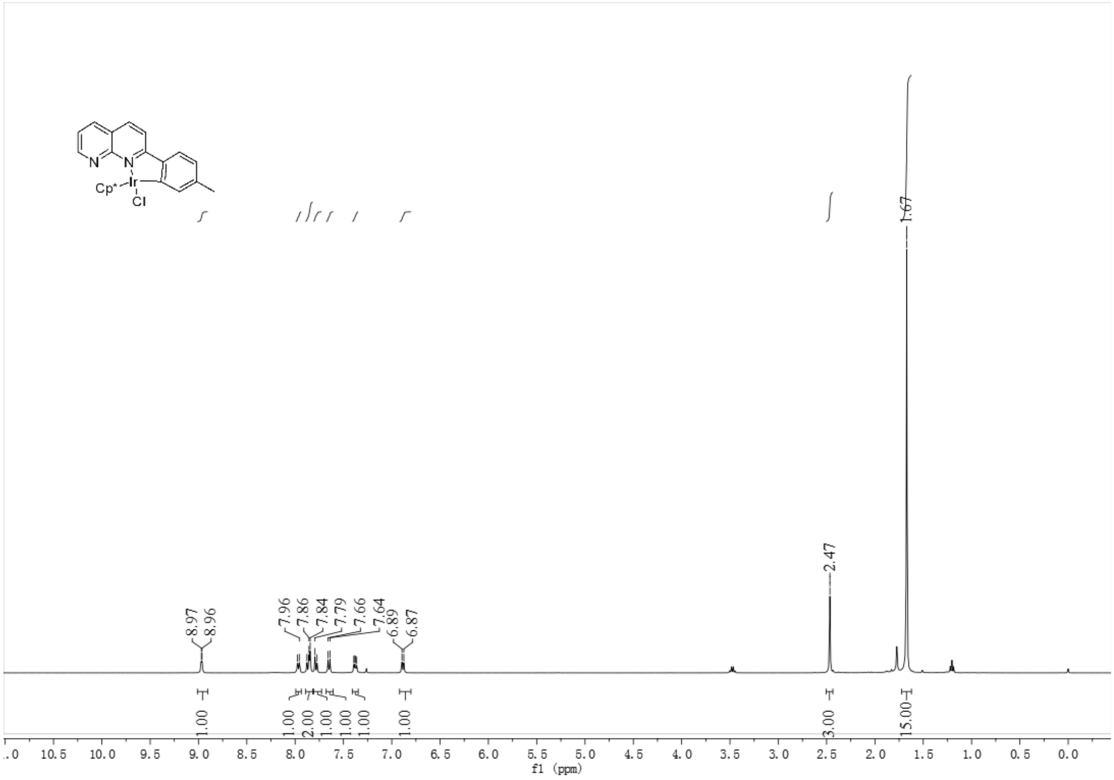

Figure S4. ^{13}C-NMR (100 MHz, $CDCl_3$) spectrum of **Ir-2**, related to **Table 1**.

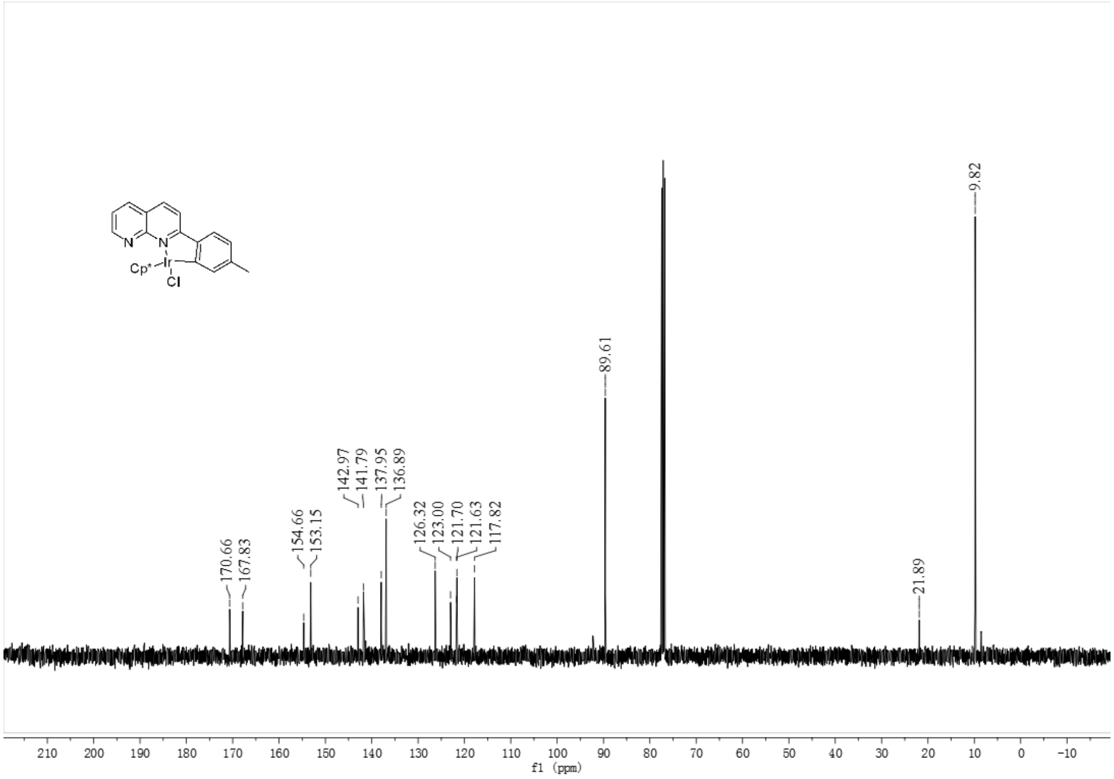

Figure S5. ^{1}H-NMR (400 MHz, $CDCl_3$) spectrum of **Ir-3**, related to **Table 1**.

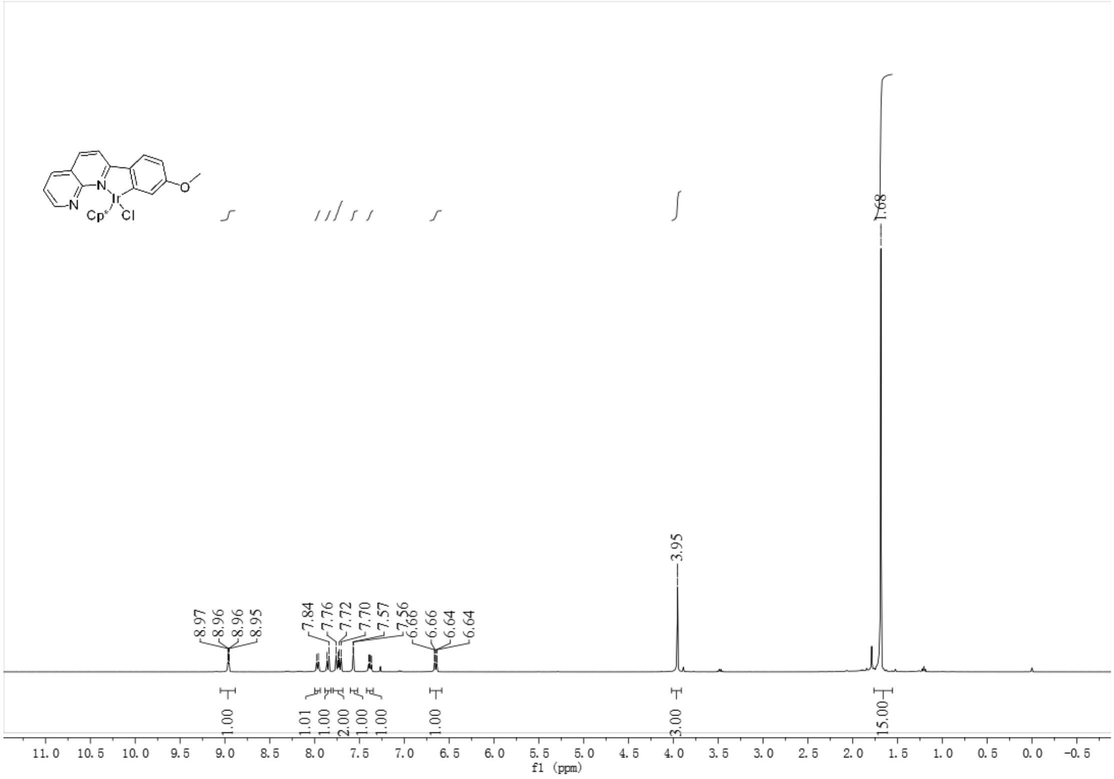

Figure S6. ^{13}C-NMR (100 MHz, $CDCl_3$) spectrum of **Ir-3**, related to **Table 1**.

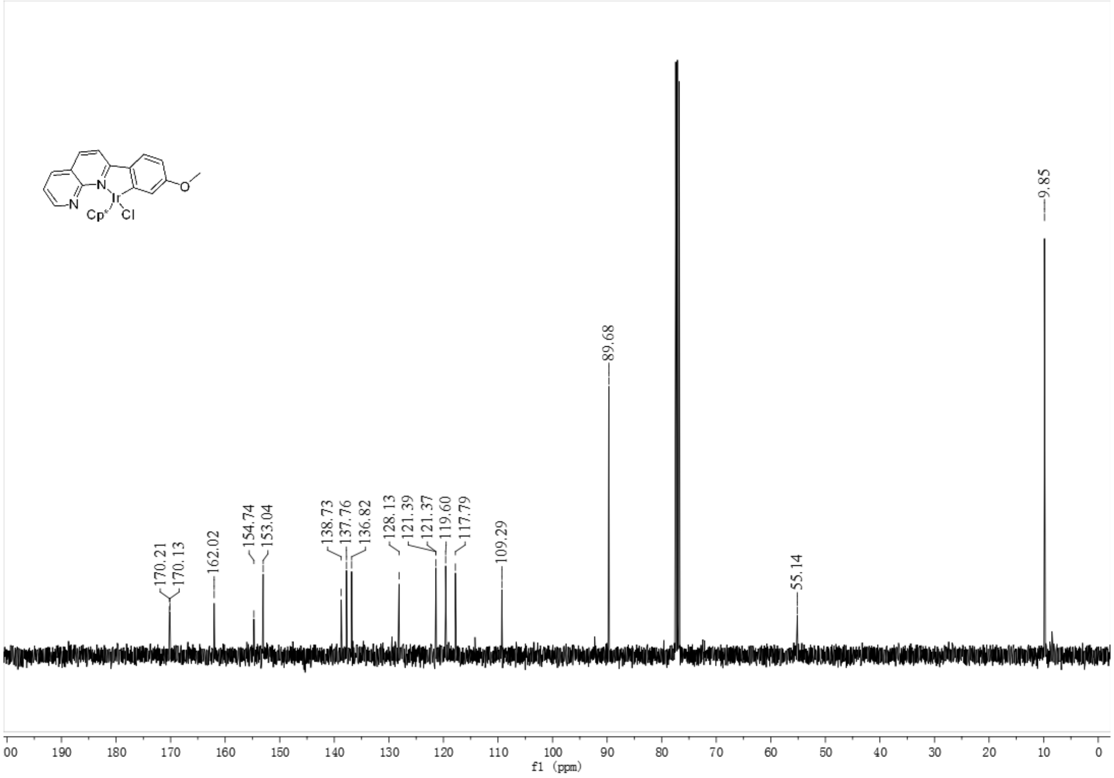

Figure S7. ^{1}H-NMR (400 MHz, $CDCl_3$) spectrum of **Ir-4**, related to **Table 1**.

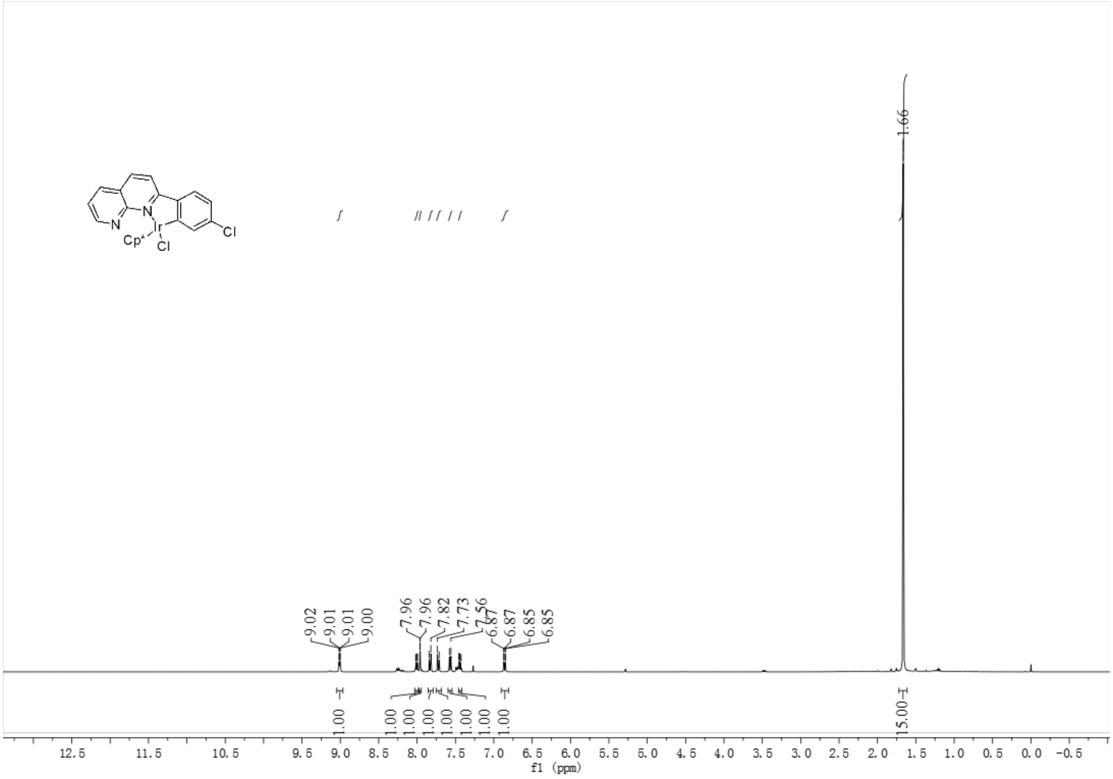

Figure S8. ^{13}C-NMR (100 MHz, $CDCl_3$) spectrum of **Ir-4**, related to **Table 1**.

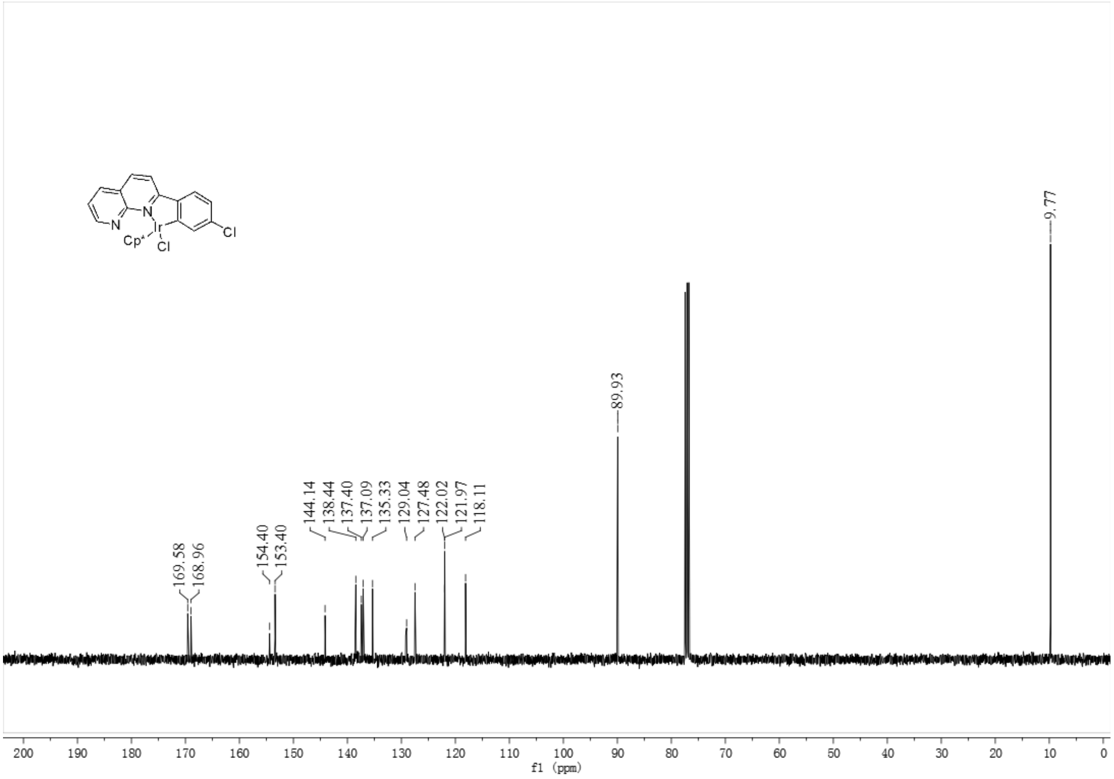

Figure S9. ^{1}H-NMR (400 MHz, $CDCl_3$) spectrum of **Ir-5**, related to **Table 1**.

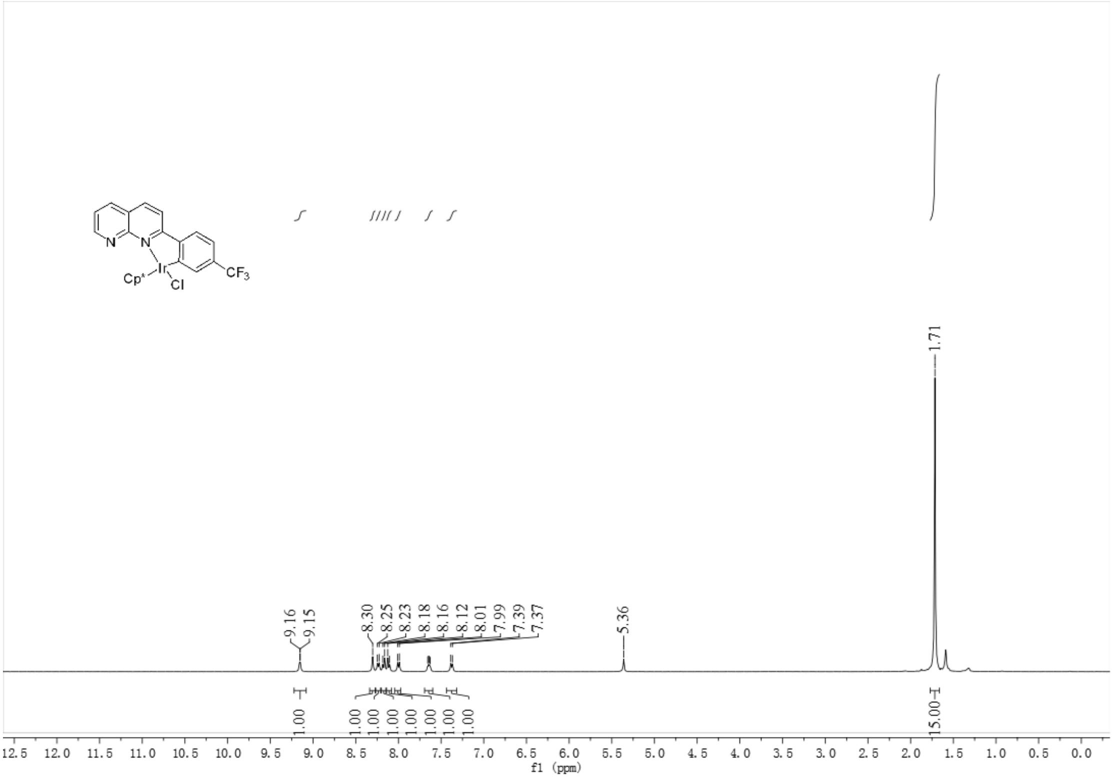

Figure S10. ^{13}C-NMR (100 MHz, $CDCl_3$) spectrum of **Ir-5**, related to **Table 1**.

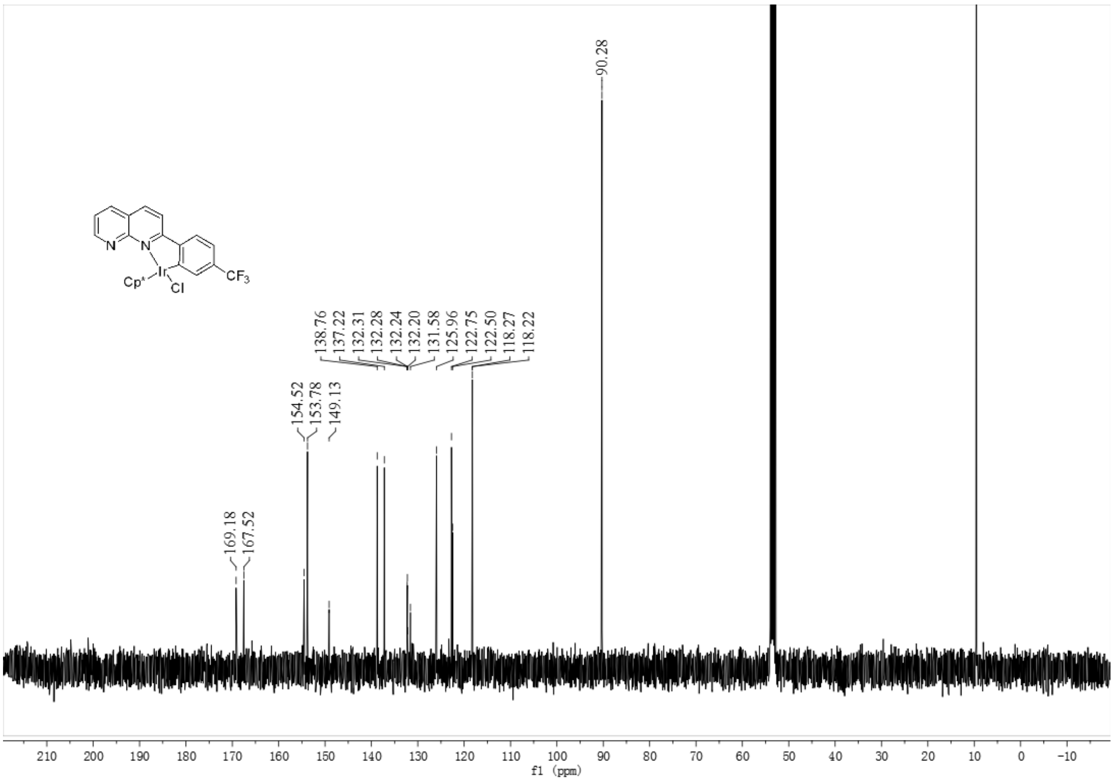

Figure S11. ^{19}F-NMR (400 MHz, $CDCl_3$) spectrum of **Ir-5**, related to **Table 1**.

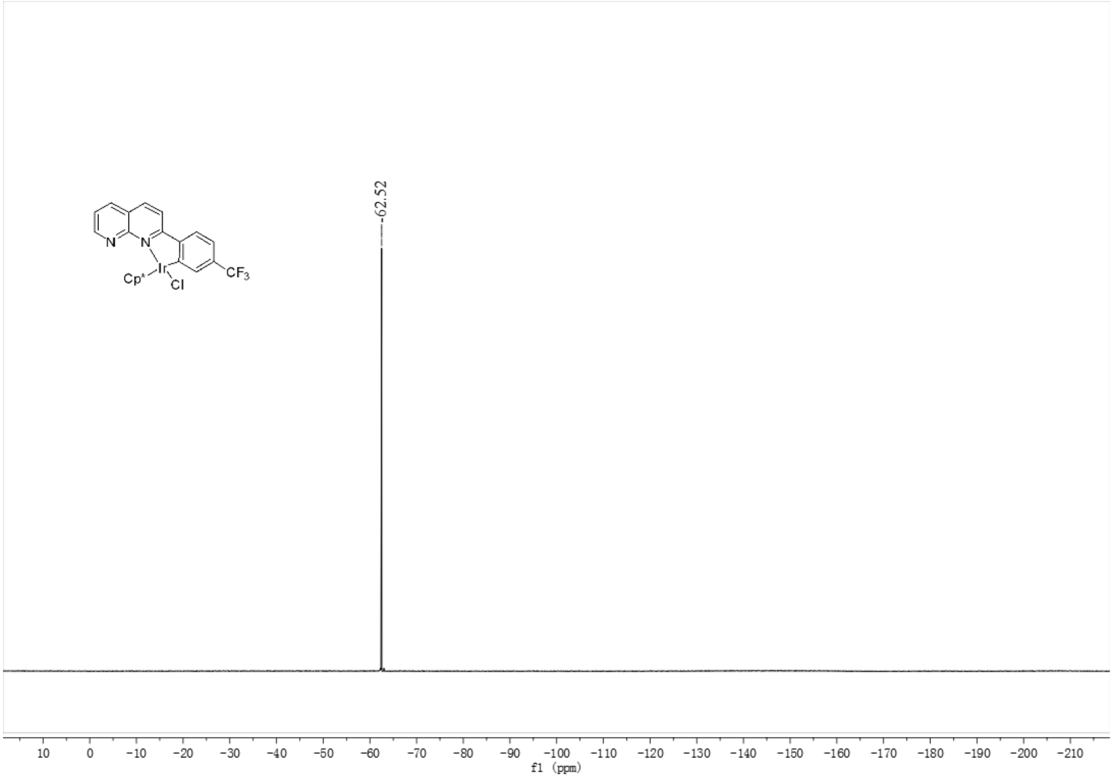

Figure S12. ^{1}H-NMR (400 MHz, $CDCl_3$) spectrum of **Ir-6**, related to **Table 1**.

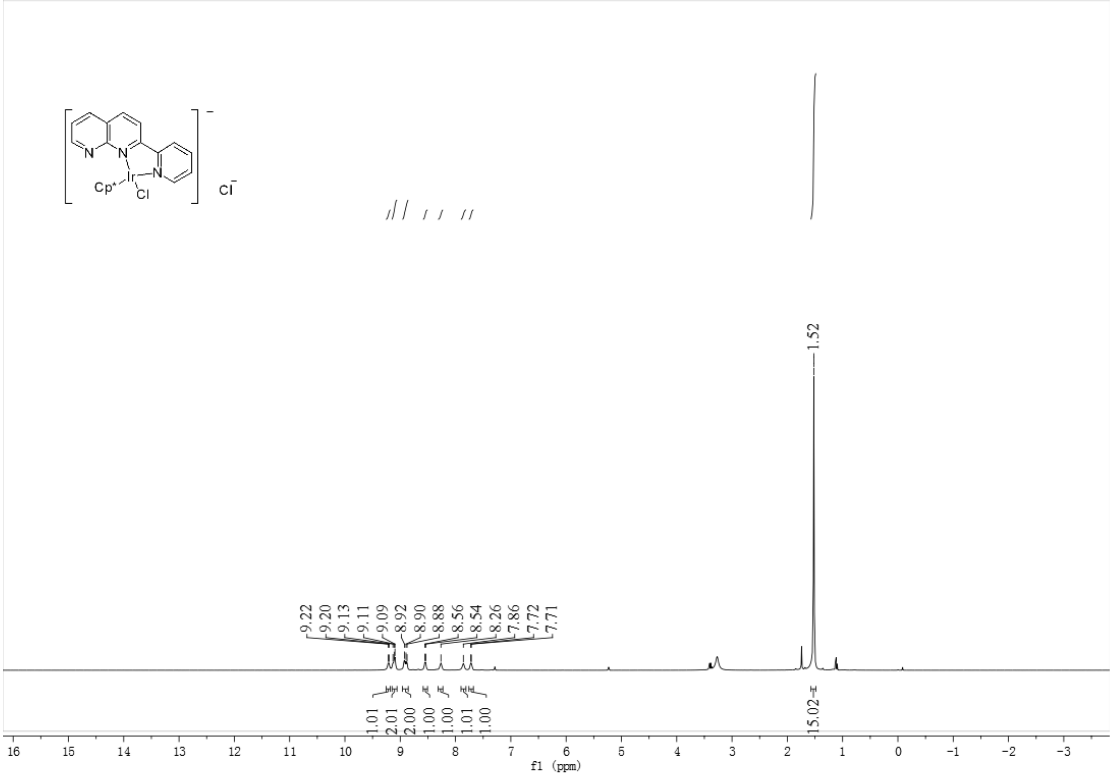

Figure S13. ^{13}C-NMR (400 MHz, $CDCl_3$) spectrum of **Ir-6**, related to **Table 1**.

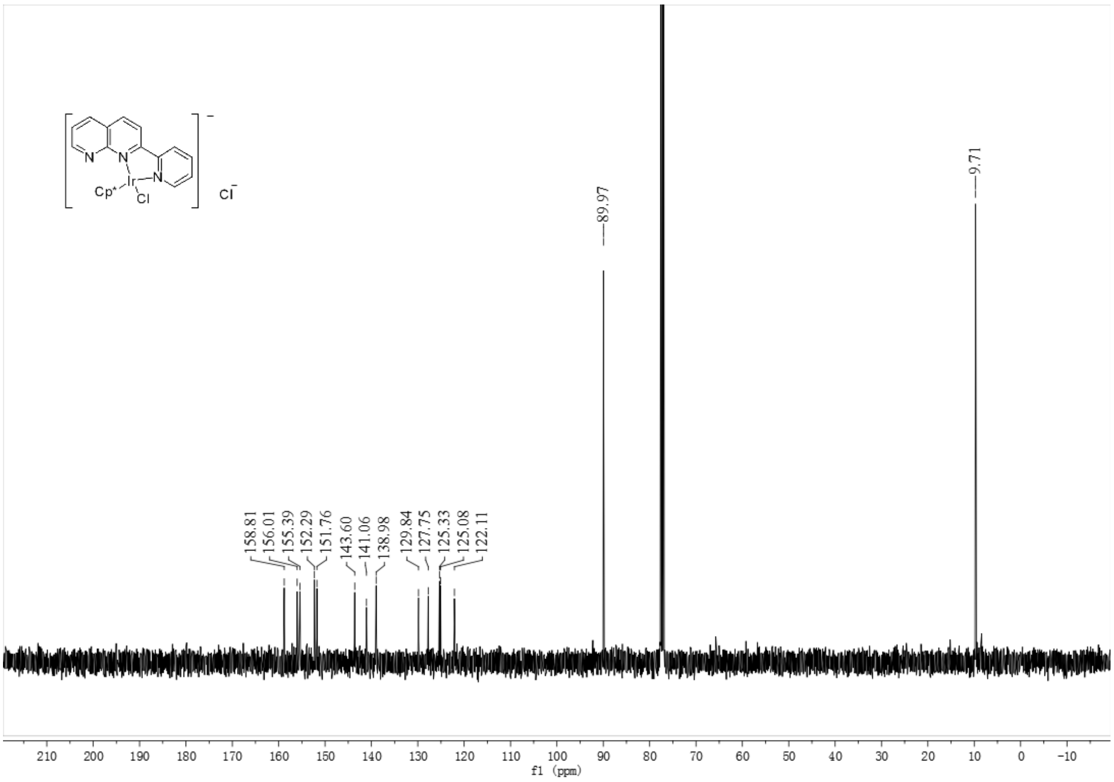

Figure S14. ^{1}H-NMR (400 MHz, $CDCl_3$) spectrum of **Ir-7**, related to **Table 1**.

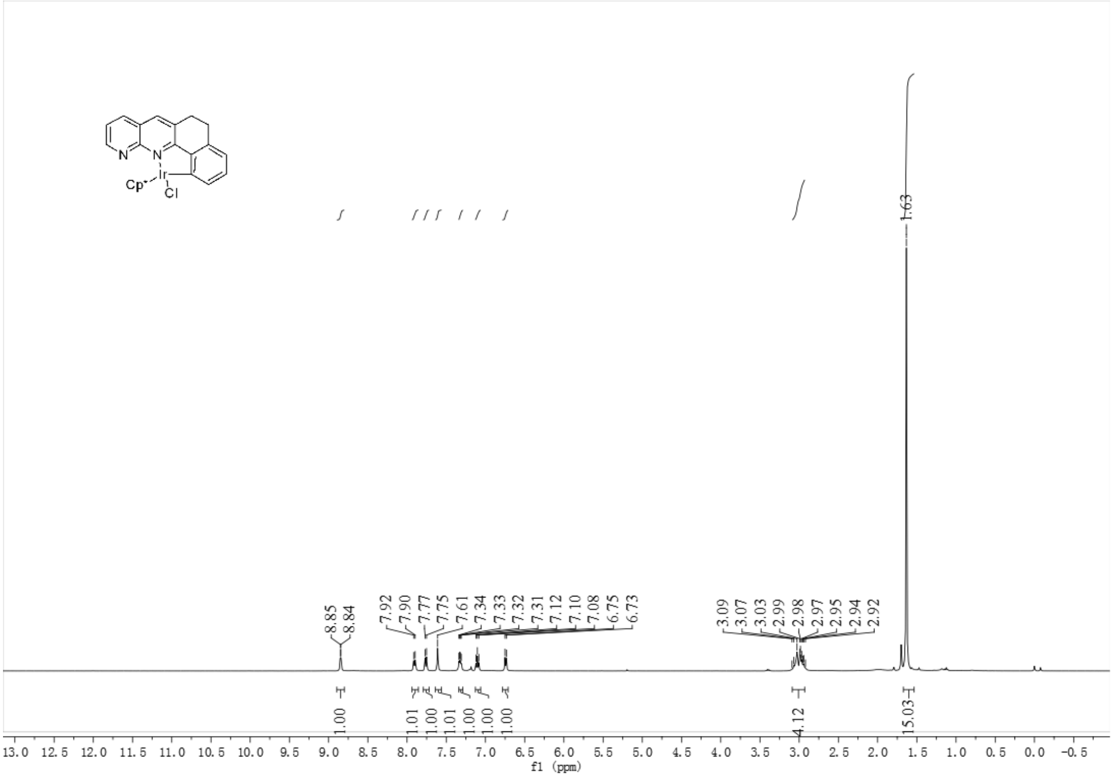

Figure S15. ^{13}C-NMR (100 MHz, $CDCl_3$) spectrum of **Ir-7**, related to **Table 1**.

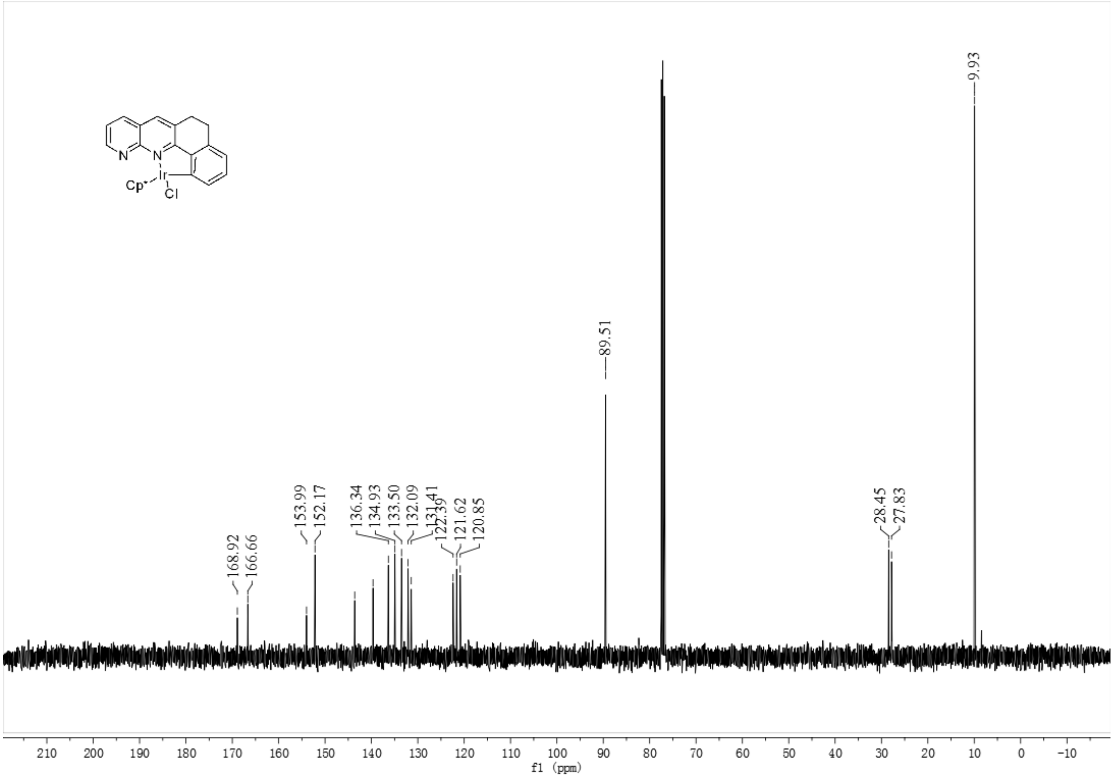

Figure S16. ^{1}H-NMR (400 MHz, $CDCl_3$) spectrum of **Ir-8**, related to **Table 1**.

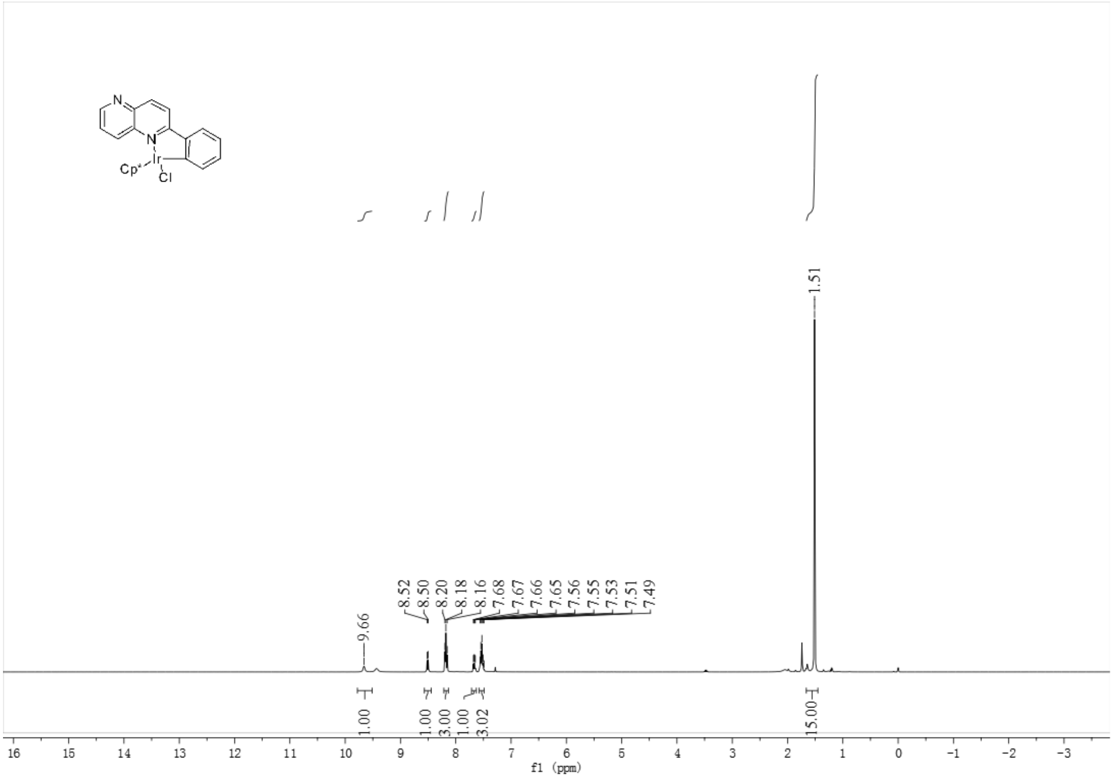

Figure S17. ^{13}C-NMR (100 MHz, $CDCl_3$) spectrum of **Ir-8**, related to **Table 1**.

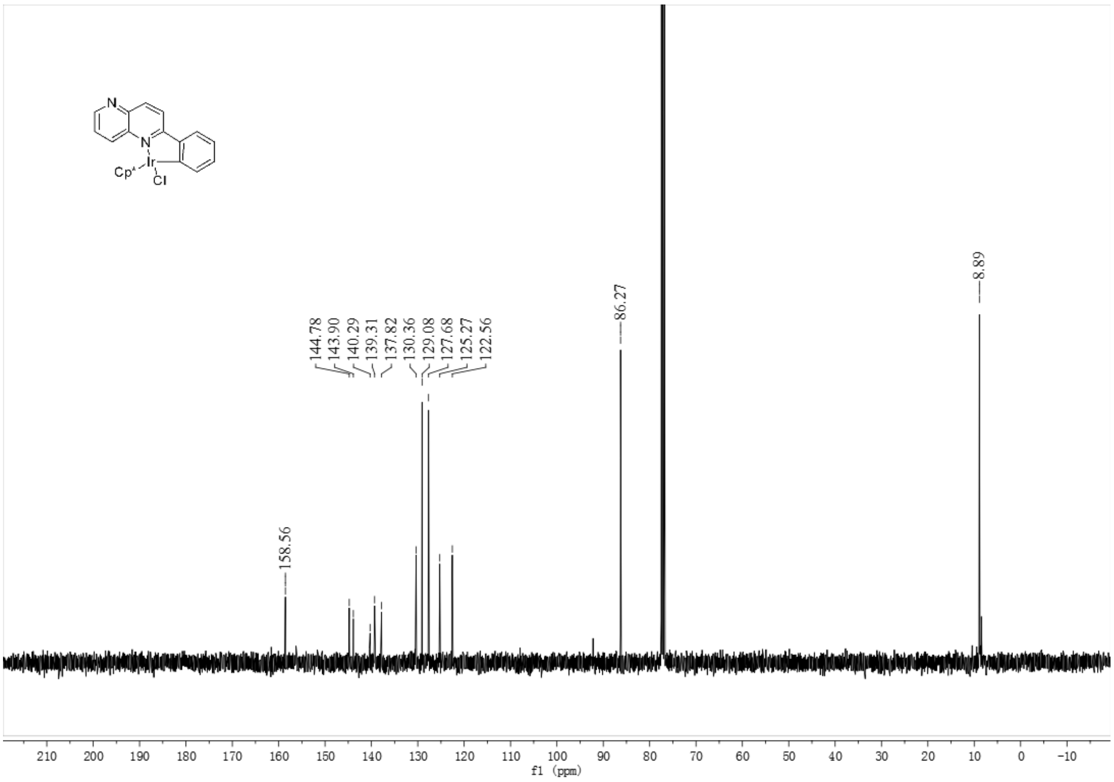

Figure S18. ^{1}H-NMR (400 MHz, $CDCl_3$) spectrum of **Ir-9**, related to **Table 1**.

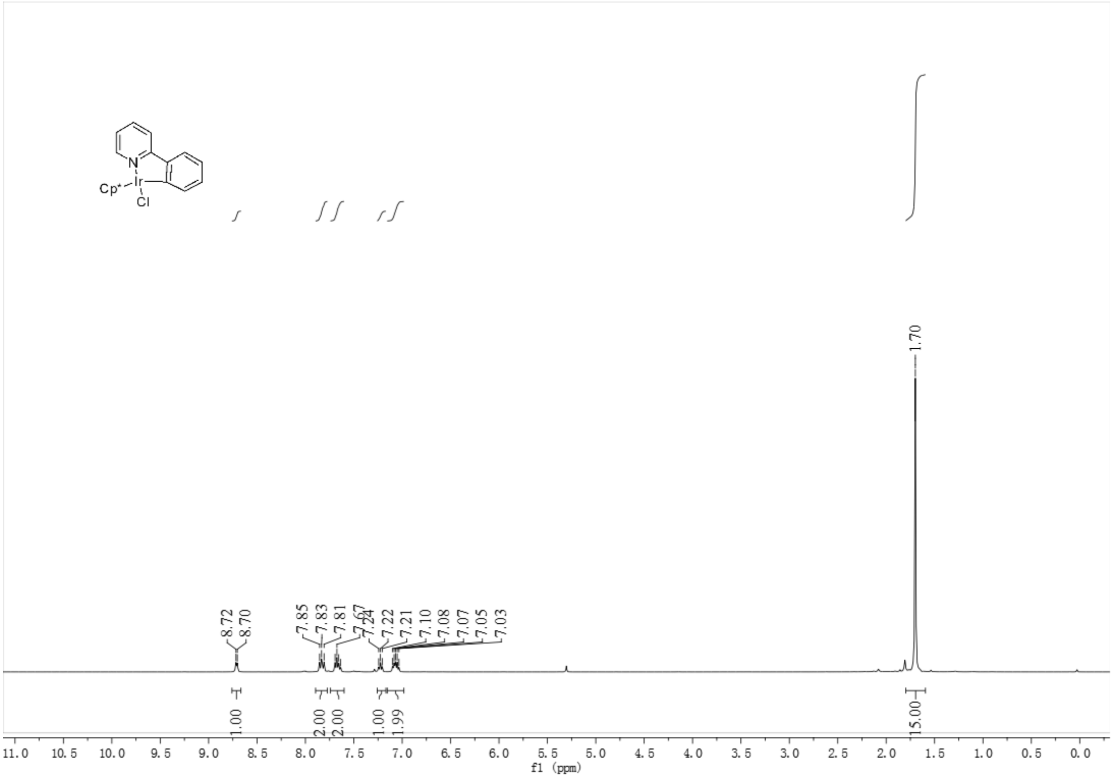

Figure S19. ^{13}C-NMR (100 MHz, $CDCl_3$) spectrum of **Ir-9**, related to **Table 1**.

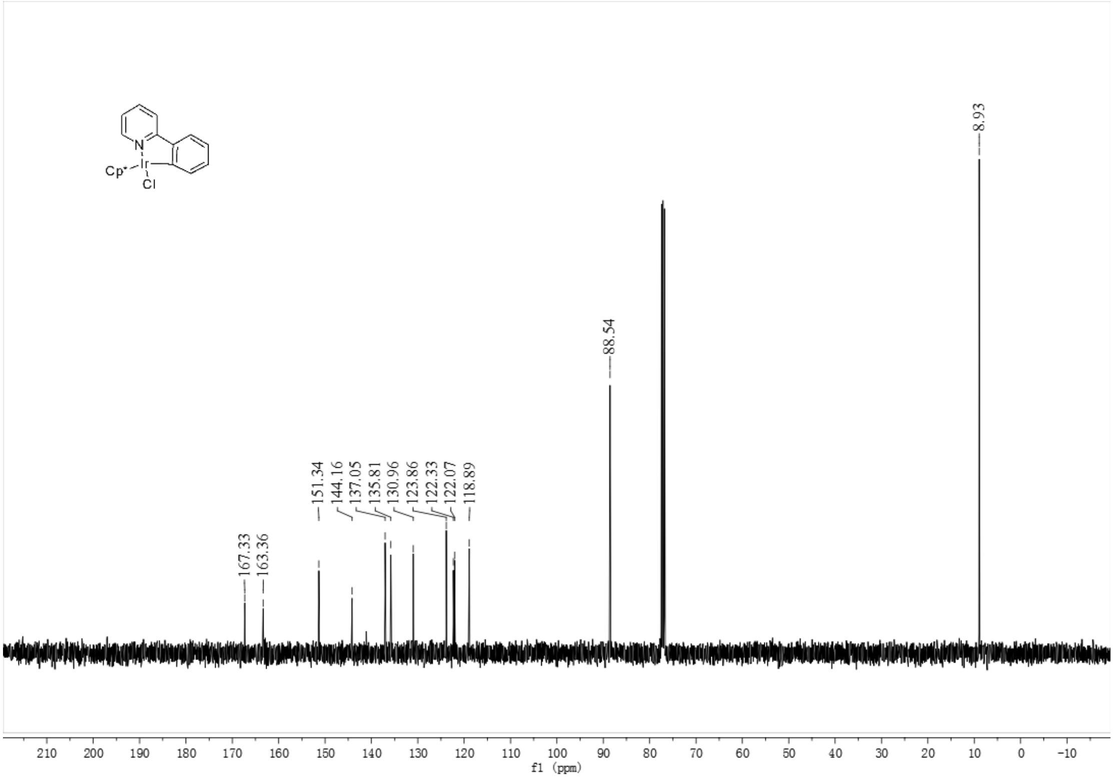

Figure S20. ^{1}H-NMR (400 MHz, $CDCl_3$) spectrum of **3aa**, related to **Scheme 2**.

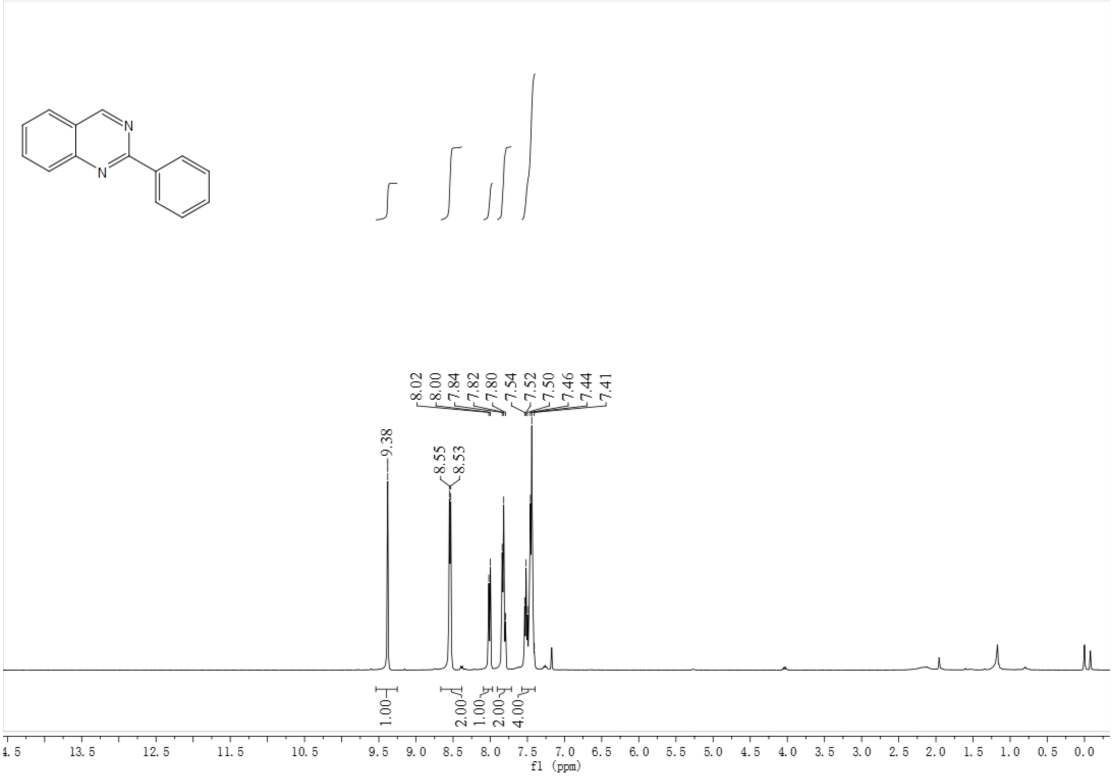

Figure S21. ^{13}C-NMR (100 MHz, $CDCl_3$) spectrum of **3aa**, related to **Scheme 2**.

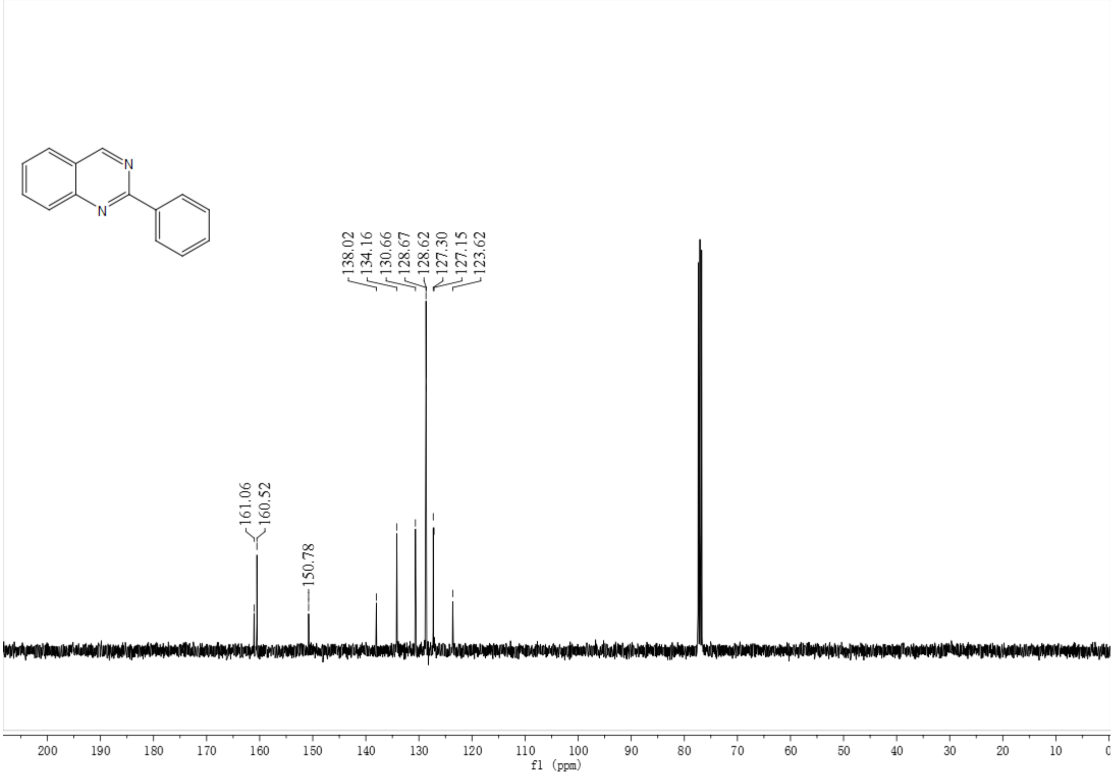

Figure S22. ^{1}H-NMR (400 MHz, $CDCl_3$) spectrum of **3ab**, related to **Scheme 2**.

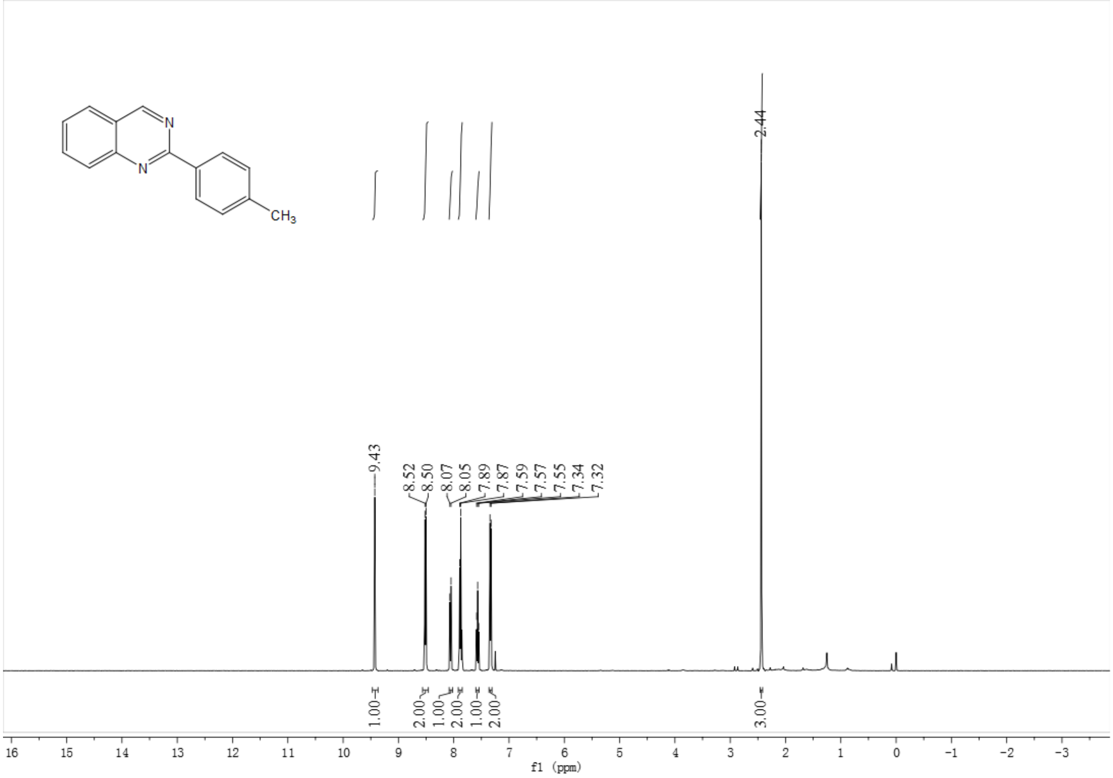

Figure S23. ^{13}C-NMR (100 MHz, $CDCl_3$) spectrum of **3ab**, related to **Scheme 2**.

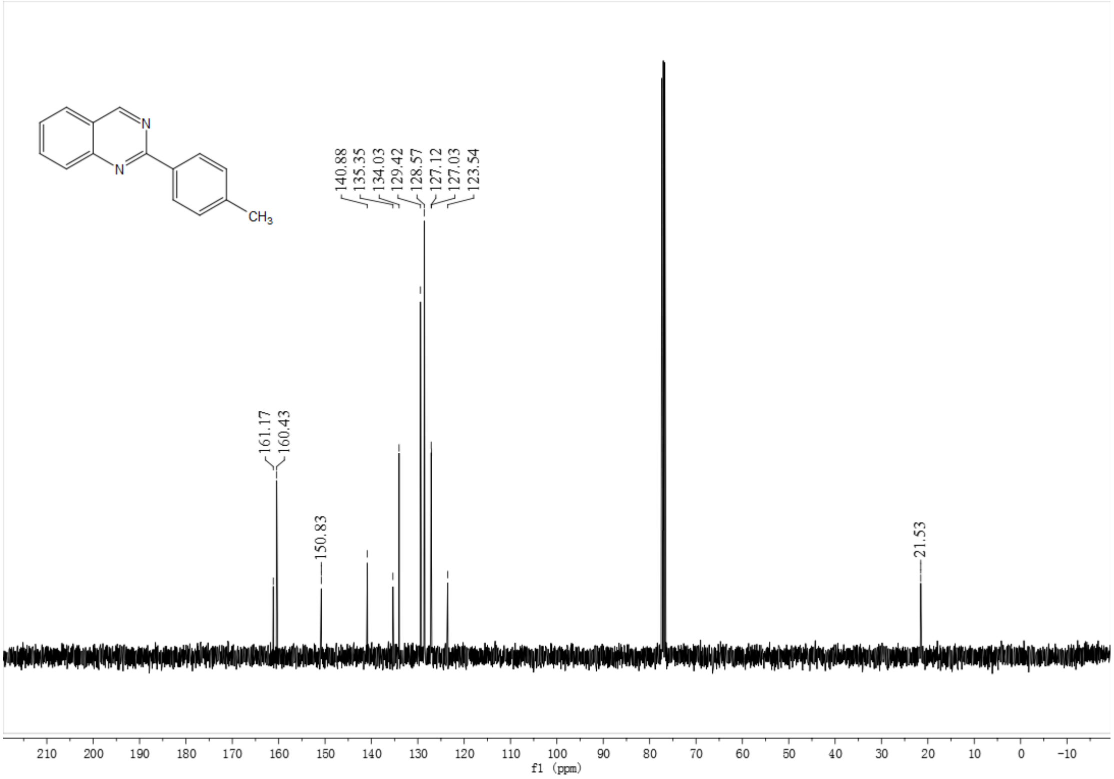

Figure S24. ^{1}H-NMR (400 MHz, $CDCl_3$) spectrum of **3ac**, related to **Scheme 2**.

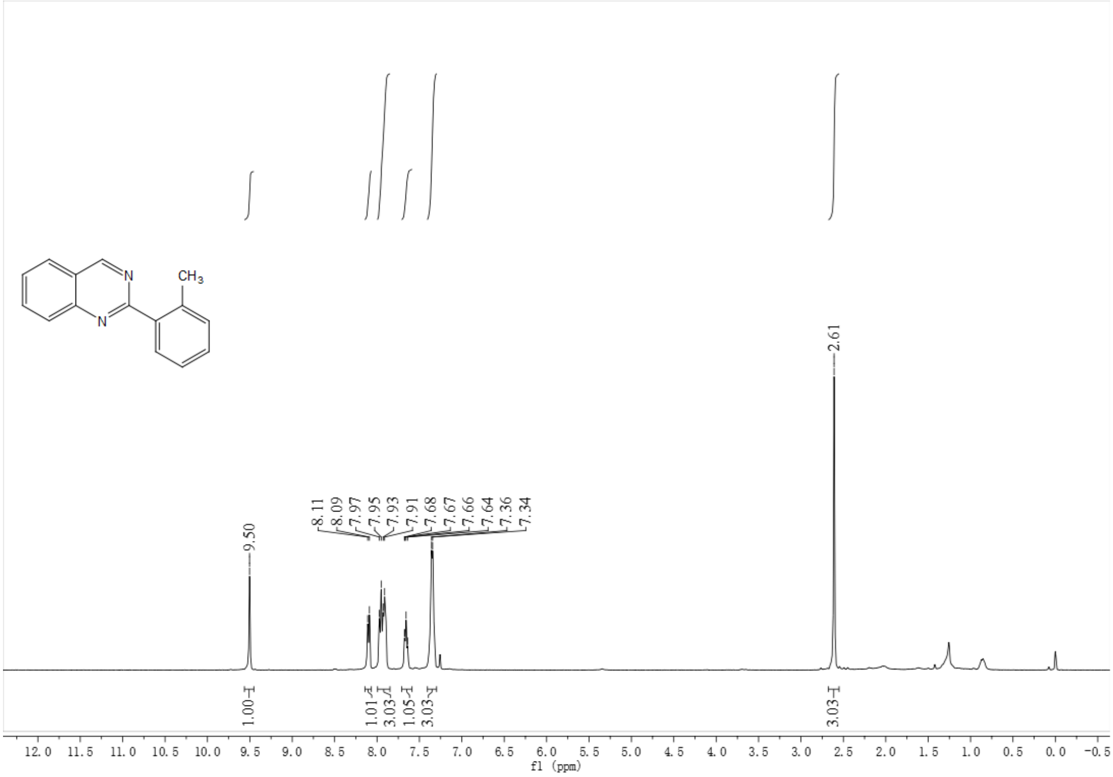

Figure S25. ^{13}C-NMR (100 MHz, $CDCl_3$) spectrum of **3ac**, related to **Scheme 2**.

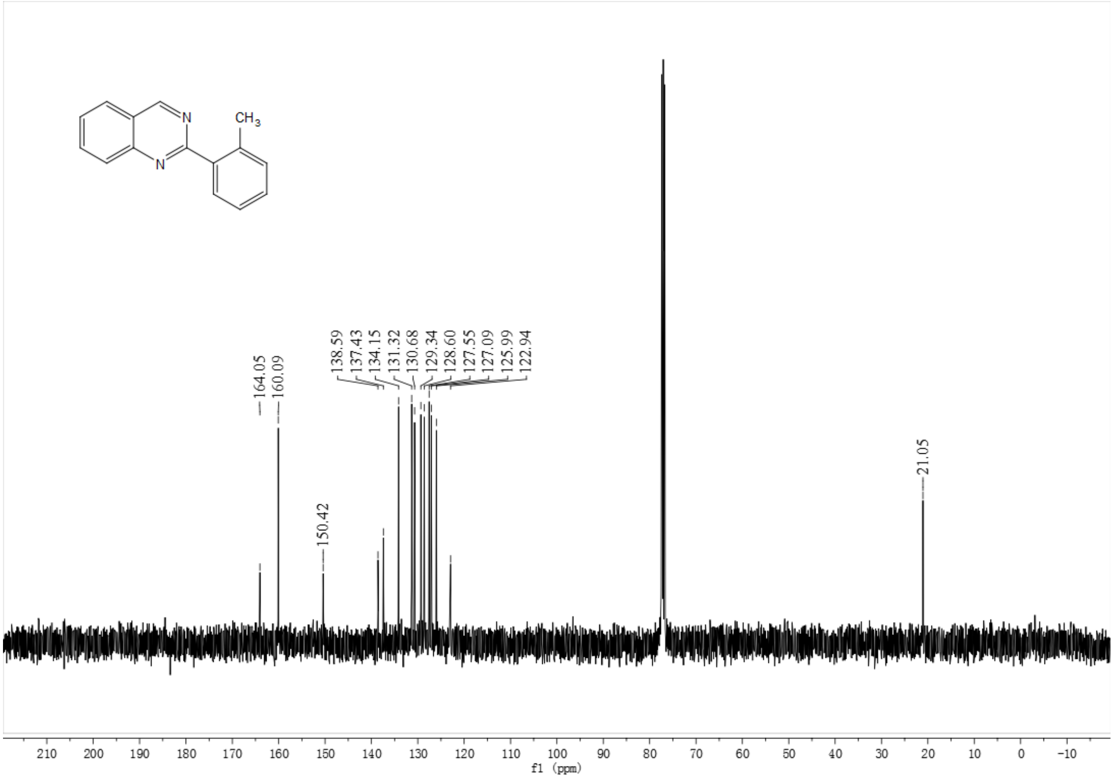

Figure S26. ^{1}H-NMR (400 MHz, $CDCl_3$) spectrum of **3ad**, related to **Scheme 2**.

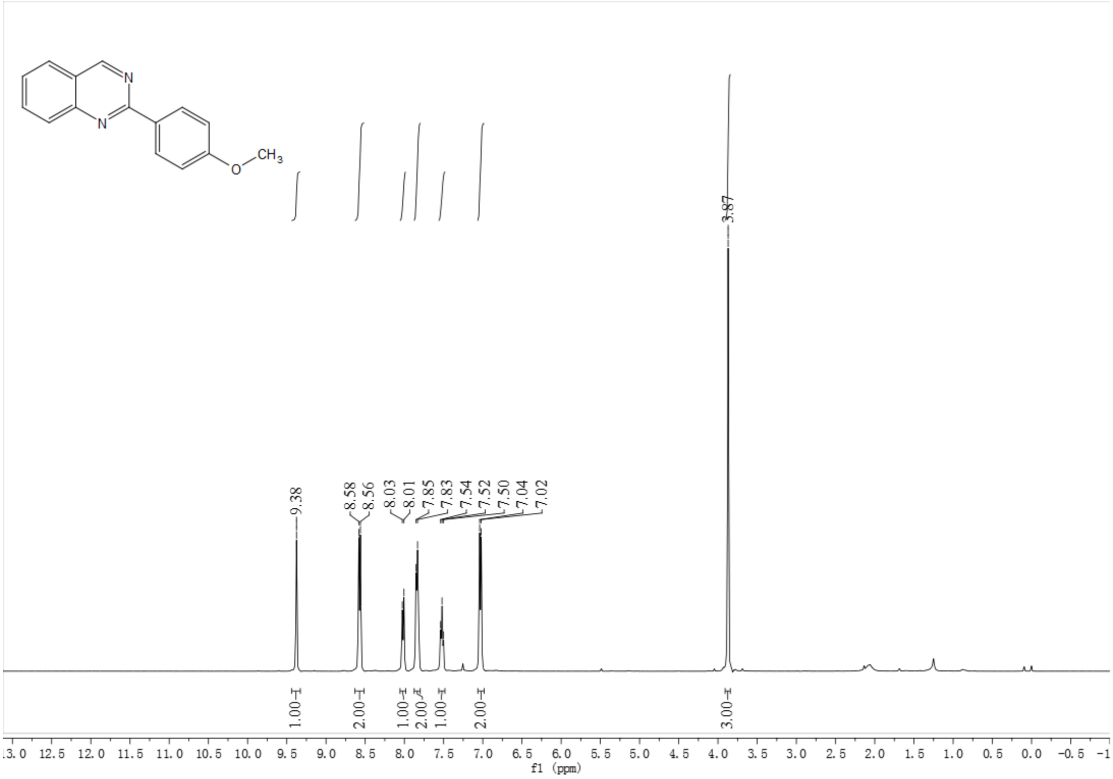

Figure S27. ^{13}C-NMR (100 MHz, $CDCl_3$) spectrum of **3ad**, related to **Scheme 2**.

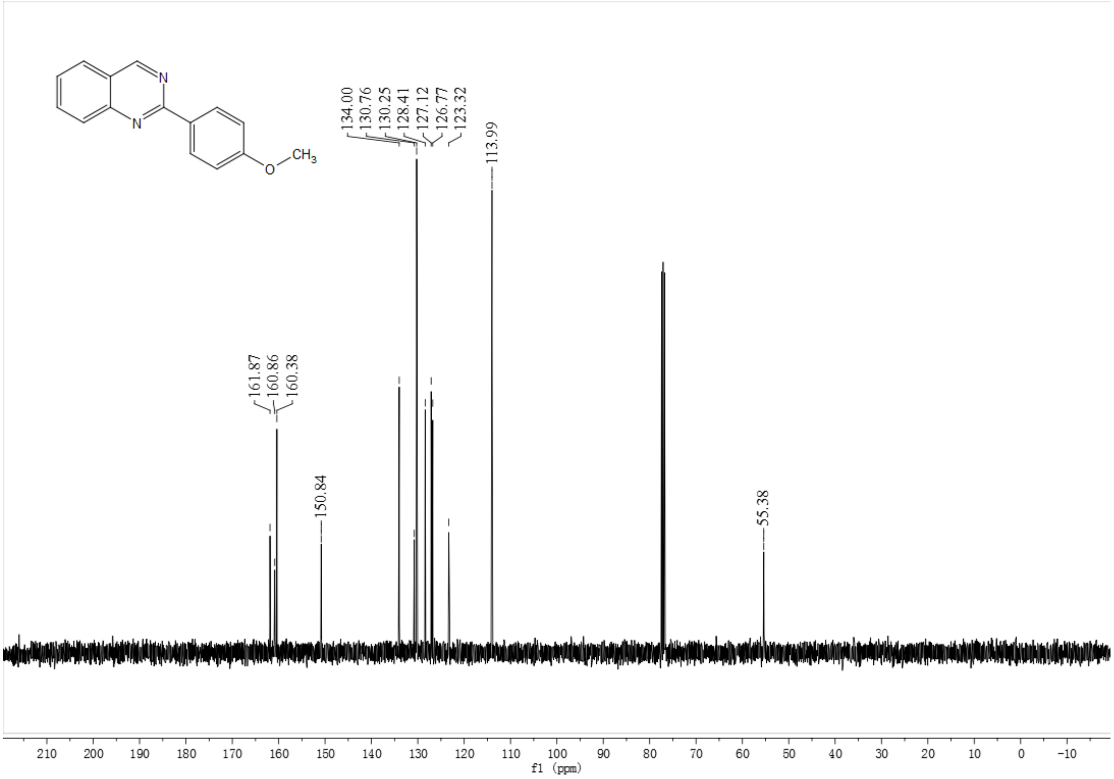

Figure S28. ^{1}H-NMR (400 MHz, $CDCl_3$) spectrum of **3ae**, related to **Scheme 2**.

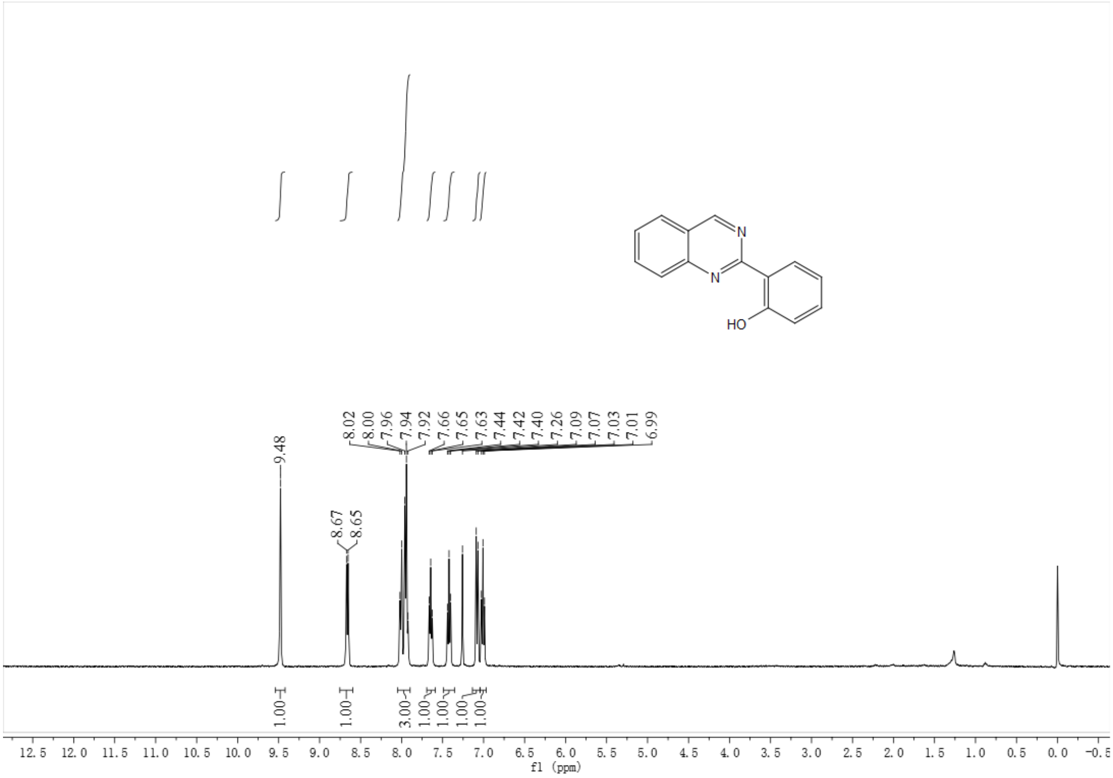

Figure S29. ^{13}C-NMR (100 MHz, $CDCl_3$) spectrum of **3ae**, related to **Scheme 2**.

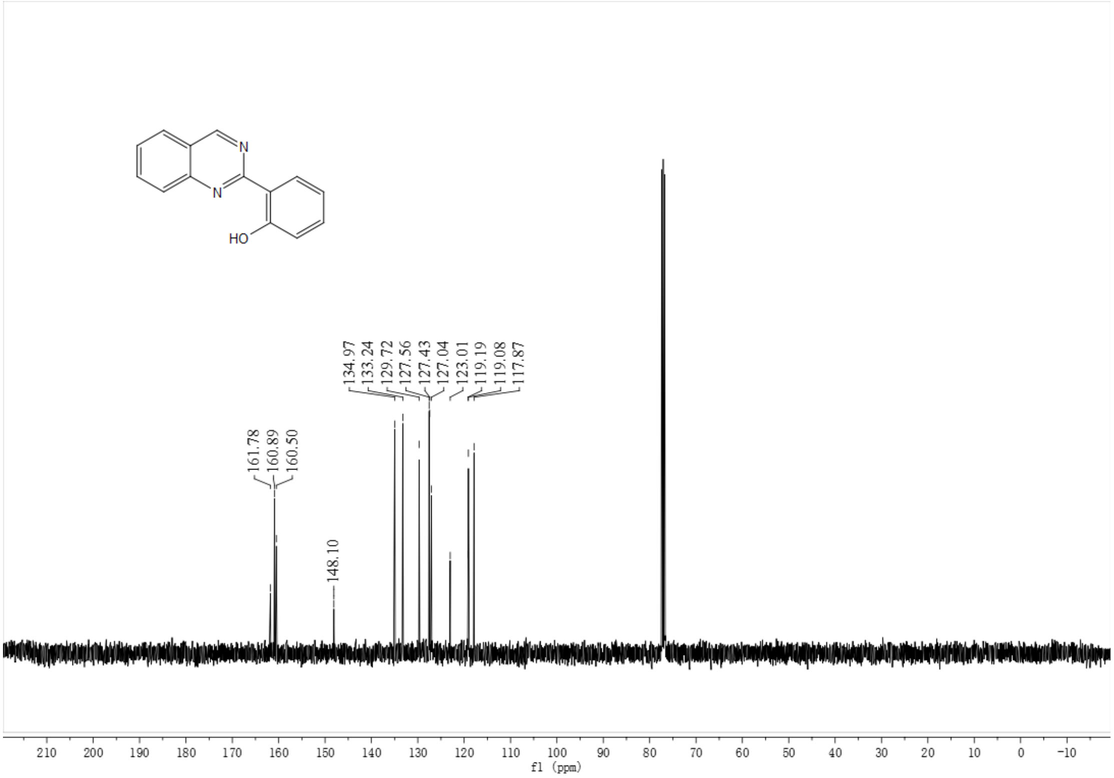

Figure S30. ^{1}H-NMR (400 MHz, $CDCl_3$) spectrum of **3af**, related to **Scheme 2**.

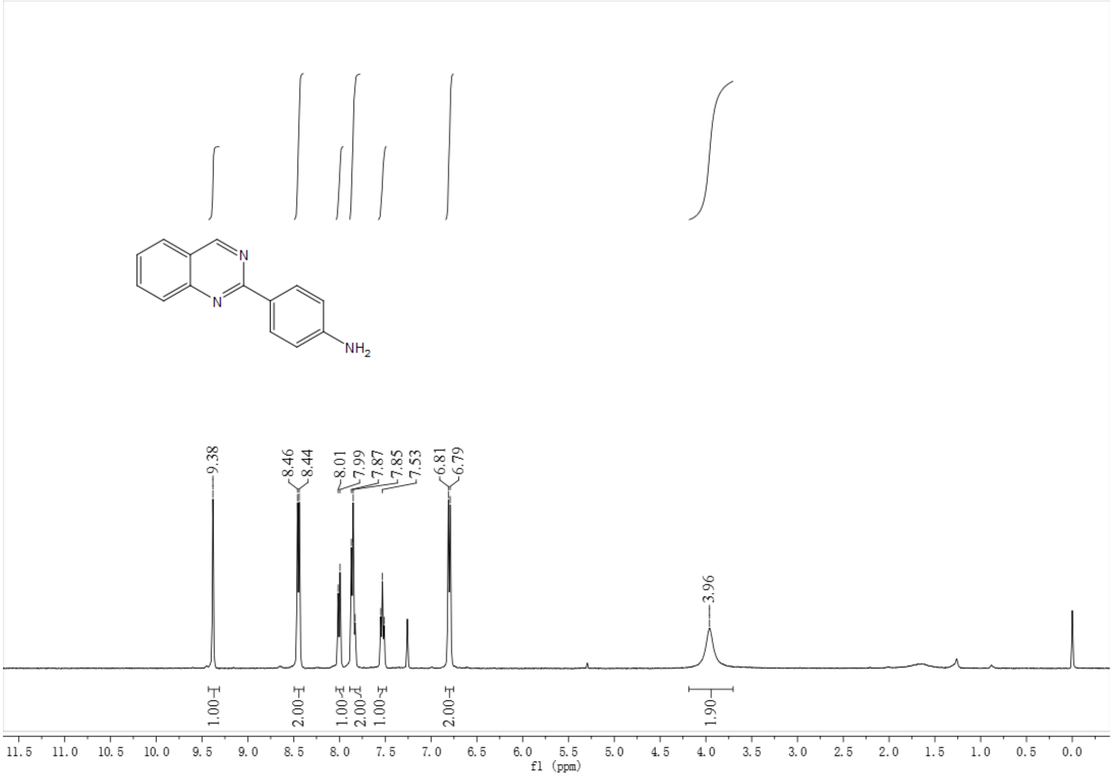

Figure S31. ^{13}C-NMR (100 MHz, $CDCl_3$) spectrum of **3af**, related to **Scheme 2**.

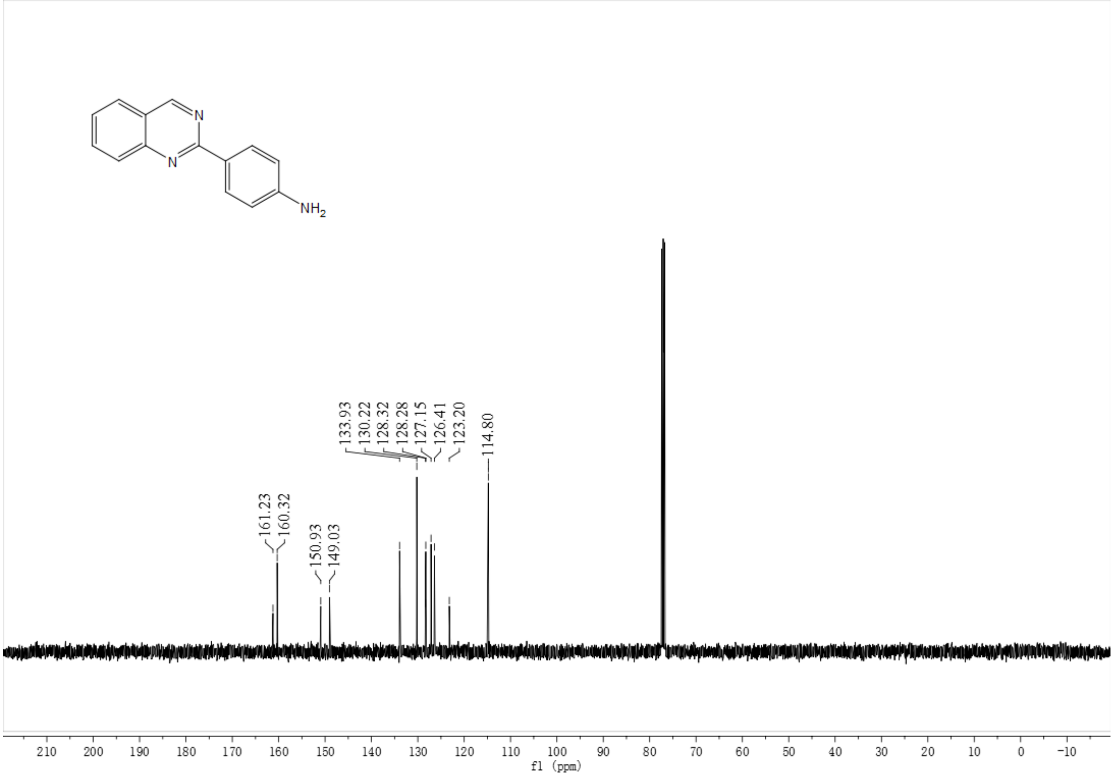

Figure S32. ^{1}H-NMR (400 MHz, $CDCl_3$) spectrum of **3ag**, related to **Scheme 2**.

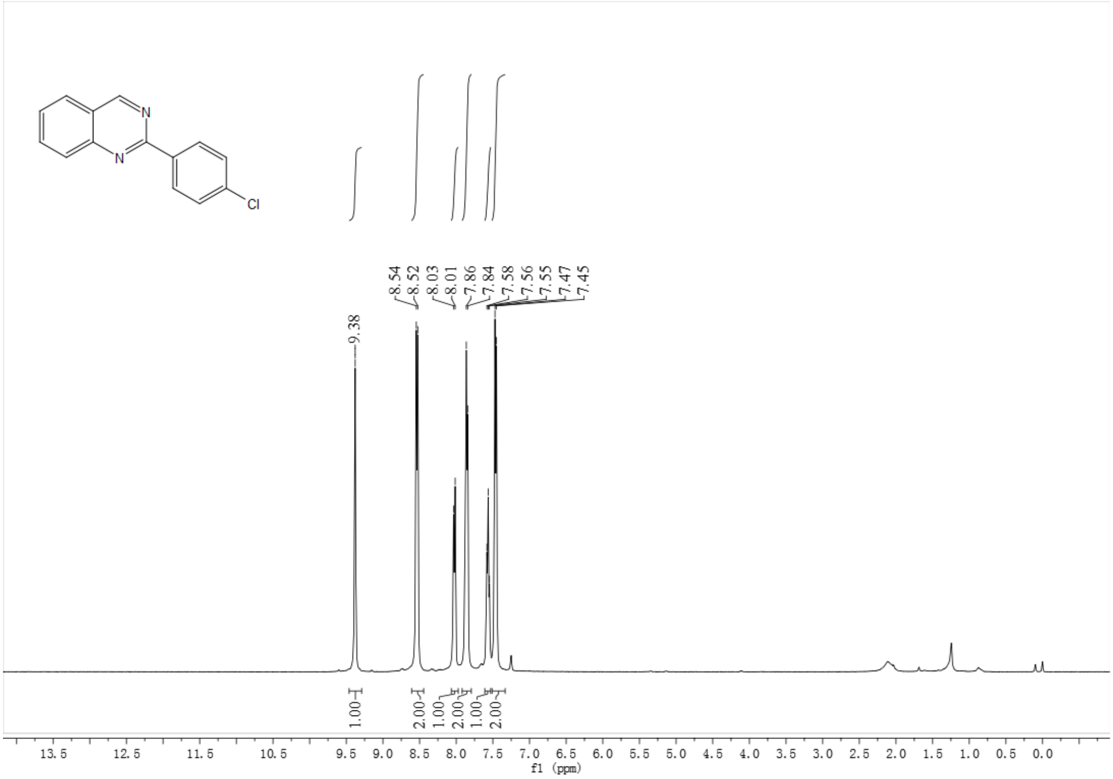

Figure S33. ^{13}C-NMR (100 MHz, $CDCl_3$) spectrum of **3ag**, related to **Scheme 2**.

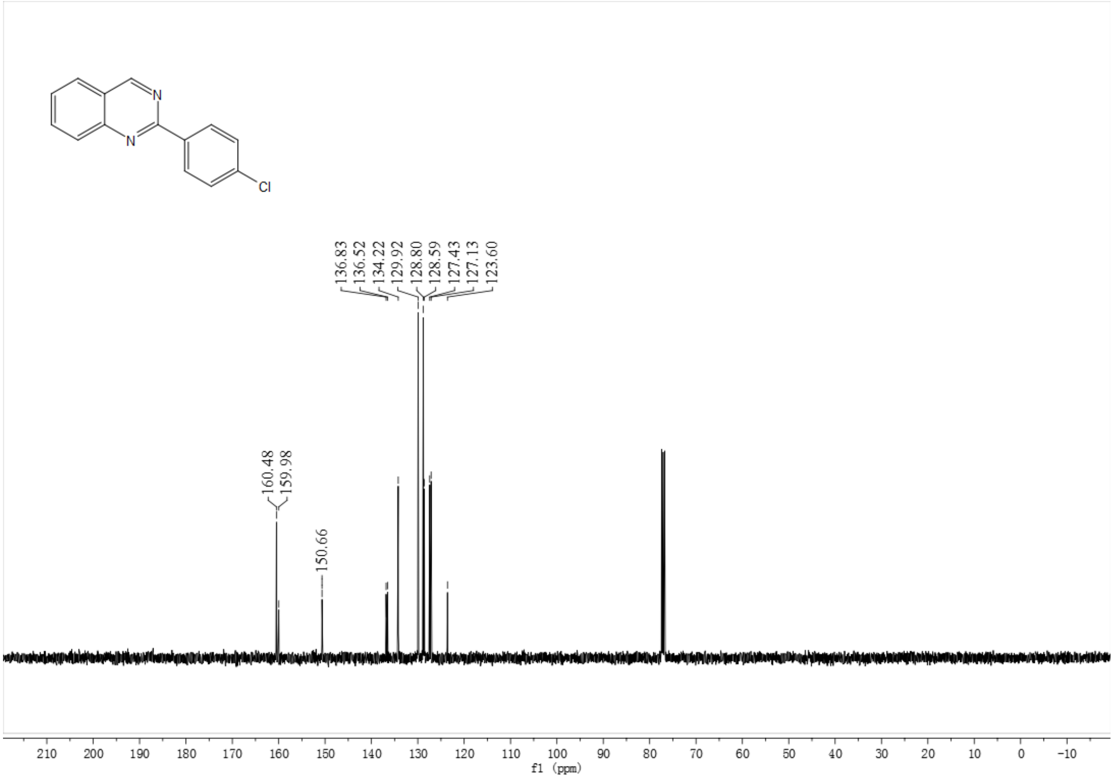

Figure S34. ^{1}H-NMR (400 MHz, $CDCl_3$) spectrum of **3ah**, related to **Scheme 2**.

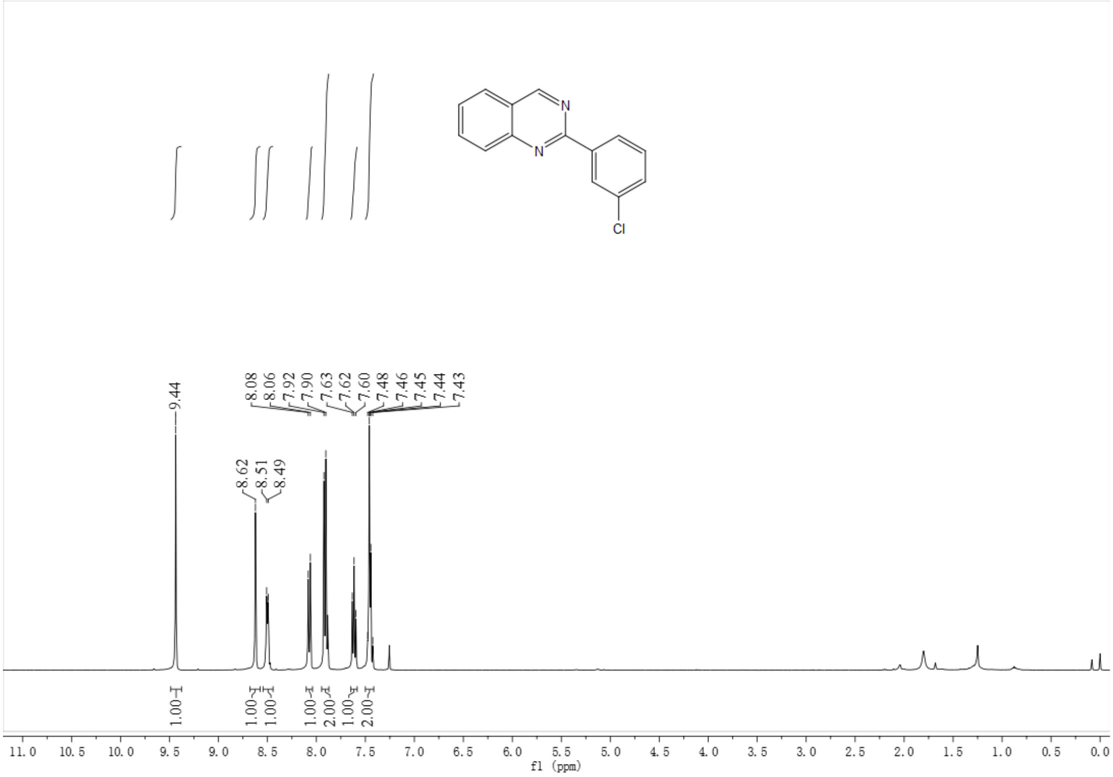

Figure S35. ^{13}C-NMR (100 MHz, $CDCl_3$) spectrum of **3ah**, related to **Scheme 2**.

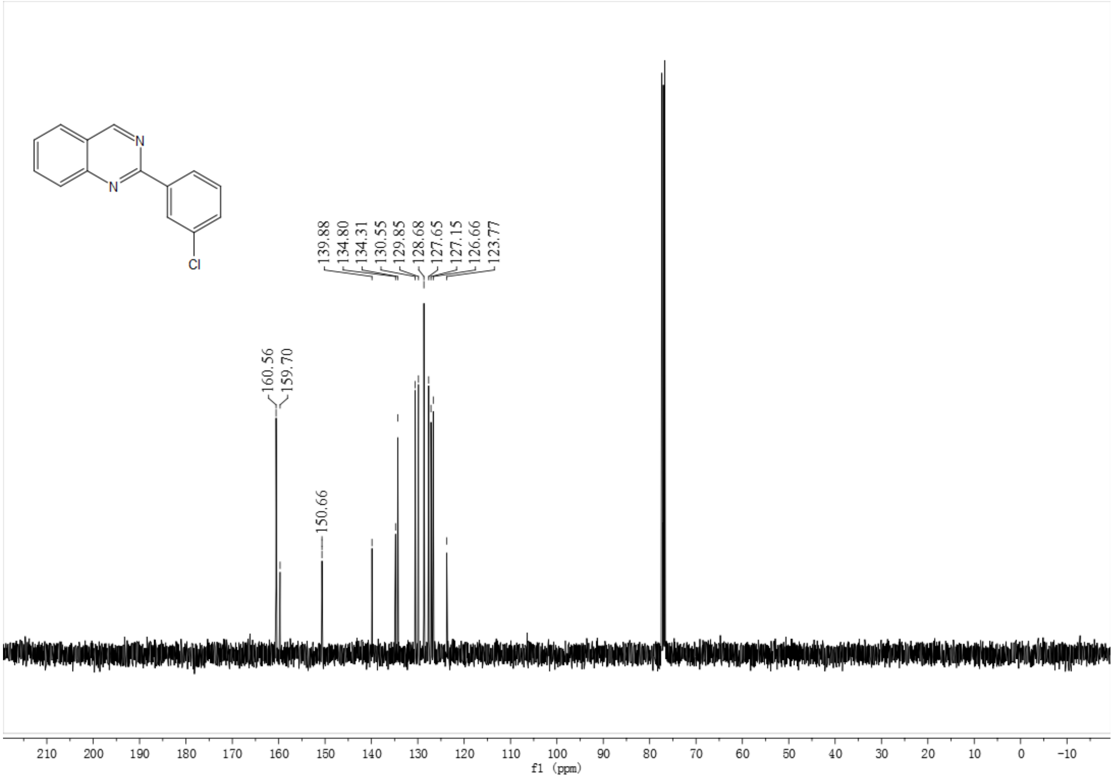

Figure S36. ^{1}H-NMR (400 MHz, $CDCl_3$) spectrum of **3ai**, related to **Scheme 2**.

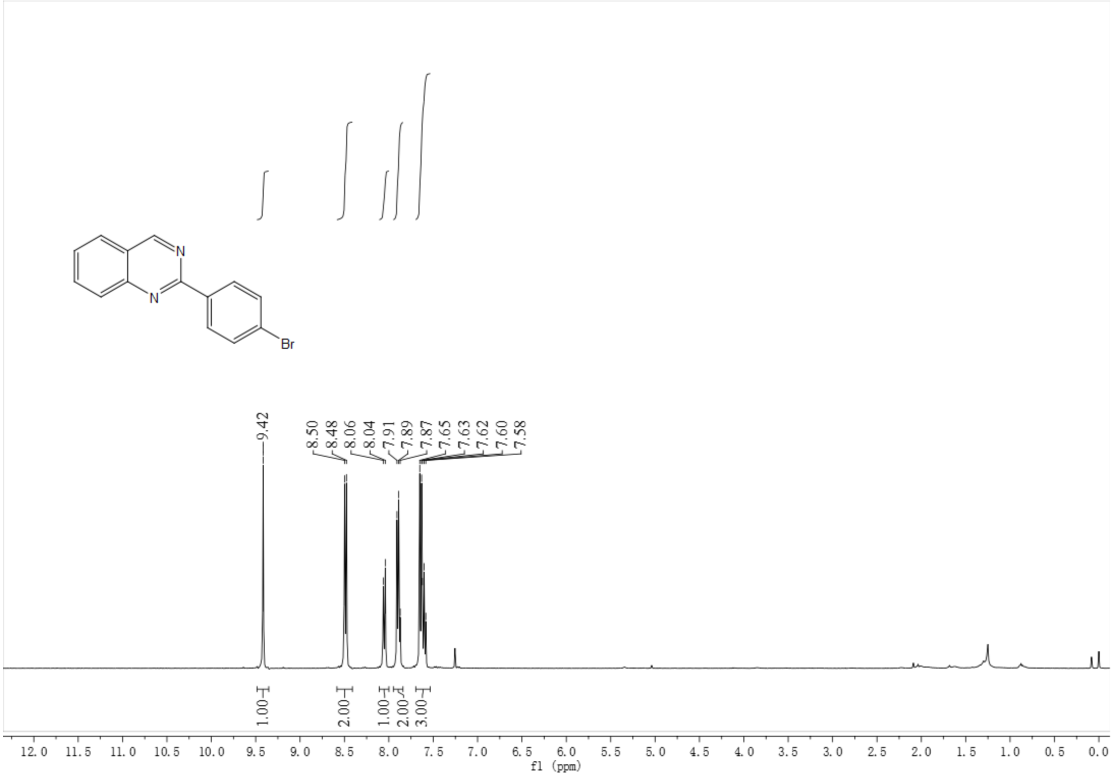

Figure S37. ^{13}C-NMR (100 MHz, $CDCl_3$) spectrum of **3ai**, related to **Scheme 2**.

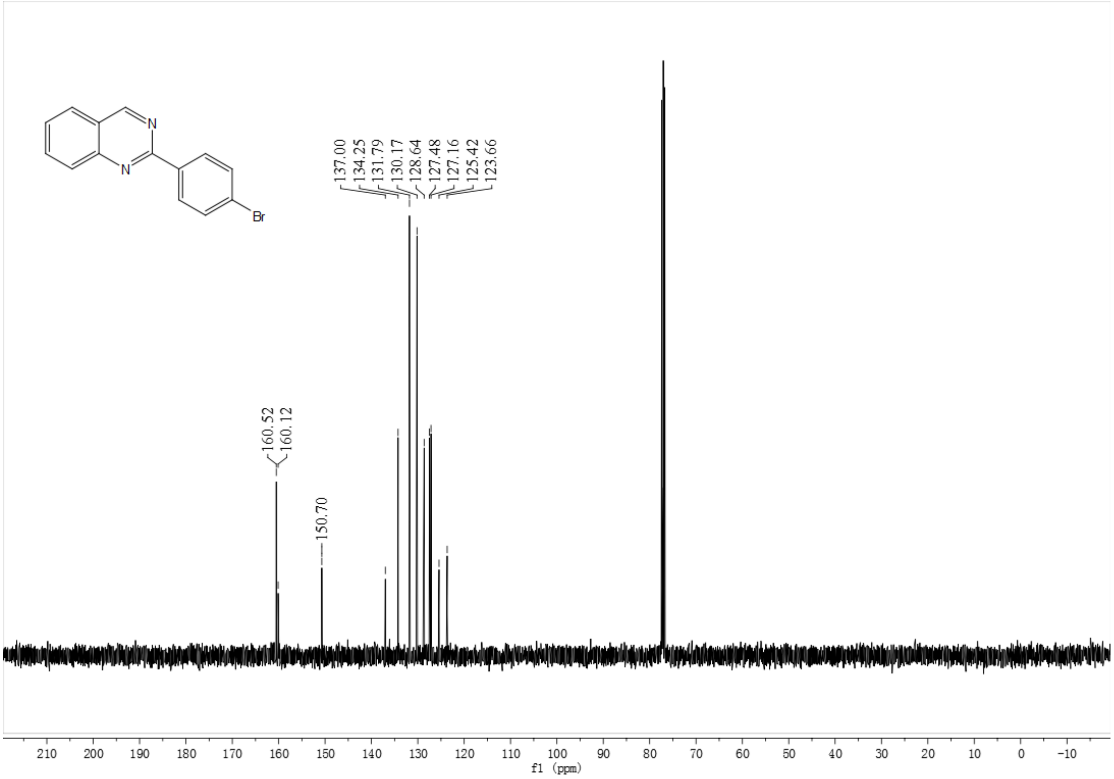

Figure S38. ^{1}H-NMR (400 MHz, $CDCl_3$) spectrum of **3aj**, related to **Scheme 2**.

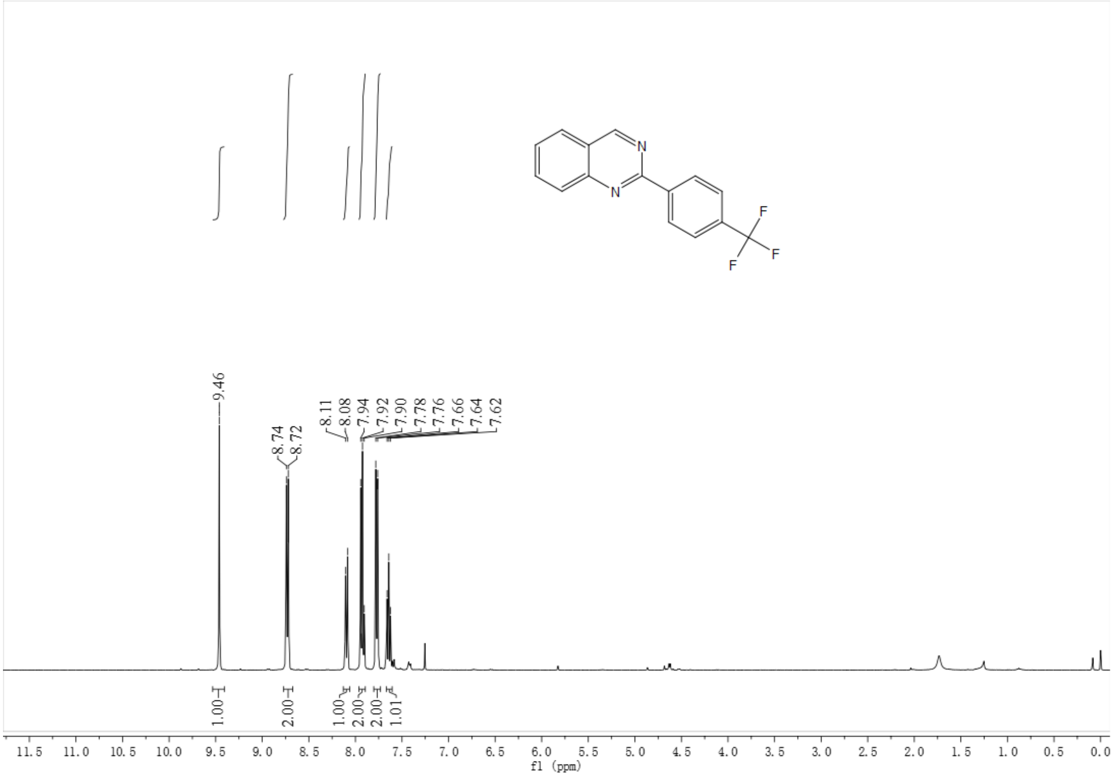

Figure S39. ^{13}C-NMR (100 MHz, $CDCl_3$) spectrum of **3aj**, related to **Scheme 2**.

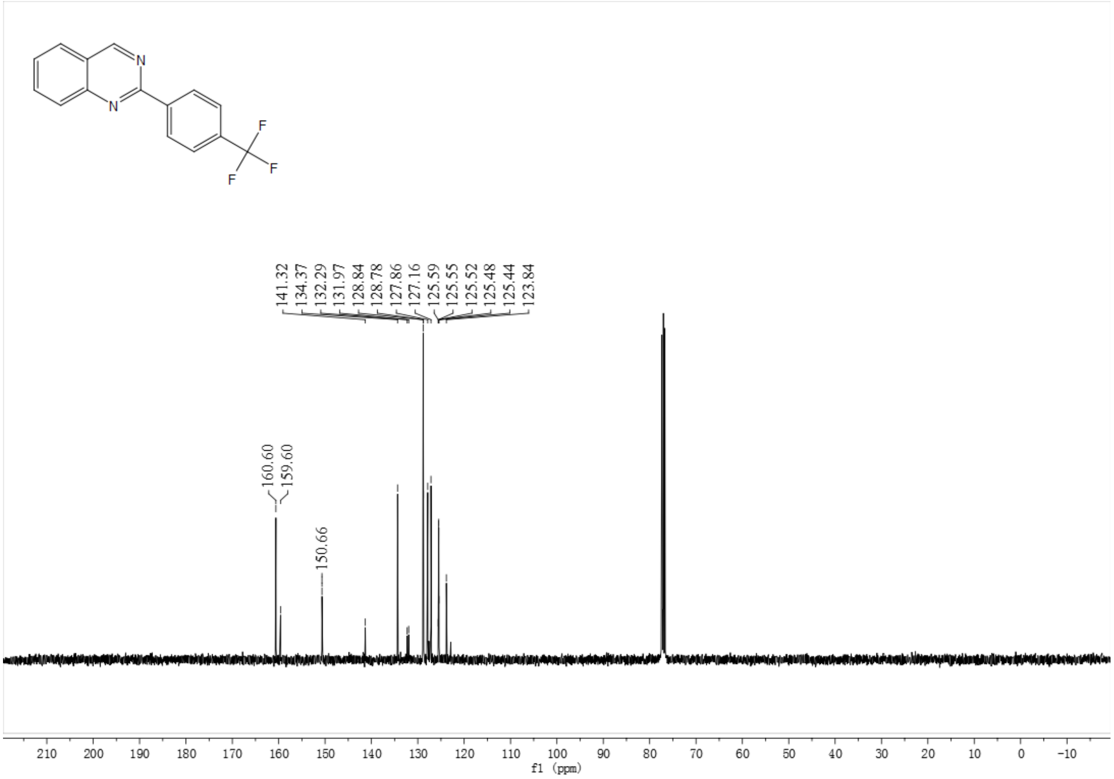

Figure S40. ^{19}F-NMR (100 MHz, $CDCl_3$) spectrum of **3aj**, related to **Scheme 2**.

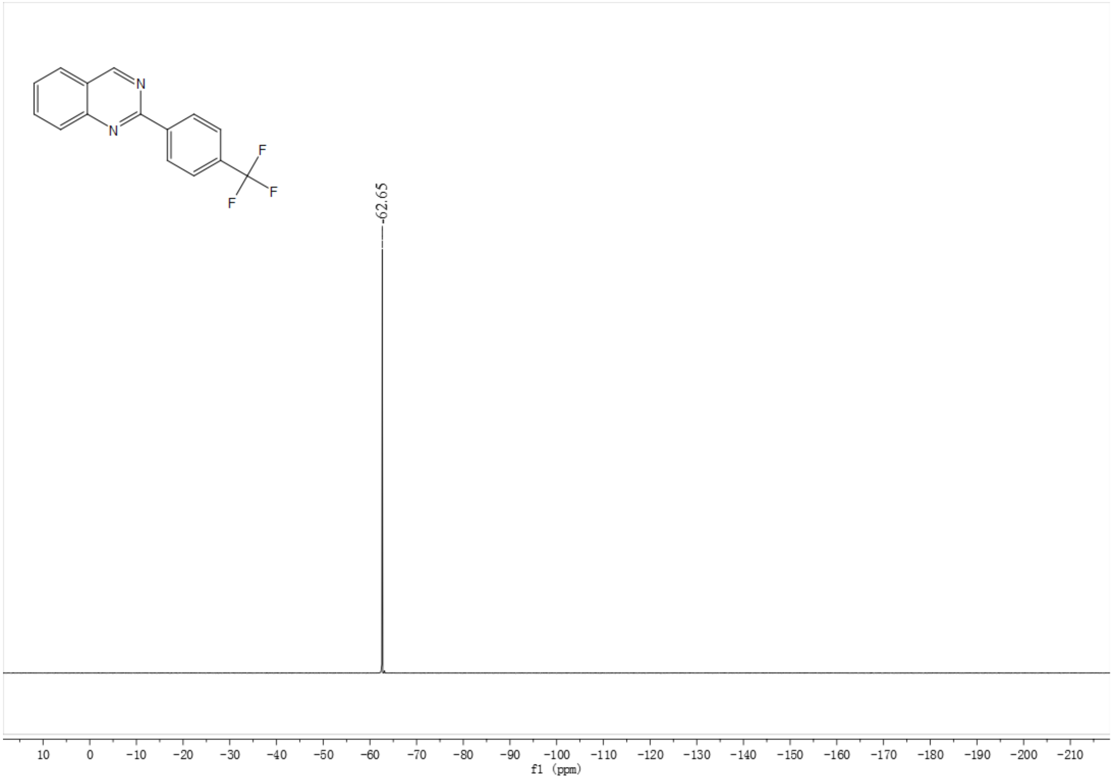

Figure S41. ^{1}H-NMR (400 MHz, $CDCl_3$) spectrum of **3ak**, related to **Scheme 2**.

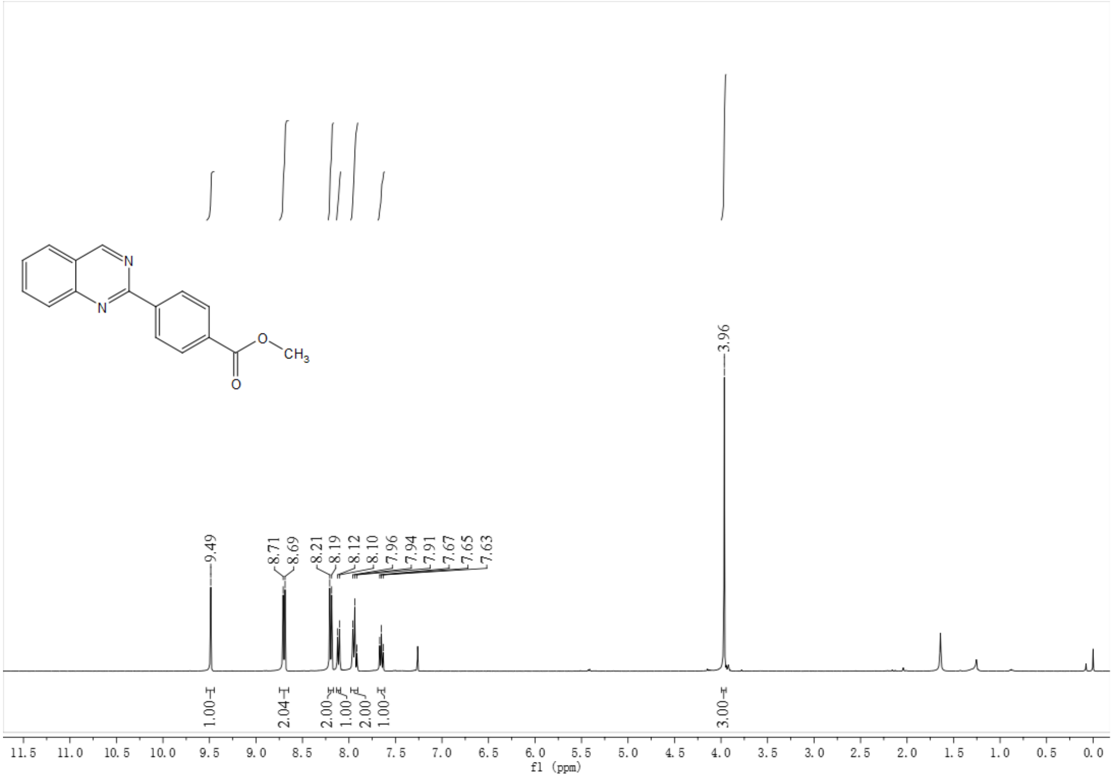

Figure S42. ^{13}C-NMR (100 MHz, $CDCl_3$) spectrum of **3ak**, related to **Scheme 2**.

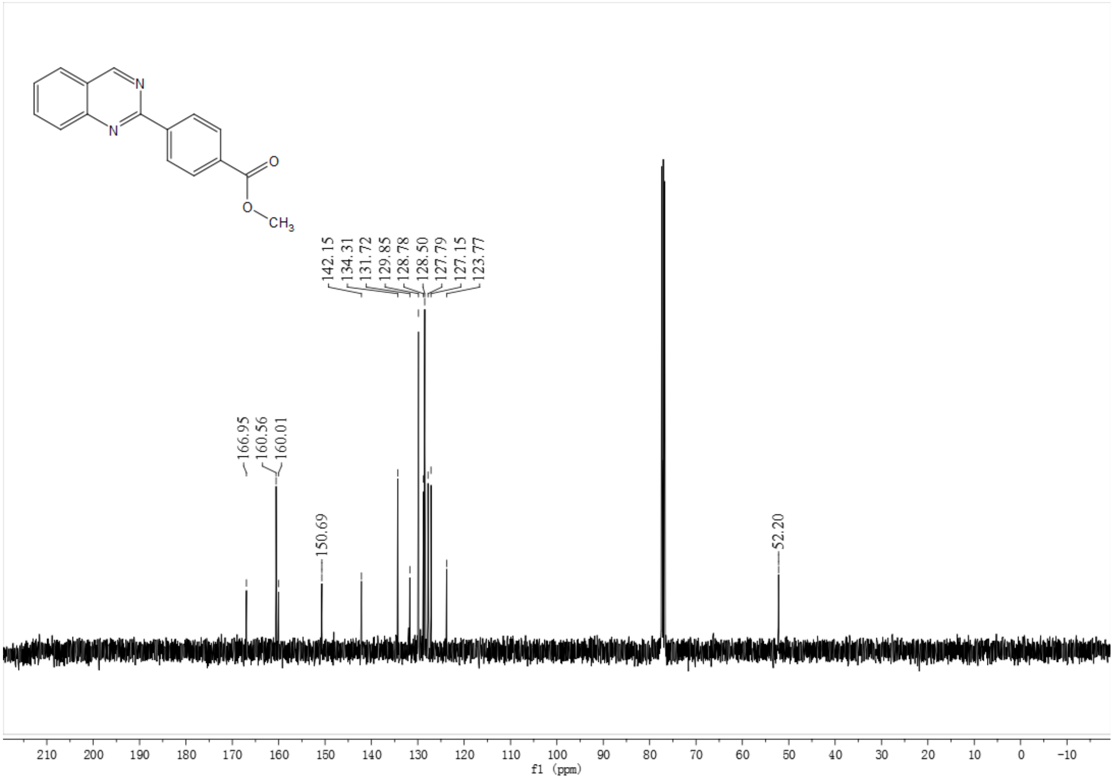

Figure S43. ^{1}H-NMR (400 MHz, $CDCl_3$) spectrum of **3al**, related to **Scheme 2**.

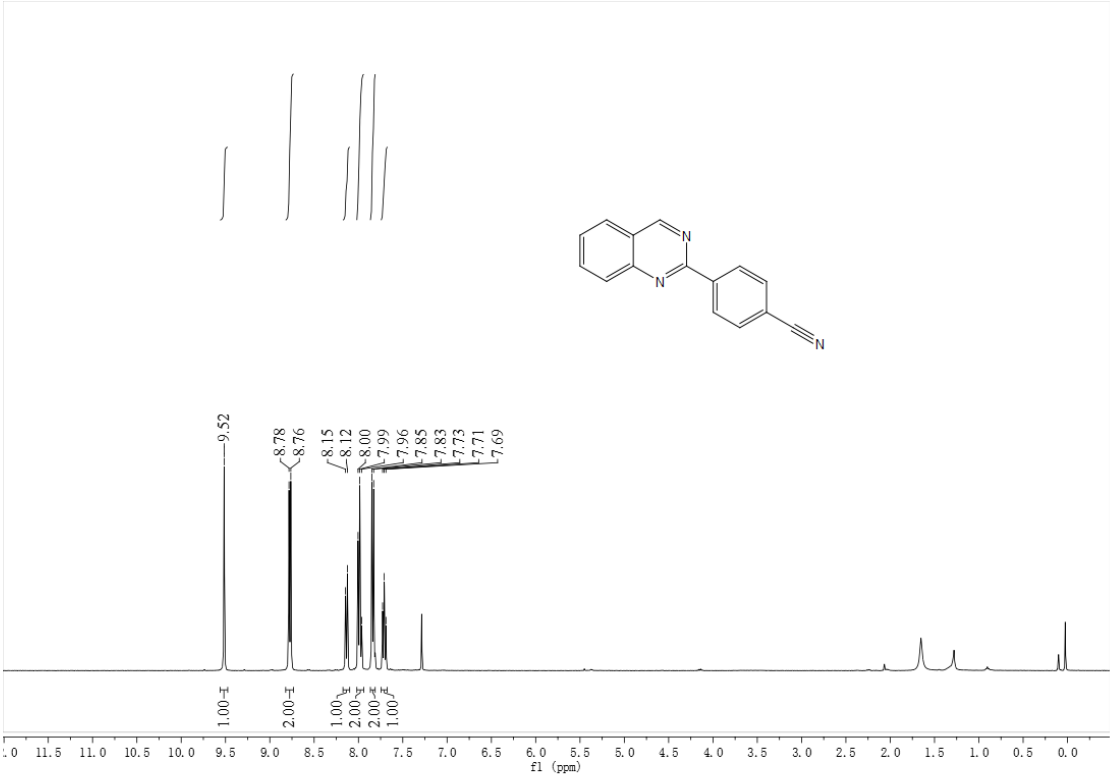

Figure S44. ^{13}C-NMR (100 MHz, $CDCl_3$) spectrum of **3al**, related to **Scheme 2**.

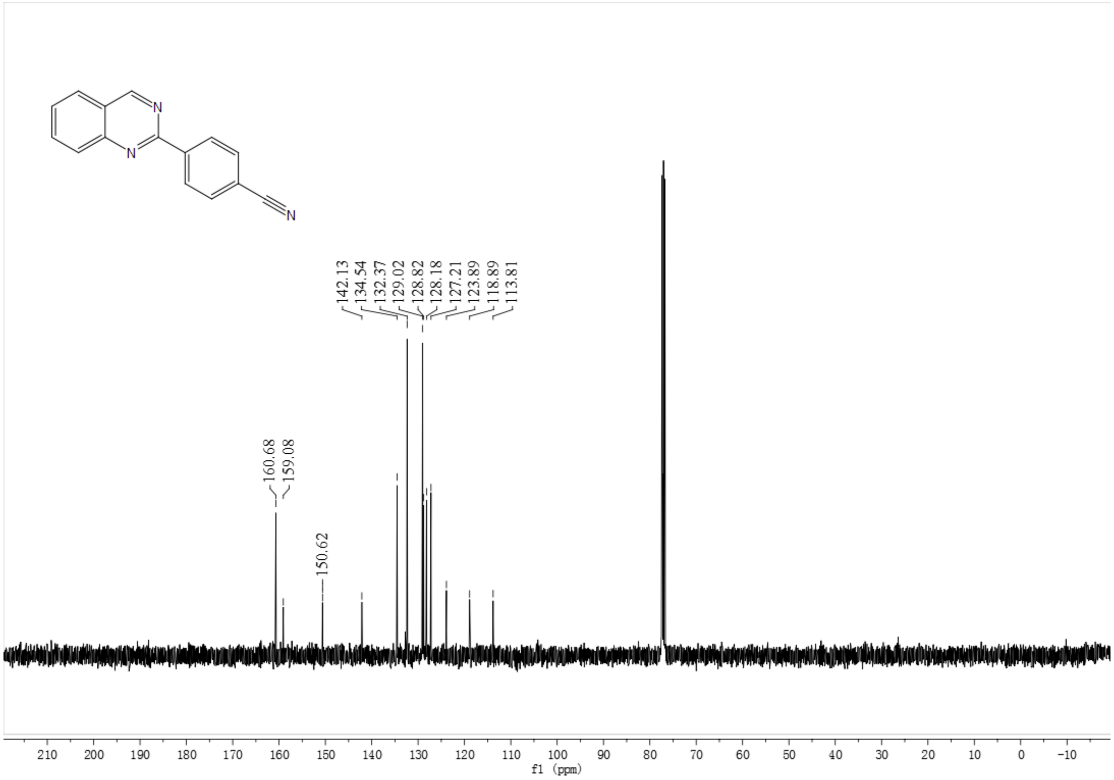

Figure S45. ^{1}H-NMR (400 MHz, $CDCl_3$) spectrum of **3am**, related to **Scheme 2**.

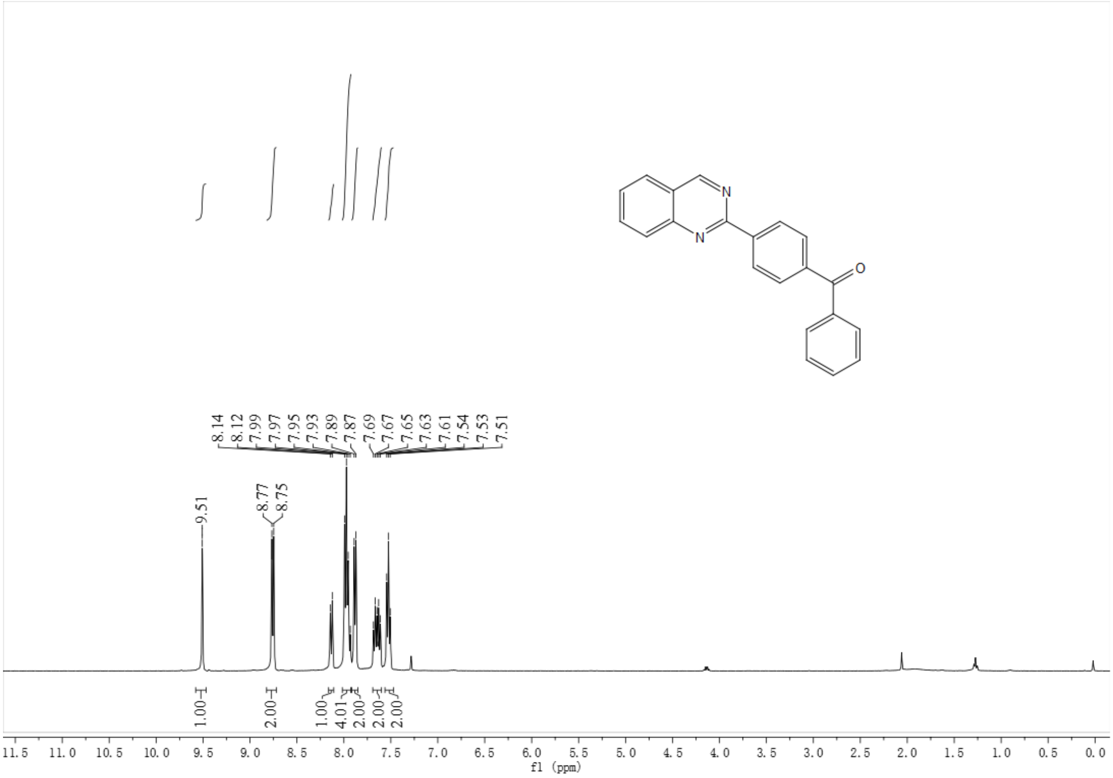

Figure S46. ^{13}C-NMR (100 MHz, $CDCl_3$) spectrum of **3am**, related to **Scheme 2**.

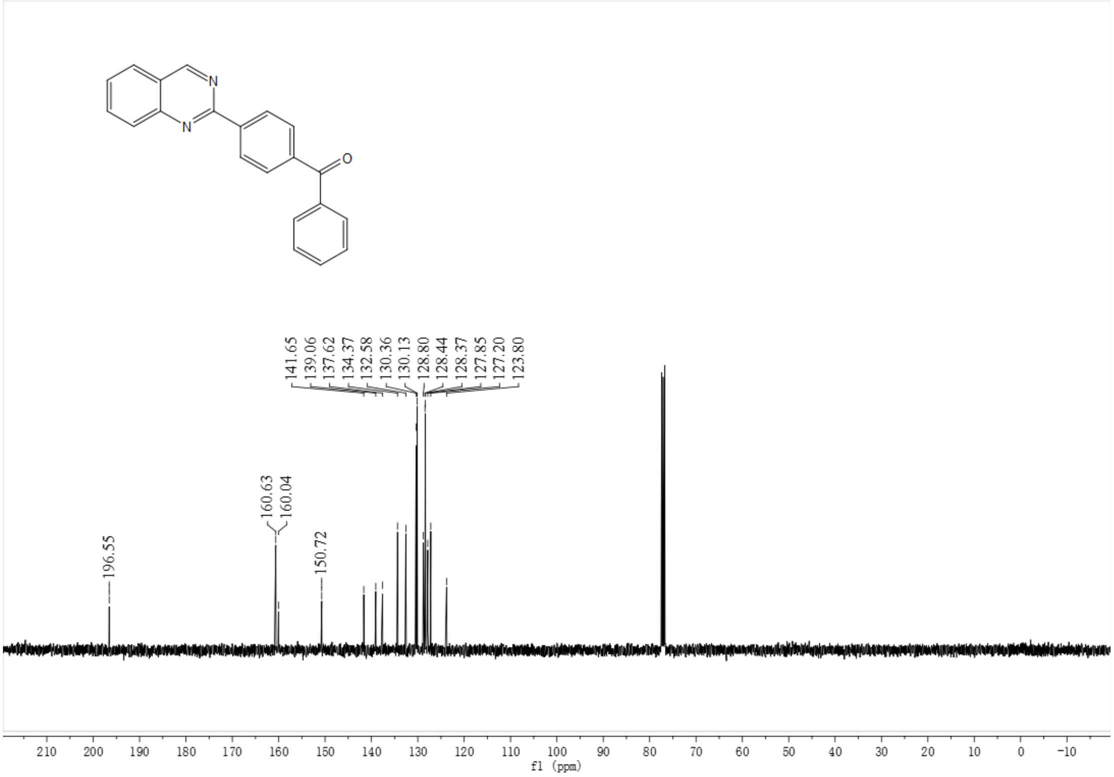

Figure S47. ^{1}H-NMR (400 MHz, $CDCl_3$) spectrum of **3an**, related to **Scheme 2**.

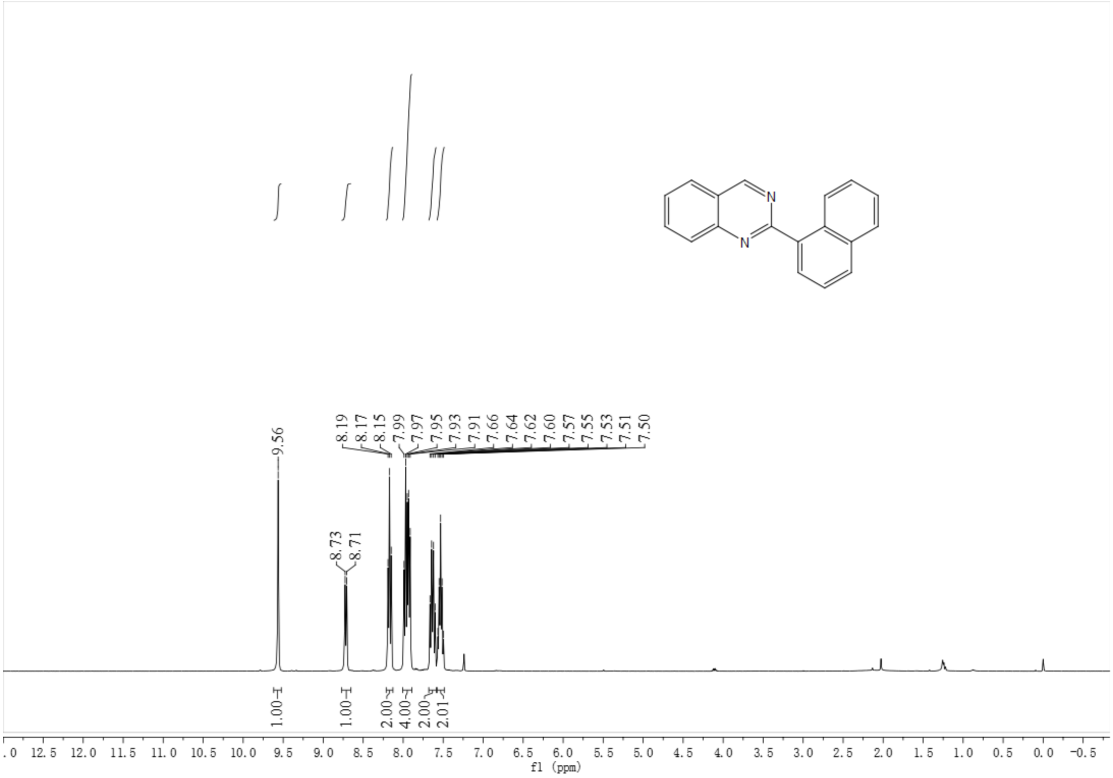

Figure S48. ^{13}C-NMR (100 MHz, $CDCl_3$) spectrum of **3an**, related to **Scheme 2**.

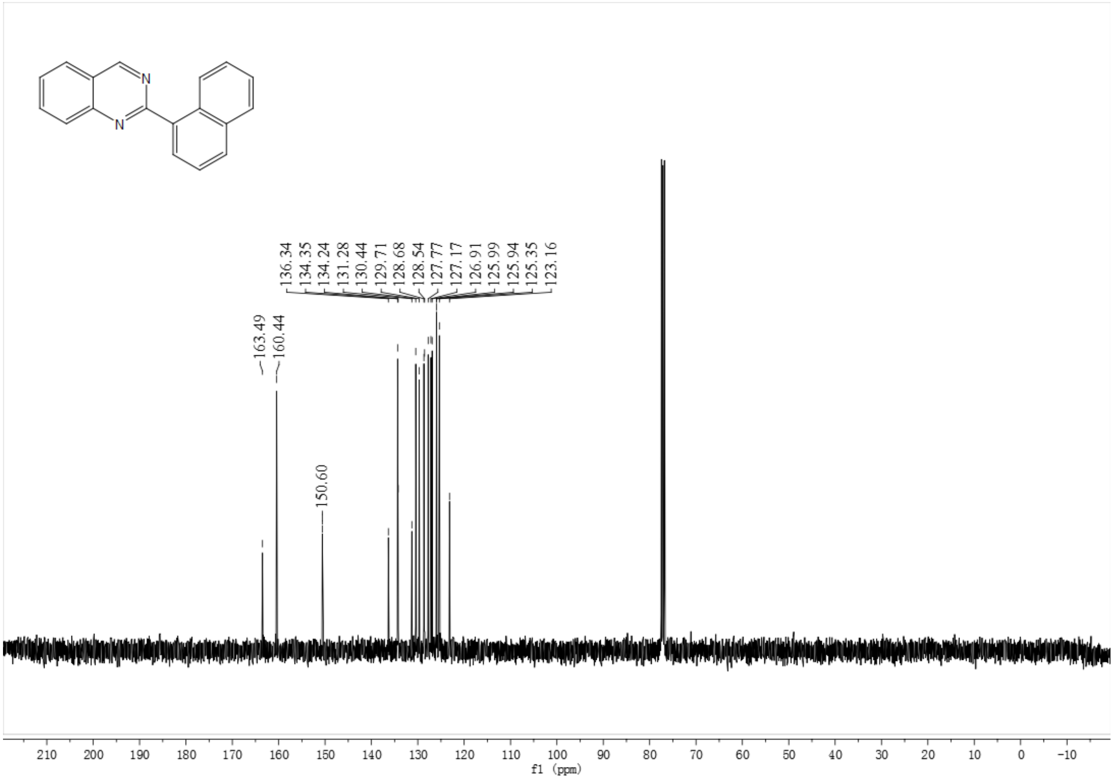

Figure S49. ^{1}H-NMR (400 MHz, $CDCl_3$) spectrum of **3ao**, related to **Scheme 2**.

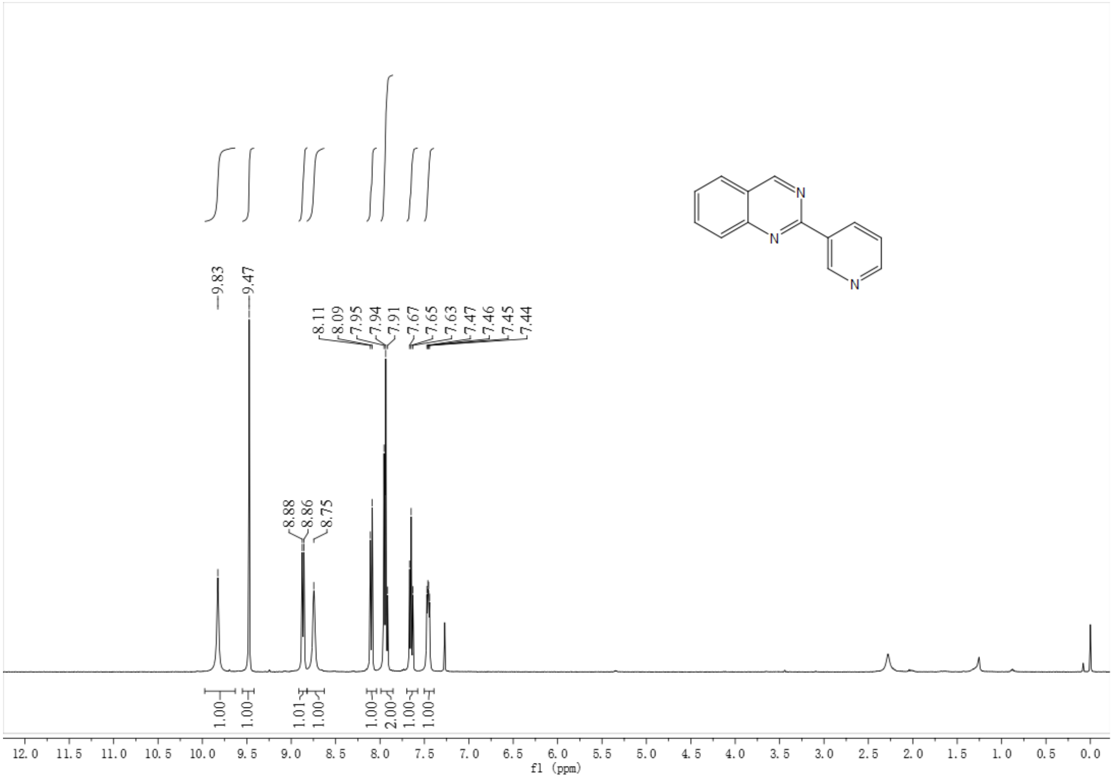

Figure S50. ^{13}C-NMR (100 MHz, $CDCl_3$) spectrum of **3ao**, related to **Scheme 2**.

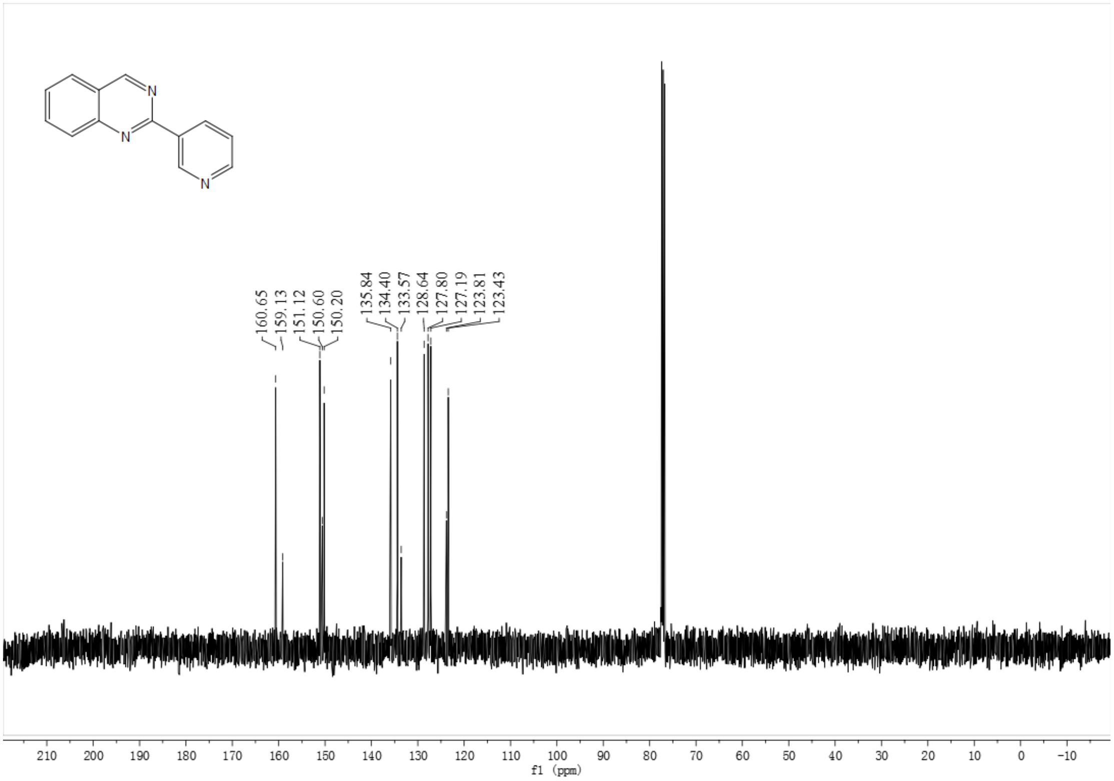

Figure S51. ^{1}H-NMR (400 MHz, $CDCl_3$) spectrum of **3ap**, related to **Scheme 2**.

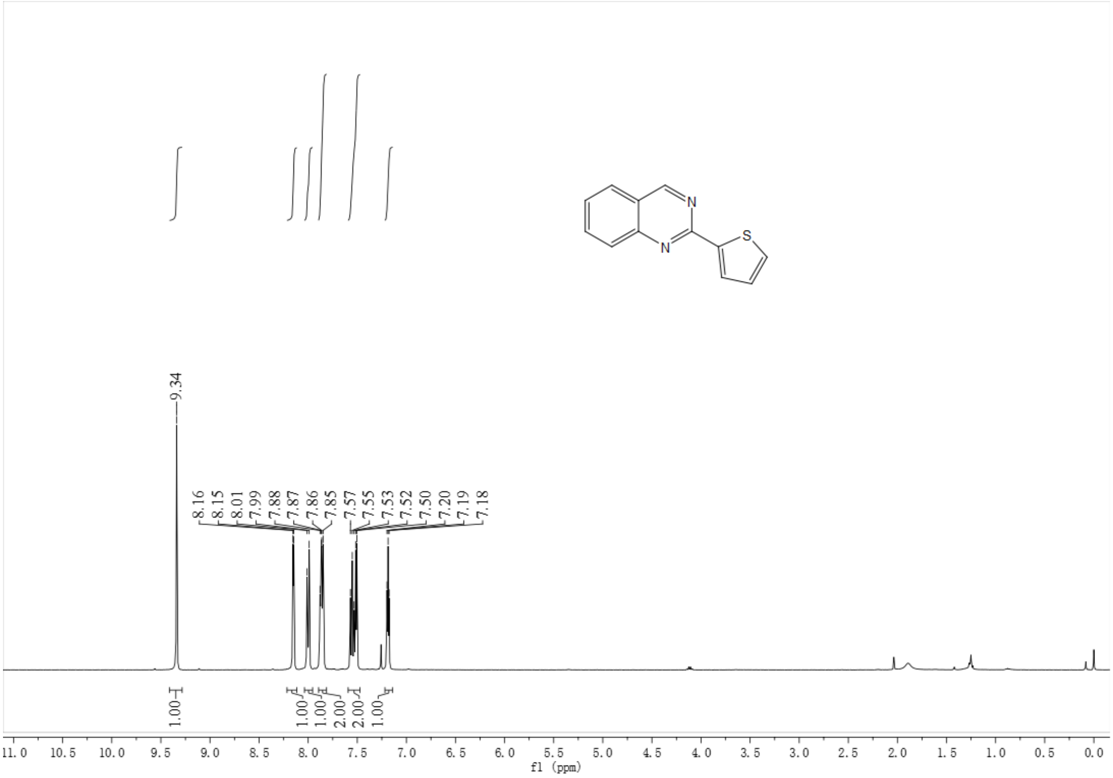

Figure S52. ^{13}C-NMR (100 MHz, $CDCl_3$) spectrum of **3ap**, related to **Scheme 2**.

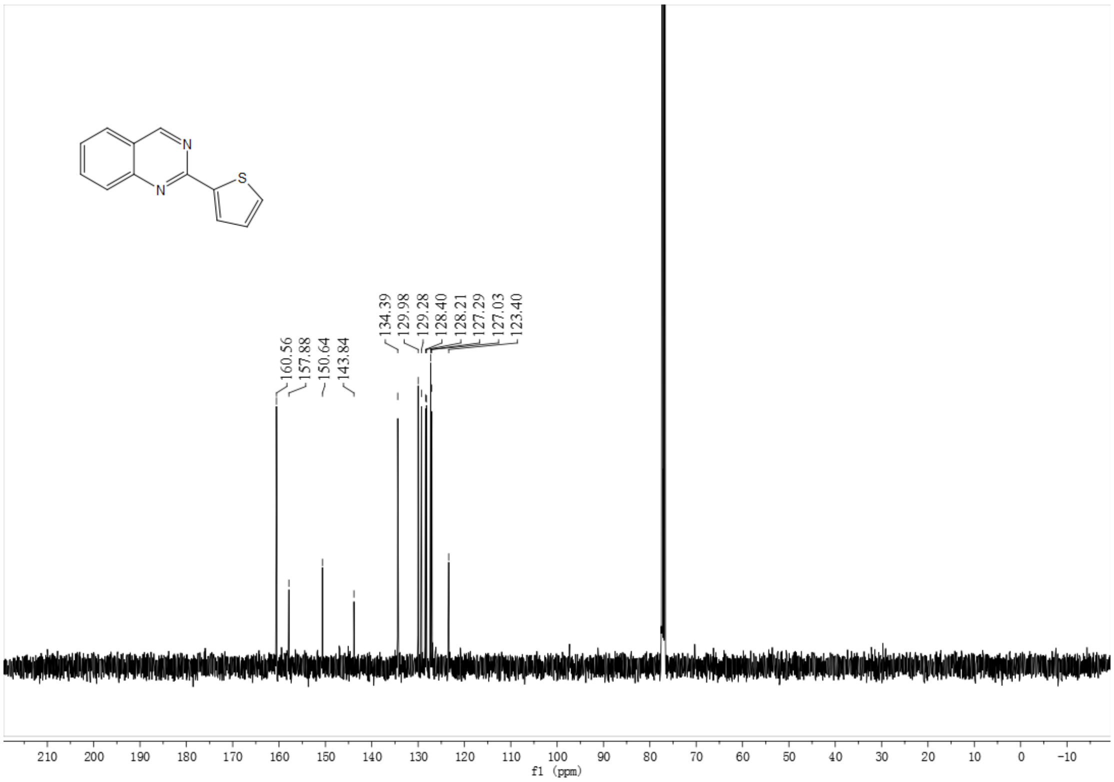

Figure S53. ^{1}H-NMR (400 MHz, $CDCl_3$) spectrum of **3aq**, related to **Scheme 2**.

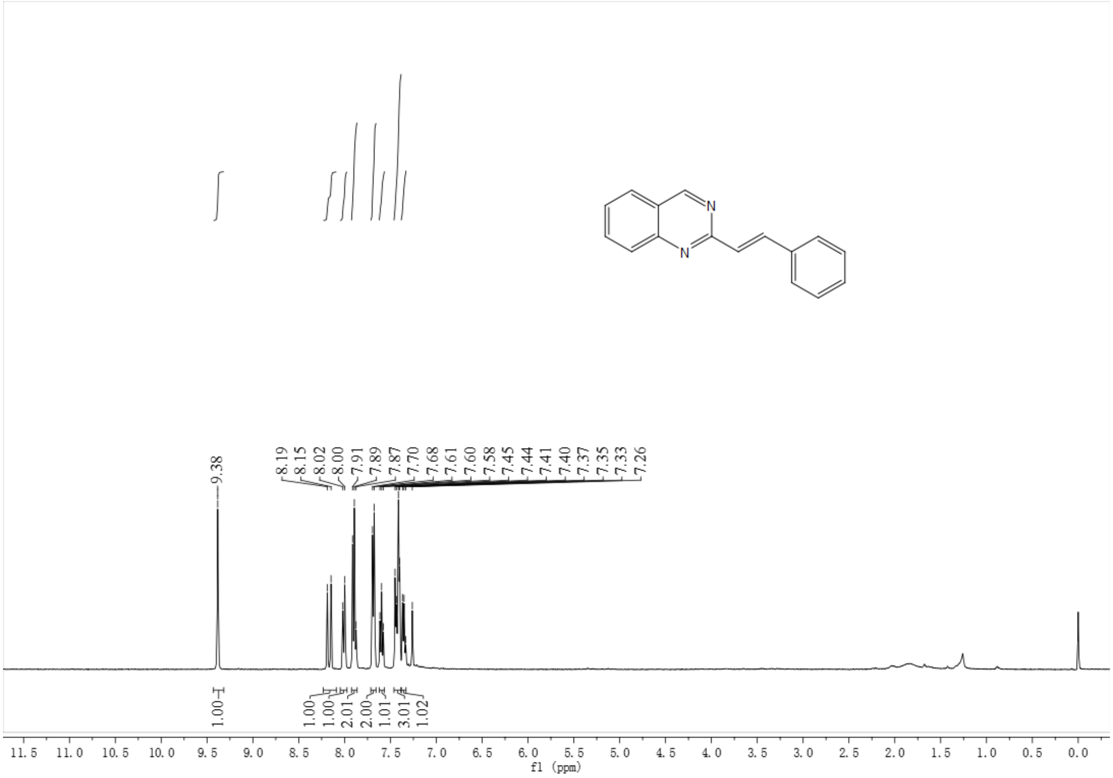

Figure S54. ^{13}C-NMR (100 MHz, $CDCl_3$) spectrum of **3aq**, related to **Scheme 2**.

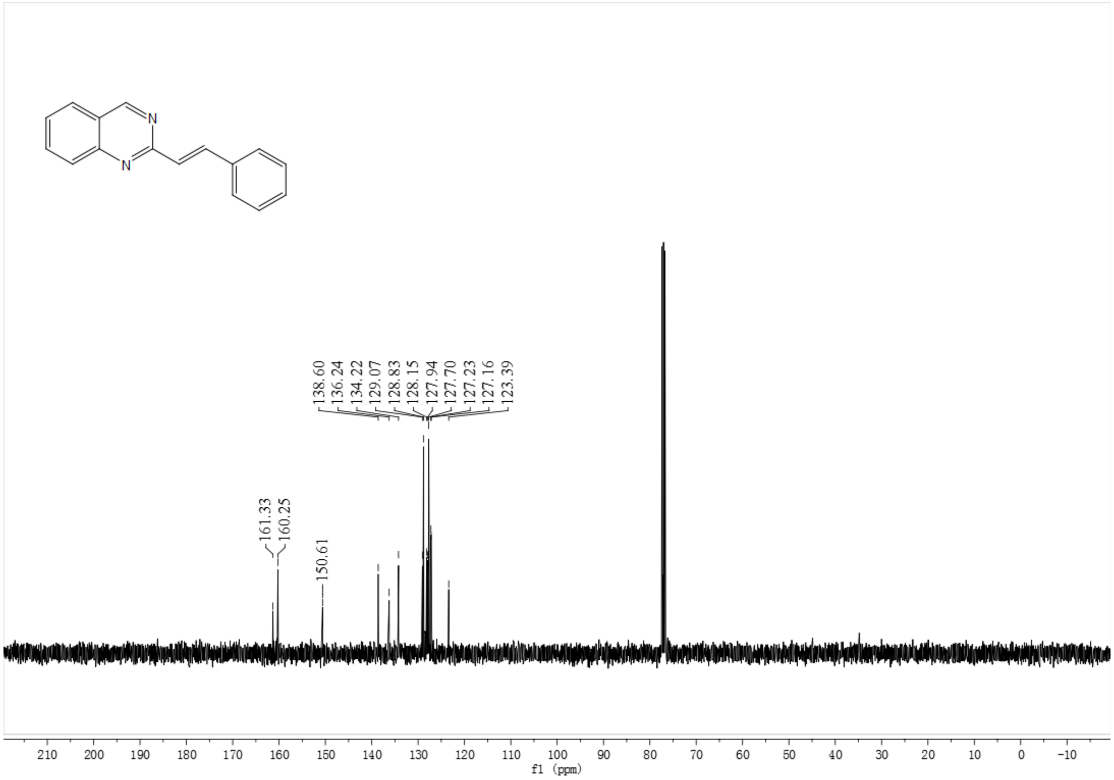

Figure S55. ^{1}H-NMR (400 MHz, $CDCl_3$) spectrum of **3ar**, related to **Scheme 2**.

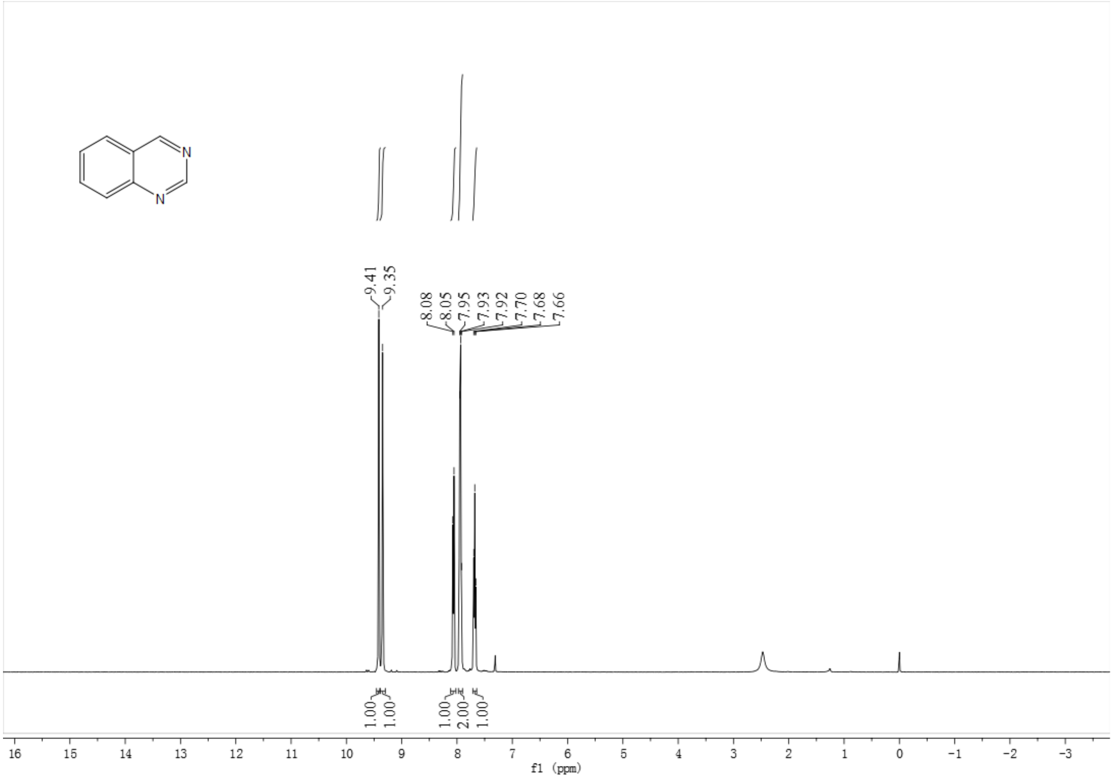

Figure S56. ^{13}C-NMR (100 MHz, $CDCl_3$) spectrum of **3ar**, related to **Scheme 2**.

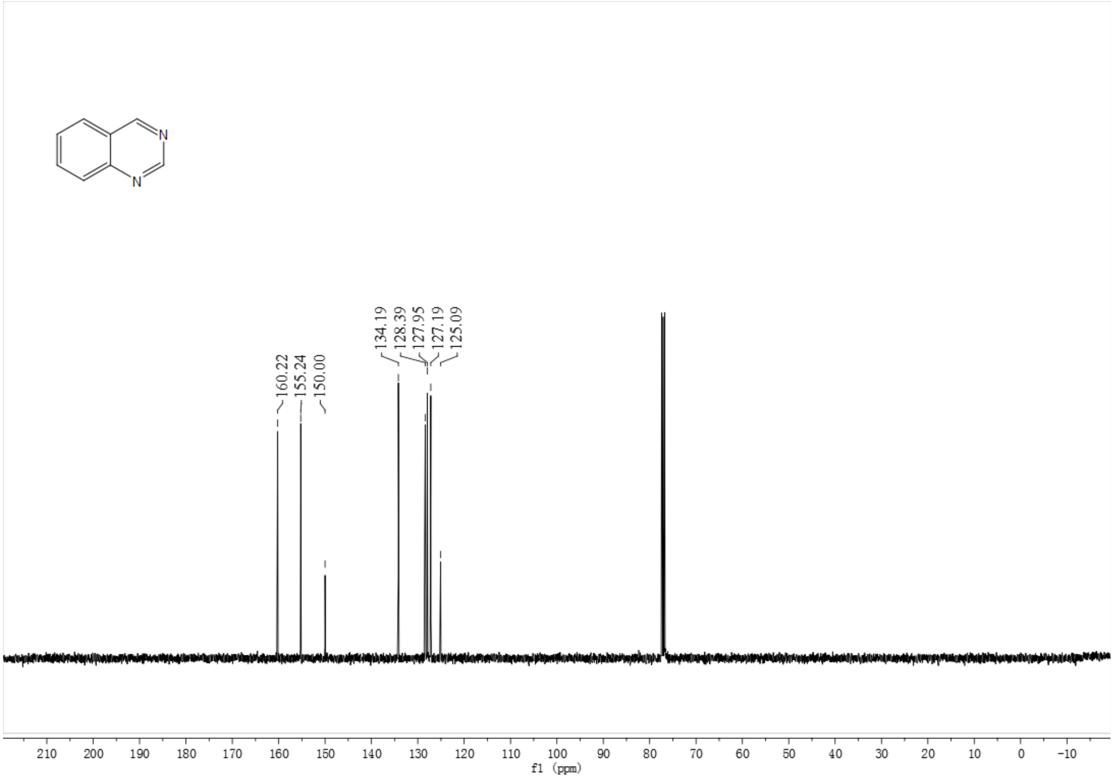

Figure S57. ^{1}H-NMR (400 MHz, $CDCl_3$) spectrum of **3as**, related to **Scheme 2**.

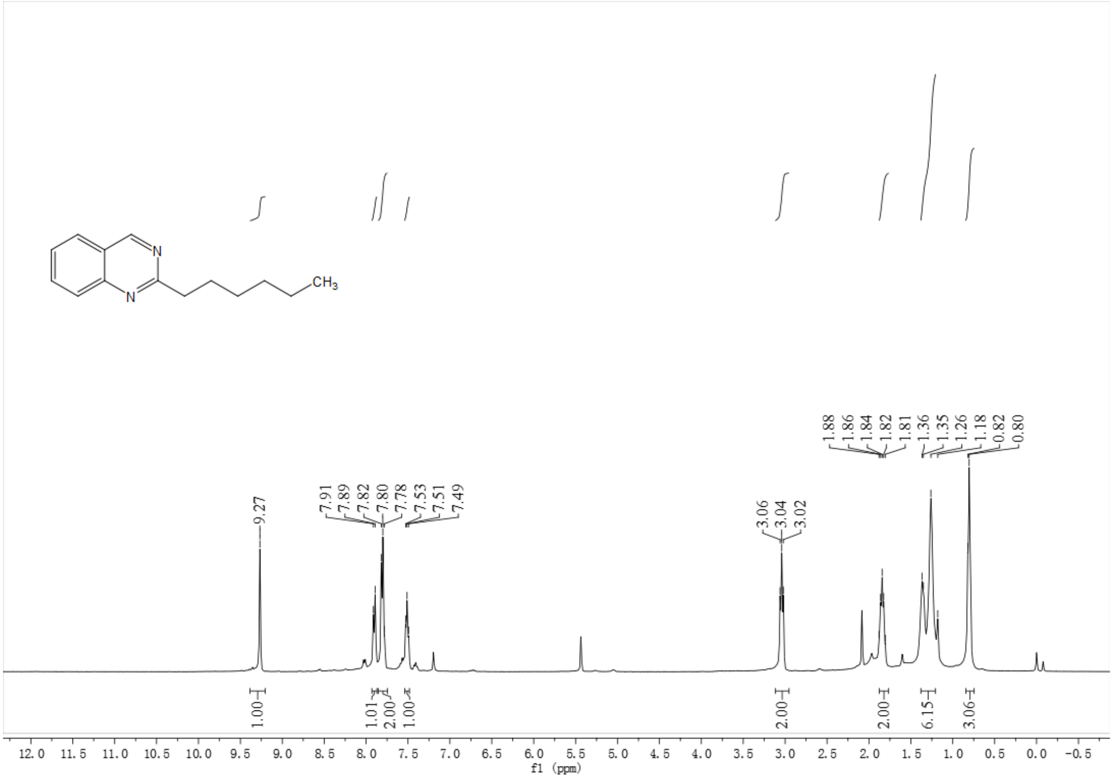

Figure S58. ^{13}C-NMR (100 MHz, $CDCl_3$) spectrum of **3as**, related to **Scheme 2**.

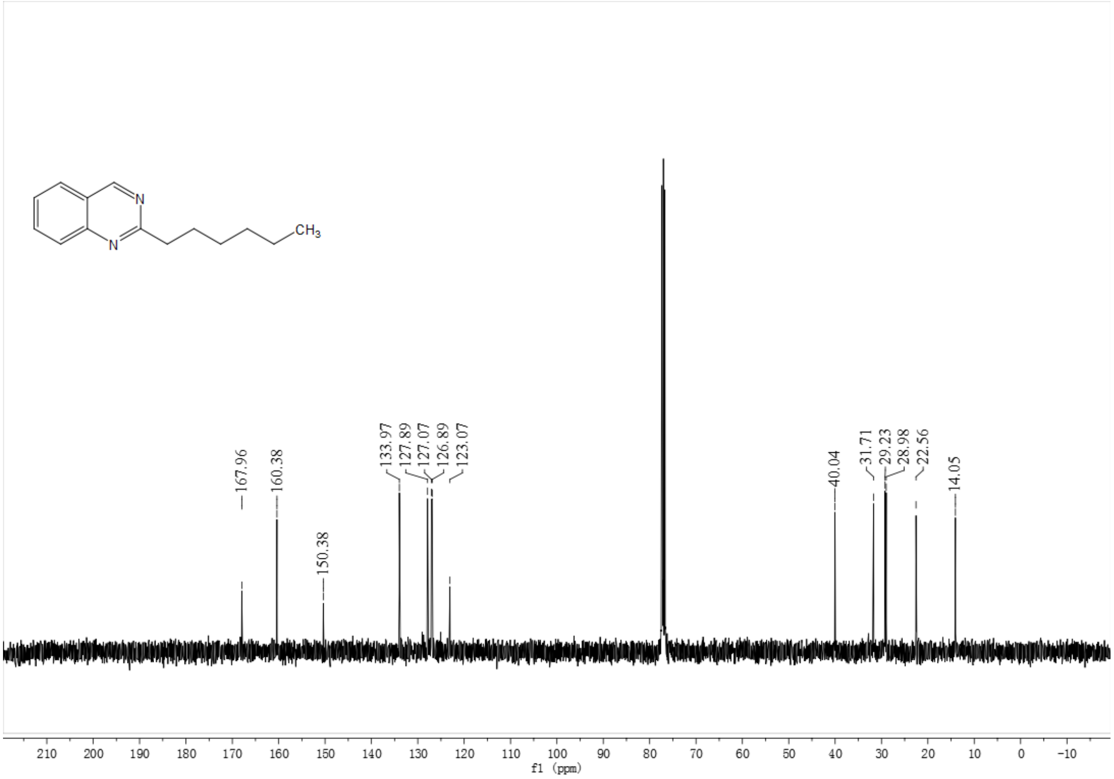

Figure S59. ^{1}H-NMR (400 MHz, $CDCl_3$) spectrum of **3at**, related to **Scheme 2**.

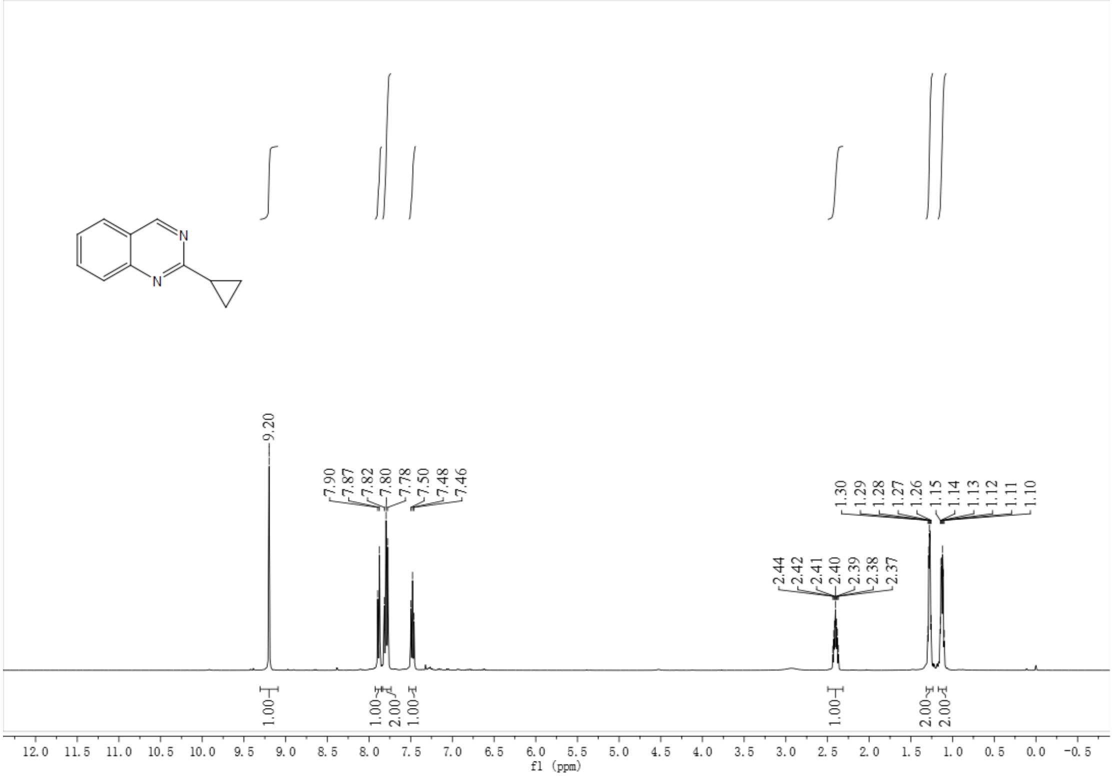

Figure S60. ^{13}C-NMR (100 MHz, $CDCl_3$) spectrum of **3at**, related to **Scheme 2**.

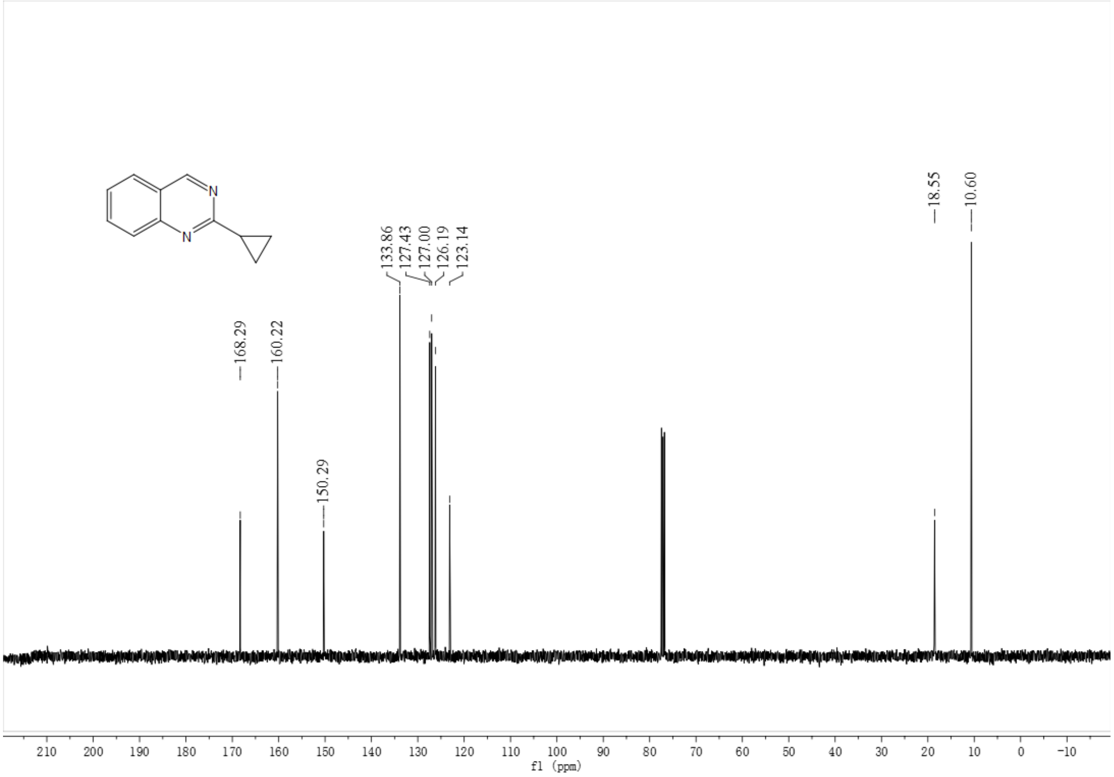

Figure S61. ^{1}H-NMR (400 MHz, $CDCl_3$) spectrum of **3ba**, related to **Scheme 3**.

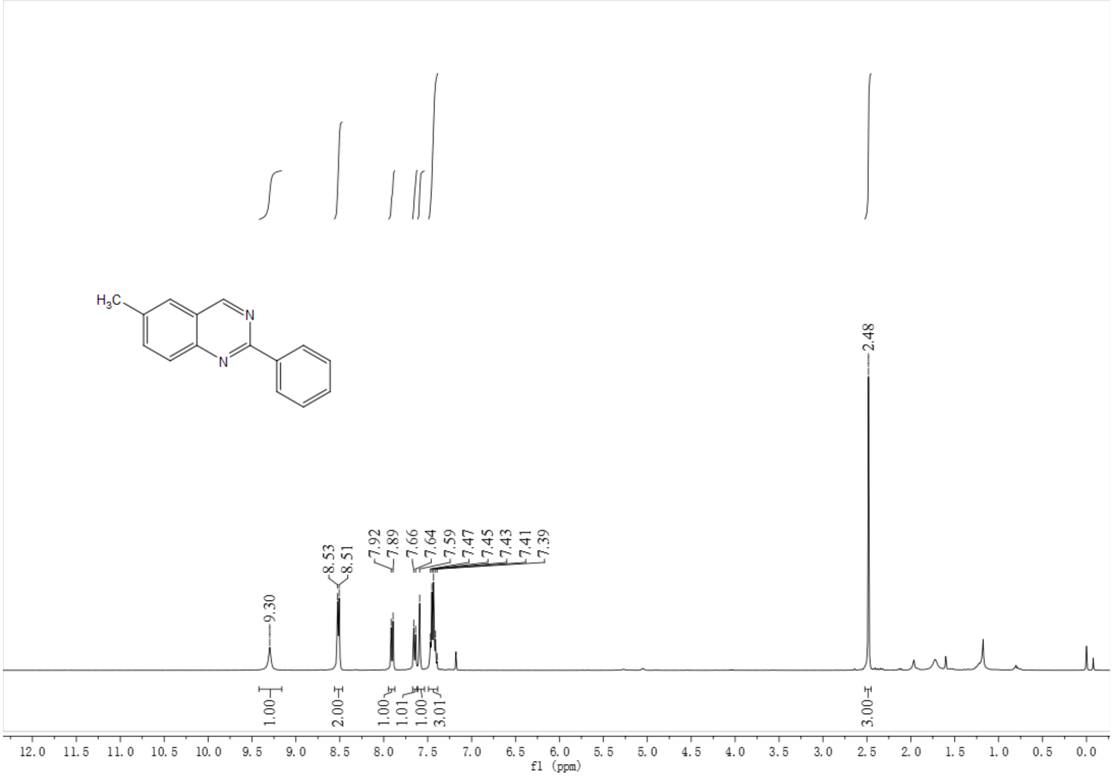

Figure S62. ^{13}C-NMR (100 MHz, $CDCl_3$) spectrum of **3ba**, related to **Scheme 3**.

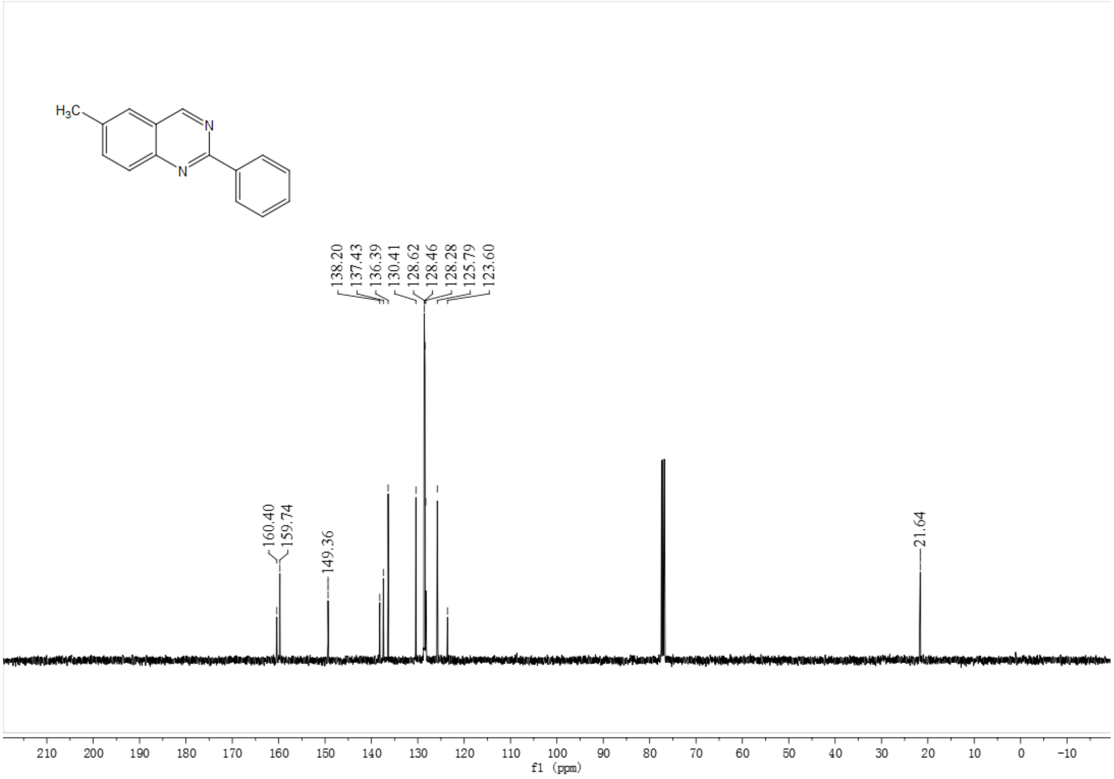

Figure S63. ^{1}H-NMR (400 MHz, $CDCl_3$) spectrum of **3bd**, related to **Scheme 3**.

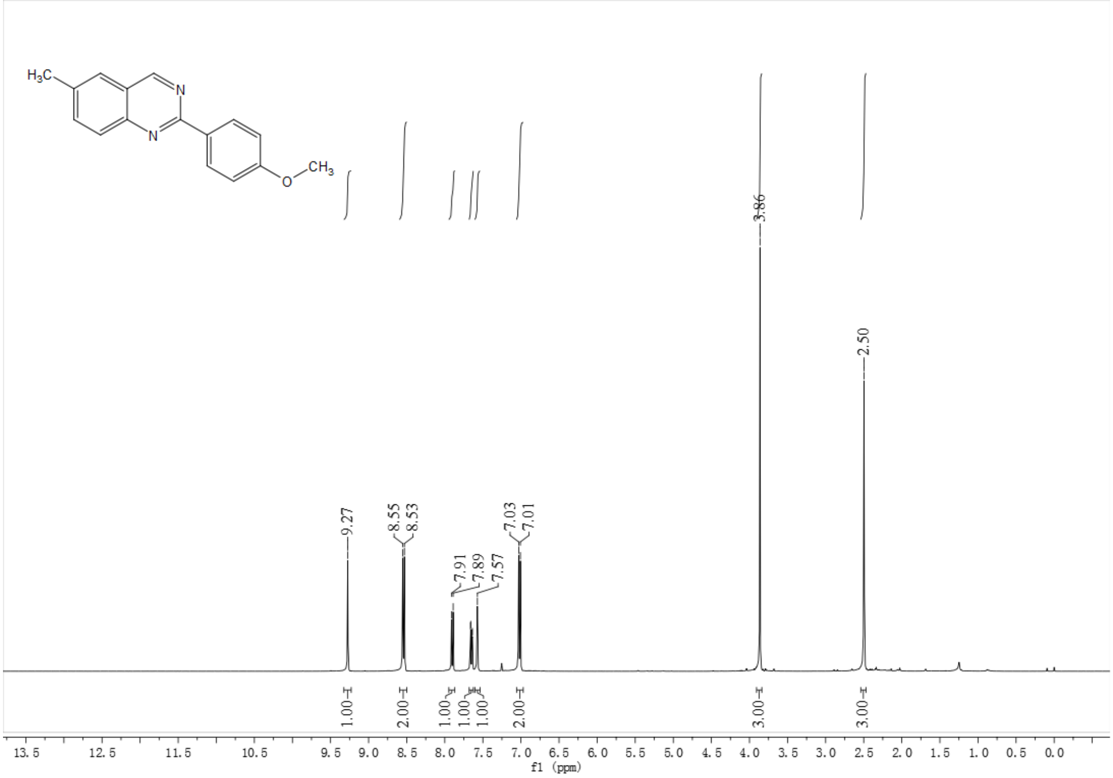

Figure S64. ^{13}C-NMR (100 MHz, $CDCl_3$) spectrum of **3bd**, related to **Scheme 3**.

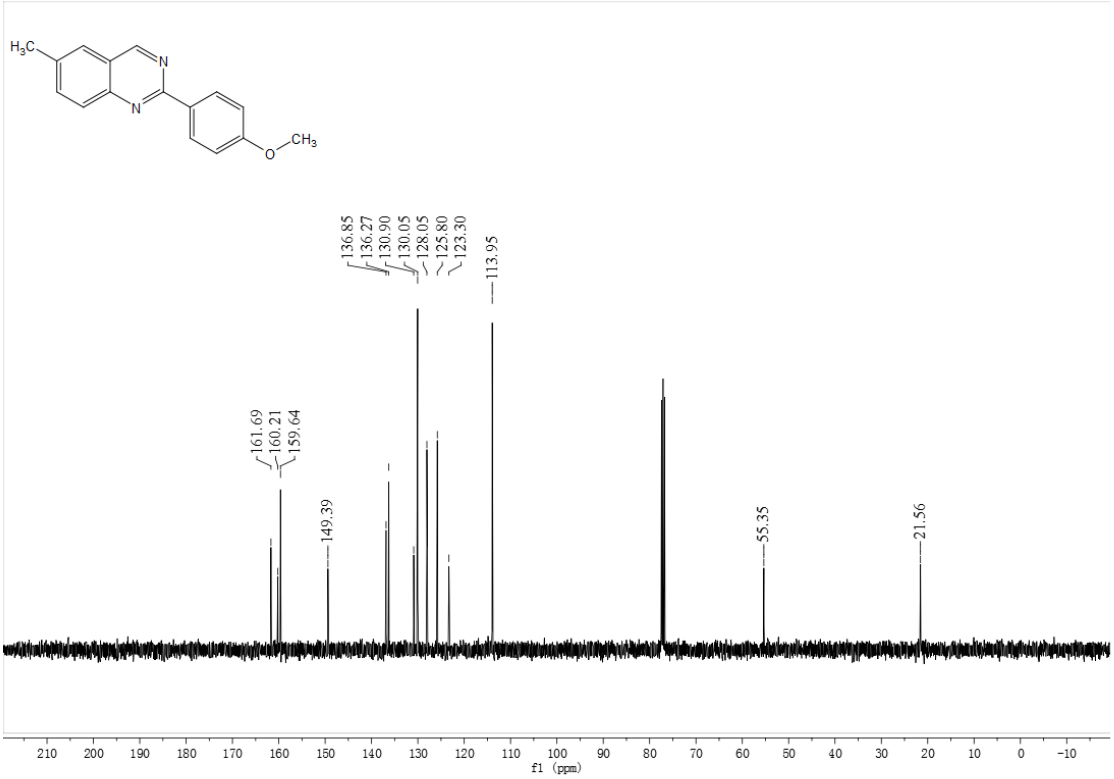

Figure S65. ^{1}H-NMR (400 MHz, $CDCl_3$) spectrum of **3ca**, related to **Scheme 3**.

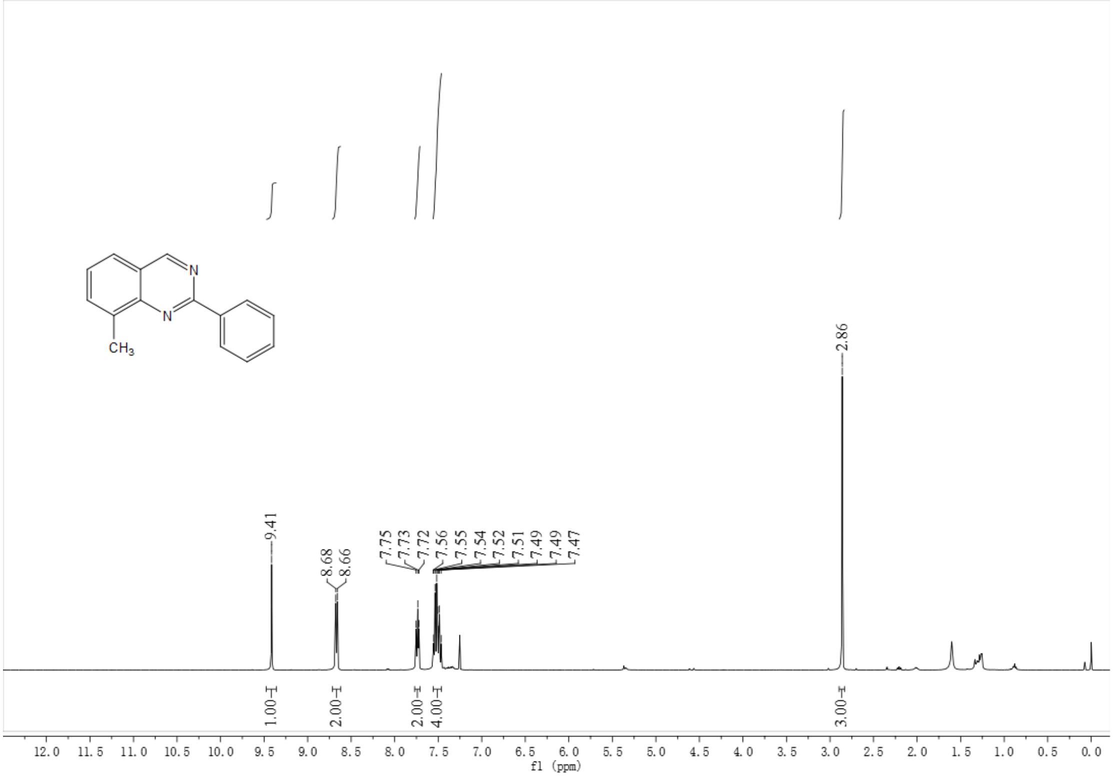

Figure S66. ^{13}C-NMR (100 MHz, $CDCl_3$) spectrum of **3ca**, related to **Scheme 3**.

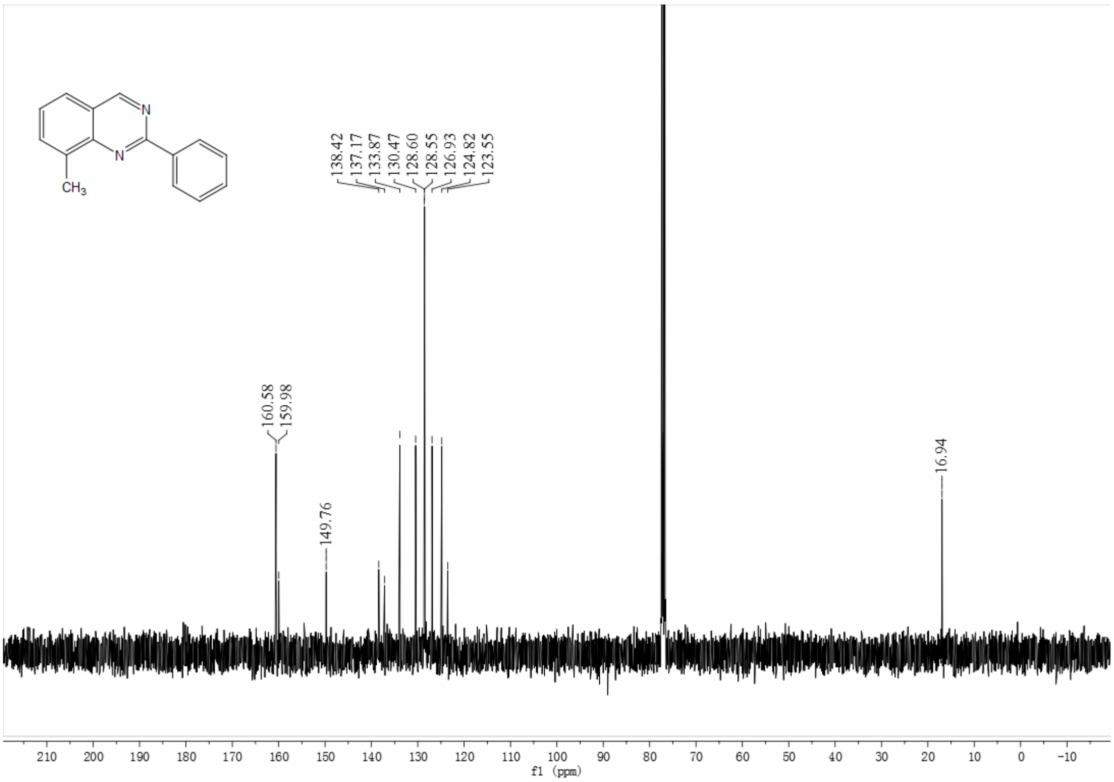

Figure S67. ^{1}H-NMR (400 MHz, $CDCl_3$) spectrum of **3da**, related to **Scheme 3**.

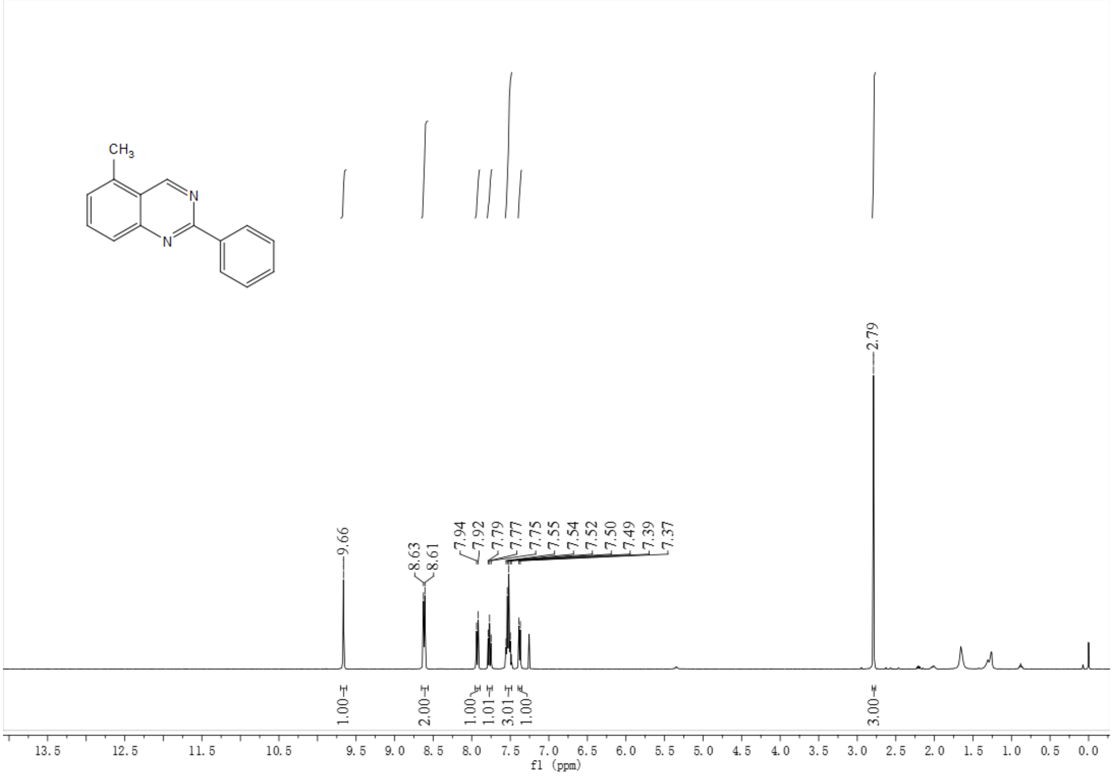

Figure S68. ^{13}C-NMR (100 MHz, $CDCl_3$) spectrum of **3da**, related to **Scheme 3**.

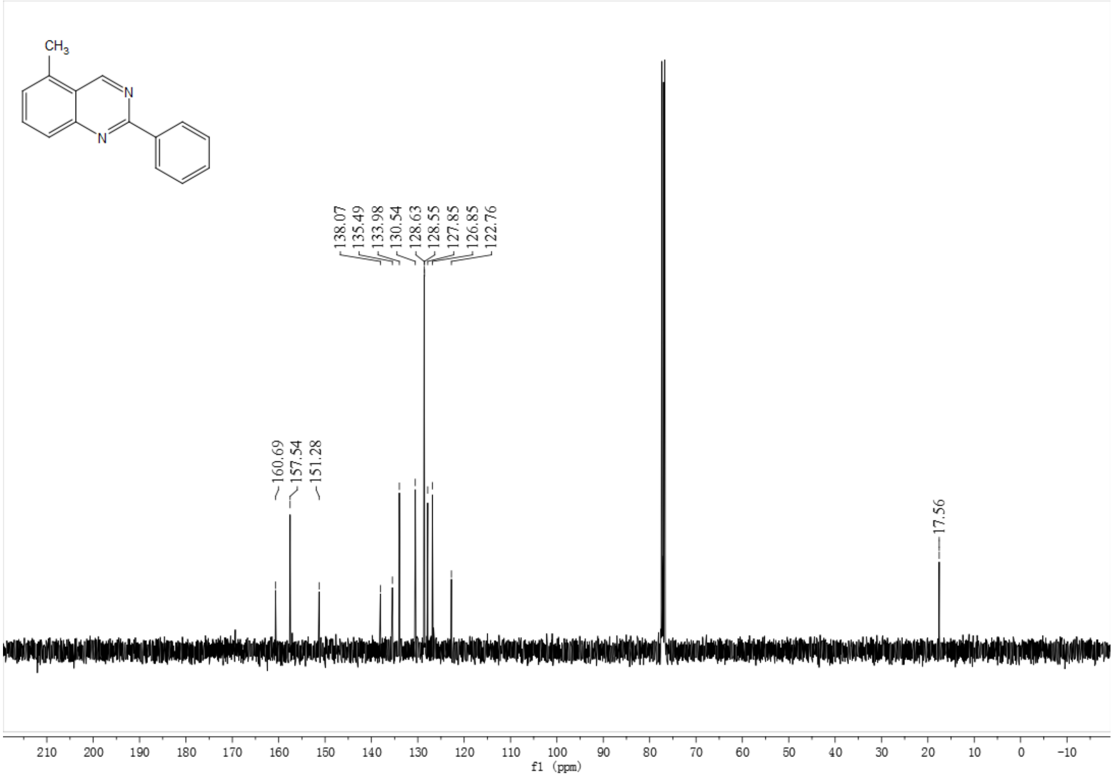

Figure S69. ^{1}H-NMR (400 MHz, $CDCl_3$) spectrum of **3ea**, related to **Scheme 3**.

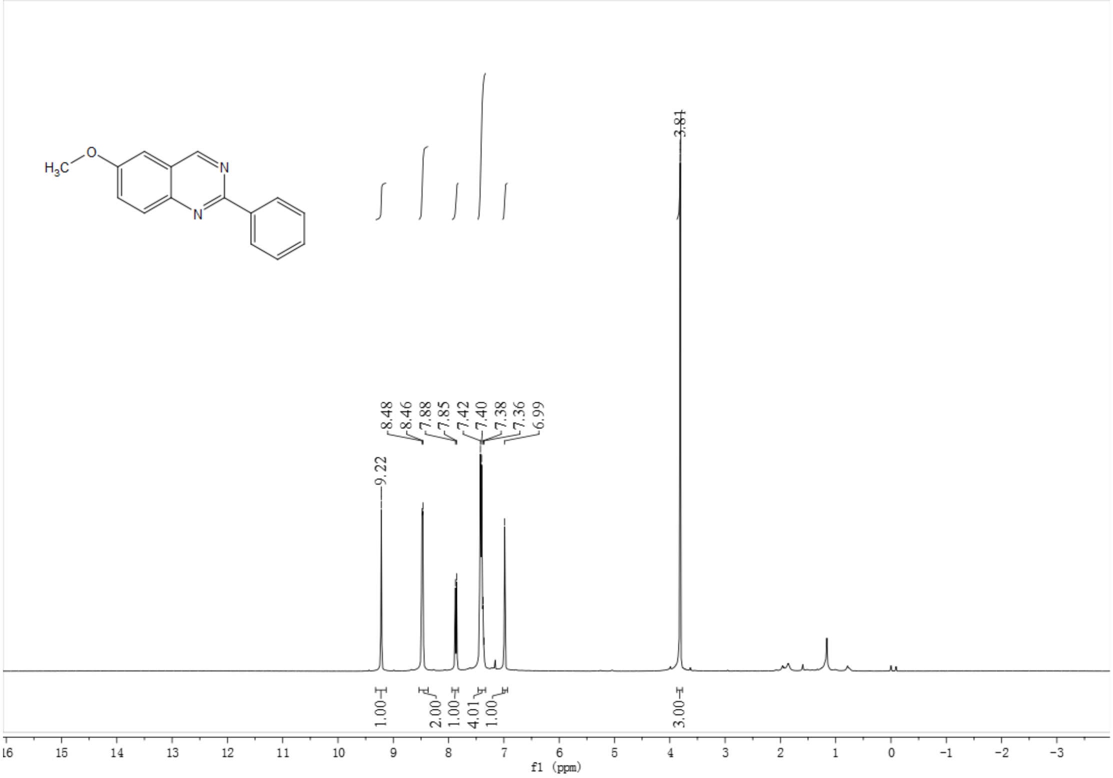

Figure S70. ^{13}C-NMR (100 MHz, $CDCl_3$) spectrum of **3ea**, related to **Scheme 3**.

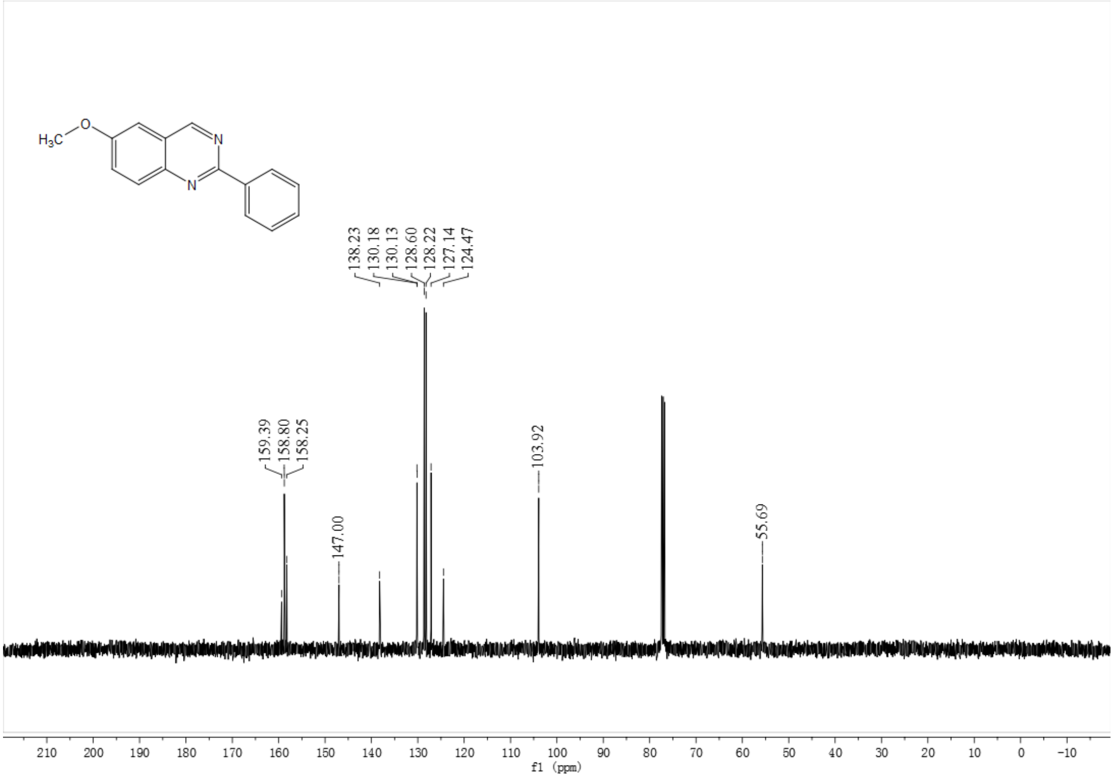

Figure S71. ^{1}H-NMR (400 MHz, $CDCl_3$) spectrum of **3eg**, related to **Scheme 3**.

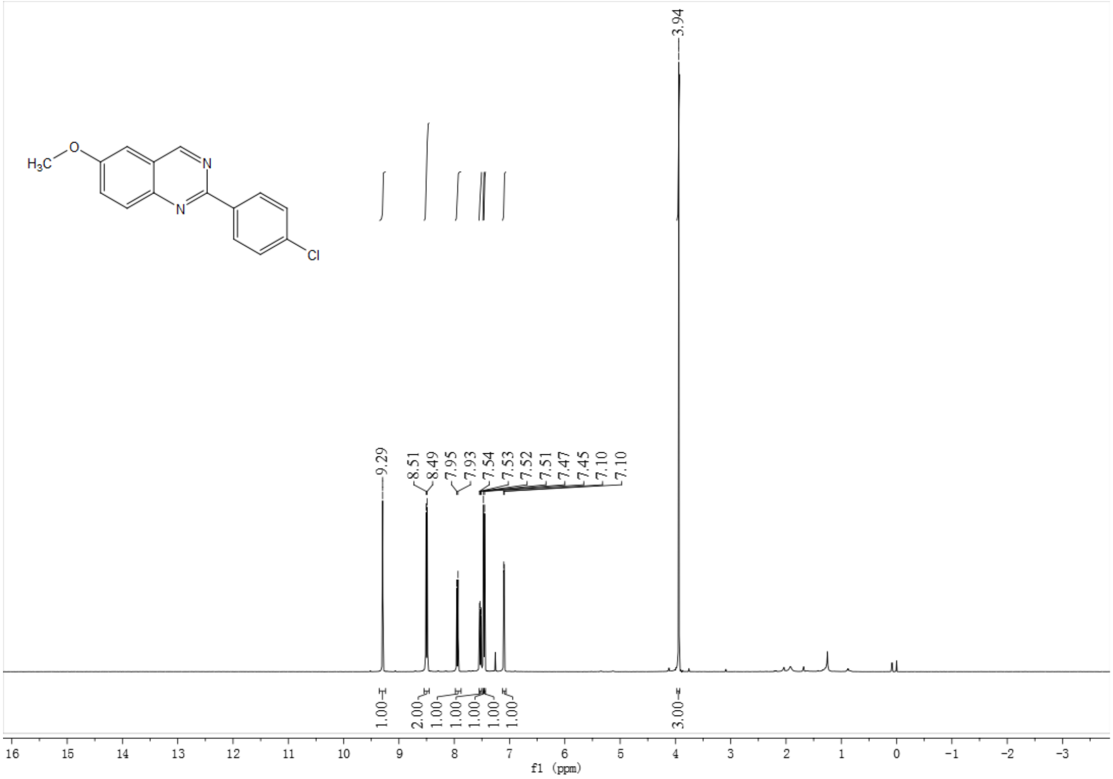

Figure S72. ^{13}C-NMR (100 MHz, $CDCl_3$) spectrum of **3eg**, related to **Scheme 3**.

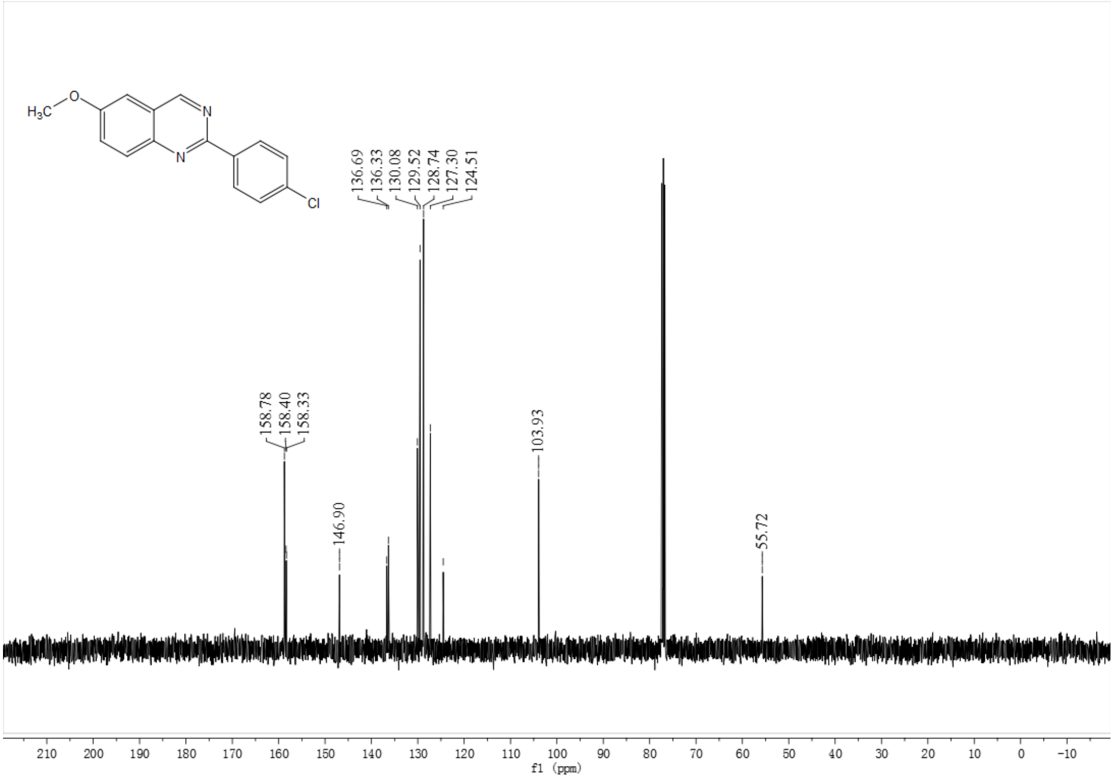

Figure S73. ^{1}H-NMR (400 MHz, $CDCl_3$) spectrum of **3fa**, related to **Scheme 3**.

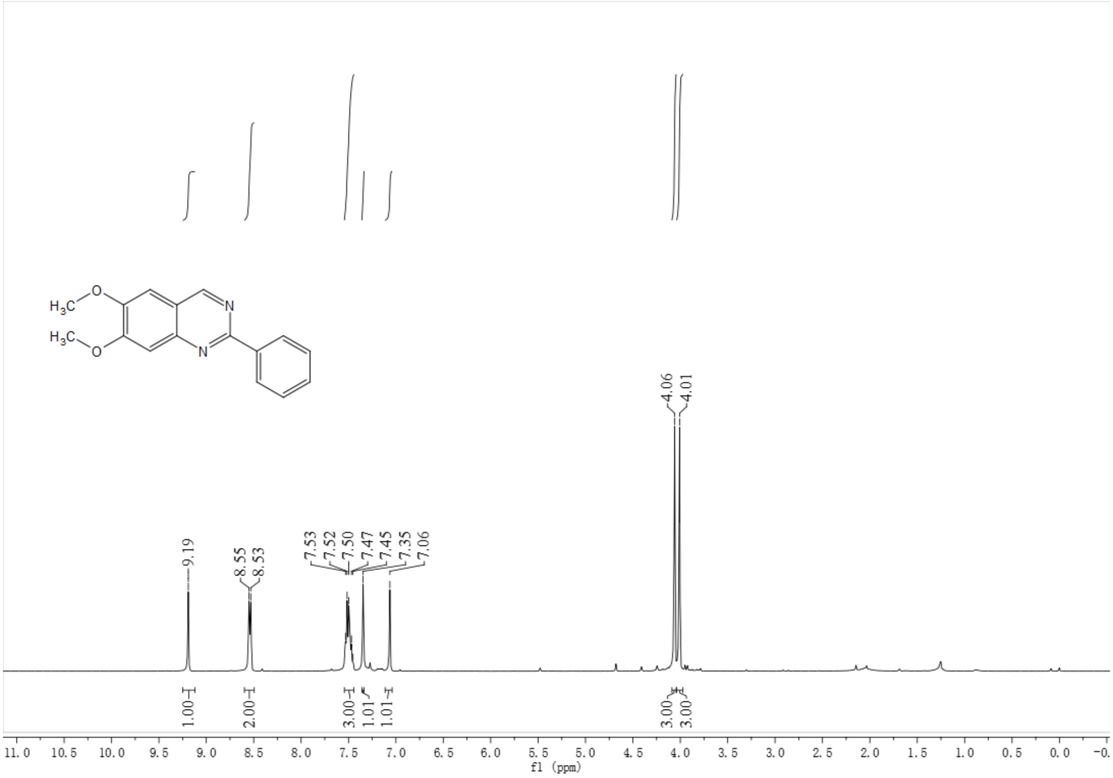

Figure S74. ^{13}C-NMR (100 MHz, $CDCl_3$) spectrum of **3fa**, related to **Scheme 3**.

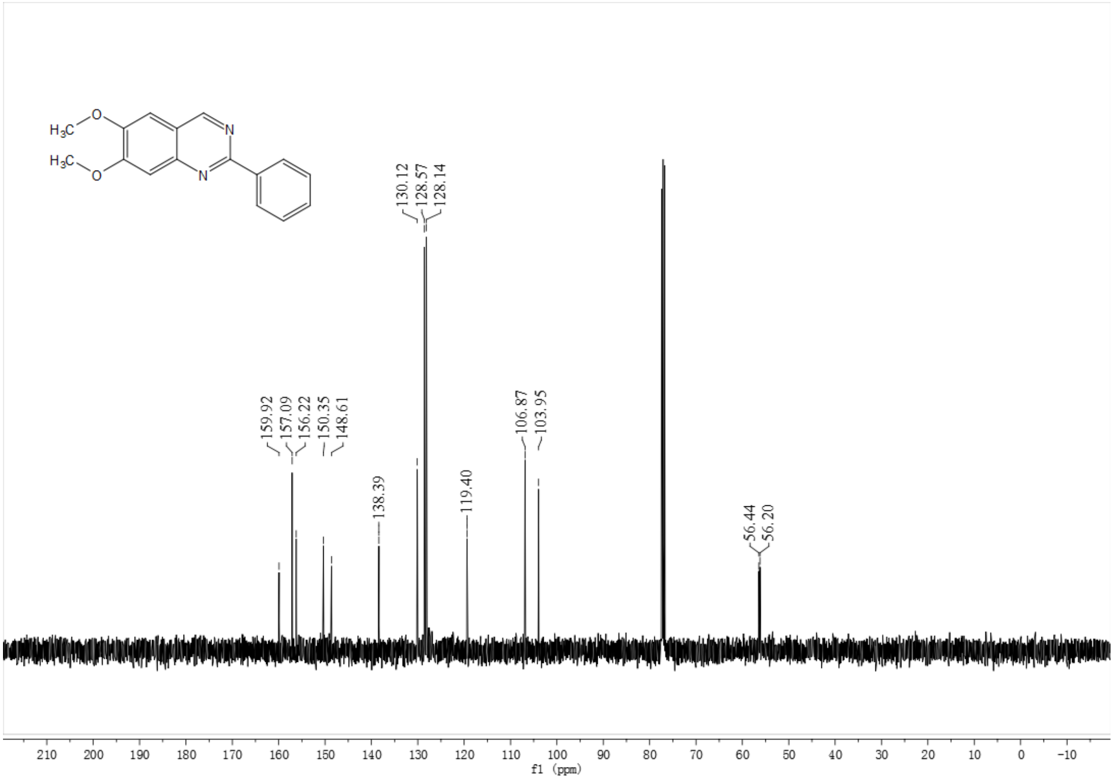

Figure S75. ^{1}H-NMR (400 MHz, $CDCl_3$) spectrum of **3fi**, related to **Scheme 3**.

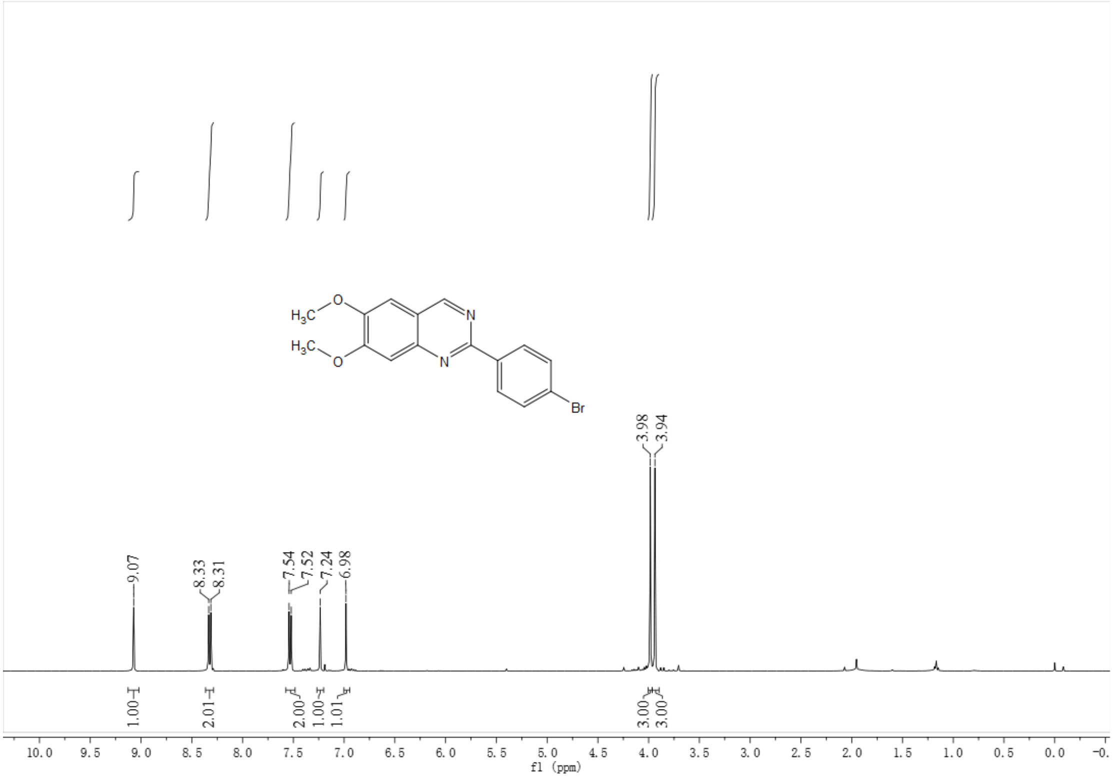

Figure S76. ^{13}C-NMR (100 MHz, $CDCl_3$) spectrum of **3fi**, related to **Scheme 3**.

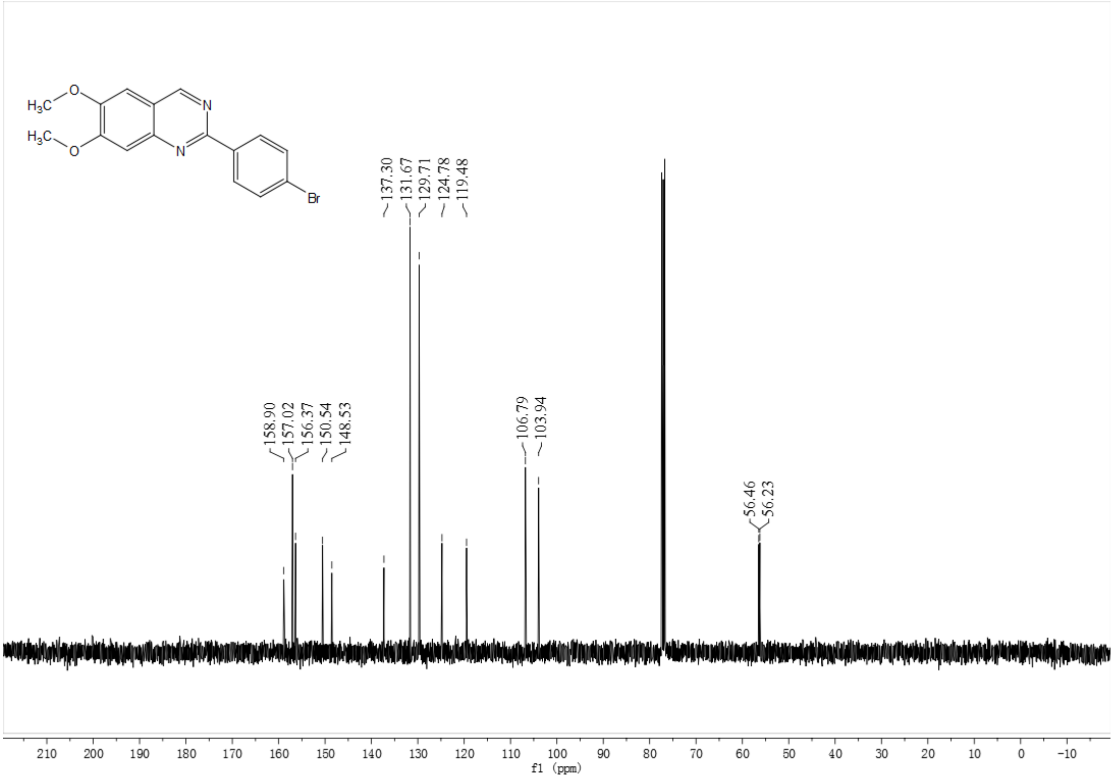

Figure S77. ^{1}H-NMR (400 MHz, $CDCl_3$) spectrum of **3ga**, related to **Scheme 3**.

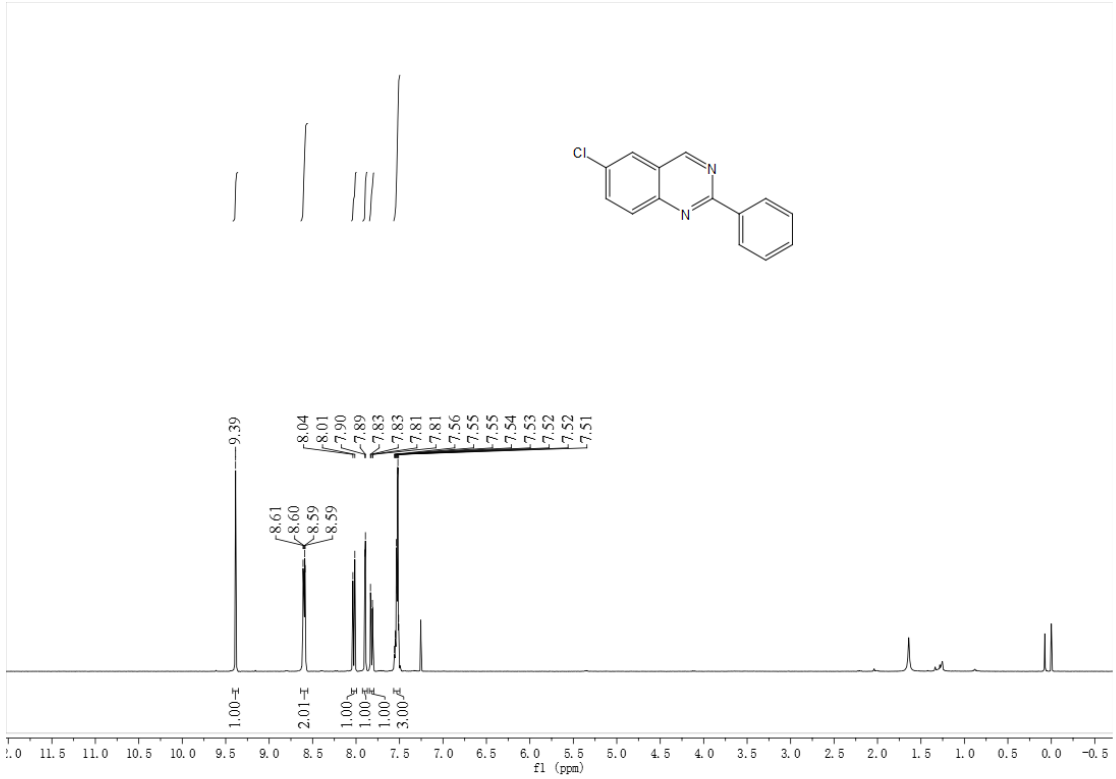

Figure S78. ^{13}C-NMR (100 MHz, $CDCl_3$) spectrum of **3ga**, related to **Scheme 3**.

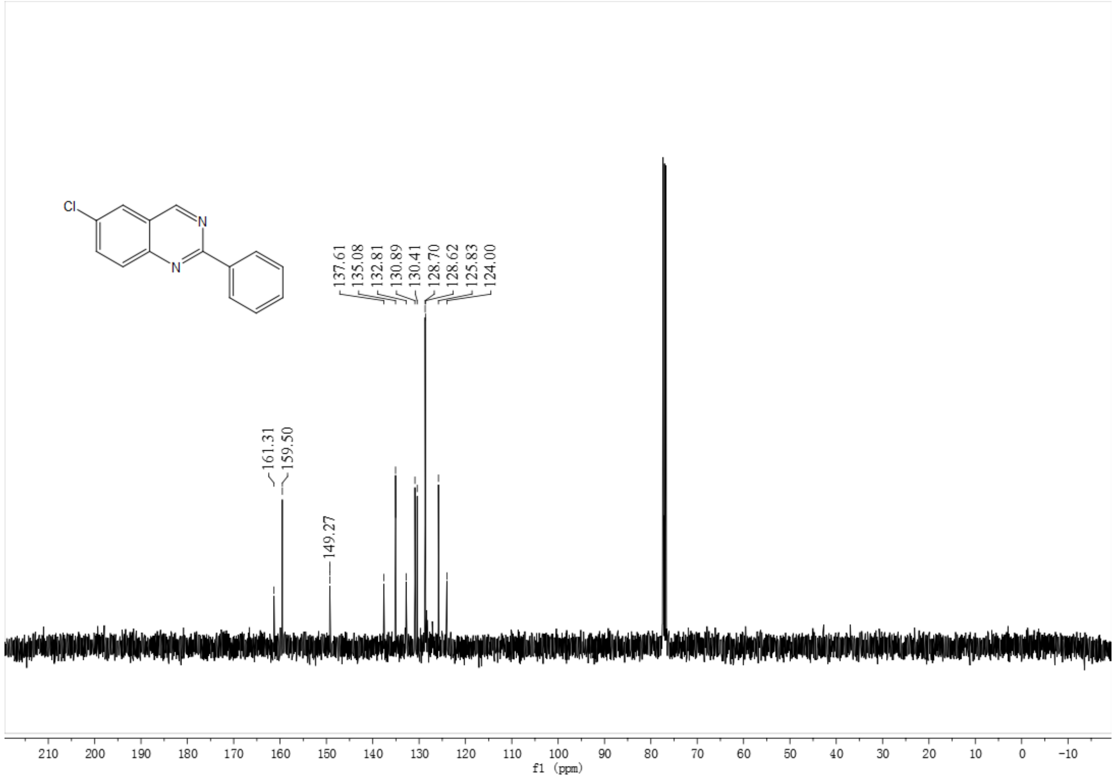

Figure S79. ^{1}H-NMR (400 MHz, $CDCl_3$) spectrum of **3gk**, related to **Scheme 3**.

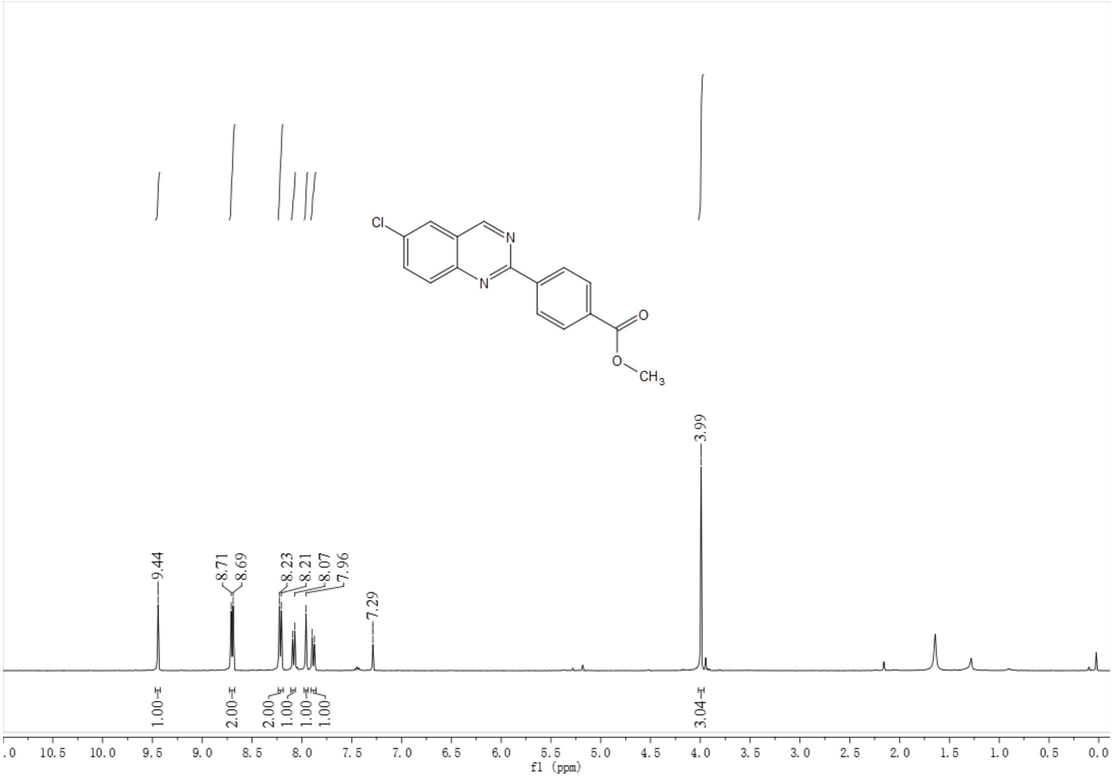

Figure S80. ^{13}C-NMR (100 MHz, $CDCl_3$) spectrum of **3gk**, related to **Scheme 3**.

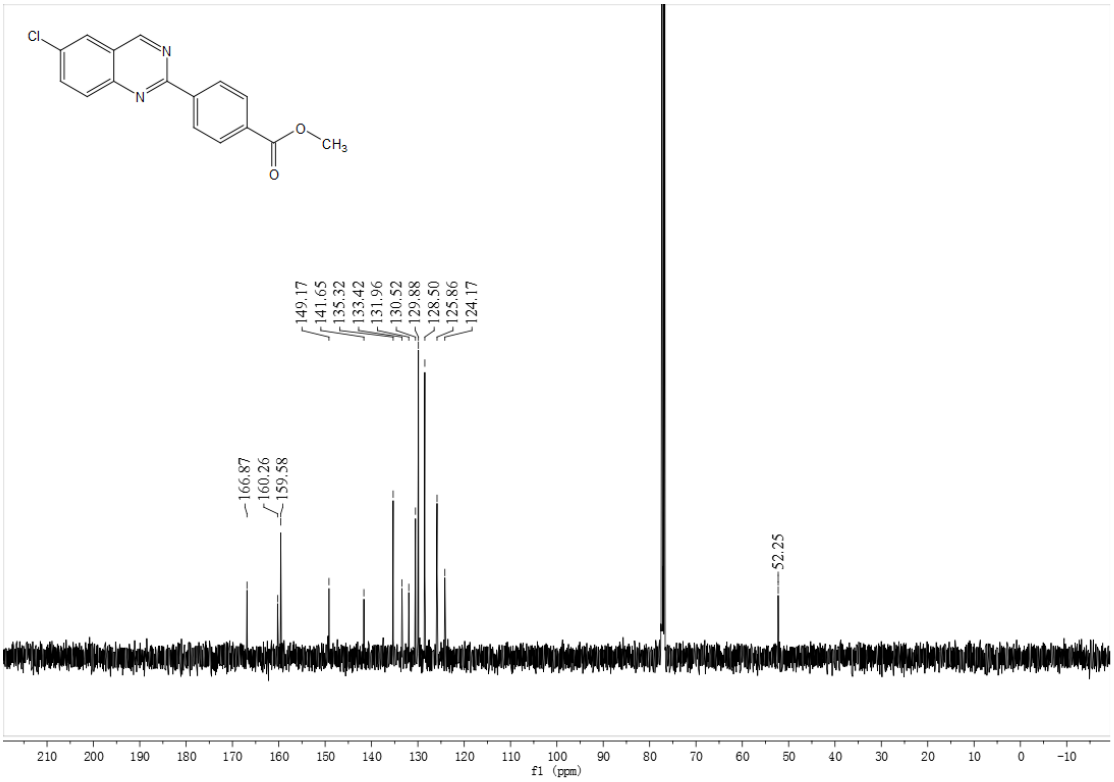

Figure S81. ^{1}H-NMR (400 MHz, $CDCl_3$) spectrum of **3ha**, related to **Scheme 3**.

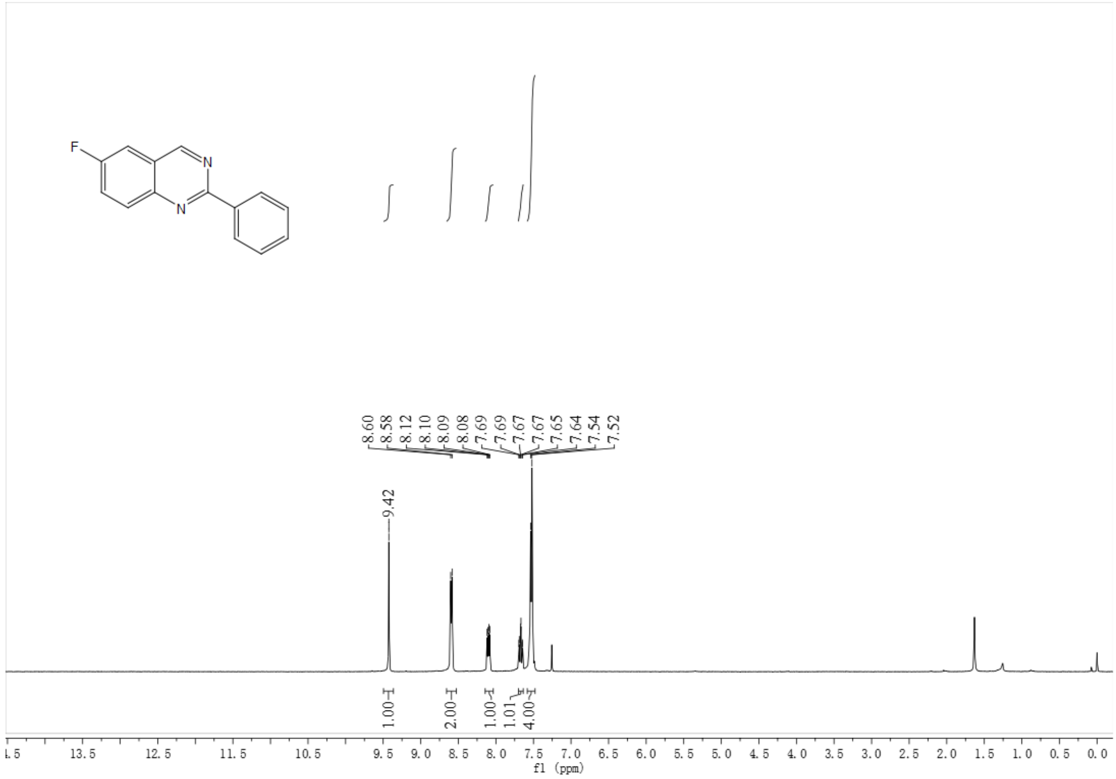

Figure S82. ^{13}C-NMR (100 MHz, $CDCl_3$) spectrum of **3ha**, related to **Scheme 3**.

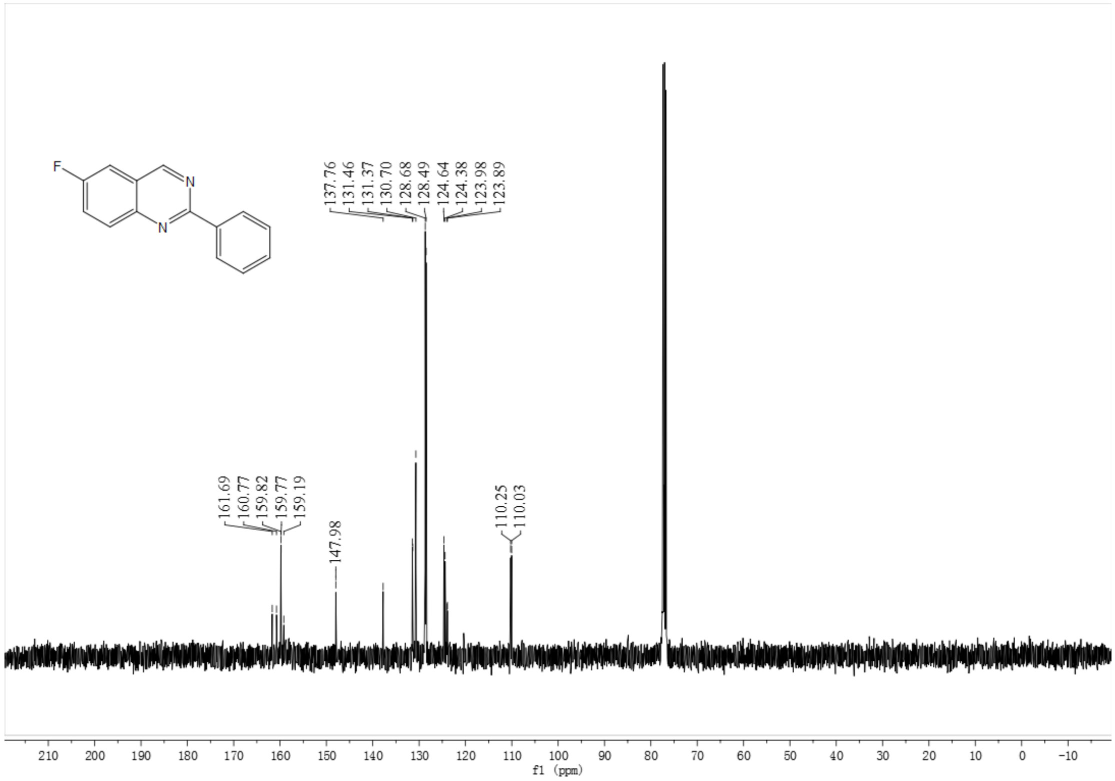

Figure S83. ^{19}F-NMR (400 MHz, $CDCl_3$) spectrum of **3ha**, related to **Scheme 3**.

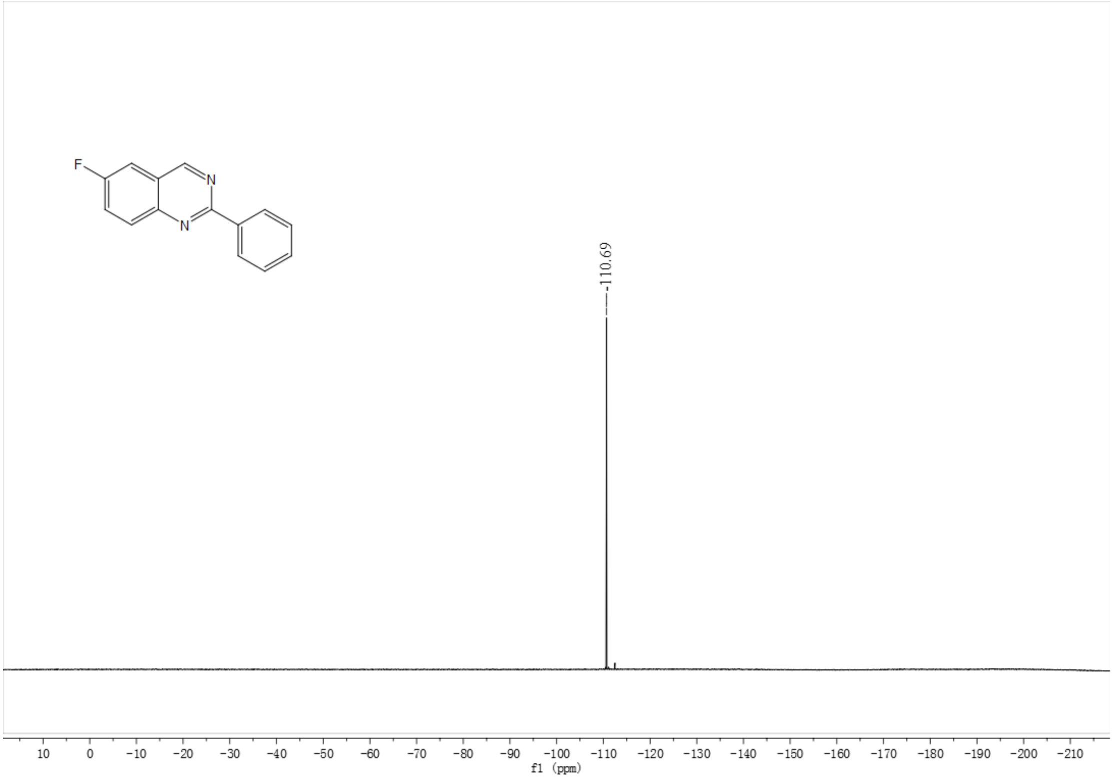

Figure S84. ^{1}H-NMR (400 MHz, $CDCl_3$) spectrum of **3ia**, related to **Scheme 3**.

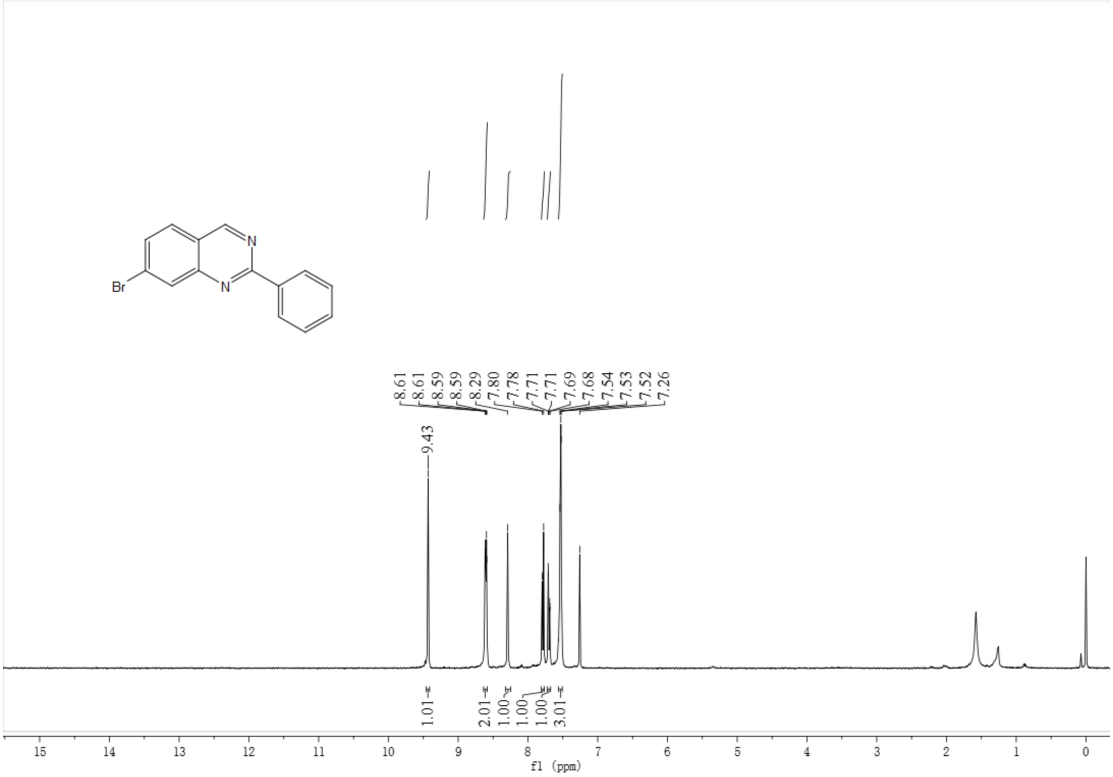

Figure S85. ^{13}C-NMR (100 MHz, $CDCl_3$) spectrum of **3ia**, related to **Scheme 3**.

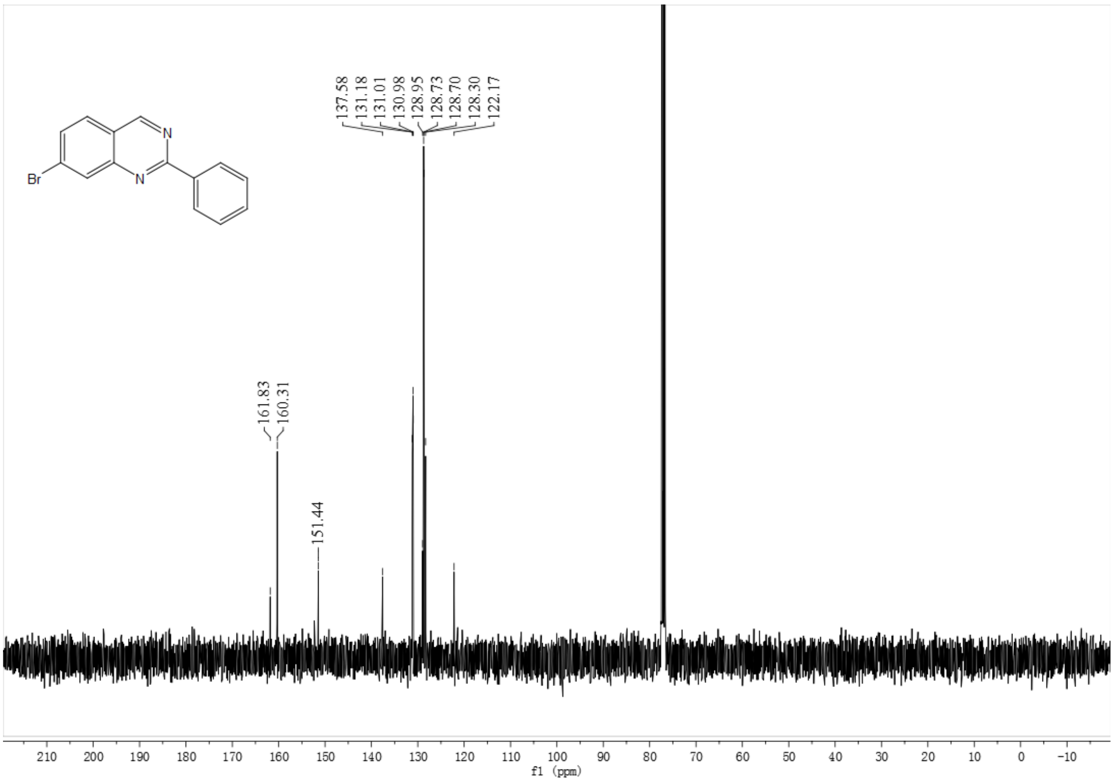

Figure S86. ^{1}H-NMR (400 MHz, $CDCl_3$) spectrum of **3ja**, related to **Scheme 3**.

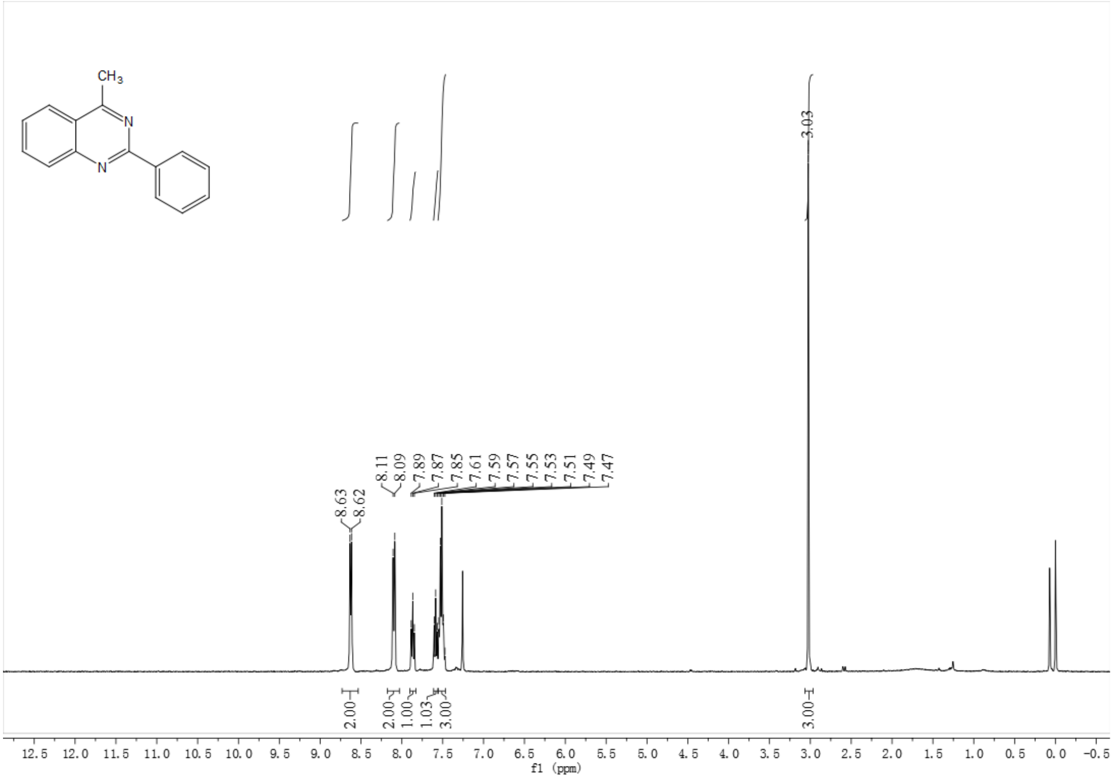

Figure S87. ^{13}C-NMR (100 MHz, $CDCl_3$) spectrum of **3ja**, related to **Scheme 3**.

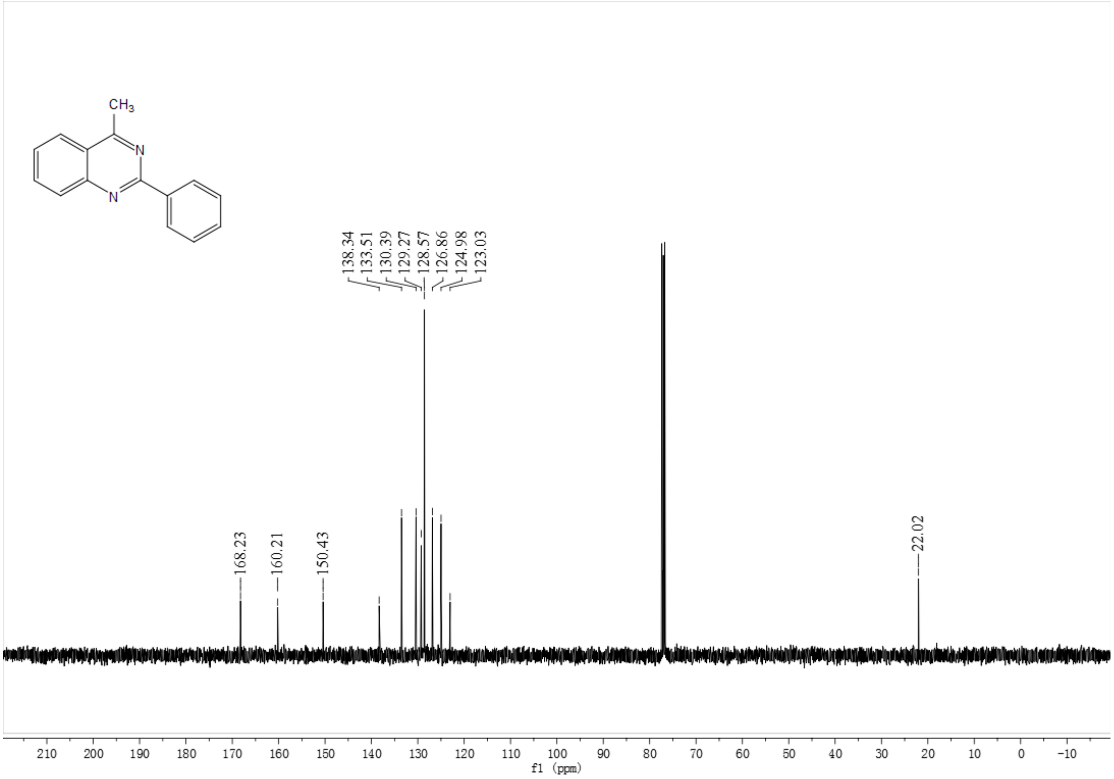

Figure S88. ^{1}H-NMR (400 MHz, $CDCl_3$) spectrum of **3jl**, related to **Scheme 2**.

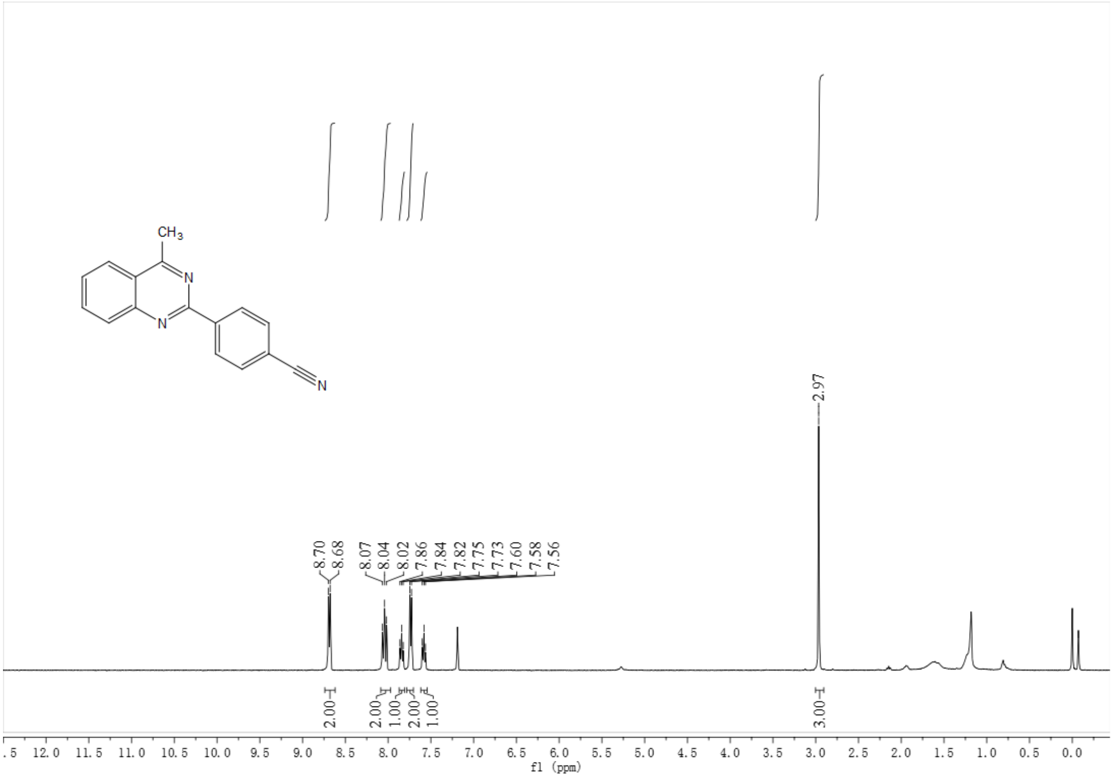

Figure S89. ^{13}C-NMR (100 MHz, $CDCl_3$) spectrum of **3jl**, related to **Scheme 3**.

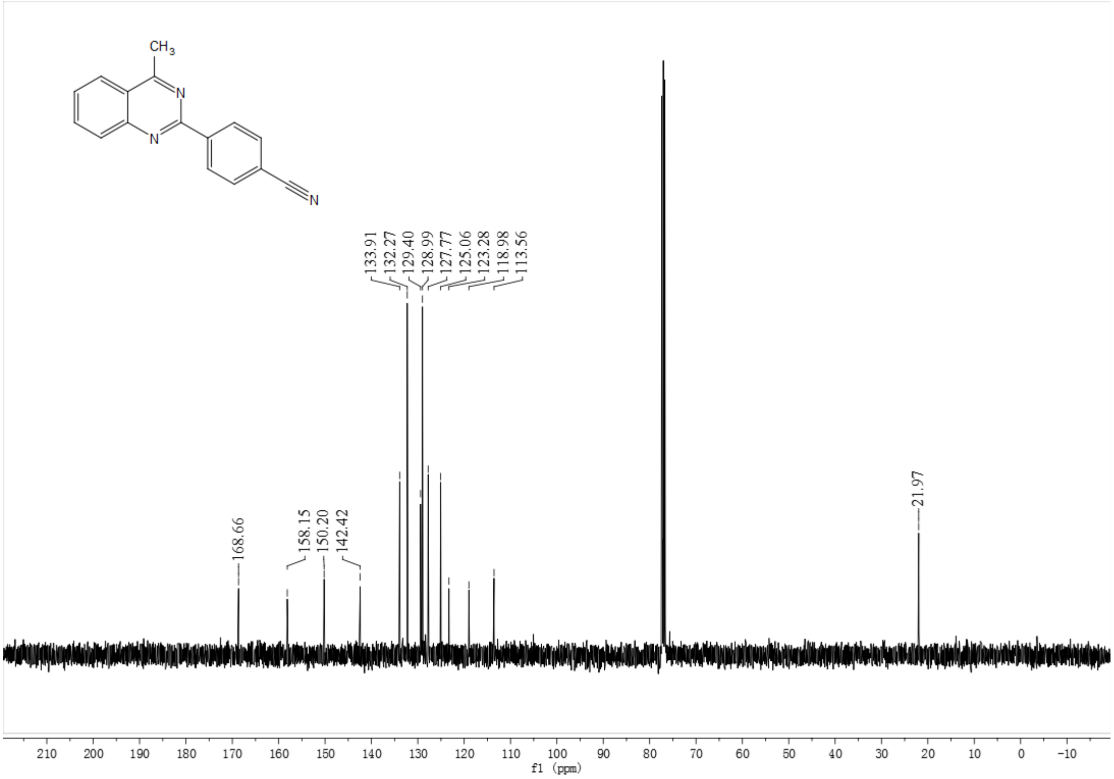

Figure S90. ^{1}H-NMR (400 MHz, $CDCl_3$) spectrum of **3ka**, related to **Scheme 2**.

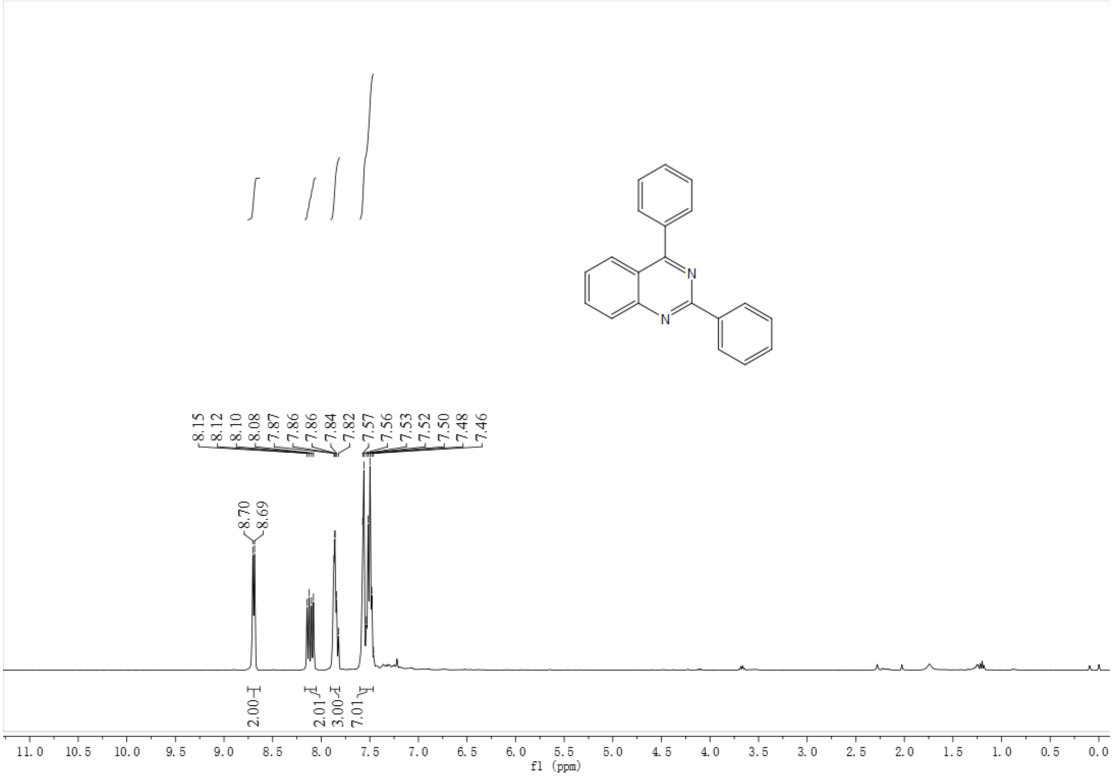

Figure S91. ^{13}C-NMR (100 MHz, $CDCl_3$) spectrum of **3ka**, related to **Scheme 3**.

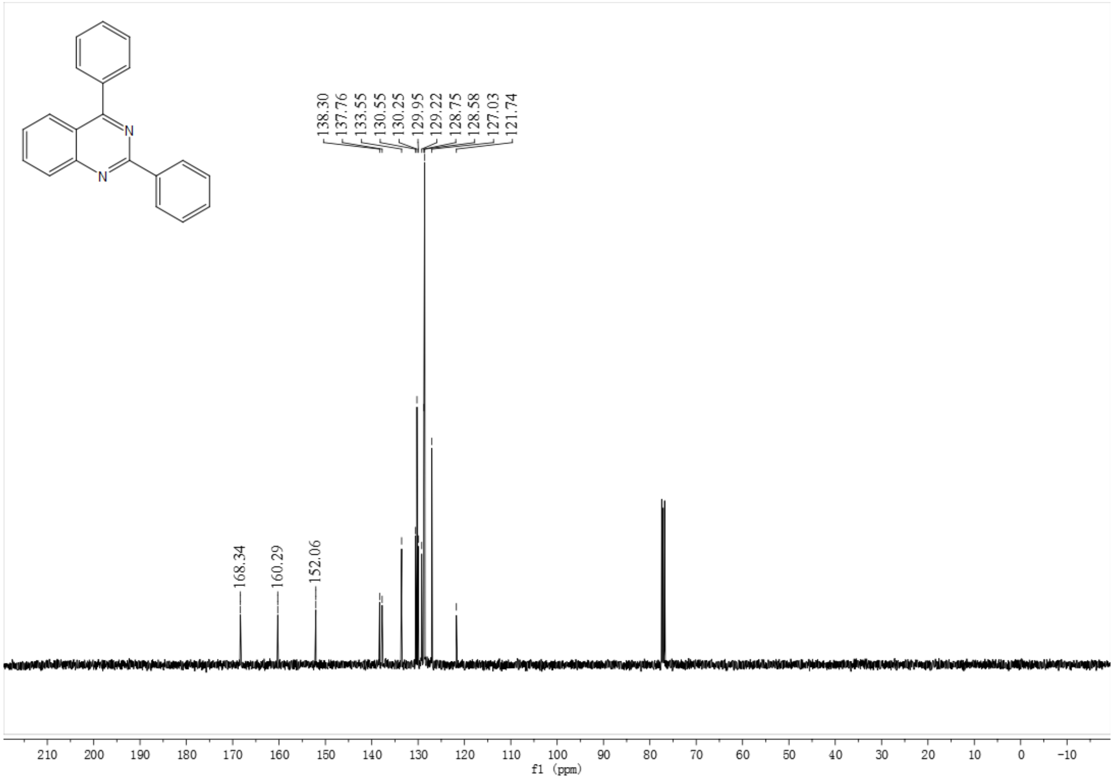

Figure S92. ^{1}H-NMR (400 MHz, $CDCl_3$) spectrum of **3la**, related to **Scheme 2**.

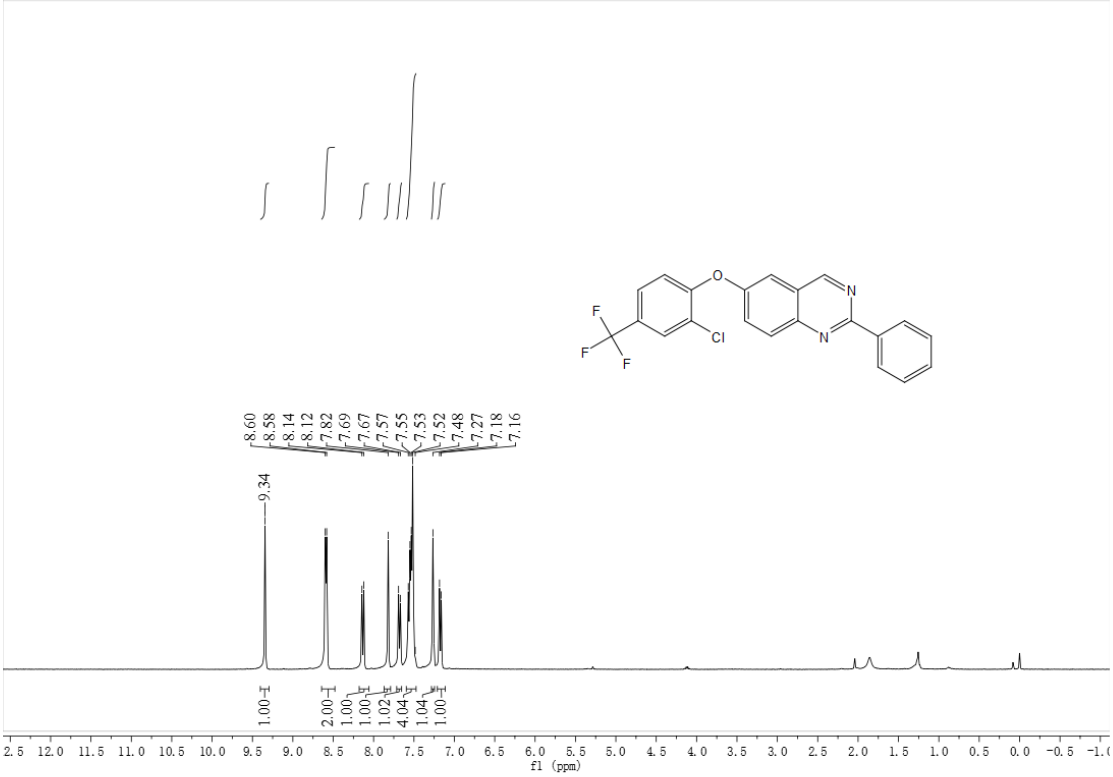

Figure S93. ^{13}C-NMR (100 MHz, $CDCl_3$) spectrum of **3la**, related to **Scheme 3**.

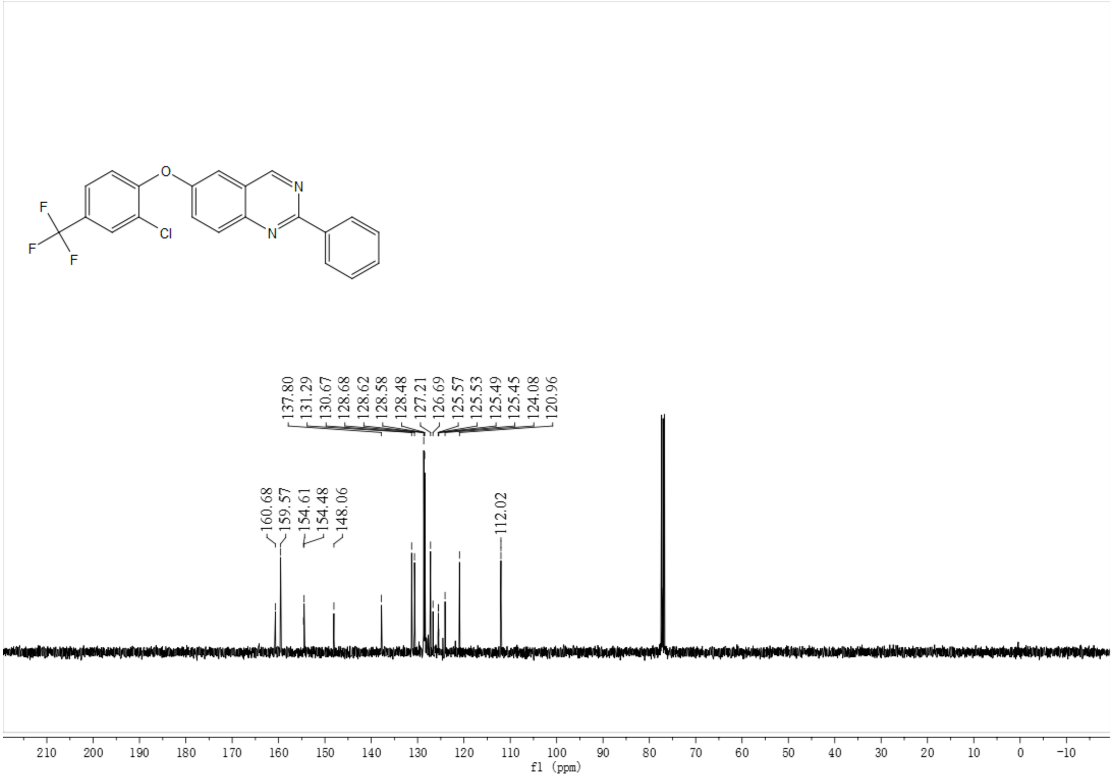

Figure S94. ^{19}F-NMR (100 MHz, $CDCl_3$) spectrum of **3la**, related to **Scheme 3**.

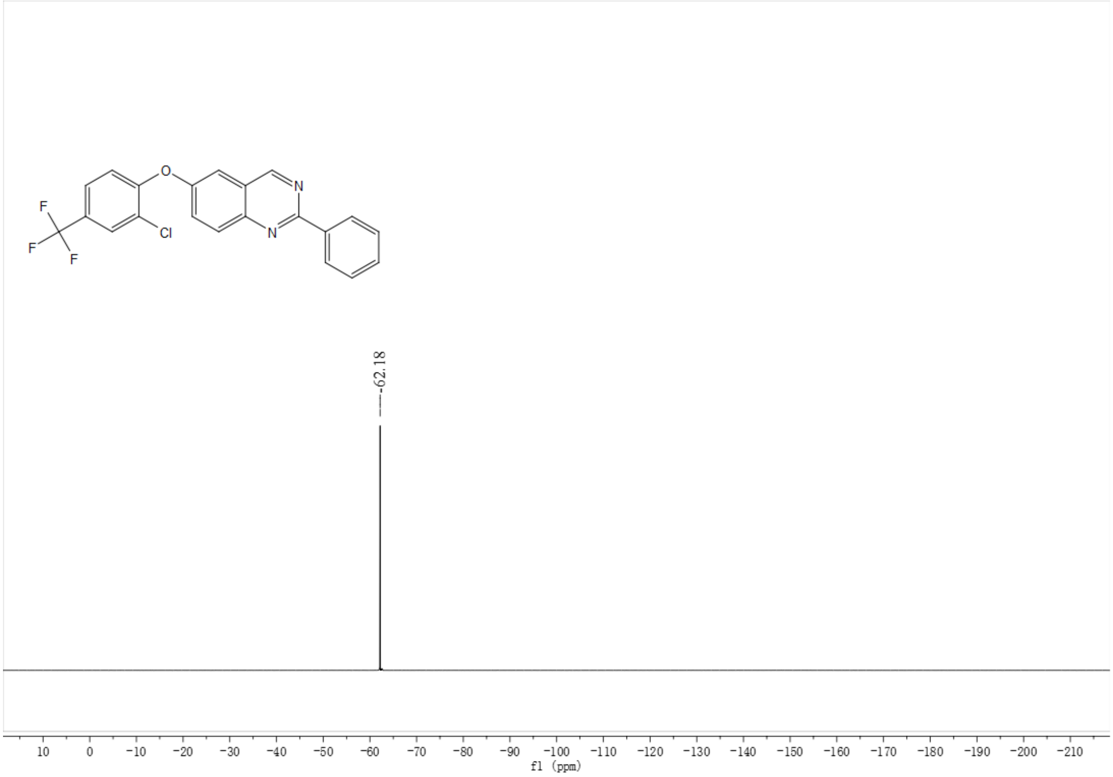

Figure S94. ^{1}H-NMR (400 MHz, $CDCl_3$) spectrum of **4ja**, related to **Scheme 4**.

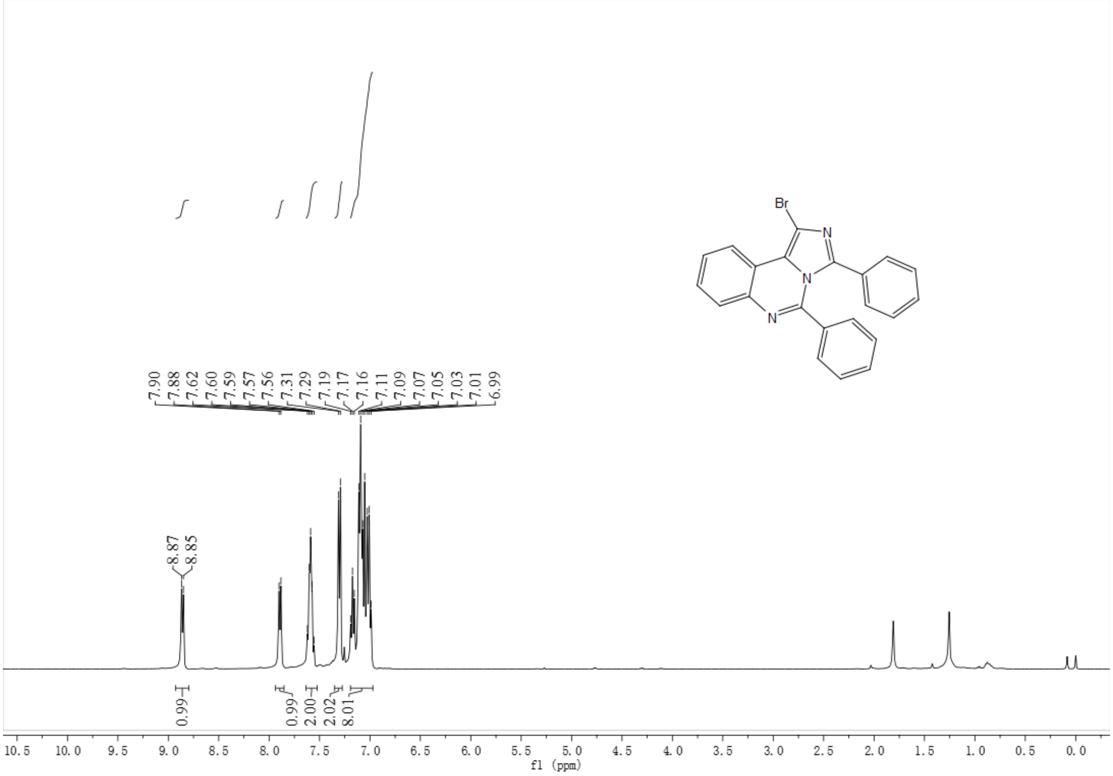

Figure S95. ^{13}C-NMR (100 MHz, $CDCl_3$) spectrum of **4ja**, related to **Scheme 4**.

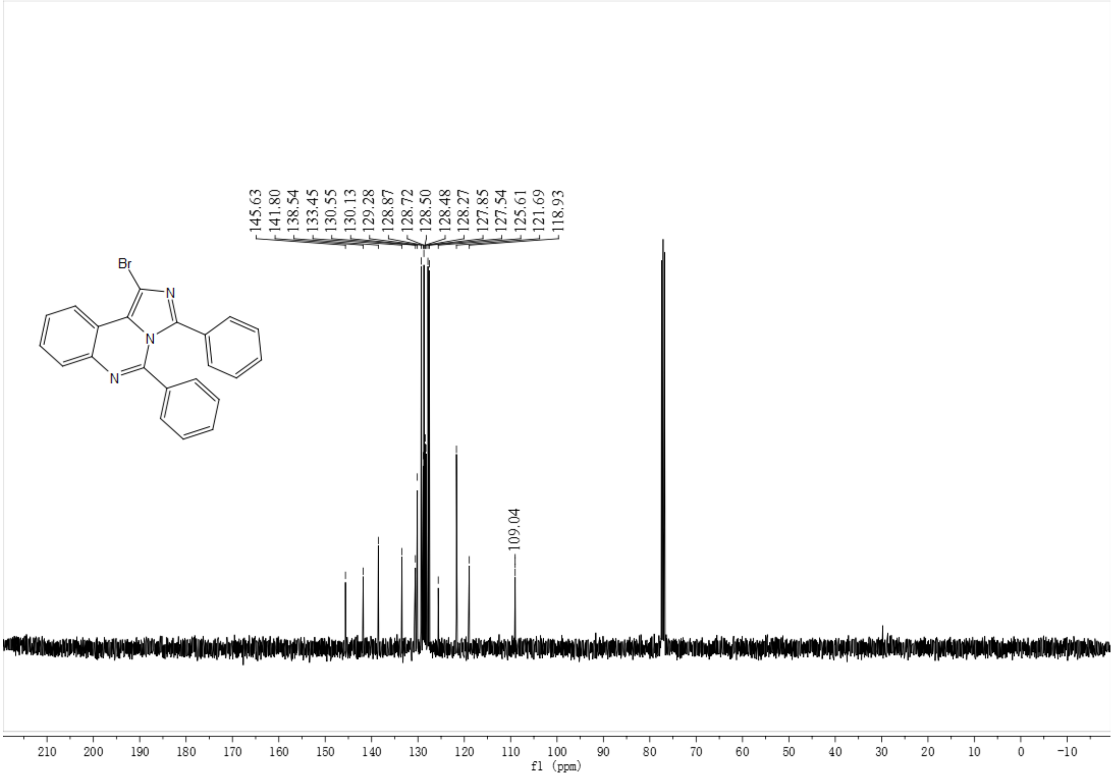

Figure S96. ^{1}H-NMR (400 MHz, $CDCl_3$) spectrum of **5ja**, related to **Scheme 4**.

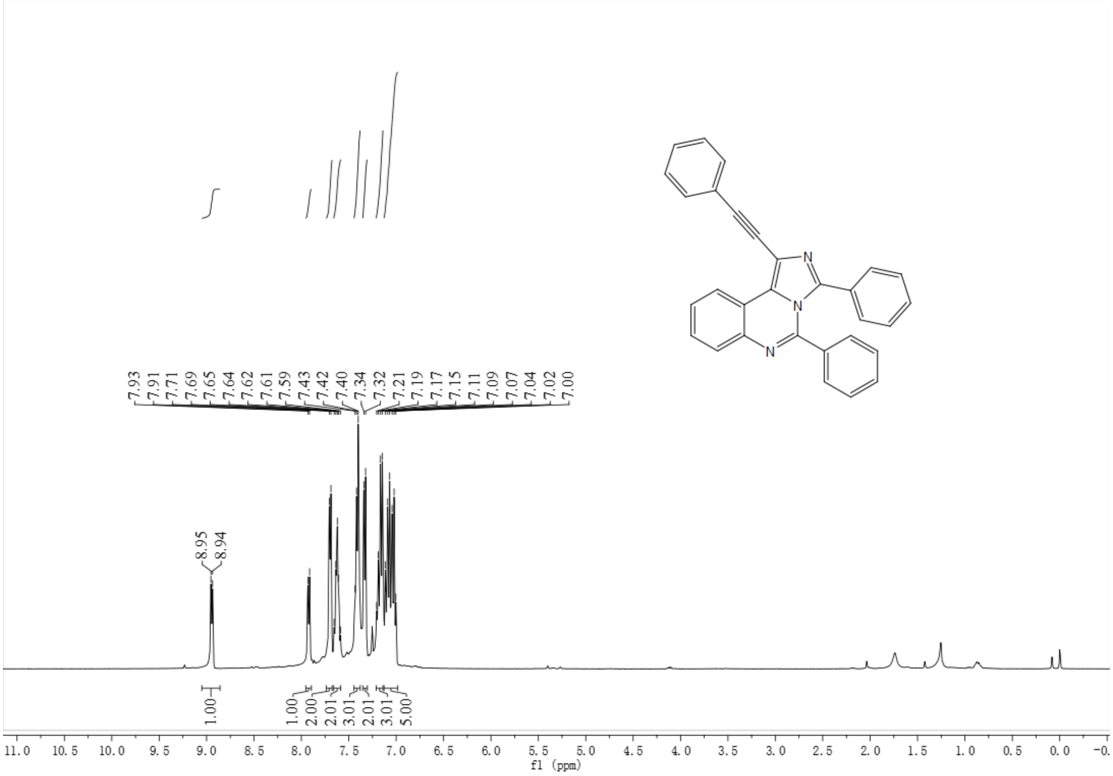

Figure S97. ^{13}C-NMR (100 MHz, $CDCl_3$) spectrum of **5ja**, related to **Scheme 4**.

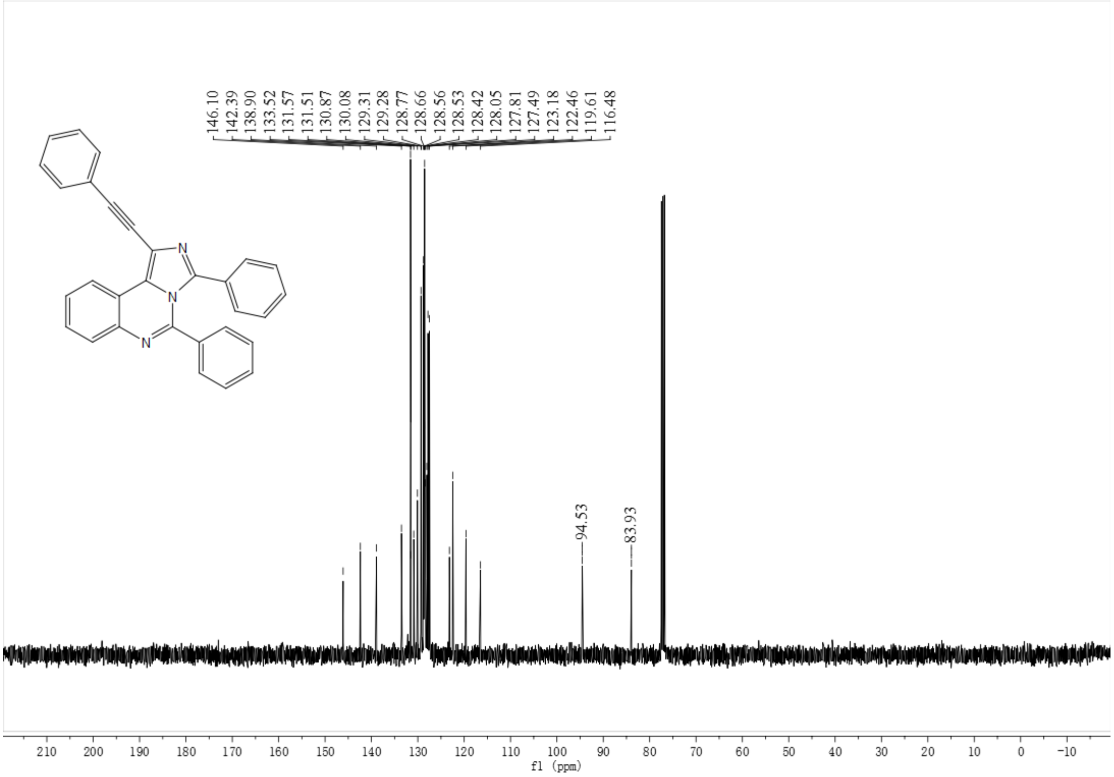

Transparent Methods.

All the obtained products were characterized by melting points (m.p), ^{1}H-NMR, ^{13}C-NMR and infrared spectra (IR). Melting points were measured on an Electrothemal SGW-X4 microscopy digital melting point apparatus and are uncorrected; IR spectra were recorded on a FTLA2000 spectrometer; ^{1}H-NMR and ^{13}C-NMR spectra were obtained on Bruker-400 and referenced to 7.26 ppm for chloroform solvent with TMS as internal standard (0 ppm). Chemical shifts were reported in parts per million (ppm, δ) downfield from tetramethylsilane. Proton coupling patterns are described as singlet (s), doublet (d), triplet (t), multiplet (m); TLC was performed using commercially prepared 100-400 mesh silica gel plates (GF254), and visualization was effected at 254 nm; Unless otherwise stated, all the reagents were purchased from commercial sources (Energy Chemical, J&K Chemic, TCI, Fluka, Acros, SCRC), used without further purification. 1,8-naphthyridines were prepared by the condensation cyclization of 2-aminonicotinaldehyde with ketones in the presence of *t*-BuOK (Chen et al., 2017). 2-Nitrobenzyl alcohol **1l** was prepared by the reduction of Acifluorfen Acid (Rajendran et al., 2015). All calculations were performed for reactants in solution using the solvent model density (SMD) (Marenich et al., 2009) method (solvent = toluene) and employing the Gaussian 09 package (Frisch, M. J. Gaussian 09, Revision C.01; Gaussian, Inc: Wallingford, CT, 2010.). All stationary points were optimized without any constraints at the B3LYP level of theory. (Becke, 1993; Lee et al., 1988; Stephens et al., 1994) Frequency calculations at the same level of theory were also performed to identify all stationary points as minima (zero imaginary frequencies) or transition states (one imaginary frequency), and to calculate the free energies. Intrinsic reaction coordinate calculations were performed to verify the transition-state structures. (Fukui, 1970; Fukui, 1981) The LANL2DZ effective core potential method (Hay et al., 1985; Wadt et al., 1985) with an extra f-polarization function ($\square_{f}$ = 0.938) (Ehlers et al., 1993) was used as the basis set for Ir, while the 6-31G(d) (Krishnan et al., 1980; McLean et al., 1980) basis set was used for all other atoms (C, H, N, O, Na and Cl). To obtain better accuracy, energies of the optimized geometries were recalculated using M06 (Zhao et al., 2005; Zhao et al., 2008; Zhao et al., 2008; Zhao et al., 2009) single point calculations with a larger basis set, employing the LANL2TZ(f) (Roy et al., 2008) basis set for Ir and the 6-311++G** basis set for all other atoms. Empirical D3 dispersion corrections were included for the M06 functional. (Grimme et al., 2010) The final Gibbs free energies reported herein (ΔG_{sol}) represent the M06 single point energies with Gibbs free energy corrections.

Optimization of reaction conditions.

Table S1. Screening of different metal catalysts and ligands. Related to **Table 1**. Related to the first paragraph of “RESULTS AND DISCUSSION” in the main text.

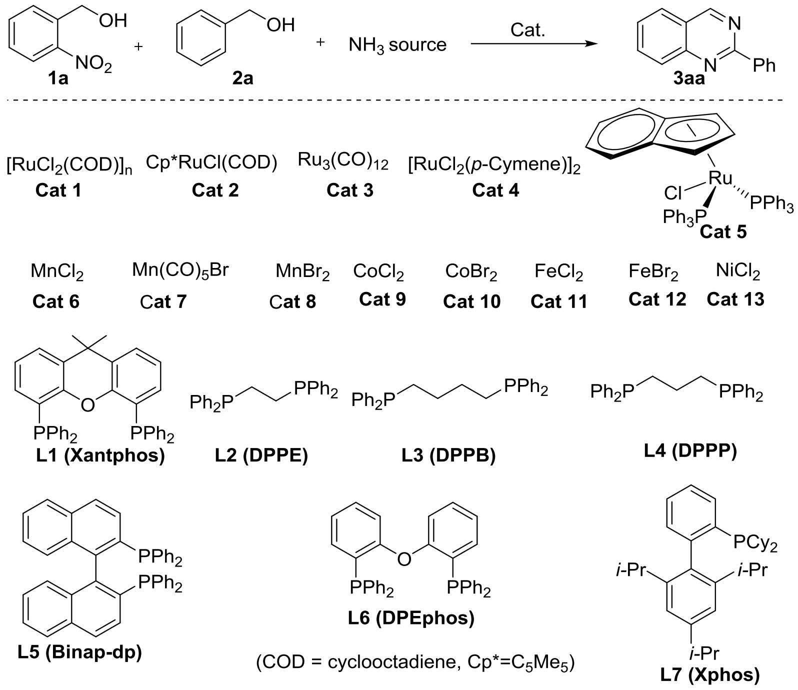

Entry	Catalyst	Ligand	Additive	Solvent	Temperature	N source	Yields of **3aa** a, b
1	**Cat 3**	**L1**	*t*-BuOK	toluene	130	NH_4OAc	5%
2	**Cat 3**	**L2**	*t*-BuOK	toluene	130	NH_4OAc	-
3	**Cat 3**	**L3**	*t*-BuOK	toluene	130	NH_4OAc	trace
4	**Cat 3**	**L4**	*t*-BuOK	toluene	130	NH_4OAc	<5%
5	**Cat 3**	**L5**	*t*-BuOK	toluene	130	NH_4OAc	-
6	**Cat 3**	**L6**	*t*-BuOK	toluene	130	NH_4OAc	<5%
7	**Cat 3**	**L7**	*t*-BuOK	toluene	130	NH_4OAc	-
8	**Cat 1**	**L3**	*t*-BuOK	toluene	130	NH_4OAc	trace
9	**Cat 2**	**L3**	*t*-BuOK	toluene	130	NH_4OAc	trace
10	**Cat 4**	**L3**	*t*-BuOK	toluene	130	NH_4OAc	<5%
11	**Cat 5**	**L3**	*t*-BuOK	toluene	130	NH_4OAc	trace
12	**Cat 6**	**L3**	*t*-BuOK	toluene	130	NH_4OAc	-
13	**Cat 7**	**L3**	*t*-BuOK	toluene	130	NH_4OAc	-
14	**Cat 8**	**L3**	*t*-BuOK	toluene	130	NH_4OAc	-
15	**Cat 9**	**L3**	*t*-BuOK	toluene	130	NH_4OAc	-
16	**Cat 10**	**L3**	*t*-BuOK	toluene	130	NH_4OAc	-
17	**Cat 11**	**L3**	*t*-BuOK	toluene	130	NH_4OAc	-
18	**Cat 12**	**L3**	*t*-BuOK	toluene	130	NH_4OAc	-
19	**Cat 13**	**L3**	*t*-BuOK	toluene	130	NH_4OAc	-
20	**Cat 4**	**L6**	*t*-BuOK	toluene	130	NH_4OAc	<5%
21	**Cat 4**	**-**	*t*-BuOK	toluene	130	NH_4OAc	trace
22	**Cat 3**	**L1**	*t*-BuONa	toluene	130	NH_4OAc	5%
23	**Cat 3**	**L1**	NaOH	toluene	130	NH_4OAc	<5%
24	**Cat 3**	**L1**	KOH	toluene	130	NH_4OAc	<5%
25	**Cat 3**	**L1**	$NaOCH_3$	toluene	130	NH_4OAc	<5%
26	**Cat 3**	**L1**	*t*-BuOK	*p*-xylene	130	NH_4OAc	<5%
27	**Cat 3**	**L1**	*t*-BuOK	chlorobenzene	130	NH_4OAc	<5%
28	**Cat 3**	**L1**	*t*-BuOK	*t*-amyl alcohol	130	NH_4OAc	<5%
29	**Cat 3**	**L1**	*t*-BuOK	DMF	130	NH_4OAc	<5%
30	**Cat 3**	**L1**	*t*-BuOK	toluene	130	NH_4Cl	<5%
31	**Cat 3**	**L1**	*t*-BuOK	toluene	130	$HCOONH_4$	trace

32	**Cat 3**	**L1**	*t*-BuOK	toluene	130	$NH_3{\cdot}H_2O$	trace

[a] The reaction was performed with **1a** (0.5 mmol), **2a** (0.5 mmol), catalyst (1 mol%), ligand (3 mol%), additive (50 mol%), NH_3 sources (1.0 mmol) in solvent (1.5 mL) for 24 h under Ar protection. [b] GC yield by using hexadecane as an internal standard.

Table S2. Optimization of reaction conditions with Ir catalysts. Related to **Table 1**. Related to the first paragraph of "RESULTS AND DISCUSSION" in the main text.

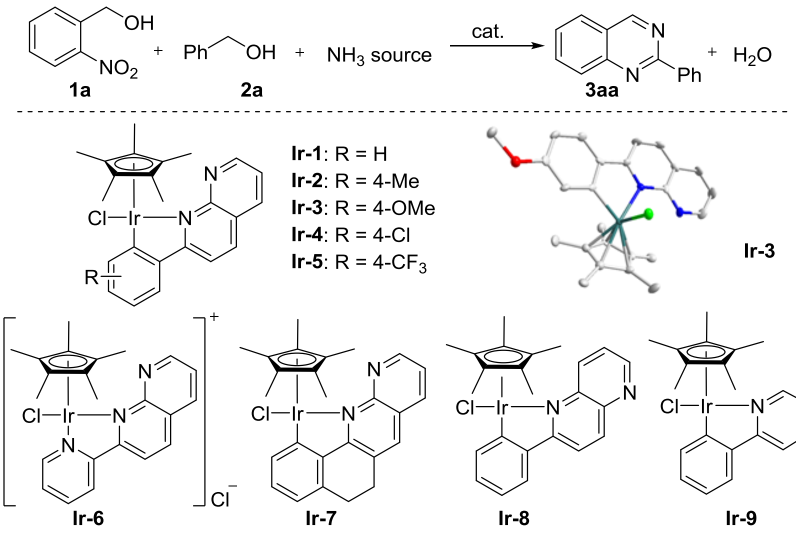

Entry	Catalyst	Additive	Solvent	Temperature	N source	Yields of **3aa**[a, b]
1	$[IrCp^*Cl_2]_2$	*t*-BuOK	toluene	130	NH_4OAc	15
2	$[IrCp^*Cl_2]_2$	*t*-BuOK	*p*-xylene	130	NH_4OAc	trace
3	$[IrCp^*Cl_2]_2$	*t*-BuOK	chlorobenzene	130	NH_4OAc	-
4	$[IrCp^*Cl_2]_2$	*t*-BuOK	*t*-amyl alcohol	130	NH_4OAc	14
5	$[IrCp^*Cl_2]_2$	*t*-BuOK	DMSO	130	NH_4OAc	10
6	$[IrCp^*Cl_2]_2$	*t*-BuOK	DMF	130	NH_4OAc	15
7	$[IrCp^*Cl_2]_2$	*t*-BuOK	1,4-dioxane	130	NH_4OAc	trace
8	$[IrCp^*Cl_2]_2$	*t*-BuONa	toluene	130	NH_4OAc	16
9	$[IrCp^*Cl_2]_2$	NaOH	toluene	130	NH_4OAc	15
10	$[IrCp^*Cl_2]_2$	NaOAc	toluene	130	NH_4OAc	11
11	$[IrCp^*Cl_2]_2$	Cs_2CO_3	toluene	130	NH_4OAc	12
12	$[IrCp^*Cl_2]_2$	NaOMe	toluene	130	NH_4OAc	9
13	$[IrCp^*Cl_2]_2$	-	toluene	130	NH_4OAc	10
14	$[IrCp^*Cl_2]_2$	*t*-BuONa	toluene	120	NH_4OAc	11
15	$[IrCp^*Cl_2]_2$	*t*-BuONa	toluene	140	NH_4OAc	18
16	$[IrCp^*Cl_2]_2$	*t*-BuONa	toluene	150	NH_4OAc	15
17	**Ir-1**	*t*-BuONa	toluene	140	NH_4OAc	72
18	**Ir-2**	*t*-BuONa	toluene	140	NH_4OAc	75
19	**Ir-3**	*t*-BuONa	toluene	140	NH_4OAc	82
20	**Ir-4**	*t*-BuONa	toluene	140	NH_4OAc	61
21	**Ir-5**	*t*-BuONa	toluene	140	NH_4OAc	67
22	**Ir-6**	*t*-BuONa	toluene	140	NH_4OAc	71
23	**Ir-7**	*t*-BuONa	toluene	140	NH_4OAc	68

24	**Ir-8**	*t*-BuONa	toluene	140	NH_4OAc	15
25	**Ir-9**	*t*-BuONa	toluene	140	NH_4OAc	21
26	-	*t*-BuONa	toluene	140	NH_4OAc	-
27	**Ir-3**	*t*-BuONa	toluene	140	NH_4Cl	5
28	**Ir-3**	*t*-BuONa	toluene	140	$HCOONH_4$	trace
29	**Ir-3**	*t*-BuONa	toluene	140	$NH_3{\cdot}H_2O$	trace
30	**Ir-3**	*t*-BuONa	toluene	140	$(NH_4)_2SO_4$	22
31	**Ir-3**	*t*-BuONa	toluene	140	NH_3	88 [c]
32	**Ir-3**	*t*-BuONa	toluene	140	NH_3	81[c, d]
33	**Ir-3**	*t*-BuONa	toluene	140	NH_3	88 [c, e]

[a] The reaction was performed with **1a** (0.5 mmol), **2a** (0.5 mmol), Ir (1 mol%), additive (50 mol%), N sources (1.0 mmol) in solvent (1.5 mL) for 24 h under Ar protection. [b] GC yield by using hexadecane as an internal standard. [c] 4 bar of NH_3. [d] With additive (30 mol%)). [e] With additive (40 mol%).

Typical procedure for the synthesis of complexes Ir-1 – Ir-5, Ir-7, Ir-8 and Ir-9.

Under N_2 atmosphere, $[Cp^*IrCl_2]_2$ (0.2 mmol), NaOAc (0.6 mmol) and 2-substituted 1,8-naphthyridine (0.4 mmol, Chen et al., 2017) and dichloromethane (5 mL) were introduced in a Schlenk tube, successively. Then the Schlenk tube was closed and the resulting mixture was stirred at 60 °C for 12 h. After cooling down to room temperature, the reaction mixture was filtered through celite, eluting with CH_2Cl_2, dried over $MgSO_4$ and filtered. The solvent was evaporated to give a crude solid followed by the addition of 1 mL of diethyl ether with washing for three times.

Typical procedure for the synthesis of complex Ir-6.

Under N_2 atmosphere, $[Cp^*IrCl_2]_2$ (0.2 mmol), and 2-(pyridin-2-yl)-1,8-naphthyridine (0.4 mmol) and dichloromethane (5 mL) were introduced in a Schlenk tube, successively. Then the Schlenk tube was closed and the resulting mixture was stirred at 60 °C for 12 h. After cooling down to room temperature, the reaction mixture was filtered through celite, eluting with CH_2Cl_2, dried over $MgSO_4$ and filtered. The solvent was evaporated to give a crude solid followed by the addition of 1 mL of diethyl ether with washing for three times.

Typical procedure for the synthesis of 3aa.

A vial was charged with (2-nitrophenyl)methanol **1a** (0.5 mmol), **Ir-3** (1 mol %), *t*-BuONa (40 mol %) in succession. The vial was transferred into the autoclave. Once sealed, the autoclave was purged with argon gas, and then was injected with phenylmethanol **2a** (0.5 mmol) in toluene (1.5 mL) under Ar atmosphere. Then the autoclave was aerated with NH_3, pressurized to 4 bar, heated up and kept at 140 °C for 24 h under thorough stirring. After the reaction, the autoclave was cooled to room temperature, depressurized, and the resulting mixture was purified by preparative TCL on silica to get 2-phenylquinazoline **3aa**.

Scheme S1. Substrates employed for the synthesis of quinazolines. Related to **Scheme 2, 3** & **4**. Related to the second and third paragraph of “RESULTS AND DISCUSSION” in the main text.

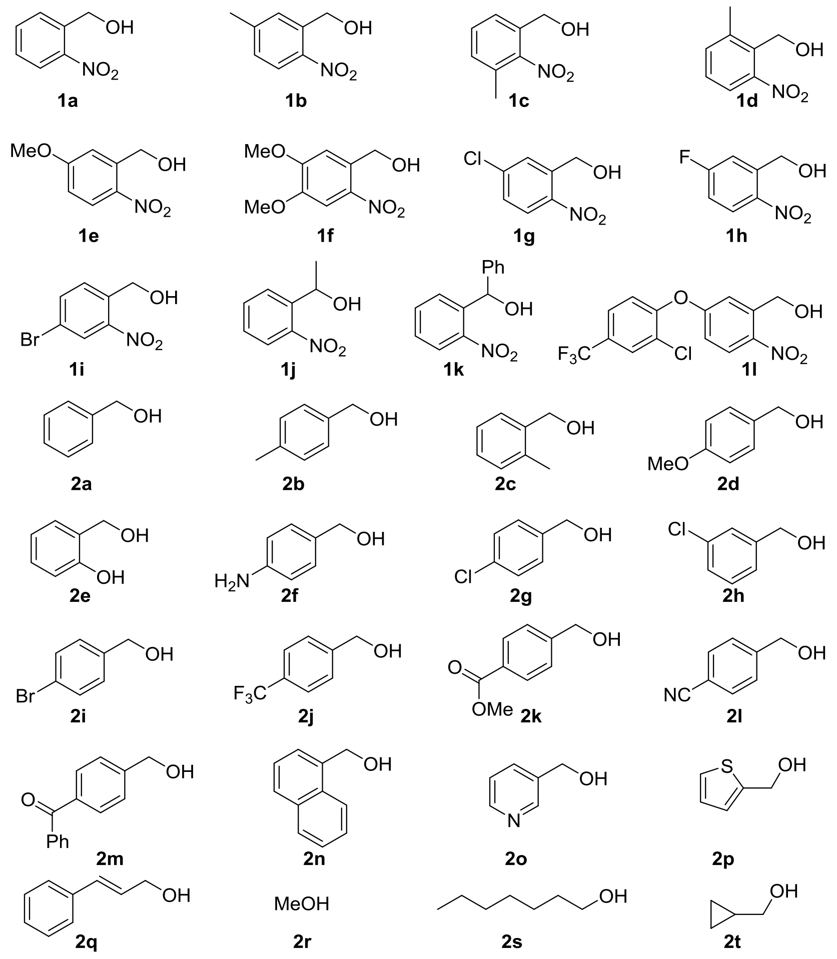

The Control Experiments. Related to the fifth paragraph of "RESULTS AND DISCUSSION" in the main text.

Scheme S2. Control experiments. Related to **Scheme 5**. Related to the fifth paragraph of "RESULTS AND DISCUSSION" in the main text.

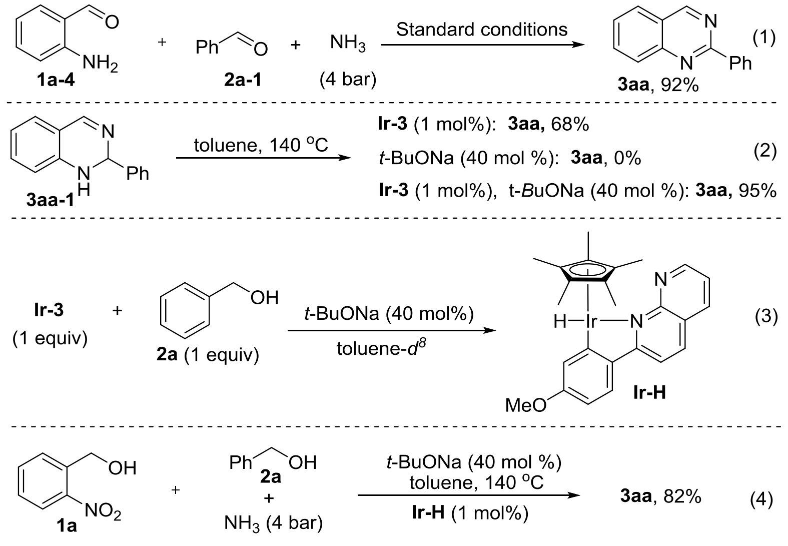

Data S1. The Experimental Procedure for the Time-Concentration Profile. Related to **Scheme 5**. Related to the fifth paragraph of "RESULTS AND DISCUSSION" in the main text.

A vial was charged with (2-nitrophenyl)methanol **1a** (0.5 mmol), **Ir-3** (1 mol %), *t*-BuONa (40 mol %) in succession, which was then transferred into the autoclave. Once sealed, the autoclave was purged with argon gas, and then was injected with phenylmethanol **2a** (0.5 mmol) in toluene (1.5 mL) under Ar atmosphere. Then the autoclave was aerated with NH_3, pressurized to 4 bar, heated up and kept at 140 °C for 0−24 h (1, 2, 4, 8, 12, 16, 20 h) under thorough stirring. After the reaction, the autoclave was cooled to room temperature, depressurized, and added hexadecane (25 mg) as an internal standard. The yield was determined by the GC-MS. For **1a-4**, MS (EI, m/z): 121.08 $[M]^+$; ^1H NMR (400 MHz, $CDCl_3$) δ 9.85 (s, 1H), 7.46 (dd, *J* = 7.8, 1.6 Hz, 1H), 7.29 (t, *J* = 8.3 Hz, 1H), 6.73 (t, *J* = 7.8 Hz, 1H), 6.62 (d, *J* = 8.2 Hz, 1H), 6.05 (s, 2H). For **3aa-1**, MS (EI, m/z): 208.12 $[M]^+$; ^1H NMR (400 MHz, $CDCl_3$) δ 8.15 (s, 1H), 7.50-7.53 (m, 2H), 7.31-7.45 (m, 3H), 7.14-7.21 (m, 2H), 6.70 (s, 1H), 6.56-6.63 (m, 2H), 5.97 (s, 1H).

Data S2. Preparation of Cyclometalated Iridium Hydride **Ir-H.** Related to **Scheme 5**. Related to the fifth paragraph of "RESULTS AND DISCUSSION" in the main text.

According to Xiao's reference method, (Wang et al., 2013) under N_2 atmosphere, **Ir-3** (1 equiv.) and HCOOH/Et_3N (F/T) azeotrope (4 equiv.) in methanol were introduced in a Schlenk tube, successively. The solution was left overnight; Crystals of **Ir-H** were collected after removing the liquid with syringe and washed with MeOH. 42% yield as red crystals; ^1H NMR (400 MHz, Tol) δ 7.20 (s, 1H), 7.05 (s, 1H), 7.01 (d, *J* = 8.4 Hz, 1H), 6.56 (d, *J* = 15.5 Hz, 2H), 6.34 (d, *J* = 7.8 Hz, 1H), 6.29 (d, *J* = 9.1 Hz, 1H), 6.05 (d, *J* = 7.8 Hz, 1H), 3.22 (s, 3H), 1.30 (s, 15H), -15.19 (s, 1H).

Figure S98. ^1H NMR spectrum of complex **Ir-H** in toluene-d^8. Related to **Scheme 5**. Related to the fifth paragraph of "RESULTS AND DISCUSSION" in the main text.

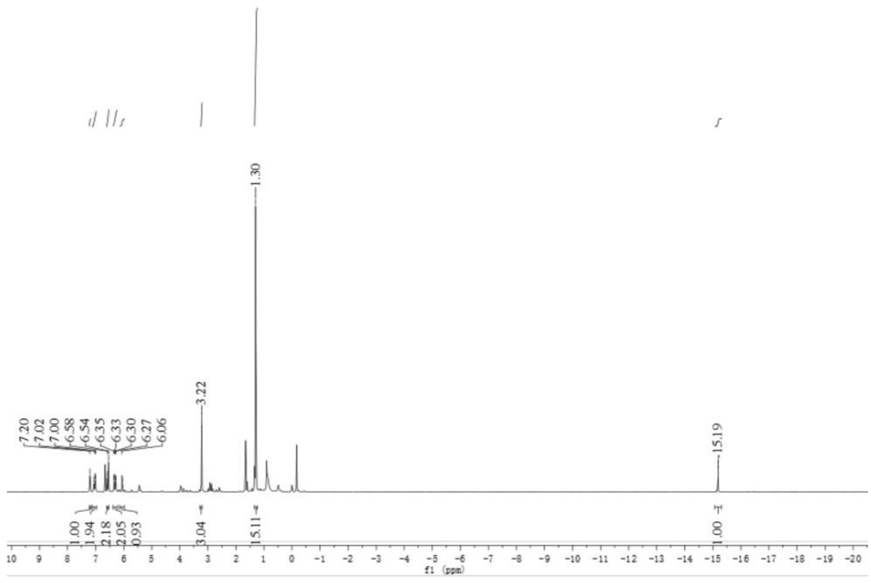

The details of the synthetic utility. Related to the fourth paragraph of “RESULTS AND DISCUSSION” in the main text.

Scheme S3. The synthetic utility of the developed chemistry. Related to **Scheme 4**.

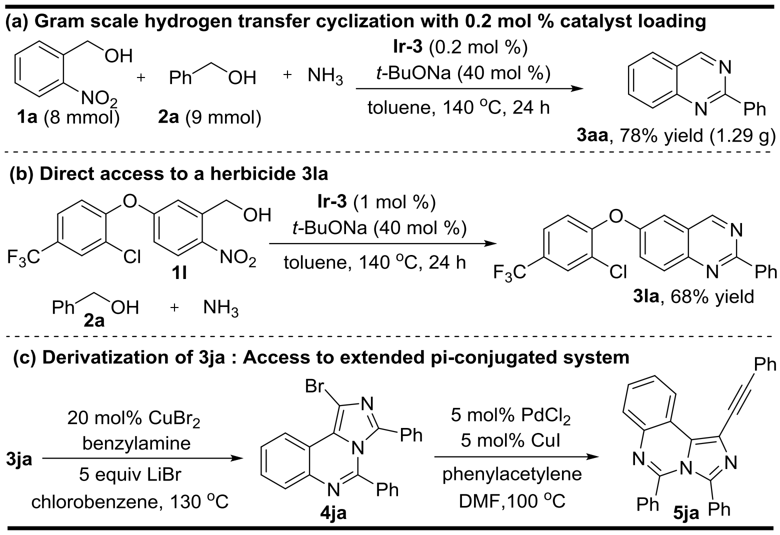

(1) **Preparation of 1l**：To a solution of 5-(2-chloro-4-(trifluoromethyl)phenoxy)-2-nitrobenzoic acid (Acifluorfen Acid, 2 mmol) in THF under N_2 atmosphere, BH_3-THF (1.0 M in THF. 2 mmol, 2 mL) was added at 0 °C over 1 hour. The resulting mixture was allowed to warm to room temperature over 32 hours. After reaction, THF was removed under vacuum; the reaction mixture was quenched with water, and extracted with EtOAc. The combined organic layer was dried with anhydrous sodium sulfate and solvent were removed under reduced pressure to give crude product. Crude product was purified by column chromatography to get **1l**.

(2) **Synthesis of a herbicide 3la**

A vial was charged with **1l** (0.5 mmol), **Ir-3** (1 mol %), *t*-BuONa (40 mol %) in succession. The vial was transferred into the autoclave. Once sealed, the autoclave was purged with argon gas, and then was injected with phenylmethanol **2a** (0.5 mmol) in toluene (1.5 mL) under Ar atmosphere. Then the autoclave was aerated with NH_3, pressurized to 4 bar, heated up and kept at 140 °C for 24 h under thorough stirring. After the reaction, the autoclave was cooled to room temperature, depressurized, and the resulting mixture was purified by preparative TCL on silica to get the product **3la**.

(3) **Synthesis of herbicide 4ja**

The mixture of **3ja** (0.5 mmol), benzylamine (1 mmol), lithium bromide (3 mmol), and $CuBr_2$ (0.1 mmol) in chlorobenzene (3 mL) was stirred at 120 °C for 32 h using an O_2 balloon. After being cooled to room temperature, the resulting mixture was extracted with chloroform, washed with 5% Na_2CO_3 solution, dried with anhydrous sodium sulfate, and then concentrated by removing the solvent under vacuum. Finally, the residue was purified by preparative TLC on silica to give the product **4ja**.

(4) **Synthesis of functionalized multi conjugate N-heteroaromatic 5ja**

Under N_2 atmosphere, **4ja** (0.5 mmol), ethynylbenzene (0.65 mmol), $PdCl_2$ (5 mol %), CuI (20 mol %), PPh_3 (10 mol %), $N(C_2H5)_3$ (1.5 mmol), and DMF (1.0 mL) were introduced in a Schlenk tube, successively. Then the Schlenk tube was closed, and the resulting mixture was stirred at 90 °C for 12 h. After being cooled to room temperature, the resulting mixture was extracted with chloroform, washed with 5% Na_2CO_3 solution, dried with anhydrous sodium

sulfate, and then concentrated by removing the solvent under vacuum. Finally, the residue was purified by preparative TLC on silica to give the product **5ja**.

Scheme S4. MPV-O TH pathway. Related to **Figure 2**. (Values shown are relative free energies in kcal/mol.) Related to the sixth paragraph of “RESULTS AND DISCUSSION” in the main text.

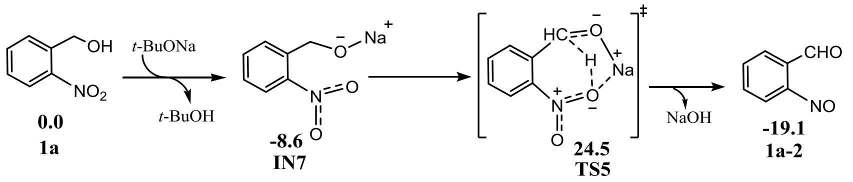

The calculated free-energy profile for the 2nd TH of **1a-2** to **1a-3** is shown in Figure S99. The reaction begins with coordination of **2a** to the Ir center of **Ir-O1** to form intermediate **IN8**, which is an endergonic process. Subsequent O–H deprotonation via four-centered transition-state **TS6** with an energy barrier of 23.3 kcal/mol relative to **Ir-O1** gives Ir–alkoxide intermediate **Ir-O2** and *t*-BuOH. From **Ir-O2**, C–H cleavage via transition state **TS7** gives Ir–benzaldehyde **IN9** with an energy barrier of 27.0 kcal/mol relative to **Ir-O2**. Intermediate **IN9** would then dissociate to give **Ir-H** together with the benzaldehyde. The next step is the TH from **Ir-H** to **1a-2**. We calculated two possible pathways: hydrogen migration to N atom via **TS8′** (blue line) and hydrogen migration to O atom via **TS8″** (red line). The results show that the barrier (24.7 kcal/mol) for hydrogen migration to O atom is higher than that (19.6 kcal/mol) for hydrogen migration to N atom, therefore the hydrogen migration to N atom is kinetically favorable. Previously, it has been shown that such a H-transfer could be facilitated by a transferring shuttle such as water or alcohol proton shuttle. We performed calculations for hydrogen migration to N atom when using benzyl alcohol **2a** as the proton transferring shuttle (black line). The calculated free energy of transition state **TS8** for the hydrogenation assisted by **2a** is -12.2 kcal/mol, which is lower than that of **TS8′**. Therefore, the hydrogen migration to the N atom using **2a** as the proton transferring shuttle constitutes a preferred mode. The relative instability of **TS8** compared with **TS8′** can be attributed to the ring strain associated with the four-membered ring in **TS8′**. **IN10** then isomerizes to the less stable intermediate **IN11** by coordination of **2a** to the Ir center and dissociation of one of the arms of the 1,8-naphthyridyl ligand. The TH is accomplished via transition state **TS9** with an energy barrier of 23.0 kcal/mol relative to **IN10**, which generates the 2-(hydroxyamino)benzaldehyde **1a-3** and complex **Ir-O2**.

Figure S99. Calculated energy profiles for transformation of 2-nitrosobenzaldehyde **1a-2** to 2-(hydroxyamino)benzaldehyde **1a-3**. Values shown are relative free energies in kcal/mol. Related to **Figure 2** & **Figure 3**. Related to the seventh paragraph of “RESULTS AND DISCUSSION” in the main text.

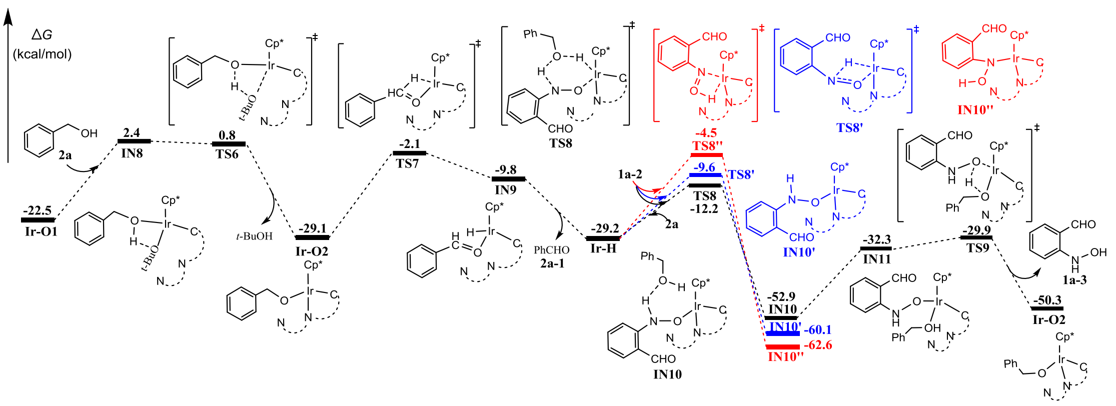

Figure S100. Calculated energy profiles for transformation of 2-(hydroxyamino)benzaldehyde **1a-3** to 2-aminobenzaldehyde. Values shown are relative free energies in kcal/mol. Related to **Figure 2** & **Figure 3**. Related to the seventh paragraph of “RESULTS AND DISCUSSION” in the main text.

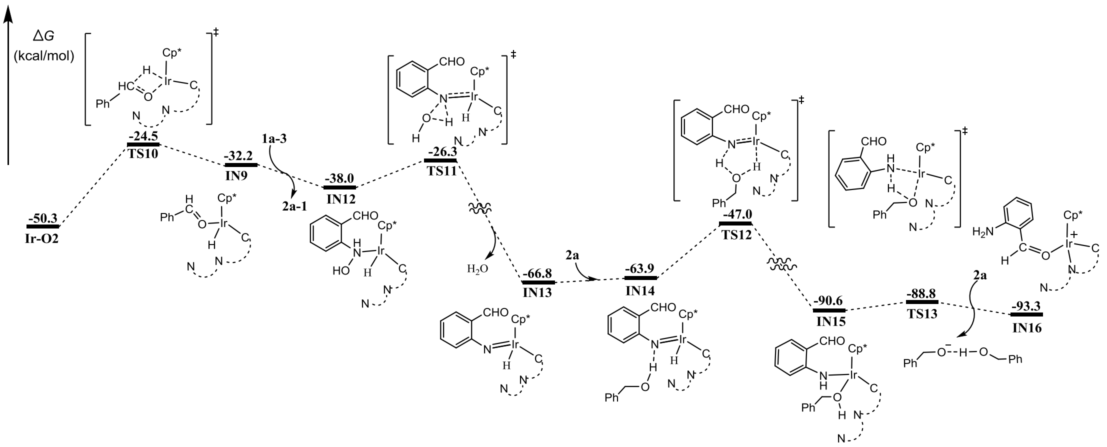

As shown in Figure S100 (3rd TH), **Ir-O2** is transformed to **IN9** through *β*-H elimination. The identified transition state is denoted as **TS10** and the calculated energy barrier is 25.8 kcal/mol. A molecule of **1a-3** enters and then the benzaldehyde dissociates to yield intermediate **IN12**. Subsequently, **IN12** undergoes dehydration by passing transition state **TS11**, affording an Ir-nitrene intermediate **IN13**. Binding of **IN13** with benzyl alcohol **2a** via a hydrogen bond affords intermediate **IN14**. From **IN14**, the TH using **2a** as the proton transferring shuttle takes place via **TS12** to give complex **IN15**, in which **2a** is bound to Ir. The hydrogen of **2a** is transferred to the nitrogen atom via **TS13** with a very small barrier of 1.8 kcal/mol. The iridium 2-aminobenzaldehyde **IN16** is generated with the formation of alcohol anion, which is stabilized by the hydrogen bonding by alcohol **2a**.

Scheme S5. Hydrogen migration to N atom using *t*-BuOH as the proton transferring shuttle. Values shown are relative free energies in kcal/mol. Related to **Figure 2** & **Figure 3**. Related to the seventh paragraph of “RESULTS AND DISCUSSION” in the main text.

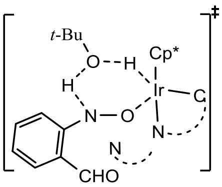

-10.4
TS8-1

Scheme S6. Other possible pathway started from **IN12**. Values shown are relative free energies in kcal/mol. Related to **Figure 2** & **Figure 3**. Related to the seventh paragraph of "RESULTS AND DISCUSSION" in the main text.

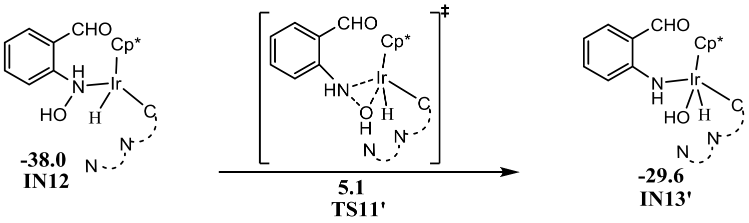

Scheme S7. Other possible pathway started from **IN19**. Values shown are relative free energies in kcal/mol. Related to **Figure 3**. Related to the seventh paragraph of "RESULTS AND DISCUSSION" in the main text.

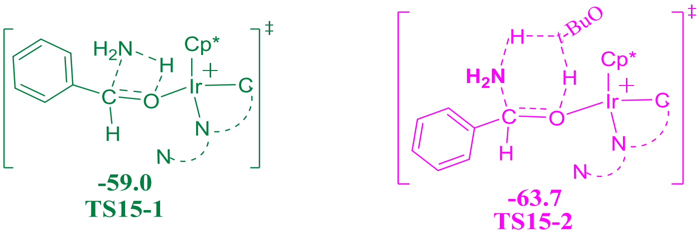

Scheme S8. Other possible pathway started from **IN21**. Values shown are relative free energies in kcal/mol. Related to **Figure 3**. Related to the seventh paragraph of "RESULTS AND DISCUSSION" in the main text.

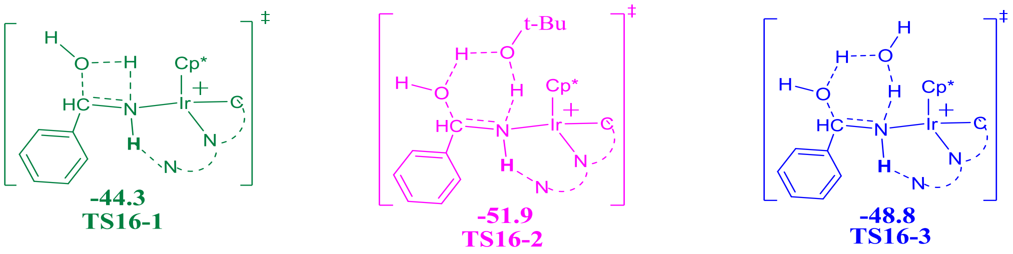

Table S3. Energies (in Hartree) for all TS and intermediates. Related to **Figure 2**, **Figure 3**, **Figure S99**, Figure **S100** and **Scheme S4**, **Scheme S5**, **Scheme S6**, **Scheme S7** & **Scheme S8**.

Geometry	E_0	E	$H_{413.15}$	$G_{413.15}$	$E_{(sol,M06)}$
**Ir-O1**	-1490.293732	-1490.229403	-1490.228095	-1490.402536	-1489.75506
**1a**	-551.134366	-551.117956	-551.116647	-551.187508	-551.0114631
**IN1**	-2041.416957	-2041.333032	-2041.331723	-2041.557112	-2040.745557

**TS1**	-2041.408804	-2041.326030	-2041.324722	-2041.544271	-2040.732445
**IN2**	-1807.908586	-1807.839290	-1807.837982	-1808.028124	-1807.287753
**TS2**	-1807.855907	-1807.786936	-1807.785627	-1807.974158	-1807.243077
**IN3**	-1807.870208	-1807.800571	-1807.799263	-1807.989963	-1807.25635
**Ir-H**	-1807.910263	-1807.839392	-1807.838084	-1808.037770	-1807.29225
**TS3**	-1807.877623	-1807.807900	-1807.806592	-1807.997863	-1807.261206
**IN4**	-1807.907399	-1807.836988	-1807.835680	-1808.027498	-1807.296366
***t*-BuOH**	-233.536268	-233.524255	-233.522947	-233.580384	-233.4931922
**IN5**	-2041.458380	-2041.374111	-2041.372803	-2041.595922	-2040.781565
**TS4**	-2041.404858	-2041.320656	-2041.319347	-2041.539368	-2040.742495
**IN6**	-2041.417579	-2041.331447	-2041.330139	-2041.555450	-2040.759786
**1a-2**	-474.748119	-474.733612	-474.732303	-474.799019	-474.619815
**H_2O**	-76.390125	-76.386179	-76.384871	-76.416321	-76.42743
***t*-BuONa**	-395.263703	-395.249546	-395.248238	-395.314796	-395.2140859
**IN7**	-712.877431	-712.858262	-712.856954	-712.936649	-712.7460679
**TS5**	-712.826173	-712.807984	-712.806675	-712.881912	-712.6933673
**NaOH**	-238.108646	-238.103584	-238.102276	-238.142390	-238.1421522
**2a**	-346.636496	-346.623523	-346.622215	-346.686686	-346.5400135
**IN8**	-1836.911027	-1836.831642	-1836.830334	-1837.042138	-1836.255335
**TS6**	-1836.913327	-1836.834518	-1836.833210	-1837.043820	-1836.257927
**Ir-O2**	-1603.408090	-1603.342829	-1603.341521	-1603.522468	-1602.81245
**TS7**	-1603.362712	-1603.297952	-1603.296643	-1603.474749	-1602.769385
**IN9**	-1603.377738	-1603.312241	-1603.310933	-1603.491010	-1602.78172
**2a-1**	-345.466000	-345.454515	-345.453207	-345.511975	-345.3587192
**TS8**	-2079.312101	-2079.229325	-2079.228017	-2079.448190	-2078.589596
**IN10**	-2079.384394	-2079.300413	-2079.299104	-2079.524868	-2078.654402
**TS8'**	-1732.657052	-1732.588403	-1732.587095	-1732.777218	-1732.045384
**IN10'**	-1732.735862	-1732.667536	-1732.666227	-1732.851169	-1732.126231
**TS8''**	-1732.641503	-1732.573418	-1732.572109	-1732.757193	-1732.037325
**IN10''**	-1732.728208	-1732.660113	-1732.658804	-1732.842567	-1732.129858
**TS8-1**	-1966.204210	-1966.122334	-1966.121026	-1966.336549	-1965.540029
**IN11**	-2079.347461	-2079.263782	-2079.262473	-2079.487346	-2078.621626
**TS9**	-2079.344560	-2079.261430	-2079.260122	-2079.482288	-2078.617761
**1a-3**	-475.953316	-475.937391	-475.936082	-476.005475	-475.8397945
**TS10**	-1603.362712	-1603.297952	-1603.296643	-1603.474749	-1602.769385
**IN12**	-1733.873484	-1733.803692	-1733.802383	-1733.991017	-1733.272035
**TS11**	-1733.844795	-1733.773839	-1733.772530	-1733.966068	-1733.253426
**IN13**	-1733.909215	-1733.837124	-1733.835815	-1734.033472	-1733.317896
**TS11'**	-1733.810514	-1733.740262	-1733.738954	-1733.929844	-1733.203408
**IN13'**	-1733.894687	-1733.824729	-1733.823420	-1734.012897	-1733.301025
**IN14**	-2004.142375	-2004.060198	-2004.058890	-2004.280108	-2003.425822
**TS12**	-2004.126634	-2004.045630	-2004.044321	-2004.261454	-2003.398905
**IN15**	-2004.184670	-2004.102997	-2004.101689	-2004.320682	-2003.468364
**TS13**	-2004.182515	-2004.101566	-2004.100258	-2004.317473	-2003.46559

**IN16**	-2350.855816	-2350.758697	-2350.757388	-2351.016560	-2350.016557
**IN17**	-2295.510124	-2295.415682	-2295.414374	-2295.666119	-2294.671788
**1a-4**	-400.801794	-400.787647	-400.786339	-400.850827	-400.6899132
**NH_3**	-56.514029	-56.509968	-56.508659	-56.540764	-56.5317547
**TS14**	-1659.203233	-1659.135024	-1659.133716	-1659.319764	-1658.58771
**IN18**	-1659.201798	-1659.133525	-1659.132216	-1659.318261	-1658.58754
**TS14'**	-1714.532549	-1714.463006	-1714.461698	-1714.648483	-1713.91008
**IN18'**	-1714.542462	-1714.473759	-1714.472451	-1714.656625	-1713.918227
**IN19**	-1715.733012	-1715.659948	-1715.658640	-1715.857467	-1715.118292
**IN19'**	-1771.060274	-1770.986486	-1770.985177	-1771.180845	-1770.452496
**TS15**	-1715.723377	-1715.652685	-1715.651377	-1715.840531	-1715.107175
**IN20**	-1715.728350	-1715.655572	-1715.654264	-1715.849553	-1715.120182
**TS15'**	-1771.062498	-1770.988987	-1770.987679	-1771.183810	-1770.443594
**IN20'**	-1771.064440	-1770.989750	-1770.988441	-1771.186668	-1770.450352
**TS15-1**	-1659.167866	-1659.100380	-1659.099072	-1659.284664	-1658.557134
**TS15-2**	-1892.729659	-1892.649609	-1892.648301	-1892.857627	-1892.057864
**IN21**	-1715.747857	-1715.675119	-1715.673811	-1715.868374	-1715.139997
**IN21'**	-1771.083117	-1771.008716	-1771.007408	-1771.204529	-1770.468709
**TS16**	-1715.703685	-1715.631683	-1715.630375	-1715.823540	-1715.095749
**IN22**	-1715.747576	-1715.673008	-1715.671699	-1715.873345	-1715.139803
**TS16'**	-1771.042429	-1770.968860	-1770.967551	-1771.161731	-1770.424036
**IN22'**	-1771.091888	-1771.015464	-1771.014156	-1771.217697	-1770.476703
**IN21''**	-1715.744916	-1715.672216	-1715.670908	-1715.866802	-1715.13918
**TS16''**	-1715.701957	-1715.630330	-1715.629021	-1715.820097	-1715.087871
**IN22''**	-1715.749138	-1715.673841	-1715.672532	-1715.877915	-1715.143996
**TS16-1**	-1659.137223	-1659.069786	-1659.068478	-1659.250792	-1658.533044
**TS16-2**	-1892.704617	-1892.624842	-1892.623534	-1892.832588	-1892.038956
**TS16-3**	-1735.561694	-1735.491091	-1735.489783	-1735.678620	-1734.968226
**2a-2**	-325.571476	-325.559576	-325.558268	-325.618207	-325.4553086
**2a-3**	-380.907260	-380.893214	-380.891906	-380.955684	-380.7845477

E_0 = Sum of electronic and zero-point Energies by B3LYP in solvent

E = Sum of electronic and thermal Energies by B3LYP in solvent

$H_{413.15}$ = Sum of electronic and thermal Enthalpies by B3LYP in solvent

$G_{413.15}$ = Sum of electronic and thermal Free Energies by B3LYP in solvent

$E_{(sol, M06)}$ = Single point energies calculated by M06 in solvent

Calculated imaginary frequencies of all transition states species

Table S4. Calculated imaginary frequencies of all transition states species for substrate. Related to **Figure 2**, **Figure 3**, **Figure S99**, Figure **S100** and **Scheme S4**, **Scheme S5**, **Scheme S6**, **Scheme S7** & **Scheme S8**.

Species	Frequency
**TS1**	-584.09
**TS2**	-743.09
**TS3**	-425.57

**TS4**	-715.20
**TS5**	-1746.69
**TS6**	-737.34
**TS7**	-686.33
**TS8**	-901.67
**TS8'**	-860.06
**TS8''**	-679.02
**TS8-1**	-860.46
**TS9**	-757.76
**TS10**	-686.33
**TS11**	-593.96
**TS11'**	-627.49
**TS12**	-563.41
**TS13**	-1148.07
**TS14**	-144.35
**TS14'**	-140.97
**TS15**	-57.07
**TS15'**	-316.98
**TS15-1**	-1654.38
**TS15-2**	-829.31
**TS16**	-697.59
**TS16'**	-281.04
**TS16''**	-716.03
**TS16-1**	-1584.06
**TS16-2**	-832.94
**TS16-3**	-1062.09

Crystallographic data of complex Ir-3.

Figure S101. Molecular structure of **Ir-3** is displayed with thermal ellipsoids set at 50% probability (Hydrogen atoms are omitted for clarity. CCDC: 1848110). Related to **Table 1**.

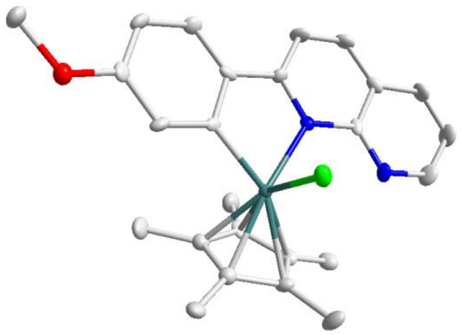

Table S5. Crystal data and structure refinement for **Ir-3**. Related to **Table 1**.

Identification code	**Ir-3**
Empirical formula	$C_{25}H_{26}ClIrN_2O$
Formula weight	598.13

Temperature/K	100.00(10)
Crystal system	orthorhombic
Space group	$P2_12_12_1$
a/Å	9.0070(4)
b/Å	14.7851(8)
c/Å	16.1262(6)
α/°	90
β/°	90
γ/°	90
Volume/Å^3	2147.52(17)
Z	4
ρ_{calc}g/cm^3	1.850
μ/mm^{-1}	6.361
F(000)	1168.0
Crystal size/mm^3	0.13 × 0.12 × 0.11
Radiation	MoKα (λ = 0.71073)
2Θ range for data collection/°	5.052 to 49.994
Index ranges	-10 ≤ h ≤ 9, -12 ≤ k ≤ 17, -19 ≤ l ≤ 15
Reflections collected	14026
Independent reflections	3783 [R_{int} = 0.0488, R_{sigma} = 0.0487]
Data/restraints/parameters	3783/12/277
Goodness-of-fit on F^2	1.033
Final R indexes [I>=2σ (I)]	R_1 = 0.0294, wR_2 = 0.0519
Final R indexes [all data]	R_1 = 0.0356, wR_2 = 0.0545
Largest diff. peak/hole / e Å^{-3}	1.03/-0.77
Flack parameter	-0.027(7)

Table S6. Fractional Atomic Coordinates (×10^4) and Equivalent Isotropic Displacement Parameters (Å^2×10^3) for **Ir-3**. U_{eq} is defined as 1/3 of of the trace of the orthogonalised U_{IJ} tensor. Related to **Table 1**.

**Atom**	***x***	***y***	***z***	**U(eq)**
C(1)	671(10)	6964(7)	59(6)	25(2)
C(2)	698(10)	7845(8)	-244(6)	29(3)
C(3)	1351(10)	8496(7)	223(6)	24(2)
C(4)	2021(9)	8266(6)	992(5)	18(2)
C(5)	1950(8)	7340(6)	1227(5)	14(2)
C(6)	2747(10)	8882(6)	1519(5)	23(2)
C(7)	3433(9)	8583(6)	2212(5)	20(2)
C(8)	3393(7)	7652(5)	2427(6)	15.3(18)
C(9)	4163(9)	7236(6)	3116(5)	16(2)
C(10)	5133(9)	7695(7)	3643(5)	18(2)

C(11)	5935(9)	7244(7)	4236(5)	20(2)
C(12)	5797(9)	6299(7)	4293(5)	18(2)
C(13)	4798(8)	5843(7)	3793(5)	16(2)
C(14)	3948(8)	6291(6)	3206(5)	11.5(19)
C(15)	7620(10)	6218(6)	5376(5)	24(2)
C(16)	192(9)	5757(7)	2999(5)	19(2)
C(17)	1123(9)	5157(7)	3447(6)	20(2)
C(18)	1600(10)	4445(6)	2885(5)	20(2)
C(19)	824(10)	4601(6)	2111(6)	20(2)
C(20)	-36(9)	5378(6)	2185(6)	20(2)
C(21)	-569(11)	6583(7)	3324(6)	30(3)
C(22)	1490(11)	5208(8)	4362(6)	31(3)
C(23)	2466(10)	3611(6)	3116(5)	24(2)
C(24)	994(11)	4030(7)	1348(6)	27(3)
C(25)	-1211(9)	5728(7)	1591(6)	25(2)
Cl(1)	4379(2)	5281.0(16)	1547.5(13)	18.9(5)
Ir(1)	2385.5(3)	5739.5(2)	2458.3(2)	12.78(10)
N(1)	1296(7)	6697(5)	765(4)	17.2(18)
N(2)	2595(7)	7057(4)	1966(4)	12.4(15)
O(1)	6620(6)	5772(5)	4823(3)	20.6(14)

Table S7. Anisotropic Displacement Parameters (Å^2×10^3) for **Ir-3**. The Anisotropic displacement factor exponent takes the form: $-2\pi^2[h^2a^{*2}U_{11}+2hka^*b^*U_{12}+\ldots]$. Related to **Table 1**.

**Atom**	**U_{11}**	**U_{22}**	**U_{33}**	**U_{23}**	**U_{13}**	**U_{12}**
C(1)	15(5)	35(7)	25(6)	-4(5)	-8(4)	6(4)
C(2)	31(6)	36(8)	20(6)	5(5)	-10(4)	6(5)
C(3)	29(6)	21(7)	21(6)	10(5)	-2(4)	3(5)
C(4)	15(5)	18(6)	21(5)	7(4)	5(3)	4(4)
C(5)	6(4)	25(6)	12(5)	3(4)	3(3)	3(4)
C(6)	22(5)	14(5)	31(5)	4(4)	2(4)	-1(4)
C(7)	21(5)	9(5)	31(6)	1(4)	-4(4)	1(4)
C(8)	15(4)	16(5)	16(5)	-4(5)	1(4)	-1(3)
C(9)	19(5)	13(6)	16(5)	0(4)	9(4)	-3(4)
C(10)	16(5)	18(6)	19(5)	-5(4)	3(4)	2(4)
C(11)	15(5)	29(7)	16(5)	-7(5)	-1(4)	-6(4)
C(12)	14(5)	29(7)	11(5)	3(4)	5(3)	-1(4)
C(13)	11(4)	18(6)	19(5)	-5(4)	0(3)	-4(4)
C(14)	9(4)	14(5)	11(5)	-4(4)	5(3)	-1(4)
C(15)	23(5)	28(6)	21(5)	1(4)	-10(4)	-4(5)

C(16)	11(5)	25(6)	20(5)	7(5)	9(3)	3(5)
C(17)	19(2)	20(2)	20(2)	0.7(13)	1.0(13)	-1.3(13)
C(18)	20(3)	19(3)	19(3)	0.4(13)	0.2(13)	-1.3(13)
C(19)	22(5)	15(6)	24(5)	0(4)	-6(4)	-4(4)
C(20)	10(5)	23(6)	27(6)	4(4)	1(3)	1(4)
C(21)	36(6)	33(7)	23(6)	-3(5)	4(4)	0(5)
C(22)	37(6)	42(7)	14(5)	3(5)	4(4)	-9(5)
C(23)	33(6)	19(5)	20(5)	3(4)	0(5)	-11(5)
C(24)	32(6)	19(7)	30(6)	-6(5)	-16(4)	2(4)
C(25)	17(5)	26(6)	32(6)	4(5)	0(4)	0(5)
Cl(1)	21.8(12)	19.1(14)	15.7(12)	0.3(10)	1.6(9)	4.2(10)
Ir(1)	14.04(16)	12.03(17)	12.28(16)	-0.10(17)	-0.40(18)	-0.51(13)
N(1)	18(4)	20(5)	14(4)	1(4)	-2(3)	7(3)
N(2)	8(4)	15(4)	14(3)	2(3)	2(3)	2(3)
O(1)	19(3)	23(4)	20(3)	4(3)	-4(2)	1(3)

Table S8. Bond Lengths for **Ir-3**. Related to **Table 1**.

Atom	Atom	Length/Å	Atom	Atom	Length/Å
C(1)	C(2)	1.391(14)	C(14)	Ir(1)	2.024(8)
C(1)	N(1)	1.331(11)	C(15)	O(1)	1.429(9)
C(2)	C(3)	1.356(14)	C(16)	C(17)	1.418(12)
C(3)	C(4)	1.420(12)	C(16)	C(20)	1.443(12)
C(4)	C(5)	1.422(12)	C(16)	C(21)	1.494(13)
C(4)	C(6)	1.406(12)	C(16)	Ir(1)	2.160(8)
C(5)	N(1)	1.343(11)	C(17)	C(18)	1.454(13)
C(5)	N(2)	1.391(10)	C(17)	C(22)	1.514(13)
C(6)	C(7)	1.352(12)	C(17)	Ir(1)	2.139(9)
C(7)	C(8)	1.420(12)	C(18)	C(19)	1.450(12)
C(8)	C(9)	1.447(12)	C(18)	C(23)	1.505(12)
C(8)	N(2)	1.357(10)	C(18)	Ir(1)	2.154(9)
C(9)	C(10)	1.396(12)	C(19)	C(20)	1.391(13)
C(9)	C(14)	1.418(13)	C(19)	C(24)	1.499(12)
C(10)	C(11)	1.372(12)	C(19)	Ir(1)	2.263(9)
C(11)	C(12)	1.406(13)	C(20)	C(25)	1.517(12)
C(12)	C(13)	1.383(12)	C(20)	Ir(1)	2.288(9)
C(12)	O(1)	1.373(10)	Cl(1)	Ir(1)	2.417(2)
C(13)	C(14)	1.386(12)	Ir(1)	N(2)	2.112(6)

Table S9. Bond Angles for **Ir-3**. Related to **Table 1**.

Atom	Atom	Atom	Angle/˚	Atom	Atom	Atom	Angle/˚
N(1)	C(1)	C(2)	124.8(9)	C(18)	C(19)	Ir(1)	66.8(5)
C(3)	C(2)	C(1)	118.5(9)	C(20)	C(19)	C(18)	109.1(8)
C(2)	C(3)	C(4)	120.0(10)	C(20)	C(19)	C(24)	126.3(8)
C(3)	C(4)	C(5)	116.3(9)	C(20)	C(19)	Ir(1)	73.2(5)
C(6)	C(4)	C(3)	124.8(9)	C(24)	C(19)	Ir(1)	123.9(6)
C(6)	C(4)	C(5)	118.9(8)	C(16)	C(20)	C(25)	122.8(9)
N(1)	C(5)	C(4)	123.6(8)	C(16)	C(20)	Ir(1)	66.3(5)
N(1)	C(5)	N(2)	116.4(8)	C(19)	C(20)	C(16)	108.6(8)
N(2)	C(5)	C(4)	119.9(8)	C(19)	C(20)	C(25)	128.0(9)
C(7)	C(6)	C(4)	120.1(9)	C(19)	C(20)	Ir(1)	71.2(5)
C(6)	C(7)	C(8)	120.4(9)	C(25)	C(20)	Ir(1)	135.0(6)
C(7)	C(8)	C(9)	125.9(8)	C(14)	Ir(1)	C(16)	113.0(3)
N(2)	C(8)	C(7)	120.6(8)	C(14)	Ir(1)	C(17)	95.0(3)
N(2)	C(8)	C(9)	113.5(7)	C(14)	Ir(1)	C(18)	113.3(3)
C(10)	C(9)	C(8)	124.1(8)	C(14)	Ir(1)	C(19)	151.5(3)
C(10)	C(9)	C(14)	120.2(8)	C(14)	Ir(1)	C(20)	150.6(3)
C(14)	C(9)	C(8)	115.6(8)	C(14)	Ir(1)	Cl(1)	87.6(2)
C(11)	C(10)	C(9)	121.2(9)	C(14)	Ir(1)	N(2)	77.9(3)
C(10)	C(11)	C(12)	118.8(8)	C(16)	Ir(1)	C(19)	62.7(4)
C(13)	C(12)	C(11)	120.2(8)	C(16)	Ir(1)	C(20)	37.7(3)
O(1)	C(12)	C(11)	123.8(8)	C(16)	Ir(1)	Cl(1)	158.2(3)
O(1)	C(12)	C(13)	116.0(9)	C(17)	Ir(1)	C(16)	38.5(3)
C(12)	C(13)	C(14)	121.7(9)	C(17)	Ir(1)	C(18)	39.6(3)
C(9)	C(14)	Ir(1)	115.5(6)	C(17)	Ir(1)	C(19)	63.6(3)
C(13)	C(14)	C(9)	117.7(8)	C(17)	Ir(1)	C(20)	62.8(3)
C(13)	C(14)	Ir(1)	126.8(7)	C(17)	Ir(1)	Cl(1)	137.3(3)
C(17)	C(16)	C(20)	107.7(9)	C(18)	Ir(1)	C(16)	65.3(4)
C(17)	C(16)	C(21)	127.2(8)	C(18)	Ir(1)	C(19)	38.2(3)
C(17)	C(16)	Ir(1)	69.9(5)	C(18)	Ir(1)	C(20)	62.7(3)
C(20)	C(16)	C(21)	124.8(8)	C(18)	Ir(1)	Cl(1)	100.9(2)
C(20)	C(16)	Ir(1)	75.9(5)	C(19)	Ir(1)	C(20)	35.6(3)
C(21)	C(16)	Ir(1)	124.8(7)	C(19)	Ir(1)	Cl(1)	95.9(2)
C(16)	C(17)	C(18)	108.2(8)	C(20)	Ir(1)	Cl(1)	121.7(2)
C(16)	C(17)	C(22)	126.4(9)	N(2)	Ir(1)	C(16)	102.8(3)
C(16)	C(17)	Ir(1)	71.5(5)	N(2)	Ir(1)	C(17)	134.3(3)
C(18)	C(17)	C(22)	125.4(9)	N(2)	Ir(1)	C(18)	165.8(3)
C(18)	C(17)	Ir(1)	70.7(5)	N(2)	Ir(1)	C(19)	130.4(3)
C(22)	C(17)	Ir(1)	126.1(6)	N(2)	Ir(1)	C(20)	103.2(3)
C(17)	C(18)	C(23)	126.3(8)	N(2)	Ir(1)	Cl(1)	87.94(18)

C(17)	C(18)	Ir(1)	69.7(5)	C(1)	N(1)	C(5)	116.7(8)
C(19)	C(18)	C(17)	106.1(8)	C(5)	N(2)	Ir(1)	124.2(6)
C(19)	C(18)	C(23)	126.4(8)	C(8)	N(2)	C(5)	119.7(7)
C(19)	C(18)	Ir(1)	75.0(5)	C(8)	N(2)	Ir(1)	116.1(5)
C(23)	C(18)	Ir(1)	129.6(6)	C(12)	O(1)	C(15)	117.9(7)
C(18)	C(19)	C(24)	124.5(8)				

Table S10. Hydrogen Atom Coordinates (Å×10^4) and Isotropic Displacement Parameters (Å^2×10^3) for **Ir-3**. Related to **Table 1**.

Atom	*x*	*y*	*z*	U(eq)
H(1)	179.5	6529.69	-255.38	30
H(2)	278.59	7984.1	-755.41	35
H(3)	1360.63	9092.16	39.49	28
H(6)	2754.63	9494.83	1390.15	27
H(7)	3934.73	8990.1	2551.23	24
H(10)	5238.41	8317.93	3591.53	21
H(11)	6559.39	7557.2	4594.56	24
H(13)	4694.44	5220.43	3851.87	19
H(15A)	8361.16	6534.5	5061.52	35
H(15B)	8091.64	5778.45	5726.26	35
H(15C)	7080.1	6641.16	5712.29	35
H(21A)	-1582.4	6440.83	3453.81	46
H(21B)	-540.4	7050.56	2911.77	46
H(21C)	-70.98	6787.5	3816.27	46
H(22A)	1764.71	5816.42	4502.18	46
H(22B)	2300.14	4807.76	4482.86	46
H(22C)	636.42	5032.8	4680.29	46
H(23A)	3268.95	3775.21	3476.79	36
H(23B)	2856.32	3335.27	2622.84	36
H(23C)	1823.73	3191.27	3394.19	36
H(24A)	1987.92	3797.17	1322.17	41
H(24B)	800.31	4391.11	865.54	41
H(24C)	303	3536.37	1369.29	41
H(25A)	-2173.78	5537.53	1777.93	37
H(25B)	-1029.28	5489.81	1046.84	37
H(25C)	-1173.12	6376.16	1574.28	37

Crystal structure determination of Ir-3. Related to **Table 1**.

Crystal Data for $C_{25}H_{26}ClIrN_2O$ (*M* =598.13 g/mol): orthorhombic, space group $P2_12_12_1$ (no. 19), *a* = 9.0070(4) Å, *b* = 14.7851(8) Å, *c* = 16.1262(6) Å, *V* = 2147.52(17) Å^3, *Z* = 4, *T* = 100.00(10) K, μ(MoKα) = 6.361 mm^{-1}, *Dcalc* = 1.850 g/cm^3, 14026 reflections measured

(5.052° ≤ 2Θ ≤ 49.994°), 3783 unique (R_{int} = 0.0488, R_{sigma} = 0.0487) which were used in all calculations. The final R_1 was 0.0294 (I > 2σ(I)) and wR_2 was 0.0545 (all data).

Data S3. Analytic data of the obtained compounds. Related to **Table 1, Scheme 2, Scheme 3** & **Scheme 4.**

Complex Ir-1

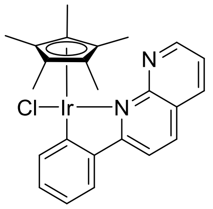

Orange red solid (107.9 mg, 95% Yield); ^{1}H NMR (400 MHz, $CDCl_3$) δ 9.06 – 8.94 (m, 1H), 8.02 (t, *J* = 9.2 Hz, 2H), 7.90 (dd, *J* = 20.0, 8.6 Hz, 2H), 7.78 (d, *J* = 7.7 Hz, 1H), 7.43 (dd, *J* = 7.8, 4.2 Hz, 1H), 7.25 (t, *J* = 7.1 Hz, 1H), 7.07 (t, *J* = 7.4 Hz, 1H), 1.68 (s, 15H). ^{13}C NMR (101 MHz, $CDCl_3$) δ 170.77, 167.73, 154.67, 153.34, 145.43, 138.08, 136.92, 136.23, 131.76, 126.42, 121.91, 121.89, 121.74, 117.94, 89.78, 9.84. IR (KBr): 3055, 2966, 2907, 1604, 1533, 1507, 1467, 1427, 1323, 1283, 847 cm^{-1}. HRMS (ESI): Calcd. for $C_{24}H_{24}IrN_2$ [M-Cl]$^+$: 533.1563; found: 533.1559.

Complex Ir-2

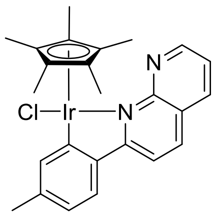

Orange red solid (105.9 mg, 91% Yield); ^{1}H NMR (400 MHz, $CDCl_3$) δ 8.97 (d, *J* = 2.6 Hz, 1H), 7.97 (d, *J* = 7.7 Hz, 1H), 7.90 – 7.82 (m, 2H), 7.78 (d, *J* = 8.6 Hz, 1H), 7.65 (d, *J* = 7.9 Hz, 1H), 7.38 (dd, *J* = 7.8, 4.2 Hz, 1H), 6.88 (d, *J* = 7.8 Hz, 1H), 2.47 (s, 3H), 1.67 (s, 15H). ^{13}C NMR (101 MHz, $CDCl_3$) δ 170.66, 167.83, 154.66, 153.15, 142.97, 141.79, 137.95, 136.89, 126.32, 123.00, 121.70, 121.63, 117.82, 89.61, 21.89, 9.82. IR (KBr): 3052, 2966, 2909, 2787, 1604, 1582, 1550, 1506, 1452, 1322, 1283, 846, 798 cm^{-1}. HRMS (ESI): Calcd. for $C_{25}H_{26}IrN_2$ [M-Cl]$^+$: 547.1720; found: 547.1723.

Complex Ir-3

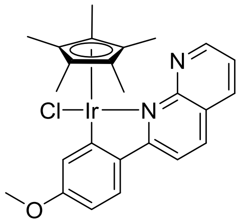

Orange red solid (112.4 mg, 94% Yield); ^{1}H NMR (400 MHz, $CDCl_3$) δ 8.96 (dd, *J* = 4.1, 1.6 Hz, 1H), 7.97 (d, *J* = 7.9 Hz, 1H), 7.85 (d, *J* = 8.6 Hz, 1H), 7.73 (dd, *J* = 13.8, 8.7 Hz, 2H), 7.57 (d, *J* = 2.4 Hz, 1H), 7.38 (dd, *J* = 7.9, 4.3 Hz, 1H), 6.65 (dd, *J* = 8.6, 2.4 Hz, 1H), 3.95 (s, 3H), 1.68 (s, 15H). ^{13}C NMR (101 MHz, $CDCl_3$) δ 170.21, 170.13, 162.02, 154.74, 153.04, 138.73, 137.76, 136.82, 128.13, 121.39, 121.37, 119.60, 117.79, 109.29, 89.68, 55.14, 9.85. IR (KBr): 3056, 2966, 2907, 2789, 1605, 1536, 1508, 1465, 1425, 1292, 1264, 1220, 842 cm^{-1}. HRMS (ESI): Calcd. for $C_{25}H_{26}IrN_2O$ [M-Cl]$^+$: 563.1669; found: 563.1677. Crystals suitable for a single-crystal X-ray diffraction study were grown from a concentrated solution of $CHCl_3$ layered with *n*-hexane in degassed NMR tube.

Complex Ir-4

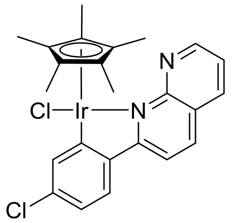

Red solid (105.9 mg, 88% Yield); ^{1}H NMR (400 MHz, $CDCl_3$) δ 9.01 (dd, *J* = 4.2, 1.9 Hz, 1H), 8.01 (dd, *J* = 8.0, 1.8 Hz, 1H), 7.96 (d, *J* = 2.0 Hz, 1H), 7.83 (d, *J* = 8.6 Hz, 1H), 7.72 (d, *J* = 8.7 Hz, 1H), 7.57 (d, *J* = 8.4 Hz, 1H), 7.44 (dd, *J* = 7.9, 4.2 Hz, 1H), 6.86 (dd, *J* = 8.3, 2.0 Hz, 1H), 1.66 (s, 15H). ^{13}C NMR (101 MHz, $CDCl_3$) δ 169.58, 168.96, 154.40, 153.40, 144.14, 138.44, 137.40, 137.09, 135.33, 129.04, 127.48, 122.02, 121.97,

118.11, 89.93, 9.77. IR (KBr): 3056, 2966, 2910, 2788, 1603, 1531, 1508, 1451, 1318, 1275, 1087, 1028, 842, 734 cm^{-1}. HRMS (ESI): Calcd. for $C_{24}H_{23}ClIrN_2$ [M-Cl]$^+$: 567.1174; found: 567.1168.

Complex Ir-5

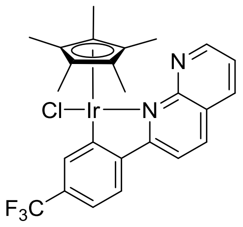

Brown solid (115.7 mg, 91% Yield); ^{1}H NMR (400 MHz, CD_2Cl_2) δ 9.15 (d, *J* = 2.2 Hz, 1H), 8.30 (s, 1H), 8.24 (d, *J* = 7.8 Hz, 1H), 8.17 (d, *J* = 8.6 Hz, 1H), 8.11 (d, *J* = 8.5 Hz, 1H), 8.00 (d, *J* = 7.9 Hz, 1H), 7.64 (dd, *J* = 7.8, 4.2 Hz, 1H), 7.38 (d, *J* = 7.8 Hz, 1H), 1.71 (s, 15H). ^{13}C NMR (101 MHz, CD_2Cl_2) δ 169.18, 167.52, 154.52, 153.78, 149.13, 138.76, 137.22, 132.26 (q, $J_{C\text{-}F}$ = 3.8 Hz), 131.58, 125.96, 122.75, 122.50, 118.27, 118.22, 90.28, 9.53. ^{19}F NMR (376 MHz, CD_2Cl_2) δ -62.52. IR (KBr): 3076, 2964, 2915, 2790, 1603, 1510, 1453, 1428, 1318, 1109 cm^{-1}. HRMS (ESI): Calcd. for $C_{25}H_{23}F_3IrN_2$ [M-Cl]$^+$: 601.1437; found: 601.1437.

Complex Ir-6

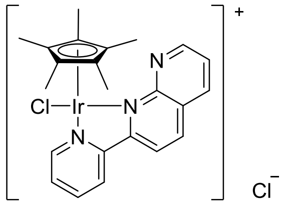

Brownish red solid (115.0 mg, 95% Yield); ^{1}H NMR (400 MHz, $CDCl_3$) δ 9.21 (d, *J* = 6.7 Hz, 1H), 9.15 – 9.07 (m, 2H), 8.96 – 8.84 (m, 2H), 8.55 (d, *J* = 7.2 Hz, 1H), 8.26 (s, 1H), 7.86 (s, 1H), 7.72 (d, *J* = 3.4 Hz, 1H), 1.52 (s, 15H). ^{13}C NMR (101 MHz, $CDCl_3$) δ 158.81, 156.01, 155.39, 152.29, 151.76, 143.60, 141.06, 138.98, 129.84, 127.75, 125.33, 125.08, 122.11, 89.97, 9.71. IR (KBr): 3049, 2964, 2923, 2792, 1602, 1547, 1515, 1470, 1427, 1262, 1030, 859, 799 cm^{-1}. HRMS (ESI): Calcd. for $C_{23}H_{24}ClIrN_3$ [M-Cl]$^+$: 570.1283; found: 570.1272.

Complex Ir-7

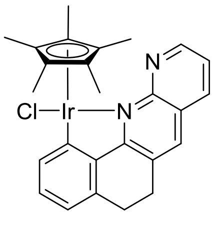

Brownish red solid (104.5 mg, 88% Yield); ^{1}H NMR (400 MHz, $CDCl_3$) δ 8.85 (d, *J* = 2.8 Hz, 1H), 7.91 (d, *J* = 7.7 Hz, 1H), 7.76 (d, *J* = 7.6 Hz, 1H), 7.61 (s, 1H), 7.32 (dd, *J* = 7.7, 4.2 Hz, 1H), 7.10 (t, *J* = 7.4 Hz, 1H), 6.74 (d, *J* = 7.2 Hz, 1H), 3.12 – 2.85 (m, 4H), 1.63 (s, 15H). ^{13}C NMR (101 MHz, $CDCl_3$) δ 168.92, 166.66, 153.99, 152.17, 143.60, 139.66, 136.34, 134.93, 133.50, 132.09, 131.41, 122.39, 121.62, 120.85, 89.51, 28.45, 27.83, 9.93. IR (KBr): 3060, 2966, 2914, 2794, 1509, 1466, 1427, 1316, 1270, 1026, 845, 753, 732 cm^{-1}. HRMS (ESI): Calcd. for $C_{26}H_{26}IrN_2$ [M-Cl]$^+$: 559.1720; found: 559.1725.

Complex Ir-8

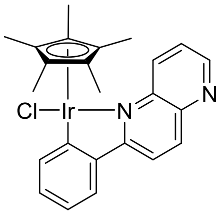

Yellow solid (96.6 mg, 85% Yield); ^{1}H NMR (400 MHz, $CDCl_3$) δ 9.66 (s, 1H), 8.51 (d, *J* = 8.4 Hz, 1H), 8.18 (t, *J* = 8.0 Hz, 3H), 7.66 (dd, *J* = 8.4, 5.2 Hz, 1H), 7.59 – 7.45 (m, 3H), 1.51 (s, 15H). ^{13}C NMR (101 MHz, $CDCl_3$) δ 158.56, 144.78, 143.90, 140.29, 139.31, 137.82, 130.36, 129.08, 127.68, 125.27, 122.56, 86.27, 8.89. IR (KBr): 3045, 2965, 2906, 2794, 1607, 1568, 1489, 1439, 1273, 1028, 731 cm^{-1}. HRMS (ESI): Calcd. for $C_{24}H_{24}IrN_2$ [M-Cl]$^+$: 533.1563; found: 533.1560.

Complex Ir-9

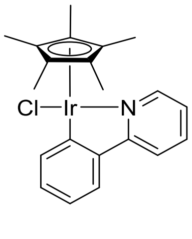

Yellow solid (91.0 mg, 88% Yield); ^{1}H NMR (400 MHz, $CDCl_3$) δ 8.71 (d, *J* = 5.5 Hz, 1H), 7.89 – 7.78 (m, 2H), 7.67 (dd, *J* = 15.1, 7.7 Hz, 2H), 7.22 (t, *J* =

7.3 Hz, 1H), 7.14 – 6.99 (m, 2H), 1.70 (s, 15H). ^{13}C NMR (101 MHz, $CDCl_3$) δ 167.33, 163.36, 151.34, 144.16, 137.05, 135.81, 130.96, 123.86, 122.33, 122.07, 118.89, 88.54, 8.93. IR (KBr): 3039, 2967, 2916, 1620, 1600, 1543, 1371, 1024, 753, 734 cm^{-1}. HRMS (ESI): Calcd. for $C_{21}H_{23}IrN$ $[M-Cl]^+$: 482.1454; found: 482.1456.

(1) 2-phenylquinazoline **(3aa)**

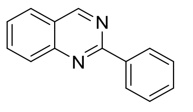

Pale yellow solid (87.6 mg, 85% Yield), m.p.: 99-100°C (Chen et al., 2014); ^{1}H NMR (400 MHz, $CDCl_3$) δ 9.38 (s, 1H), 8.54 (d, *J* = 7.2 Hz, 2H), 8.01 (d, *J* = 8.4 Hz, 1H), 7.82 (t, *J* = 8.8 Hz, 2H), 7.58 – 7.38 (m, 4H). ^{13}C NMR (101 MHz, $CDCl_3$) δ 161.06, 160.52, 150.78, 138.02, 134.16, 130.66, 128.67, 128.62, 127.30, 127.15, 123.62. IR (KBr): 3063, 2963, 2928, 1616, 1549, 772, 704 cm^{-1}. MS (EI, m/z): 206.15 $[M]^+$.

(2) 2-(p-tolyl)quinazoline **(3ab)**

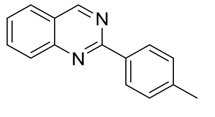

Pale yellow solid, (82.5 mg, 75% Yield), m.p.: 98-99°C (Chen et al., 2014); ^{1}H NMR (400 MHz, $CDCl_3$) δ 9.43 (s, 1H), 8.51 (d, *J* = 8.0 Hz, 2H), 8.06 (d, *J* = 8.4 Hz, 1H), 7.88 (d, *J* = 7.9 Hz, 2H), 7.57 (t, *J* = 7.5 Hz, 1H), 7.33 (d, *J* = 7.9 Hz, 2H), 2.44 (s, 3H). ^{13}C NMR (101 MHz, $CDCl_3$) δ 161.17, 160.43, 150.83, 140.88, 135.35, 134.03, 129.42, 128.57, 127.12, 127.03, 123.54, 21.53. IR (KBr): 3028, 2919, 2795, 1619, 1550, 724 cm^{-1}. MS (EI, m/z): 220.14 $[M]^+$.

(3) 2-(o-tolyl)quinazoline **(3ac)**

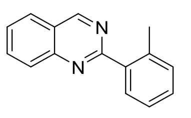

Pale yellow solid, (63.8 mg, 58% Yield), m.p.: 45-46°C (Ma et al., 2017); ^{1}H NMR (400 MHz, $CDCl_3$) δ 9.50 (s, 1H), 8.10 (d, *J* = 8.2 Hz, 1H), 8.01 – 7.87 (m, 3H), 7.66 (dd, *J* = 10.8, 3.8 Hz, 1H), 7.35 (d, *J* = 5.1 Hz, 3H), 2.61 (s, 3H). ^{13}C NMR (101 MHz, $CDCl_3$) δ 164.05, 160.09, 150.42, 138.59, 137.43, 134.15, 131.32, 130.68, 129.34, 128.60, 127.55, 127.09, 125.99, 122.94, 21.05. IR (KBr): 3058, 2964, 2924, 1619, 1553, 769, 727 cm^{-1}. MS (EI, m/z): 220.16 $[M]^+$.

(4) 2-(4-methoxyphenyl)quinazoline **(3ad)**

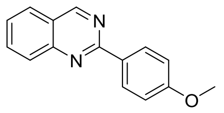

Pale yellow solid, (84.9 mg, 72% Yield), m.p.: 96-97°C (Chen et al., 2014); ^{1}H NMR (400 MHz, $CDCl_3$) δ 9.38 (s, 1H), 8.57 (d, *J* = 8.0 Hz, 2H), 8.02 (d, *J* = 8.4 Hz, 1H), 7.84 (d, *J* = 7.3 Hz, 2H), 7.52 (t, *J* = 7.3 Hz, 1H), 7.03 (d, *J* = 8.0 Hz, 2H), 3.87 (s, 3H). ^{13}C NMR (101 MHz, $CDCl_3$) δ 161.87, 160.86, 160.38, 150.84, 134.00, 130.76, 130.25, 128.41, 127.12, 126.77, 123.32, 113.99, 55.38. IR (KBr): 3055, 2969, 2833, 1605, 1585, 1407, 1247, 1162, 1028, 836, 796, 733 cm^{-1}. MS (EI, m/z): 236.15 $[M]^+$.

(5) 2-(quinazolin-2-yl)phenol **(3ae)**

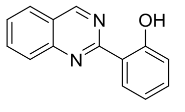

White solid, (61.1 mg, 55% Yield), m.p.: 135-136°C (Gujjarappa et al., 2018); ^{1}H NMR (400 MHz, $CDCl_3$) δ 9.48 (s, 1H), 8.66 (d, *J* = 7.9 Hz, 1H), 8.07 – 7.87 (m, 3H), 7.64 (t, *J* = 7.3 Hz, 1H), 7.42 (t, *J* = 7.6 Hz, 1H), 7.08 (d, *J* = 8.1 Hz, 1H), 7.01 (t, *J* = 7.6 Hz, 1H). ^{13}C NMR (101 MHz, $CDCl_3$) δ 161.78, 160.89, 160.50, 148.10, 134.97, 133.24, 129.72, 127.56, 127.43, 127.04, 123.01, 119.19, 119.08, 117.87. IR (KBr): 3351, 3041, 1584, 1476, 1382, 1280, 1239, 759 cm^{-1}. MS (EI, m/z): 222.12 $[M]^+$.

(6) 4-(quinazolin-2-yl)aniline **(3af)**

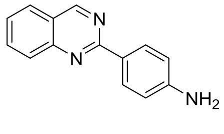

Pale yellow solid, (79.6 mg, 72% Yield), m.p.: 176-177°C (Saha et al., 2017); ^{1}H NMR (400 MHz, $CDCl_3$) δ 9.38 (s, 1H), 8.45 (d, *J* = 7.7 Hz, 2H),

8.00 (d, *J* = 8.3 Hz, 1H), 7.85 (t, *J* = 8.3 Hz, 2H), 7.53 (t, *J* = 7.2 Hz, 1H), 6.80 (d, *J* = 7.7 Hz, 2H), 3.96 (s, 2H). ^{13}C NMR (101 MHz, $CDCl_3$) δ 161.23, 160.32, 150.93, 149.03, 133.93, 130.22, 128.32, 128.28, 127.15, 126.41, 123.20, 114.80. IR (KBr): 3413, 3319, 1604, 1580, 1483, 1398, 1288, 1170, 836, 798, 734 cm^{-1}. MS (EI, m/z): 221.14 $[M]^+$.

(7) 2-(4-chlorophenyl)quinazoline **(3ag)**

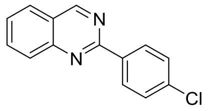

Pale yellow solid, (108 mg, 90% Yield), m.p.: 137-138°C (Chen et al., 2014); ^{1}H NMR (400 MHz, $CDCl_3$) δ 9.38 (s, 1H), 8.53 (d, *J* = 8.0 Hz, 2H), 8.02 (d, *J* = 8.3 Hz, 1H), 7.85 (d, *J* = 7.5 Hz, 2H), 7.56 (t, *J* = 7.0 Hz, 1H), 7.46 (d, *J* = 8.0 Hz, 2H). ^{13}C NMR (101 MHz, $CDCl_3$) δ 160.48, 159.98, 150.66, 136.83, 136.52, 134.22, 129.92, 128.80, 128.59, 127.43, 127.13, 123.60. IR (KBr): 3052, 2968, 1619, 1551, 1487, 1409, 846, 796, 724 cm^{-1}. MS (EI, m/z): 240.10 $[M]^+$.

(8) 2-(3-chlorophenyl)quinazoline **(3ah)**

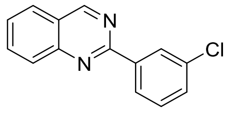

Pale yellow solid, (91.2 mg, 76% Yield), m.p.: 149-150°C (Han et al., 2012); ^{1}H NMR (400 MHz, $CDCl_3$) δ 9.44 (s, 1H), 8.62 (s, 1H), 8.50 (d, *J* = 6.6 Hz, 1H), 8.07 (d, *J* = 8.8 Hz, 1H), 7.91 (d, *J* = 7.7 Hz, 2H), 7.62 (t, *J* = 7.5 Hz, 1H), 7.50 – 7.40 (m, 2H). ^{13}C NMR (101 MHz, $CDCl_3$) δ 160.56, 159.70, 150.66, 139.88, 134.80, 134.31, 130.55, 129.85, 128.68, 127.65, 127.15, 126.66, 123.77. IR (KBr): 3067, 2967, 1617, 1549, 780, 760, 716 cm^{-1}. MS (EI, m/z): 240.10 $[M]^+$.

(9) 2-(4-bromophenyl)quinazoline **(3ai)**

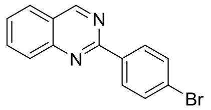

Pale yellow solid, (106 mg, 75% Yield), m.p.: 121-122°C (Chen et al., 2014); ^{1}H NMR (400 MHz, $CDCl_3$) δ 9.42 (s, 1H), 8.49 (d, *J* = 8.3 Hz, 2H), 8.05 (d, *J* = 8.8 Hz, 1H), 7.89 (t, *J* = 7.2 Hz, 2H), 7.69 – 7.55 (m, 3H). ^{13}C NMR (101 MHz, $CDCl_3$) δ 160.52, 160.12, 150.70, 137.00, 134.25, 131.79, 130.17, 128.64, 127.48, 127.16, 125.42, 123.66. IR (KBr): 3066, 2926, 1618, 1549, 1407, 796, 724 cm^{-1}. MS (EI, m/z): 284.03 $[M]^+$.

(10) 2-(4-(trifluoromethyl)phenyl)quinazoline **(3aj)**

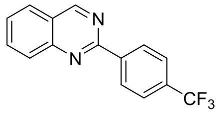

Pale yellow solid, (68.5 mg, 50% Yield), m.p.: 143-145°C (Chen et al., 2014); ^{1}H NMR (400 MHz, $CDCl_3$) δ 9.46 (s, 1H), 8.73 (d, *J* = 8.1 Hz, 2H), 8.10 (d, *J* = 8.8 Hz, 1H), 7.92 (t, *J* = 7.6 Hz, 2H), 7.77 (d, *J* = 8.2 Hz, 2H), 7.64 (t, *J* = 7.5 Hz, 1H). ^{13}C NMR (101 MHz, $CDCl_3$) δ 160.60, 159.60, 150.66, 141.32, 134.37, 132.29, 131.97, 128.84, 128.78, 127.86, 127.16, 125.59, 125.50 (q, J_{C-F} = 3.8 Hz), 123.84. ^{19}F NMR (376 MHz, $CDCl_3$) δ -62.65. IR (KBr): 3067, 2967, 1616, 1550, 1326, 1109, 855 cm^{-1}. MS (EI, m/z): 274.17 $[M]^+$.

(11) methyl 4-(quinazolin-2-yl)benzoate **(3ak)**

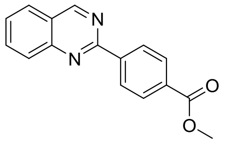

White solid, (101 mg, 77% Yield), m.p.: 162-163°C (Yamaguchi et al., 2016); ^{1}H NMR (400 MHz, $CDCl_3$) δ 9.49 (s, 1H), 8.70 (d, *J* = 8.3 Hz, 2H), 8.20 (d, *J* = 8.3 Hz, 2H), 8.11 (d, *J* = 8.4 Hz, 1H), 7.94 (t, *J* = 9.0 Hz, 2H), 7.65 (t, *J* = 7.5 Hz, 1H), 3.96 (s, 3H). ^{13}C NMR (101 MHz, $CDCl_3$) δ 166.95, 160.56, 160.01, 150.69, 142.15, 134.31, 131.72, 129.85, 128.78, 128.50, 127.79, 127.15, 123.77, 52.20. IR (KBr): 3062, 2968, 1720, 1619, 1549, 1286, 1113, 770, 711 cm^{-1}. MS (EI, m/z): 264.16 $[M]^+$.

(12) 4-(quinazolin-2-yl)benzonitrile **(3al)**

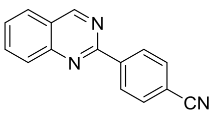

Pale yellow solid, (84.3 mg, 73% Yield), m.p.: 194-196°C (Yamaguchi et al., 2016); ^{1}H NMR (400 MHz, $CDCl_3$) δ 9.52 (s, 1H), 8.77 (d, *J* = 8.2 Hz, 2H), 8.14 (d, *J* = 8.4 Hz, 1H), 7.98 (t, *J* = 8.2 Hz, 2H), 7.84 (d, *J* = 8.2 Hz, 2H), 7.71 (t, *J* = 7.5 Hz, 1H). ^{13}C NMR (101 MHz, $CDCl_3$) δ 160.68, 159.08, 150.62, 142.13, 134.54, 132.37, 129.02, 128.82, 128.18, 127.21, 123.89, 118.89, 113.81. IR (KBr): 3065, 2968, 2225, 1616, 1546, 1429, 852, 799 cm^{-1}. MS (EI, m/z): 231.15 $[M]^+$.

(13) phenyl(4-(quinazolin-2-yl)phenyl)methanone **(3am)**

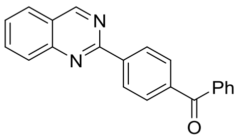

Pale yellow solid, (106.1 mg, 68% Yield), m.p.: 167-168°C (Chen et al., 2014); ^{1}H NMR (400 MHz, $CDCl_3$) δ 9.51 (s, 1H), 8.76 (d, *J* = 8.1 Hz, 2H), 8.13 (d, *J* = 8.3 Hz, 1H), 7.96 (dd, *J* = 16.0, 8.5 Hz, 4H), 7.88 (d, *J* = 7.5 Hz, 2H), 7.70 – 7.58 (m, 2H), 7.53 (t, *J* = 7.5 Hz, 2H). ^{13}C NMR (101 MHz, $CDCl_3$) δ 196.55, 160.63, 160.04, 150.72, 141.65, 139.06, 137.62, 134.37, 132.58, 130.36, 130.13, 128.80, 128.44, 128.37, 127.85, 127.20, 123.80. IR (KBr): 3061, 1656, 1577, 1274, 926, 861, 751, 706 cm^{-1}. MS (EI, m/z): 310.12 $[M]^+$.

(14) 2-(naphthalen-1-yl)quinazoline **(3an)**

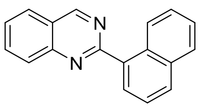

Pale yellow solid, (87.0 mg, 68% Yield), m.p.: 125-126°C (Ma et al., 2017); ^{1}H NMR (400 MHz, $CDCl_3$) δ 9.56 (s, 1H), 8.72 (d, *J* = 8.2 Hz, 1H), 8.17 (t, *J* = 8.0 Hz, 2H), 8.02 – 7.87 (m, 4H), 7.63 (dd, *J* = 17.6, 7.9 Hz, 2H), 7.58 – 7.47 (m, 2H). ^{13}C NMR (101 MHz, $CDCl_3$) δ 163.49, 160.44, 150.60, 136.34, 134.35, 134.24, 131.28, 130.44, 129.71, 128.68, 128.54, 127.77, 127.17, 126.91, 125.99, 125.94, 125.35, 123.16. IR (KBr): 3053, 2967, 1618, 1551, 1467, 1429, 972, 760 cm^{-1}. MS (EI, m/z): 256.13 $[M]^+$.

(15) 2-(pyridin-3-yl)quinazoline **(3ao)**

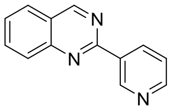

White solid, (67.2 mg, 65% Yield), m.p.: 94-95°C (Ma et al., 2017); ^{1}H NMR (400 MHz, $CDCl_3$) δ 9.83 (s, 1H), 9.47 (s, 1H), 8.87 (d, *J* = 7.9 Hz, 1H), 8.75 (s, 1H), 8.10 (d, *J* = 8.4 Hz, 1H), 7.93 (t, *J* = 8.1 Hz, 2H), 7.65 (t, *J* = 7.5 Hz, 1H), 7.46 (dd, *J* = 7.7, 4.4 Hz, 1H). ^{13}C NMR (101 MHz, $CDCl_3$) δ 160.65, 159.13, 151.12, 150.60, 150.20, 135.84, 134.40, 133.57, 128.64, 127.80, 127.19, 123.81, 123.43. IR (KBr): 3055, 2924, 2792, 1617, 1549, 1427, 1292, 759, 710 cm^{-1}. MS (EI, m/z): 207.13 $[M]^+$.

(16) 2-(thiophen-2-yl)quinazoline **(3ap)**

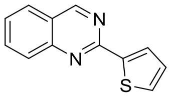

Pale yellow solid, (60.4 mg, 57% Yield), m.p.: 133-134°C (Chen et al., 2014); ^{1}H NMR (400 MHz, $CDCl_3$) δ 9.34 (s, 1H), 8.15 (d, *J* = 3.6 Hz, 1H), 8.00 (d, *J* = 8.8 Hz, 1H), 7.86 (dd, *J* = 7.2, 5.1 Hz, 2H), 7.60 – 7.46 (m, 2H), 7.19 (t, *J* = 4.3 Hz, 1H). ^{13}C NMR (101 MHz, $CDCl_3$) δ 160.56, 157.88, 150.64, 143.84, 134.39, 129.98, 129.28, 128.40, 128.21, 127.29, 127.03, 123.40. IR (KBr): 3065, 2966, 2789, 1616, 1551, 1425, 713 cm^{-1}. MS (EI, m/z): 212.08 $[M]^+$.

(17) (*E*)-2-styrylquinazoline **(3aq)**

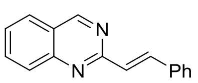

White solid, (53.3 mg, 46% Yield), m.p.: 120-121°C (Han et al., 2012); ^{1}H NMR (400 MHz, $CDCl_3$) δ 9.38 (s, 1H), 8.17 (d, *J* = 16.0 Hz, 1H), 8.01 (d, *J* = 8.5 Hz, 1H), 7.89 (t, *J* = 7.7 Hz, 2H), 7.69 (d, *J* = 7.6 Hz, 2H), 7.60 (t, *J* = 7.5 Hz, 1H), 7.46 – 7.38 (m, 3H), 7.38 – 7.33 (m, 1H). ^{13}C NMR (101 MHz, $CDCl_3$) δ 161.33, 160.25, 150.61,

138.60, 136.24, 134.22, 129.07, 128.83, 128.15, 127.94, 127.70, 127.23, 127.16, 123.39. IR (KBr): 3057, 2966, 1613, 1550, 1376, 1234, 980, 750 cm^{-1}. MS (EI, m/z): 232.15 $[M]^+$.

(18) quinazoline **(3ar)**

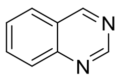

Brown solid, (31.2 mg, 48% Yield), m.p.: 45-46°C (Zhang et al., 2015); ^1H NMR (400 MHz, $CDCl_3$) δ 9.41 (s, 1H), 9.35 (s, 1H), 8.06 (d, *J* = 8.7 Hz, 1H), 7.93 (t, *J* = 7.0 Hz, 2H), 7.68 (t, *J* = 7.4 Hz, 1H). ^{13}C NMR (101 MHz, $CDCl_3$) δ 160.22, 155.24, 150.00, 134.19, 128.39, 127.95, 127.19, 125.09. IR (KBr): 3060, 2969, 1619, 1567, 1488, 1377, 754 cm^{-1}. MS (EI, m/z): 130.05 $[M]^+$.

(19) 2-hexylquinazoline **(3as)**

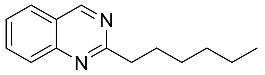

Pale yellow oil liquid (Zhang et al., 2015), (64.2 mg, 60% Yield); ^1H NMR (400 MHz, $CDCl_3$) δ 9.27 (s, 1H), 7.90 (d, *J* = 8.5 Hz, 1H), 7.80 (t, *J* = 7.5 Hz, 2H), 7.51 (t, *J* = 7.0 Hz, 1H), 3.04 (t, *J* = 7.7 Hz, 2H), 1.84 (dt, *J* = 15.0, 7.6 Hz, 2H), 1.40 – 1.22 (m, 6H), 0.81 (d, *J* = 6.3 Hz, 3H). ^{13}C NMR (101 MHz, $CDCl_3$) δ 167.96, 160.38, 150.38, 133.97, 127.89, 127.07, 126.89, 123.07, 40.04, 31.71, 29.23, 28.98, 22.56, 14.05. IR (KBr): 3063, 2959, 2927, 2858, 1619, 1529, 1466, 1428, 1232, 1141, 966, 753 cm^{-1}. MS (EI, m/z): 214.15 $[M]^+$.

(20) 2-cyclopropylquinazoline **(3at)**

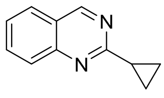

Pale yellow oil liquid (Zhang et al., 2015), (52.7 mg, 62% Yield); ^1H NMR (400 MHz, $CDCl_3$) δ 9.20 (s, 1H), 7.88 (d, *J* = 8.4 Hz, 1H), 7.80 (t, *J* = 8.5 Hz, 2H), 7.48 (t, *J* = 7.5 Hz, 1H), 2.47 – 2.34 (m, 1H), 1.32 – 1.23 (m, 2H), 1.16 – 1.07 (m, 2H). ^{13}C NMR (101 MHz, $CDCl_3$) δ 168.29, 160.22, 150.29, 133.86, 127.43, 127.00, 126.19, 123.14, 18.55, 10.60. IR (KBr): 3061, 3008, 1620, 1585, 1570, 1413, 1376, 758 cm^{-1}. MS (EI, m/z): 170.10 $[M]^+$.

(21) 6-methyl-2-phenylquinazoline **(3ba)**

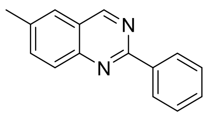

Pale yellow solid (85.8 mg, 78% Yield), m.p.: 129-130°C (Chen et al., 2014); ^1H NMR (400 MHz, $CDCl_3$) δ 9.30 (s, 1H), 8.52 (d, *J* = 7.3 Hz, 2H), 7.90 (d, *J* = 8.6 Hz, 1H), 7.65 (d, *J* = 8.7 Hz, 1H), 7.59 (s, 1H), 7.50 – 7.35 (m, 3H), 2.48 (s, 3H). ^{13}C NMR (101 MHz, $CDCl_3$) δ 160.40, 159.74, 149.36, 138.20, 137.43, 136.39, 130.41, 128.62, 128.46, 128.28, 125.79, 123.60, 21.64. IR (KBr): 3060, 2967, 2791, 1526, 1427, 831, 760 cm^{-1}. MS (EI, m/z): 220.18 $[M]^+$.

(22) 2-(4-methoxyphenyl)-6-methylquinazoline **(3bd)**

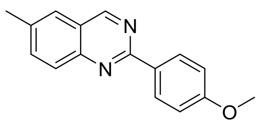

Pale yellow solid (100 mg, 80% Yield), m.p.: 119-120°C (Han et al., 2012); ^1H NMR (400 MHz, $CDCl_3$) δ 9.27 (s, 1H), 8.54 (d, *J* = 8.8 Hz, 2H), 7.90 (d, *J* = 8.6 Hz, 1H), 7.65 (dd, *J* = 8.6, 1.7 Hz, 1H), 7.57 (s, 1H), 7.02 (d, *J* = 8.8 Hz, 2H), 3.86 (s, 3H), 2.50 (s, 3H). ^{13}C NMR (101 MHz, $CDCl_3$) δ 161.69, 160.21, 159.64, 149.39, 136.85, 136.27, 130.90, 130.05, 128.05, 125.80, 123.30, 113.95, 55.35, 21.56. IR (KBr): 3047, 2962, 1602, 1552, 1514, 1425, 1244, 1026, 851, 827 cm^{-1}. MS (EI, m/z): 250.15 $[M]^+$.

(23) 8-methyl-2-phenylquinazoline **(3ca)**

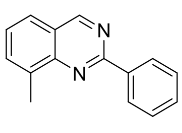

Pale yellow solid (69.3 mg, 63% Yield), m.p.: 59-60°C (Gopalaiah et al., 2017); ^1H NMR (400 MHz, $CDCl_3$) δ 9.41 (s, 1H), 8.67 (d, *J* = 6.7 Hz, 2H), 7.79 – 7.68 (m, 2H), 7.57 – 7.44 (m, 4H), 2.86 (s, 3H). ^{13}C NMR (101 MHz,

CDCl$_3$) δ 160.58, 159.98, 149.76, 138.42, 137.17, 133.87, 130.47, 128.60, 128.55, 126.93, 124.82, 123.55, 16.94. IR (KBr): 3064, 2967, 2791, 1528, 1468, 1427, 953, 760 cm^{-1}. MS (EI, m/z): 220.17 [M]$^{+}$.

(24) 5-methyl-2-phenylquinazoline **(3da)**

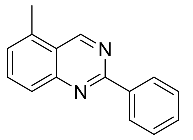

Pale yellow solid (58.3 mg, 53% Yield), m.p.: 115-117°C (Cheng et al., 2016); ^{1}H NMR (400 MHz, CDCl$_3$) δ 9.66 (s, 1H), 8.62 (d, *J* = 7.6 Hz, 2H), 7.93 (d, *J* = 8.5 Hz, 1H), 7.77 (t, *J* = 7.8 Hz, 1H), 7.58 – 7.47 (m, 3H), 7.38 (d, *J* = 7.0 Hz, 1H), 2.79 (s, 3H). ^{13}C NMR (101 MHz, CDCl$_3$) δ 160.69, 157.54, 151.28, 138.07, 135.49, 133.98, 130.54, 128.63, 128.55, 127.85, 126.85, 122.76, 17.56. IR (KBr): 3059, 2966, 2796, 1525, 1467, 1427, 755, 703 cm^{-1}. MS (EI, m/z): 220.16 [M]$^{+}$.

(25) 6-methoxy-2-phenylquinazoline **(3ea)**

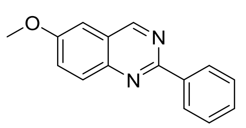

Pale yellow solid (100 mg, 85% Yield), m.p.: 120-121°C (Ma et al., 2017); ^{1}H NMR (400 MHz, CDCl$_3$) δ 9.22 (s, 1H), 8.47 (d, *J* = 7.2 Hz, 2H), 7.87 (d, *J* = 9.1 Hz, 1H), 7.39 (dd, *J* = 17.8, 7.2 Hz, 4H), 6.99 (s, 1H), 3.81 (s, 3H). ^{13}C NMR (101 MHz, CDCl$_3$) δ 159.39, 158.80, 158.25, 147.00, 138.23, 130.18, 130.13, 128.60, 128.22, 127.14, 124.47, 103.92, 55.69. IR (KBr): 3060, 2966, 2790, 1621, 1529, 1427, 1223, 1161, 1026, 834, 761 cm^{-1}. MS (EI, m/z): 236.16 [M]$^{+}$.

(26) 2-(4-chlorophenyl)-6-methoxyquinazoline **(3eg)**

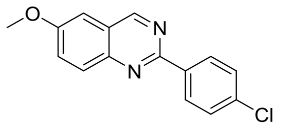

White solid (123 mg, 91% Yield), m.p.: 174-175°C (Cheng et al., 2016); ^{1}H NMR (400 MHz, CDCl$_3$) δ 9.29 (s, 1H), 8.50 (d, *J* = 8.6 Hz, 2H), 7.94 (d, *J* = 9.2 Hz, 1H), 7.53 (dd, *J* = 9.2, 2.7 Hz, 1H), 7.47 (s, 1H), 7.45 (s, 1H), 7.10 (d, *J* = 2.7 Hz, 1H), 3.94 (s, 3H). ^{13}C NMR (101 MHz, CDCl$_3$) δ 158.78, 158.40, 158.33, 146.90, 136.69, 136.33, 130.08, 129.52, 128.74, 127.30, 124.51, 103.93, 55.72. IR (KBr): 3065, 2966, 2790, 1531, 1469, 1428, 1318, 1223, 948, 837 cm^{-1}. MS (EI, m/z): 270.10 [M]$^{+}$.

(27) 6,7-dimethoxy-2-phenylquinazoline **(3fa)**

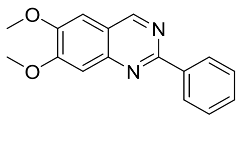

White solid (113 mg, 85% Yield), m.p.: 176-177°C (Gopalaiah et al., 2017); ^{1}H NMR (400 MHz, CDCl$_3$) δ 9.19 (s, 1H), 8.54 (d, *J* = 7.4 Hz, 2H), 7.57 – 7.42 (m, 3H), 7.35 (s, 1H), 7.06 (s, 1H), 4.06 (s, 3H), 4.01 (s, 3H). ^{13}C NMR (101 MHz, CDCl$_3$) δ 159.92, 157.09, 156.22, 150.35, 148.61, 138.39, 130.12, 128.57, 128.14, 119.40, 106.87, 103.95, 56.44, 56.20. IR (KBr): 3061, 2966, 1619, 1500, 1412, 1229, 1155, 855, 759 cm^{-1}. MS (EI, m/z): 266.13 [M]$^{+}$.

(28) 2-(4-bromophenyl)-6,7-dimethoxyquinazoline **(3fi)**

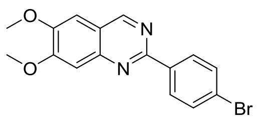

White solid, (129 mg, 75% Yield), m.p.: 157-159°C (unknown compound); ^{1}H NMR (400 MHz, CDCl$_3$) δ 9.07 (s, 1H), 8.32 (d, *J* = 8.5 Hz, 2H), 7.53 (d, *J* = 8.5 Hz, 2H), 7.24 (s, 1H), 6.98 (s, 1H), 3.98 (s, 3H), 3.94 (s, 3H). ^{13}C NMR (101 MHz, CDCl$_3$) δ 158.90, 157.02, 156.37, 150.54, 148.53, 137.30, 131.67, 129.71, 124.78, 119.48, 106.79, 103.94, 56.46, 56.23. IR (KBr): 3071, 2965, 1615, 1498, 1421, 1230, 1154, 842 cm^{-1}. MS (EI, m/z): 344.08 [M]$^{+}$. HRMS (ESI): Calcd. for $C_{16}H_{14}BrN_2O_2$ [M+H]$^{+}$: 345.0233; found: 345.0235.

(29) 6-chloro-2-phenylquinazoline **(3ga)**

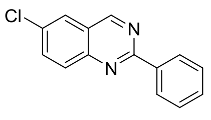

Pale yellow solid (69.6 mg, 58% Yield), m.p.: 157-158°C (Chen et al., 2014); ^{1}H NMR (400 MHz, $CDCl_3$) δ 9.39 (s, 1H), 8.60 (dd, *J* = 7.4, 2.1 Hz, 2H), 8.03 (d, *J* = 9.0 Hz, 1H), 7.89 (d, *J* = 2.2 Hz, 1H), 7.82 (dd, *J* = 9.0, 2.3 Hz, 1H), 7.57 – 7.48 (m, 3H). ^{13}C NMR (101 MHz, $CDCl_3$) δ 161.31, 159.50, 149.27, 137.61, 135.08, 132.81, 130.89, 130.41, 128.70, 128.62, 125.83, 124.00. IR (KBr): 3064, 2967, 1613, 1542, 1430, 837 cm^{-1}. MS (EI, m/z): 240.10 [M]$^+$.

(30) methyl 4-(6-chloroquinazolin-2-yl)benzoate **(3gk)**

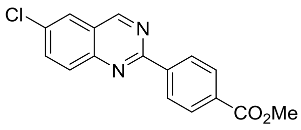

Pale yellow solid (81.9 mg, 55% Yield), m.p.: 198-199°C (unknown compound); ^{1}H NMR (400 MHz, $CDCl_3$) δ 9.44 (s, 1H), 8.70 (d, *J* = 7.8 Hz, 2H), 8.22 (d, *J* = 7.9 Hz, 2H), 8.08 (d, *J* = 9.1 Hz, 1H), 7.96 (s, 1H), 7.88 (d, *J* = 8.9 Hz, 1H), 3.99 (s, 3H). ^{13}C NMR (101 MHz, $CDCl_3$) δ 166.87, 160.26, 159.58, 149.17, 141.65, 135.32, 133.42, 131.96, 130.52, 129.88, 128.50, 125.86, 124.17, 52.25. IR (KBr): 3066, 2966, 1539, 1473, 1429, 1374, 1277, 1107, 832, 765, 715 cm^{-1}. MS (EI, m/z): 298.12 [M]$^+$. HRMS (ESI): Calcd. for $C_{16}H_{12}ClN_2O_2$ [M+H]$^+$: 299.0582; found: 299.0579.

(31) 6-fluoro-2-phenylquinazoline **(3ha)**

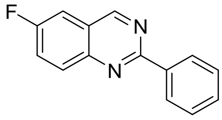

Pale yellow solid (62.7 mg, 56% Yield), m.p.: 140-141°C (Malakar et al., 2012); ^{1}H NMR (400 MHz, $CDCl_3$) δ 9.42 (s, 1H), 8.59 (d, *J* = 7.3 Hz, 2H), 8.10 (dd, *J* = 9.2, 5.0 Hz, 1H), 7.67 (td, *J* = 8.9, 2.3 Hz, 1H), 7.58 – 7.48 (m, 4H). ^{13}C NMR (101 MHz, $CDCl_3$) δ160.44 (d, $J_{C\text{-}F}$ = 251.0 Hz), 160.77, 159.79 (d, $J_{C\text{-}F}$ = 5.5 Hz), 147.98, 137.76, 131.41 (d, $J_{C\text{-}F}$ = 8.6 Hz), 130.70, 128.68, 128.49, 124.51 (d, $J_{C\text{-}F}$ = 25.8 Hz), 123.94 (d, $J_{C\text{-}F}$ = 9.3 Hz), 110.14 (d, $J_{C\text{-}F}$ = 21.8 Hz). ^{19}F NMR (376 MHz, $CDCl_3$) δ -110.69. IR (KBr): 3056, 2967, 1531, 1430, 1373, 1286, 837 cm^{-1}. MS (EI, m/z): 224.14 [M]$^+$.

(32) 7-bromo-2-phenylquinazoline **(3ia)**

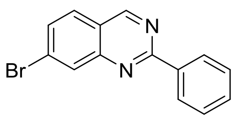

Pale yellow solid (71 mg, 50% Yield), m.p.: 125-127°C (Wang et al., 2014); ^{1}H NMR (400 MHz, $CDCl_3$) δ 9.43 (s, 1H), 8.60 (dd, *J* = 7.3, 2.2 Hz, 2H), 8.29 (s, 1H), 7.79 (d, *J* = 8.6 Hz, 1H), 7.70 (dd, *J* = 8.6, 1.6 Hz, 1H), 7.58 – 7.48 (m, 3H). ^{13}C NMR (101 MHz, $CDCl_3$) δ 161.83, 160.31, 151.44, 137.58, 131.18, 131.01, 130.98, 128.95, 128.73, 128.70, 128.30, 122.17, 77.35, 77.03, 76.71. IR (KBr): 3066, 2966, 1540, 1428, 1379, 1319, 935, 759, 700 cm^{-1}. MS (EI, m/z): 284.06 [M]$^+$.

(33) 4-methyl-2-phenylquinazoline **(3ja)**

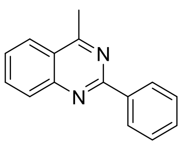

Pale yellow solid (84.7 mg, 77% Yield), m.p.: 89-90°C (Gopalaiah et al., 2017); ^{1}H NMR (400 MHz, $CDCl_3$) δ 8.63 (d, *J* = 7.4 Hz, 2H), 8.10 (d, *J* = 8.3 Hz, 2H), 7.87 (t, *J* = 7.7 Hz, 1H), 7.59 (t, *J* = 7.6 Hz, 1H), 7.56 – 7.46 (m, 3H), 3.03 (s, 3H). ^{13}C NMR (101 MHz, $CDCl_3$) δ 168.23, 160.21, 150.43, 138.34, 133.51, 130.39, 129.27, 128.57, 126.86, 124.98, 123.03, 22.02. IR (KBr): 3063, 2966, 1616, 1547, 1430, 1338, 757, 708 cm^{-1}. MS (EI, m/z): 220.16 [M]$^+$.

(34) 4-(4-methylquinazolin-2-yl)benzonitrile **(3jl)**

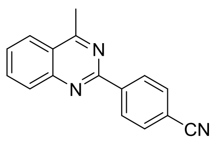

Brown solid (67.3 mg, 55% Yield), m.p.: 195-197°C (Yu et al., 2017); ^{1}H NMR (400 MHz, $CDCl_3$) δ 8.69 (d, J = 7.7 Hz, 2H), 8.11 – 7.99 (m, 2H), 7.84 (t, J = 7.7 Hz, 1H), 7.74 (d, J = 7.8 Hz, 2H), 7.58 (t, J = 7.6 Hz, 1H), 2.97 (s, 3H). ^{13}C NMR (101 MHz, $CDCl_3$) δ 168.66, 158.15, 150.20, 142.42, 133.91, 132.27, 129.40, 128.99, 127.77, 125.06, 123.28, 118.98, 113.56, 21.97. IR (KBr): 3064, 2966, 2921, 2790, 2225, 1535, 1469, 1428, 854, 759 cm^{-1}. MS (EI, m/z): 245.15 $[M]^+$.

(35) 2,4-diphenylquinazoline **(3ka)**

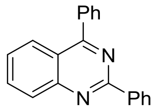

Pale yellow solid, (88.8 mg, 63% Yield), m.p.: 116-118°C (Cheng et al., 2016); ^{1}H NMR (400 MHz, $CDCl_3$) δ 8.69 (d, J = 7.3 Hz, 2H), 8.11 (dd, J = 17.7, 8.4 Hz, 2H), 7.91 – 7.80 (m, 3H), 7.61 – 7.45 (m, 7H). ^{13}C NMR (101 MHz, $CDCl_3$) δ 168.34, 160.29, 152.06, 138.30, 137.76, 133.55, 130.55, 130.25, 129.95, 129.22, 128.75, 128.58, 127.03, 121.74. IR (KBr): 3060, 2966, 1559, 1536, 1440, 1338, 769, 702 cm^{-1}. MS (EI, m/z): 282.15 $[M]^+$.

(36) 6-(2-chloro-4-(trifluoromethyl)phenoxy)-2-phenylquinazoline **(3la)**

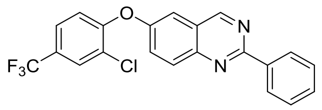

Pale yellow solid, (136 mg, 68% Yield), m.p.: 142-143°C; ^{1}H NMR (400 MHz, $CDCl_3$) δ 9.34 (s, 1H), 8.59 (d, J = 7.1 Hz, 2H), 8.13 (d, J = 9.1 Hz, 1H), 7.82 (s, 1H), 7.68 (d, J = 9.1 Hz, 1H), 7.59 – 7.46 (m, 4H), 7.27 (s, 1H), 7.17 (d, J = 8.5 Hz, 1H). ^{13}C NMR (101 MHz, $CDCl_3$) δ 160.68, 159.57, 154.61, 154.48, 148.06, 137.80, 131.29, 130.67, 128.68, 128.62, 128.58, 128.48, 127.21, 126.69, 125.51 (q, J_{C-F} = 4.0 Hz), 124.08, 120.96, 112.02. IR (KBr): 3060, 1562, 1537, 1486, 1340, 771, 702 cm^{-1}. HRMS (ESI): Calcd. for $C_{21}H_{13}ClF_3N_2O$ $[M+H]^+$: 401.0663; found: 401.0664.

(37) 1-bromo-3,5-diphenylimidazo[1,5-c]quinazoline **(4ja)**

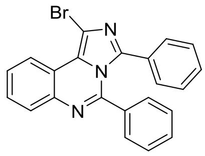

Yellow solid, (95.7 mg, 48% Yield), m.p.: 229 °C; ^{1}H NMR (400 MHz, $CDCl_3$) δ 8.86 (d, J = 7.4 Hz, 1H), 7.89 (d, J = 7.4 Hz, 1H), 7.65 – 7.53 (m, 2H), 7.30 (d, J = 7.7 Hz, 2H), 7.20 – 6.97 (m, 8H). ^{13}C NMR (101 MHz, $CDCl_3$) δ 145.63, 141.80, 138.54, 133.45, 130.55, 130.13, 129.28, 128.87, 128.72, 128.50, 128.48, 128.27, 127.85, 127.54, 125.61, 121.69, 118.93, 109.04. MS (EI, m/z): 399.05 $[M]^+$.

(38) 3,5-diphenyl-1-(phenylethynyl)imidazo[1,5-c]quinazoline **(5ja)**

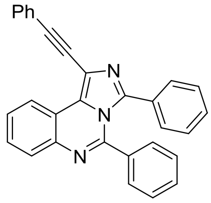

Yellow solid, (151 mg, 72% Yield), m.p.: 201-202°C; ^{1}H NMR (400 MHz, $CDCl_3$) δ 8.95 (d, J = 7.2 Hz, 1H), 7.92 (d, J = 7.1 Hz, 1H), 7.70 (d, J = 6.9 Hz, 2H), 7.66 – 7.57 (m, 2H), 7.45 – 7.37 (m, 3H), 7.33 (d, J = 7.4 Hz, 2H), 7.22 – 7.12 (m, 3H), 7.12 – 6.99 (m, 5H). ^{13}C NMR (101 MHz, $CDCl_3$) δ 146.10, 142.39, 138.90, 133.52, 131.57, 131.51, 130.87, 130.08, 129.31, 129.28, 128.77, 128.66, 128.56, 128.53, 128.42, 128.05, 127.81, 127.49, 123.18, 122.46, 119.61, 116.48, 94.53, 83.93. IR (KBr): 3061, 2219, 1548, 1477, 1330, 758, 697 cm^{-1}. HRMS (ESI): Calcd. for $C_{30}H_{20}N_3$ $[M+H]^+$: 422.1652; found: 422.1649.

Data S4. Cartesian coordinates. Related to **Figure 2**, **Figure 3**, **Figure S99**, **Figure S100** and **Scheme S4**, **Scheme S5**, **Scheme S6**, **Scheme S7** & **Scheme S8**.

69

Ir-O1 SCF Done: E(RM06) = -1490.22403005

C	0.65556600	-1.93376900	-1.77003100
C	-0.73969600	-1.66490900	-1.98479000
C	-1.50812100	-2.38720600	-0.96538800
C	0.76498600	-2.63814500	-0.50448600
C	-0.59464200	-2.95550500	-0.06758300
C	-1.34097600	-1.02188500	-3.20154700
H	-0.64607300	-0.32065600	-3.67298000
H	-1.61076000	-1.77852000	-3.95271200
H	-2.25318800	-0.47040300	-2.95331300
C	-2.99799000	-2.54865800	-0.95585700
H	-3.28965700	-3.44228100	-1.52770800
H	-3.38850100	-2.66338300	0.05928500
H	-3.49915500	-1.69072400	-1.40803600
C	-0.88421300	-3.69010000	1.20135100
H	-0.45951100	-4.70250900	1.17972100
H	-0.42448200	-3.14518600	2.03556000
H	-1.95757700	-3.77630200	1.39228600
C	1.99397000	-3.26943400	0.08485300
H	1.99346400	-4.35820700	-0.06901200
H	2.90807900	-2.88163700	-0.37359200
H	2.05243100	-3.08346300	1.16265500
C	1.77339200	-1.65298600	-2.72979500
H	1.54811000	-0.79843000	-3.37468000
H	2.71232100	-1.43705400	-2.21353900
H	1.94080600	-2.52602800	-3.37663500
C	-4.42583800	0.67558800	0.05254100
C	-2.30937800	1.52996500	-0.23579400
C	-2.83932200	2.83844800	-0.46984800
C	-4.24097500	3.00504400	-0.43793600
C	-5.04851600	1.91971700	-0.17424900
H	-5.03357500	-0.20135500	0.27072800
C	-1.93409000	3.90327400	-0.71657900
H	-4.66032600	3.99201600	-0.61723800
H	-6.12955600	2.00679400	-0.13631100
C	-0.59314200	3.64761300	-0.71326600
C	-0.10681600	2.32919100	-0.47845300
H	-2.31745800	4.90370600	-0.89930900
H	0.11568100	4.44655800	-0.89459200
N	-3.11684800	0.47825000	0.02099300
N	-0.95089900	1.29188200	-0.25047700
C	1.29848200	1.98906600	-0.44272700
C	2.34085200	2.92536300	-0.56779000
C	3.66886600	2.53928100	-0.45344900
C	3.95908700	1.18780200	-0.18614000
C	2.92527200	0.24967000	-0.06170500
C	1.58306700	0.61133500	-0.21370000
H	2.12593000	3.97506100	-0.74748500
H	4.45634100	3.27670700	-0.55400100
H	3.20903300	-0.77194100	0.16540300
Ir	0.01183500	-0.62795300	-0.12390700
O	5.22037400	0.69808000	-0.03426700
C	6.31848700	1.59606100	-0.12929400
H	6.26694300	2.38166500	0.63547200
H	7.21318100	0.99233800	0.03877100
H	6.37997500	2.05872500	-1.12305800
C	-1.67851400	-0.39160200	3.22323900
H	-1.83556400	-1.46554200	3.38284100
H	-2.24044000	-0.10300400	2.32981800
H	-2.10217300	0.14185100	4.08571700
C	-0.17546200	-0.09136700	3.04474400
C	0.05254800	1.43403400	3.00607200
H	-0.54687400	1.91010000	2.22561400
H	-0.22134000	1.89902400	3.96293500
H	1.10797600	1.65183300	2.81010100
C	0.59489300	-0.65879400	4.25916200
H	0.48000700	-1.74796900	4.30631700
H	1.66431600	-0.43628700	4.16660900
H	0.23323000	-0.23384300	5.20516400
O	0.36721400	-0.76092400	1.92891100

18

1a SCF Done: E(RM06) = -551.095580062

C	-1.23151900	-1.36260700	-0.19493900
C	-0.11523500	-0.55241700	0.03399600
C	-0.21816400	0.83818100	0.24400100
C	-1.51230800	1.37344000	0.20371000
C	-2.63652700	0.58434400	-0.03929000
C	-2.49724400	-0.78993800	-0.24163500
H	-1.09027200	-2.42641500	-0.34030000
H	-1.63302700	2.44051500	0.36866500
H	-3.62057100	1.04338400	-0.06885400
H	-3.36529000	-1.41297700	-0.43335100
O	1.18923900	-2.45142700	0.27175600
N	1.18112600	-1.24143000	0.05600700
O	2.21251400	-0.58673800	-0.15254500
C	0.95127800	1.77650300	0.51148100
H	1.60035100	1.36103800	1.29404200
H	0.54884600	2.72195700	0.88662200
O	1.68521300	2.09806900	-0.65823100
H	2.15466900	1.27824100	-0.89064900

87

IN1 SCF Done: E(RM06) = -2041.32132465

C	2.79540100	-0.66819000	-2.22001400
C	2.00676600	0.52486300	-2.40361500
C	0.65792400	0.08049200	-2.61546000
C	1.95608100	-1.83676900	-2.55362500
C	0.65480500	-1.37795200	-2.78811800
C	2.49546200	1.94238600	-2.40620400
H	3.42670100	2.04310300	-1.84134800
H	2.69160200	2.27193600	-3.43631900
H	1.75762500	2.61245500	-1.95804900
C	-0.52596400	0.93760900	-2.93464100
H	-0.72586400	0.88123000	-4.01517300
H	-1.42950300	0.59263600	-2.42195800
H	-0.36028800	1.98362500	-2.67713000
C	-0.54626200	-2.16556900	-3.21646400
H	-0.73334300	-2.01082400	-4.28854100

H	-0.41495100	-3.23922500	-3.05914600
H	-1.45094500	-1.84958400	-2.68735800
C	2.47283100	-3.23482700	-2.70011400
H	2.88697800	-3.36925600	-3.70976100
H	3.27873600	-3.44999400	-1.99312700
H	1.68935200	-3.98490900	-2.56428300
C	4.28248200	-0.72094000	-2.04235000
H	4.65396100	0.16295900	-1.51868100
H	4.59190500	-1.60464300	-1.47559700
H	4.77825100	-0.76487100	-3.02265200
C	-2.14200400	4.87588100	-2.16031000
C	-1.05970900	3.66778600	-0.52794400
C	-2.09070800	3.96122600	0.41843800
C	-3.17973100	4.76151200	-0.00170100
C	-3.20789300	5.22616600	-1.29688400
H	-2.14989800	5.23055300	-3.19073100
C	-1.95290700	3.41990700	1.72275000
H	-3.97549100	4.99622300	0.70174900
H	-4.02204200	5.84398000	-1.66294600
C	-0.84271900	2.67594000	2.02459700
C	0.16152700	2.45775200	1.02560900
H	-2.72887000	3.59877300	2.46375600
H	-0.71634600	2.23134200	3.00420900
N	-1.10978200	4.13180200	-1.80885700
N	0.03329500	2.92076500	-0.20940500
C	1.44189600	1.80390300	1.42916800
C	2.08742000	2.42561400	2.51318000
C	3.32385800	2.01176700	3.00326300
C	3.93416800	0.91336200	2.39396300
C	3.28807900	0.26549400	1.33231800
C	2.04683200	0.67170600	0.81499000
H	1.61602600	3.28923900	2.97687700
H	3.78321400	2.54119100	3.83020000
H	3.79036100	-0.60894000	0.93015700
Ir	1.33980500	-0.60987400	-0.64390900
O	5.14204400	0.39022100	2.76297500
C	5.82591400	0.99419700	3.85056700
H	5.24128400	0.94138100	4.77886500
H	6.75017300	0.42564600	3.97886500
H	6.07694100	2.04301200	3.64203900
C	0.09527600	-3.23633700	2.60274300
H	-0.90794600	-2.80928600	2.68868200
H	0.73957600	-2.73823400	3.33584800
H	0.04088200	-4.30159700	2.85817400
C	0.65838400	-3.04200100	1.18343700
C	2.06925400	-3.64995200	1.12377200
H	2.71915200	-3.16770100	1.86196800
H	2.04369000	-4.72563600	1.33884000
H	2.51669400	-3.51254300	0.13450600
C	-0.26428900	-3.71553000	0.15955500
H	-1.27349400	-3.29132000	0.20420900
H	0.11724300	-3.58192000	-0.85587000
H	-0.34436500	-4.79255800	0.35257100
O	0.75662400	-1.62141700	0.97360400
C	-5.24100600	-1.85077400	3.15232400
C	-4.03441300	-1.32885000	2.69044100
C	-3.80349000	-1.07950400	1.33003900
C	-4.86224300	-1.39539900	0.45099000
C	-6.07588700	-1.92946200	0.90084200
C	-6.27025500	-2.15332600	2.25703100
H	-5.37732800	-2.02362200	4.21658400
H	-3.23057400	-1.10195200	3.38001200
H	-6.84583100	-2.15924800	0.17530900
H	-7.21279100	-2.56093300	2.60962600
C	-2.46141500	-0.48944300	0.91716100
H	-2.04270200	-1.06557600	0.08676100
H	-2.63674600	0.52103100	0.52243000
O	-1.56497600	-0.44607000	2.00631800
H	-0.69474500	-0.79201600	1.69393900
N	-4.74514900	-1.19445900	-0.99668200
O	-3.76761800	-0.57120800	-1.42688500
O	-5.62739900	-1.65238000	-1.72605000

87

TS1 SCF Done: E(RM06) = -2041.30885579

C	2.67403100	-0.66227900	-2.10613200
C	1.79289400	0.44018300	-2.45617000
C	0.49698700	-0.12884200	-2.71119900
C	1.93560000	-1.90526400	-2.35065300
C	0.61325000	-1.58849600	-2.70083300
C	2.17766500	1.87968400	-2.62402400
H	3.03411700	2.14000500	-1.99619700
H	2.45582600	2.07262900	-3.67000400
H	1.35100600	2.54218500	-2.35622500
C	-0.70786700	0.60320700	-3.21170600
H	-0.71729500	0.57596400	-4.31168000
H	-1.63636600	0.13343000	-2.87120600
H	-0.72276900	1.64628300	-2.89552900
C	-0.47980300	-2.53968800	-3.08906000
H	-0.54564800	-2.62400100	-4.18339100
H	-0.30842600	-3.54560700	-2.69457800
H	-1.45967300	-2.20601600	-2.73656400
C	2.55735000	-3.26760200	-2.32482100
H	3.10662300	-3.42726800	-3.26402900
H	3.27860000	-3.37909100	-1.51140800
H	1.81581200	-4.06571400	-2.24022500
C	4.16479500	-0.58164900	-1.95030000
H	4.47546500	0.38127700	-1.53791800
H	4.54858400	-1.36598700	-1.28995700
H	4.65840700	-0.70073400	-2.92577800
C	-3.27220400	4.00356300	-2.49850100
C	-1.84170000	3.29931900	-0.83637300
C	-2.80185400	3.63482800	0.17026100
C	-4.04583900	4.17191100	-0.23677800
C	-4.28741200	4.35959400	-1.57876700
H	-3.44273000	4.14789500	-3.56527300
C	-2.43574200	3.41049600	1.52185600
H	-4.78929100	4.43003100	0.51415700
H	-5.22609600	4.77029300	-1.93750900
C	-1.19371900	2.90563900	1.79946200
C	-0.28671800	2.60107900	0.73067500
H	-3.14220800	3.64104800	2.31621800
H	-0.88386700	2.71838500	2.82198800
N	-2.10114600	3.49700400	-2.16012500
N	-0.61666700	2.78386300	-0.53965300
C	1.09946800	2.17880500	1.08435500

C	1.75261100	3.03977800	1.98648000
C	3.06392300	2.84546500	2.41380400
C	3.74657200	1.72519800	1.93424300
C	3.10118900	0.84862300	1.05263800
C	1.78784900	1.03315100	0.59426700
H	1.22248100	3.91811700	2.34790500
H	3.52318900	3.55287100	3.09482000
H	3.66295200	-0.02584800	0.74465100
Ir	1.11007500	-0.48192400	-0.63305700
O	5.03085500	1.39354400	2.26836700
C	5.72405200	2.23878500	3.17320100
H	5.22180300	2.28996500	4.14876800
H	6.71329700	1.79360700	3.30495400
H	5.83986600	3.25580200	2.77439300
C	0.98580100	-2.97610700	3.20122800
H	-0.08819200	-2.78235600	3.30899800
H	1.52680100	-2.23871800	3.80413900
H	1.19168900	-3.97470000	3.60666700
C	1.41257600	-2.87307400	1.72473600
C	2.92281800	-3.12468700	1.61596600
H	3.47097800	-2.40270300	2.23059700
H	3.18389500	-4.13488500	1.95508700
H	3.26454800	-3.01473200	0.58229700
C	0.62229200	-3.89103200	0.88683700
H	-0.45678400	-3.76519600	1.03924800
H	0.83131600	-3.75804700	-0.17846700
H	0.87738600	-4.92239400	1.16082100
O	1.13904100	-1.53200300	1.31882800
C	-3.75571200	-0.67117900	3.45917500
C	-2.81461800	-0.58062000	2.43459000
C	-3.01167100	-1.18903600	1.18734400
C	-4.21997800	-1.90115500	1.02709000
C	-5.16945700	-2.00685900	2.04950700
C	-4.93998500	-1.38697600	3.27083900
H	-3.56070200	-0.18055900	4.40902000
H	-1.89616500	-0.02622500	2.58281600
H	-6.07331000	-2.57356800	1.86512500
H	-5.67709400	-1.46262300	4.06440600
C	-1.92351900	-1.06316900	0.12477100
H	-1.68532000	-2.05885700	-0.27055500
H	-2.30368600	-0.47587800	-0.71616700
O	-0.77012700	-0.42049300	0.62820600
H	-0.06474900	-1.10295100	1.21898000
N	-4.55348300	-2.57466400	-0.23293500
O	-3.86584400	-2.32981700	-1.22965400
O	-5.51073600	-3.35122200	-0.24538600

72

IN2 SCF Done: E(RM06) = -1807.74615465

C	3.15928400	0.45314100	1.03261300
C	3.16524100	-0.96201500	0.78496000
C	2.40406800	-1.61480800	1.85404000
C	2.23773000	0.69658600	2.12901300
C	1.83518600	-0.61253600	2.65345000
C	4.03570500	-1.68147300	-0.20538300
H	4.24609300	-1.05999500	-1.08062600
H	4.99913600	-1.95908200	0.24645900
H	3.56492400	-2.60381300	-0.55970400
C	2.32547500	-3.09572800	2.06962700
H	2.33102100	-3.64351600	1.12480600
H	3.18972000	-3.44047200	2.65672900
H	1.41882000	-3.37944700	2.60969300
C	0.85970500	-0.78261400	3.77570000
H	1.16282200	-0.20644100	4.65876000
H	-0.12598000	-0.42152900	3.45118400
H	0.75835000	-1.83003200	4.07326000
C	2.02470700	1.99482700	2.85380300
H	2.64696600	2.04973700	3.75909500
H	2.28608200	2.85436300	2.22959800
H	0.98048200	2.11317200	3.16003600
C	4.03976000	1.46830200	0.36745900
H	4.29138700	1.17790000	-0.65676400
H	3.57009700	2.45382100	0.32381000
H	4.98025800	1.57132900	0.92714200
C	-0.46012000	-4.40863500	0.03416400
C	-0.07146300	-2.51601300	-1.20712500
C	-0.49823500	-3.17342200	-2.40310800
C	-0.89692400	-4.52483800	-2.31252200
C	-0.88212600	-5.15390000	-1.08645400
H	-0.44777100	-4.87522900	1.01808300
C	-0.52464700	-2.42892900	-3.61130300
H	-1.22226500	-5.04326500	-3.21104100
H	-1.19023100	-6.18804300	-0.97124000
C	-0.18015300	-1.10793900	-3.58913800
C	0.23107700	-0.49090600	-2.37217400
H	-0.83998800	-2.91261300	-4.53211700
H	-0.22327700	-0.51539600	-4.49474100
N	-0.06297700	-3.14667200	-0.01286200
N	0.32398500	-1.19824000	-1.22303100
C	0.53049500	0.92174600	-2.24358800
C	0.41214600	1.84998400	-3.29266300
C	0.60657200	3.20752300	-3.07433400
C	0.90273400	3.64746500	-1.77092400
C	1.02985800	2.72547700	-0.72165900
C	0.88184300	1.35098600	-0.92825800
H	0.15446800	1.52461400	-4.29688200
H	0.50902500	3.90567400	-3.89708500
H	1.24463300	3.12384600	0.26436800
Ir	1.12085200	-0.09358600	0.44732800
O	1.08814300	4.95261400	-1.43221700
C	0.95465800	5.94707900	-2.44011400
H	-0.05293900	5.94979100	-2.87533800
H	1.13115100	6.90273600	-1.94136400
H	1.69644400	5.81749900	-3.23894300
C	-5.54999300	1.18495800	1.35663600
C	-4.49148800	0.52757200	0.71580200
C	-3.14313900	0.72224400	1.08692000
C	-2.92070100	1.62353200	2.13860100
C	-3.96136500	2.29307700	2.77702600
C	-5.28664100	2.07438900	2.38896400
H	-6.56208800	0.98667000	1.02717300
H	-3.73874400	2.98853700	3.58249300
H	-6.10454600	2.59103800	2.88242900
C	-1.92987200	0.03131600	0.46101700
H	-1.86672700	0.37727700	-0.58276100
H	-2.14956600	-1.04833800	0.39825600

O	-0.77961600	0.29904600	1.18797200
N	-4.86702600	-0.37641600	-0.37492100
O	-3.97864300	-0.80970700	-1.11502400
O	-6.06082400	-0.66448200	-0.51365800
H	-1.88998400	1.77613900	2.43376900

72

TS2 SCF Done: E(RM06) = -1807.69742740

C	-0.86124200	0.75545200	2.65374200
C	0.47530300	0.17093600	2.65327700
C	0.33120700	-1.23550600	2.39801900
C	-1.79795900	-0.31949200	2.40783400
C	-1.06600000	-1.54541600	2.25805900
C	1.74518900	0.87532000	3.02479200
H	1.66487700	1.95195200	2.85130100
H	1.96573700	0.72435300	4.09147800
H	2.58816600	0.50823500	2.43563000
C	1.44136300	-2.23925300	2.36297400
H	2.40177000	-1.78351600	2.11199400
H	1.53782700	-2.71613200	3.34964400
H	1.24390000	-3.03385800	1.63594200
C	-1.64180500	-2.92025400	2.08886500
H	-1.68904300	-3.44384600	3.05452300
H	-2.65789800	-2.88981000	1.68577400
H	-1.03514300	-3.53468500	1.41635800
C	-3.29199300	-0.17623900	2.40817000
H	-3.68073400	-0.26890600	3.43169300
H	-3.60145500	0.79909800	2.02265200
H	-3.77630600	-0.94710700	1.80195400
C	-1.21305700	2.13722200	3.12068100
H	-0.44860100	2.86638600	2.84172300
H	-2.16489700	2.47811400	2.70240600
H	-1.30780800	2.15411800	4.21586600
C	5.38770100	-2.34956900	0.66102800
C	3.97753400	-0.80885300	-0.31053700
C	4.50201200	-1.10533600	-1.60864300
C	5.51853400	-2.08297400	-1.71719200
C	5.96883900	-2.71087000	-0.57764800
H	5.72997600	-2.83616600	1.57430800
C	3.96383900	-0.38923400	-2.70894600
H	5.92943000	-2.32250600	-2.69557600
H	6.74899100	-3.46510400	-0.61245600
C	2.99124600	0.54699600	-2.48218800
C	2.53020200	0.79111900	-1.14673900
H	4.32545100	-0.59550200	-3.71395700
H	2.54863000	1.10090100	-3.30312000
N	4.43710300	-1.44455700	0.80540200
N	3.01066900	0.12556900	-0.10745700
C	1.55169900	1.89565500	-0.92626100
C	1.91733900	3.13376400	-1.48233500
C	1.14038500	4.28397800	-1.35434100
C	-0.06770400	4.18451500	-0.66094500
C	-0.45464700	2.94976000	-0.11956500
C	0.32358300	1.78663100	-0.21107800
H	2.86304300	3.21122000	-2.01422100
H	1.47979400	5.21805500	-1.78745500
H	-1.41854800	2.92460100	0.37833900
Ir	-0.45288600	0.05393900	0.63451400
O	-0.93985400	5.21767700	-0.46272900
C	-0.61525300	6.48325900	-1.01743500
H	-0.52591800	6.43761900	-2.11122600
H	-1.44175100	7.14812200	-0.75508700
H	0.31563700	6.88676500	-0.59618000
C	-4.28003500	-2.70528000	-1.92651000
C	-3.46188600	-1.58045200	-1.76897600
C	-2.06846000	-1.69023800	-1.56459200
C	-1.54174700	-2.99175200	-1.54214800
C	-2.34513200	-4.11659300	-1.70627800
C	-3.72261800	-3.97684900	-1.89983100
H	-5.34300300	-2.55866100	-2.07027900
H	-1.89432500	-5.10509100	-1.69053200
H	-4.35617300	-4.84829600	-2.03328900
C	-1.06685800	-0.55841700	-1.46344600
H	-1.65595500	0.40745800	-0.49208600
H	-1.20205300	0.21229800	-2.23303000
O	0.18416600	-0.88317800	-1.17912600
N	-4.13939300	-0.28129400	-1.80531800
O	-3.44555400	0.74143500	-1.78066400
O	-5.37078700	-0.26297700	-1.85850700
H	-0.47125700	-3.08728100	-1.40169300

72

IN3 SCF Done: E(RM06) = -1807.71120778

C	-0.92972000	0.94462200	2.51188100
C	0.33573400	0.23271800	2.63881000
C	0.08309500	-1.14431200	2.39137400
C	-1.96676500	-0.05381600	2.25645400
C	-1.33301600	-1.32589200	2.15618300
C	1.64218000	0.84323700	3.05010500
H	1.67553200	1.90815800	2.80456500
H	1.77997700	0.74810800	4.13687300
H	2.48373200	0.36093700	2.54799300
C	1.07709400	-2.26250900	2.44640300
H	2.10519400	-1.90795500	2.35178900
H	0.98374100	-2.79674200	3.40361900
H	0.90057900	-2.99472100	1.65117400
C	-2.01153600	-2.65429200	2.00556200
H	-2.08666200	-3.15661800	2.98070700
H	-3.02635400	-2.55220400	1.61252900
H	-1.45863800	-3.32323200	1.33936300
C	-3.44572100	0.20794900	2.24646800
H	-3.86032300	0.07040300	3.25490400
H	-3.67225300	1.22969200	1.93128400
H	-3.97745900	-0.47204700	1.57521200
C	-1.17540800	2.35628100	2.96340000
H	-0.34290700	3.01538700	2.70485100
H	-2.08111800	2.77226200	2.51270100
H	-1.30213200	2.38895900	4.05458500
C	5.03593200	-2.87982700	1.00902100
C	3.83017500	-1.25745700	-0.09554000
C	4.33488600	-1.70248100	-1.35846900
C	5.22937800	-2.79808400	-1.37815100
C	5.58626400	-3.39339900	-0.18912400
H	5.30689000	-3.33688000	1.96070800
C	3.90614600	-1.00410200	-2.51713900
H	5.62260500	-3.15159800	-2.32881200

H 6.27159500 -4.23475600 -0.15571700
C 3.05413400 0.05765200 -2.37651500
C 2.60454100 0.44379900 -1.07165200
H 4.25718600 -1.32077800 -3.49684600
H 2.69978000 0.60908600 -3.24089400
N 4.19601300 -1.86261800 1.07056700
N 2.97716300 -0.20323300 0.02158300
C 1.79498300 1.69202200 -0.94034100
C 2.40767100 2.83495800 -1.48249100
C 1.85205600 4.11014800 -1.40178100
C 0.61818800 4.24420500 -0.76322600
C -0.02034700 3.10832700 -0.24563800
C 0.52507200 1.81636800 -0.30610800
H 3.37829600 2.72814800 -1.96171800
H 2.37801500 4.95906200 -1.82336500
H -0.99311200 3.27105300 0.20529600
Ir -0.59223200 0.25270000 0.48473000
O -0.05149000 5.42415700 -0.60232700
C 0.53854900 6.60244000 -1.13029700
H 0.66838600 6.53954400 -2.21909000
H -0.15403000 7.41561500 -0.90039000
H 1.50948300 6.81592600 -0.66306500
C -4.34243600 -2.54814300 -1.81527500
C -3.46163700 -1.46687500 -1.70612300
C -2.06862700 -1.64319000 -1.55135800
C -1.61017200 -2.97330300 -1.52280300
C -2.47491700 -4.05571000 -1.65383000
C -3.84972100 -3.84706100 -1.80290400
H -5.40239600 -2.34903300 -1.91402200
H -2.07548600 -5.06627600 -1.64525800
H -4.53089300 -4.68573300 -1.90929700
C -1.03922000 -0.55914200 -1.46166600
H -1.71769100 1.21407500 -0.06102500
H -1.16034100 0.26194900 -2.16764100
O 0.18853800 -0.88579500 -1.11817300
N -4.07517000 -0.13569400 -1.72367800
O -3.37526800 0.83118600 -2.03151300
O -5.27192600 -0.04817200 -1.43159700
H -0.54324800 -3.12868700 -1.40701700

72
Ir-H SCF Done: E(RM06) = -1807.73824524
C 1.11652700 1.74831000 2.15598100
C 1.91752400 2.46668300 1.17772000
C 3.03031200 1.64512800 0.83320000
C 1.84419500 0.52998500 2.49225900
C 2.99756000 0.46285100 1.68164900
C 1.67238700 3.87089400 0.70855900
H 2.06941100 4.59069300 1.43890600
H 2.16015900 4.07064200 -0.25046800
H 0.60760500 4.08511300 0.58358500
C 4.15126500 1.99551400 -0.10346800
H 4.51518300 1.11405900 -0.64212500
H 3.83838900 2.73137100 -0.85007900
H 5.00815600 2.42039500 0.44067100
C 4.09821500 -0.54989200 1.78149400
H 3.73074300 -1.51394200 2.14246300
H 4.58555900 -0.71800200 0.81827100
H 4.86951400 -0.20256500 2.48589900
C 1.45351800 -0.42873900 3.57941500
H 1.80486700 -0.06404700 4.55632600
H 0.37002300 -0.55542800 3.64384200
H 1.88877700 -1.41924200 3.41895400
C 0.00071200 2.32435900 2.98288200
H -0.71753000 1.55337900 3.27768600
H 0.39004600 2.78661700 3.90241500
H -0.54960800 3.09977100 2.44208500
C 3.39428800 -3.20794700 -0.59943300
C 1.92862400 -1.73661500 -1.58622800
C 1.96067300 -2.48075000 -2.80854200
C 2.77937700 -3.62886300 -2.86724800
C 3.50615200 -4.00371700 -1.75714300
H 3.94909100 -3.47868100 0.29800900
C 1.15953700 -2.03971600 -3.89507500
H 2.81711500 -4.20443900 -3.78906200
H 4.14330100 -4.88238800 -1.75991100
C 0.37141000 -0.93793000 -3.73164300
C 0.36747100 -0.22404100 -2.49762100
H 1.17486100 -2.59001400 -4.83230300
H -0.26376000 -0.59456400 -4.53951100
N 2.64568700 -2.11999900 -0.51004300
N 1.15230600 -0.60409300 -1.45828600
C -0.47534600 0.91787100 -2.23331400
C -1.35972300 1.47853500 -3.17342300
C -2.18426500 2.54194500 -2.83882700
C -2.13435300 3.04545500 -1.52339200
C -1.25988000 2.48987700 -0.58201000
C -0.38805000 1.43773200 -0.90307500
H -1.41316100 1.08713400 -4.18586900
H -2.85458900 2.96164000 -3.57949600
H -1.29135400 2.90359500 0.41982900
Ir 0.97590200 0.58613000 0.29602300
H -0.20541400 -0.45126400 0.62652700
O -2.90281700 4.07778700 -1.07840300
C -3.86085900 4.64566200 -1.96128700
H -4.59524600 3.90124300 -2.29457900
H -4.37377900 5.42063200 -1.38717800
H -3.38476200 5.10585800 -2.83726700
C -3.01475500 -3.83329900 1.19102100
C -2.99730500 -2.46349400 1.44547500
C -3.83000500 -1.56395000 0.75637400
C -4.71327200 -2.10158700 -0.18869500
C -4.75526300 -3.47092800 -0.44451800
C -3.90017500 -4.33732400 0.24041500
H -2.35418400 -4.48531500 1.74919000
H -5.35646800 -1.41313000 -0.72692200
H -5.45071200 -3.86185500 -1.18149700
H -3.92537100 -5.40485200 0.04405500
C -3.79297100 -0.06824600 0.88448200
H -2.84535700 0.38089600 1.21136800
O -4.75055000 0.61705100 0.57848700
N -2.11591500 -1.99813000 2.52573300
O -2.40629200 -0.93703400 3.08492400
O -1.16054400 -2.70684700 2.83557400

72

TS3 SCF Done: E(RM06) = -1807.71121694

C	3.04265000	0.25970500	-1.29602900
C	3.38300400	0.88335200	-0.03676700
C	2.73068900	2.18362900	0.00203100
C	2.16757400	1.16729800	-1.99722100
C	2.00328800	2.36107100	-1.21219400
C	4.38486300	0.37373400	0.95566600
H	5.40104800	0.69489300	0.68225900
H	4.18015300	0.74839300	1.96300800
H	4.38320100	-0.71883300	1.00279100
C	2.89481300	3.19048200	1.09989100
H	2.02637100	3.84864600	1.16908600
H	3.02429900	2.70530300	2.07248400
H	3.78319700	3.81403900	0.92226400
C	1.28837000	3.59151900	-1.68489700
H	0.28412200	3.35800300	-2.04787300
H	1.19768500	4.33321000	-0.88913300
H	1.84446700	4.05888000	-2.51109700
C	1.62250400	0.96841700	-3.37777900
H	2.15239600	1.62218200	-4.08572600
H	1.75449300	-0.06282400	-3.71613100
H	0.55491000	1.20735400	-3.42727900
C	3.69551800	-0.95029800	-1.90095700
H	2.97958800	-1.58413300	-2.43310300
H	4.46503800	-0.64258600	-2.62311700
H	4.19234700	-1.56413900	-1.14513800
C	-1.12270200	4.20278900	1.02335400
C	-1.00275200	1.95661500	1.49682800
C	-2.12790500	2.05434600	2.37512100
C	-2.73715000	3.31630000	2.53815200
C	-2.23895800	4.40648600	1.85609500
H	-0.70080500	5.04170600	0.47142400
C	-2.58183300	0.88416300	3.03971100
H	-3.59523200	3.40610800	3.19980100
H	-2.68174800	5.39278300	1.95053700
C	-1.92654100	-0.29468800	2.82695700
C	-0.81362500	-0.35876900	1.94541500
H	-3.43683400	0.94252400	3.70786700
H	-2.25336300	-1.19928400	3.32513300
N	-0.52073600	3.03494200	0.85032800
N	-0.36921800	0.74478400	1.27716800
C	-0.07955400	-1.57011200	1.66880600
C	-0.35779500	-2.81190100	2.26617900
C	0.37973900	-3.94305900	1.94732700
C	1.41462000	-3.82803900	1.00081200
C	1.69462400	-2.59370300	0.39944400
C	0.97160300	-1.43352700	0.70899200
H	-1.16100500	-2.91223100	2.99073600
H	0.14581000	-4.89082300	2.41698000
H	2.49374900	-2.58269100	-0.33146700
Ir	1.21938600	0.41867800	-0.05283100
H	-0.09755200	-0.33546600	-1.29412900
O	2.20239600	-4.86595600	0.60593100
C	1.94606900	-6.15951500	1.13889700
H	0.93206600	-6.50288300	0.89742200
H	2.67143700	-6.82572200	0.66639000
H	2.09120100	-6.18638000	2.22682600
C	-4.08178800	0.68190500	-1.55917200
C	-3.12041900	-0.34102100	-1.70338000
C	-3.46922300	-1.68957100	-1.40490500
C	-4.75701300	-1.93444800	-0.89672500
C	-5.69175800	-0.92191400	-0.72576100
C	-5.34746900	0.39222200	-1.07425200
H	-3.79290100	1.69304300	-1.81524200
H	-5.00445900	-2.96587400	-0.66635100
H	-6.68239900	-1.14907700	-0.34277300
H	-6.06932600	1.19637500	-0.95835000
C	-2.63769500	-2.89236500	-1.68051300
H	-1.78538000	-2.76160200	-2.36198700
O	-2.90583700	-3.99682700	-1.22872400
N	-1.85727500	0.05393700	-2.17688700
O	-0.83807000	-0.78727500	-2.02852000
O	-1.59763500	1.25513800	-2.47833300

72

IN4 SCF Done: E(RM06) = -1807.75190479

C	-1.64370400	0.50954800	-2.49714900
C	-1.70143500	-0.92606600	-2.46025500
C	-0.36033500	-1.44095300	-2.75751000
C	-0.24523000	0.88231800	-2.58066700
C	0.51764600	-0.35045300	-2.80784200
C	-2.95068200	-1.76025600	-2.44608800
H	-3.28779400	-1.98118200	-3.46910900
H	-2.78766000	-2.71811700	-1.94164200
H	-3.76814700	-1.24824000	-1.93012500
C	-0.03827800	-2.87840100	-3.02689800
H	1.01527500	-3.10216700	-2.84069300
H	-0.63716000	-3.55106300	-2.40877200
H	-0.24898800	-3.12073500	-4.07920200
C	1.99596100	-0.37814100	-3.04126100
H	2.51032100	0.26714500	-2.32215700
H	2.40664400	-1.38731200	-2.94881800
H	2.23414300	-0.00709200	-4.04812000
C	0.29916200	2.26253300	-2.81046300
H	0.42094900	2.46207800	-3.88533600
H	-0.36658700	3.03179000	-2.40880000
H	1.27454300	2.38913100	-2.33165300
C	-2.82177000	1.43445800	-2.57276900
H	-2.59808400	2.42412400	-2.16864800
H	-3.11790900	1.56476200	-3.62320800
H	-3.68611300	1.04176500	-2.02938100
C	0.76715000	-4.43355200	0.23900300
C	-0.57686600	-2.76602900	1.06875200
C	-0.94446500	-3.57624200	2.18861300
C	-0.40296700	-4.87717900	2.27513100
C	0.46314100	-5.31581500	1.29681600
H	1.45113000	-4.75042300	-0.54696500
C	-1.81499600	-3.02282300	3.16396600
H	-0.67161500	-5.50877200	3.11824700
H	0.90661200	-6.30573900	1.32803800
C	-2.24346300	-1.73428900	3.02344600
C	-1.84152400	-0.95994700	1.89493100
H	-2.11225400	-3.62330600	4.01988200
H	-2.88270900	-1.28513300	3.77354300
N	0.27093900	-3.21237100	0.11647800
N	-1.06332500	-1.48959100	0.92693000

C	-2.17939200	0.43801900	1.71512000
C	-2.94774700	1.18174000	2.62811500
C	-3.14210300	2.54562600	2.45706200
C	-2.53204200	3.18263400	1.36084500
C	-1.76914700	2.44388300	0.44340000
C	-1.60419100	1.06290700	0.56895000
H	-3.40018200	0.70403300	3.49276700
H	-3.73909900	3.09984700	3.17146300
H	-1.30076100	2.99327900	-0.36556800
Ir	-0.60189500	-0.14341000	-0.69124000
H	1.24692400	2.18586300	1.47158600
O	-2.62648400	4.51518500	1.10700200
C	-3.36570800	5.33316900	2.00592000
H	-2.94211200	5.30718500	3.01804700
H	-3.29171100	6.34966000	1.61342500
H	-4.42275600	5.03951000	2.04508900
C	3.51822500	-0.64997100	1.09710900
C	3.29217200	0.65948400	0.64580800
C	4.37168700	1.57867700	0.61968000
C	5.62172100	1.17587600	1.11733300
C	5.83405100	-0.11311900	1.59142600
C	4.77585900	-1.02745100	1.56286900
H	2.69320600	-1.34931100	1.08653500
H	6.42522100	1.90608500	1.09864800
H	6.81070800	-0.40875400	1.96439500
H	4.92566300	-2.04447400	1.91772400
C	4.28871100	2.92756300	0.00644300
H	3.45329500	3.09475000	-0.69274500
O	5.12076500	3.80381100	0.19141700
N	2.00576100	1.02677100	0.14352700
O	1.67189900	2.36810100	0.60538700
O	1.03664900	0.16955100	0.59862900

15

t-BuOH SCF Done: E(RM06) = -233.585121245

C	0.68230500	1.26641400	-0.51554700
H	1.74205100	1.28398000	-0.22710200
H	0.20416300	2.16186300	-0.10328900
H	0.63689200	1.32275200	-1.60978600
C	-0.00598600	-0.00002600	0.01419500
C	-1.49358100	-0.00356100	-0.34475300
H	-1.98910900	0.88174100	0.06901200
H	-1.63488800	-0.00350800	-1.43104600
H	-1.98479100	-0.89157000	0.06836100
C	0.68858500	-1.26278700	-0.51610700
H	1.74847300	-1.27505500	-0.22787200
H	0.21508500	-2.16079600	-0.10406400
H	0.64327800	-1.31899400	-1.61035400
O	0.02842200	-0.00027500	1.45393000
H	0.96353300	0.00155300	1.71797100

87

IN5 SCF Done: E(RM06) = -2041.35886610

C	-1.47080900	-1.58336000	-2.41450600
C	-2.14105700	-2.39965000	-1.41228200
C	-1.11555400	-3.03206000	-0.63067600
C	-0.04437000	-1.83771100	-2.29727900
C	0.18465500	-2.72641100	-1.21895400
C	-3.61958000	-2.63076800	-1.30745900
H	-4.18906700	-1.73168200	-1.55975600
H	-3.93211900	-3.42753600	-1.99724400
H	-3.91094400	-2.93417400	-0.29743000
C	-1.34587400	-4.00651700	0.48762600
H	-1.43015300	-5.02979300	0.09569700
H	-0.51938800	-3.99851900	1.20380200
H	-2.26708700	-3.78283500	1.03390300
C	1.49471700	-3.34936400	-0.83440900
H	1.66866800	-4.26199300	-1.42389300
H	2.34055100	-2.67860500	-1.01162400
H	1.50793400	-3.63708200	0.21939400
C	0.97661200	-1.17565600	-3.16825200
H	0.79083300	-1.41201100	-4.22427200
H	0.91383000	-0.08688200	-3.04756600
H	1.99693300	-1.47211000	-2.91828700
C	-2.11613300	-0.90969800	-3.59183600
H	-3.16360800	-0.66500600	-3.39604700
H	-1.59793100	0.01546200	-3.86352900
H	-2.09634500	-1.56919100	-4.47164300
C	2.30538000	-2.01258900	2.60886900
C	0.20388800	-1.06186700	2.49749700
C	0.09585400	-1.07759300	3.92296000
C	1.15132300	-1.64784600	4.66670400
C	2.26184400	-2.13567200	4.01050400
H	3.18942700	-2.33050200	2.05991200
C	-1.05263900	-0.49159700	4.51677100
H	1.07704000	-1.67993700	5.75068400
H	3.09629600	-2.57521100	4.54639600
C	-1.99551800	0.08297000	3.71445600
C	-1.86340300	0.03773500	2.29540600
H	-1.15615900	-0.48961200	5.59847600
H	-2.86482000	0.56170200	4.14837600
N	1.32309800	-1.50324900	1.87562000
N	-0.80898100	-0.57399500	1.70672200
C	-2.81624800	0.63271800	1.38188100
C	-3.90731100	1.41987000	1.79064600
C	-4.73178500	2.03838800	0.86083400
C	-4.44776300	1.87689700	-0.50756500
C	-3.36441900	1.08509100	-0.92048300
C	-2.54185800	0.43036600	-0.00190200
H	-4.11785400	1.57546500	2.84498300
H	-5.56444900	2.64479200	1.19648700
H	-3.18836500	1.00813400	-1.98784400
Ir	-0.99381700	-0.79230600	-0.43446700
O	-5.17225200	2.44801700	-1.50608000
C	-6.27208100	3.28331100	-1.16338100
H	-5.95199800	4.14990000	-0.57091400
H	-6.68814600	3.63166600	-2.11119400
H	-7.04473200	2.73041500	-0.61373600
H	1.94217100	-0.26804200	0.55627900
C	2.43604900	3.27117300	0.69182400
C	1.38641600	2.70048600	-0.07340600
C	0.69242300	3.50565200	-0.98885300
C	1.05880400	4.83696600	-1.17471100
C	2.12233700	5.39621300	-0.45884300
C	2.79691200	4.60942600	0.46728200
H	0.51226200	5.43977100	-1.89606700

H	2.40845300	6.43276400	-0.61355600
H	3.61124300	5.01446800	1.06053700
N	0.99162700	1.34540600	0.12992800
O	0.11175300	0.93351800	-0.83614600
O	2.21746400	0.52974900	0.04459700
C	5.36168600	0.95864100	-2.33868200
H	4.74838500	1.69485000	-1.80514300
H	4.98520400	0.88268900	-3.36522400
H	6.38873500	1.34241900	-2.37487500
C	5.29649300	-0.41039200	-1.64451500
C	6.12234300	-1.44659100	-2.41163900
H	5.76244800	-1.53502700	-3.44306300
H	7.18163400	-1.16665800	-2.43766200
H	6.03734700	-2.43144200	-1.93776300
C	5.77506300	-0.30493000	-0.18774700
H	5.16057700	0.40906400	0.37283500
H	5.70236900	-1.28115900	0.30671700
H	6.81746600	0.03150300	-0.12894700
O	3.94702600	-0.90192100	-1.67481300
H	3.36002800	-0.24373400	-1.24548900
H	-0.11880300	3.06541700	-1.55245300
C	3.11960200	2.55427200	1.79246900
H	2.62273600	1.63961900	2.15397700
O	4.14439600	2.94933900	2.33053500

87

TS4 SCF Done: E(RM06) = -2041.31614895

C	2.42943500	-0.50009300	-2.32562500
C	1.23293200	0.25145200	-2.67793200
C	0.12934200	-0.68699700	-2.69769400
C	2.03111200	-1.88101100	-2.14871400
C	0.62828900	-2.00633700	-2.39910300
C	1.18768300	1.67681100	-3.13997400
H	2.01760500	2.25355100	-2.72230100
H	1.26479400	1.72137800	-4.23577000
H	0.25905600	2.16180000	-2.83116800
C	-1.28500100	-0.37770800	-3.07196500
H	-1.47358000	-0.72614400	-4.09767400
H	-1.98927100	-0.89601400	-2.41323900
H	-1.50377600	0.69042600	-3.02577600
C	-0.15806300	-3.28263700	-2.42289100
H	-0.14171900	-3.73758800	-3.42378800
H	0.25278600	-4.02440100	-1.72986200
H	-1.20798900	-3.11283900	-2.16637600
C	2.93180900	-3.01926800	-1.78108200
H	3.07559400	-3.67697600	-2.64887000
H	3.91591300	-2.67243500	-1.45736800
H	2.49881400	-3.61246900	-0.97095700
C	3.83988700	0.00265500	-2.41185800
H	3.90767300	1.06578100	-2.16960400
H	4.51032200	-0.53565400	-1.73526500
H	4.22447000	-0.13087800	-3.43304200
C	-4.06803800	2.86360600	-2.53224800
C	-2.41622700	2.67166400	-0.93858400
C	-3.32238300	3.08151300	0.09040300
C	-4.65858700	3.37918300	-0.26706900
C	-5.03834500	3.27151200	-1.58552300
H	-4.34948900	2.77469600	-3.58133300
C	-2.81357900	3.16234500	1.41142200
H	-5.36286100	3.68885200	0.50181600
H	-6.05189400	3.49043700	-1.90678500
C	-1.49642200	2.86271000	1.63867300
C	-0.65051400	2.47230600	0.54801600
H	-3.47353100	3.45436200	2.22524200
H	-1.08246700	2.90615500	2.64046400
N	-2.81377700	2.57407800	-2.23998500
N	-1.11325600	2.37338700	-0.69080400
C	0.80798700	2.28596600	0.80262700
C	1.40703800	3.34462300	1.50982400
C	2.77569300	3.42133600	1.76019000
C	3.58149300	2.38860000	1.27907400
C	2.99302700	1.31511500	0.59464800
C	1.61681900	1.19846200	0.34463600
H	0.78337800	4.17029100	1.84433500
H	3.18353500	4.27244100	2.29347600
H	3.67151300	0.54111600	0.25737800
Ir	1.01403400	-0.51901800	-0.66530400
O	4.93871500	2.32523600	1.42460200
C	5.58692900	3.38625500	2.11038300
H	5.23974700	3.47154300	3.14883600
H	6.65139800	3.13990000	2.10959900
H	5.44133400	4.34851000	1.60125900
H	-0.88709900	-3.51521900	0.18172700
C	-3.88896900	-1.89353000	1.03359400
C	-2.68614200	-1.21517000	1.32897900
C	-2.55149200	-0.51256400	2.53183100
C	-3.60740400	-0.48251200	3.43827400
C	-4.80718700	-1.14654200	3.15273600
C	-4.94234500	-1.84487100	1.95724100
H	-3.49523700	0.05686500	4.37500900
H	-5.62778200	-1.12008300	3.86413600
H	-5.86026500	-2.37016300	1.71332600
N	-1.64243200	-1.26011300	0.33794900
O	-0.72995100	-0.41306600	0.57442900
O	-0.80574900	-3.02990700	1.01943100
C	1.51664300	-1.10250900	3.23407000
H	0.44190000	-1.25269500	3.38343200
H	1.67828600	-0.08416000	2.87020500
H	2.00939900	-1.20007500	4.21007700
C	2.06987100	-2.13573000	2.24129900
C	3.57821200	-1.94919500	2.04581200
H	3.81482400	-0.93906000	1.70762700
H	4.11050700	-2.11816100	2.99019300
H	3.96151000	-2.66445800	1.30930700
C	1.80917400	-3.55983100	2.77272900
H	0.73786000	-3.74605100	2.89342400
H	2.20757300	-4.30837600	2.07735900
H	2.29957500	-3.70325200	3.74331800
O	1.40256600	-2.03464000	0.96263200
H	0.34960000	-2.54296200	1.01129900
H	-1.61852600	-0.00461600	2.74062200
C	-4.07495900	-2.66731900	-0.21800400
H	-3.21491300	-2.68326900	-0.90897100
O	-5.10423200	-3.26149100	-0.49437200

87

IN6 SCF Done: E(RM06) = -2041.33327065

C	2.49424900	-0.99165500	-2.06057300
C	1.48899700	-0.08207100	-2.59070800
C	0.24242000	-0.79553100	-2.62884300
C	1.85386000	-2.29965700	-1.91337300
C	0.49060000	-2.18289200	-2.25211500
C	1.73047300	1.29830600	-3.12308100
H	2.57605600	1.77801600	-2.62402700
H	1.95645500	1.24959500	-4.19759800
H	0.85454500	1.93709800	-2.98567400
C	-1.05565400	-0.28029200	-3.16394200
H	-1.16988300	-0.58234500	-4.21549200
H	-1.90534400	-0.69028000	-2.61037500
H	-1.12603800	0.80571900	-3.10496700
C	-0.52154900	-3.29114100	-2.23055700
H	-1.50334900	-2.93614600	-2.55412700
H	-0.22393800	-4.09631900	-2.91582600
H	-0.62033500	-3.71340800	-1.22402300
C	2.53686200	-3.52212900	-1.39024500
H	1.93284500	-4.41877500	-1.55616100
H	3.51071200	-3.66909600	-1.87164500
H	2.69921400	-3.40512800	-0.31057800
C	3.98225900	-0.78842000	-2.07940900
H	4.25112900	0.27037100	-2.06219400
H	4.46788200	-1.27486400	-1.22744500
H	4.41112300	-1.21988100	-2.99522700
C	-4.26301000	2.62795900	-2.54754800
C	-2.53375600	2.53990300	-1.03075000
C	-3.39498300	2.99127000	0.02036400
C	-4.75249900	3.25183200	-0.28546000
C	-5.19362700	3.07031100	-1.57566600
H	-4.59368800	2.48297600	-3.57582000
C	-2.82613400	3.14891800	1.30908500
H	-5.42307100	3.59211700	0.50045000
H	-6.22500500	3.25798300	-1.85755800
C	-1.49245300	2.88595200	1.48632200
C	-0.69519600	2.45496400	0.37606900
H	-3.45152400	3.47464600	2.13732100
H	-1.02631100	2.99841100	2.45953200
N	-2.99222800	2.37022300	-2.30367200
N	-1.21410200	2.27273300	-0.83040900
C	0.78317600	2.34543600	0.55621100
C	1.37506400	3.51890800	1.05891700
C	2.75163700	3.68587100	1.19170500
C	3.57119400	2.62369800	0.80870500
C	2.99229600	1.43396300	0.34340600
C	1.61056300	1.23205000	0.21346700
H	0.73246300	4.35686300	1.31886200
H	3.15245400	4.62114000	1.56508000
H	3.68330500	0.63989000	0.09442500
Ir	1.01976500	-0.61855700	-0.51665500
O	4.93650100	2.63351400	0.86052400
C	5.57836600	3.80846100	1.33432600
H	5.29978700	4.03318700	2.37266800
H	6.65084700	3.60491100	1.28933800
H	5.35162600	4.67827000	0.70321600
H	0.32645200	-4.58403100	1.07587600
C	-4.00751500	-1.65456600	0.94772500
C	-2.77524100	-1.02967900	1.28031100
C	-2.61328300	-0.42381100	2.54371500
C	-3.65684200	-0.44163200	3.45774900
C	-4.87628800	-1.05553600	3.13232100
C	-5.04482900	-1.65352100	1.88670300
H	-3.52600000	0.02054900	4.43221800
H	-5.68743600	-1.06764300	3.85475900
H	-5.97824100	-2.13726800	1.61764800
N	-1.77133000	-1.07097700	0.30286700
O	-0.69308700	-0.51936000	0.65430700
O	-0.21011200	-3.77685200	1.04721900
C	1.30687900	-0.62804500	3.22632400
H	0.29165700	-1.02808900	3.32000800
H	1.25410300	0.34201700	2.72464200
H	1.70263700	-0.47031300	4.23844600
C	2.19469200	-1.60302400	2.43595300
C	3.65872400	-1.12302400	2.45372600
H	3.74977100	-0.08636500	2.12732700
H	4.07917300	-1.19145200	3.46574700
H	4.26946700	-1.75001600	1.79295700
C	2.14676100	-2.98724900	3.12505100
H	1.12920900	-3.38903800	3.12537600
H	2.79818800	-3.69856700	2.60184400
H	2.49173100	-2.91728500	4.16423000
O	1.72196200	-1.81093100	1.10421900
H	0.46663600	-3.04992400	1.07441100
H	-1.66855300	0.04682000	2.78305000
C	-4.23341300	-2.32628100	-0.36009800
H	-3.37215700	-2.35976400	-1.04562600
O	-5.30322200	-2.82329300	-0.67112800

15

1a-2 SCF Done: E(RM06) = -474.675779651

C	1.03180400	-1.33220900	0.01250300
C	-0.12818600	-0.55552900	-0.06032800
C	-0.04526900	0.85525900	-0.07575200
C	1.21857900	1.45458500	-0.03320100
C	2.37567100	0.67787700	0.04634000
C	2.28083700	-0.71682200	0.06992000
H	0.93154300	-2.41249200	-0.00303400
H	1.29077700	2.53938500	-0.04280100
H	3.34908600	1.15787100	0.08417200
H	3.18088200	-1.32285600	0.12040100
O	-1.48744900	-2.33165900	0.11612600
N	-1.41884300	-1.17394500	-0.26705700
C	-1.23395400	1.74531800	-0.07260300
H	-1.00135000	2.79553700	-0.35269200
O	-2.36454200	1.41782000	0.22663300

3

H2O SCF Done: E(RM06) = -76.4222760449

O	0.00000000	0.00000000	0.12002100
H	0.00000000	-0.76170600	-0.48008200
H	0.00000000	0.76170600	-0.48008200

15

t-BuONa SCF Done: E(RM06) = -395.287318925

C	1.15477700	0.97578400	1.07835800

H 0.78239700 0.67710900 2.06706300
H 0.78653500 1.99032700 0.87676800
H 2.25264300 1.01348700 1.12360200
C 0.61924400 -0.00014500 -0.00004100
C 1.15334600 0.44648300 -1.38434200
H 0.77937300 1.45189100 -1.61799100
H 2.25111500 0.46753800 -1.44129400
H 0.78392000 -0.23537900 -2.16133900
C 1.15376100 -1.42245200 0.30535200
H 0.78116600 -1.75678700 1.28251700
H 0.78413800 -2.12781100 -0.45059700
H 2.25154100 -1.48066400 0.31993300
O -0.75527600 0.00000000 0.00013600
Na -2.71794400 0.00020700 0.00038900

18
IN7 SCF Done: E(RM06) = -712.810324879
C 1.51910700 1.43002600 -0.06034500
C 0.52865900 0.45365700 0.03590400
C 0.79195800 -0.91702600 0.06458400
C 2.14084800 -1.28923700 0.01870000
C 3.15949300 -0.33842700 -0.05472700
C 2.85163000 1.02459200 -0.09939400
H 1.24802500 2.48098200 -0.09268500
H 2.38808900 -2.34872100 0.01942000
H 4.19649000 -0.66095700 -0.09657200
H 3.64011000 1.76787000 -0.17098100
O -1.37215800 1.51305600 -0.82577800
N -0.85818600 0.93242900 0.14581000
O -1.40265900 0.89362200 1.26127200
C -0.36494600 -1.92900100 0.09151000
H -0.41610000 -2.30695300 1.14322300
H -0.00713100 -2.80503800 -0.49660600
O -1.52853700 -1.40112100 -0.36027000
Na -3.12507600 -0.11874100 -0.17324100

18
TS5 SCF Done: E(RM06) = -712.755776275
C 1.72815600 1.19925300 -0.47386300
C 0.52561100 0.52772100 -0.11530700
C 0.52081700 -0.88201600 0.17223200
C 1.79489900 -1.50112500 0.29559200
C 2.95657100 -0.84219200 -0.04162200
C 2.91792800 0.51530300 -0.45861600
H 1.67698300 2.25634900 -0.70427400
H 1.82293200 -2.55145200 0.57533700
H 3.90543800 -1.37137000 -0.01953600
H 3.83796600 1.02270100 -0.73459300
O -0.51576900 2.56193900 0.33783500
N -0.55336900 1.30705400 0.25289500
O -1.58734500 0.65307900 0.79888400
C -0.69263100 -1.66614700 0.41643300
H -1.09508300 -0.52008100 1.16882100
H -0.49570800 -2.56239000 1.03090900
O -1.72631500 -1.72971600 -0.38514700
Na -3.05923800 -0.12821100 -0.71532800

3
NaOH SCF Done: E(RM06) = -238.118923163
Na 0.04216600 -0.92733100 0.00000000
O 0.04216600 1.08048800 0.00000000
H -0.80116000 1.55674300 0.00000000

16
2a SCF Done: E(RM06) = -346.623269538
C 1.61938200 1.20796900 -0.07982200
C 0.24464900 1.20556300 0.16211200
C -0.45759700 0.00048000 0.28975400
C 0.24387600 -1.20512600 0.16270100
C 1.61859800 -1.20852900 -0.07922300
C 2.30959800 -0.00052900 -0.20017600
H 2.15146500 2.15130700 -0.17253700
H -0.29076300 2.14787800 0.25479100
H -0.29213300 -2.14705600 0.25584600
H 2.15007900 -2.15225400 -0.17145400
H 3.38065100 -0.00092000 -0.38559200
C -1.94906300 0.00098500 0.51700000
H -2.23538200 -0.88554500 1.10408500
H -2.23514000 0.88922700 1.10161400
O -2.60550800 -0.00073000 -0.75485600
H -3.56137400 -0.00166700 -0.58198600

85
IN8 SCF Done: E(RM06) = -1836.83696080
C -2.60767100 -0.31319200 -1.98166300
C -1.47837200 -1.20500100 -2.22516200
C -0.40685500 -0.38497500 -2.70497600
C -2.26276100 0.99767800 -2.55498900
C -0.92625600 0.96395400 -2.96418000
C -1.46510000 -2.69864400 -2.09079500
H -2.18609500 -3.04004400 -1.34331400
H -1.72921300 -3.16447000 -3.05091900
H -0.47760600 -3.05795700 -1.79061100
C 0.92751700 -0.85902700 -3.19129100
H 0.88867100 -1.00399400 -4.28147100
H 1.71715000 -0.12683800 -2.99171700
H 1.22753400 -1.79959100 -2.73078900
C -0.14356300 2.06390400 -3.61695200
H -0.12023700 1.92813500 -4.70769100
H -0.57463600 3.04936000 -3.41744400
H 0.89675700 2.07664500 -3.27732200
C -3.23813600 2.11688700 -2.75324600
H -3.84664600 1.90786400 -3.64524800
H -3.92975200 2.22148700 -1.91416200
H -2.74443900 3.07906700 -2.91267900
C -4.00950500 -0.74620800 -1.66387200
H -4.02162700 -1.65701400 -1.06069000
H -4.55856800 0.02467500 -1.11396400
H -4.56505300 -0.95249800 -2.59036000
C 4.49795200 -3.08518200 -2.17823800
C 2.97747100 -2.52554000 -0.54133000
C 4.02862000 -2.35162500 0.41399400
C 5.36314900 -2.56261800 -0.00569300
C 5.60467100 -2.93256000 -1.30935400
H 4.66793700 -3.38112700 -3.21344900
C 3.65866900 -1.98353800 1.73300700

H	6.17521400	-2.43277400	0.70629900
H	6.61158400	-3.10781500	-1.67542800
C	2.33168900	-1.82863700	2.03218600
C	1.33843200	-2.03141700	1.01713400
H	4.42924200	-1.83013200	2.48528000
H	2.01344900	-1.54352600	3.02920600
N	3.23973300	-2.89395700	-1.82757200
N	1.66333100	-2.35448600	-0.22570400
C	-0.09635900	-1.99049800	1.42506700
C	-0.40952200	-2.84205700	2.50155800
C	-1.69588800	-2.98084500	3.01667900
C	-2.71166800	-2.21014500	2.44659400
C	-2.40924300	-1.33899400	1.39278200
C	-1.12680600	-1.20216800	0.83946000
H	0.38391400	-3.44459400	2.93811000
H	-1.88271500	-3.66823100	3.83393300
H	-3.22667400	-0.73182800	1.02180600
Ir	-1.00862200	0.20413200	-0.66200900
O	-4.01941800	-2.22522000	2.84794100
C	-4.37462000	-3.06945300	3.93161100
H	-3.83530500	-2.80194300	4.85043300
H	-5.44546200	-2.92123100	4.09153100
H	-4.19093500	-4.12797400	3.70196900
C	-1.62452100	3.18936000	2.62168200
H	-0.54514600	3.36721500	2.69848300
H	-1.89670500	2.43099000	3.36392100
H	-2.14256300	4.12383700	2.87334600
C	-1.99182100	2.70137700	1.20551000
C	-3.50754500	2.44925100	1.14373300
H	-3.79883700	1.71944400	1.90691800
H	-4.07798900	3.37139200	1.31436400
H	-3.79788500	2.04788200	0.16815300
C	-1.57275800	3.77325300	0.18232500
H	-0.50692600	4.00898000	0.29095800
H	-1.73944700	3.42438400	-0.84101500
H	-2.13504800	4.70603500	0.31950900
O	-1.28604300	1.48456400	1.02114000
C	3.86617400	2.95042400	2.78460000
C	3.05292100	2.18657800	1.94445600
C	2.69821800	2.66153000	0.67586200
C	3.17270100	3.91525000	0.26651000
C	3.99315000	4.67542400	1.10144300
C	4.34161800	4.19499800	2.36595200
H	4.12785000	2.57170400	3.76962300
H	2.68314800	1.21976700	2.27001900
H	4.35090900	5.64660800	0.76874100
H	4.97346800	4.78870500	3.02147500
C	1.84368100	1.83837900	-0.27119800
H	1.26333700	2.50952500	-0.91869500
H	2.48403900	1.23052900	-0.92146500
O	0.98519600	0.91952300	0.40155700
H	0.26419500	1.41303400	0.93455400
H	2.89571500	4.30080600	-0.71294600

85

TS6 SCF Done: E(RM06) = -1836.83635657

C	-2.60906900	-0.32241200	-2.02880800
C	-1.47196700	-1.19831700	-2.25529600
C	-0.39060000	-0.36789300	-2.71643600
C	-2.25170800	1.00100600	-2.55105600
C	-0.90827000	0.98186700	-2.95446500
C	-1.44722600	-2.69266200	-2.13230100
H	-2.17981400	-3.04684100	-1.40243000
H	-1.68712500	-3.15158500	-3.10195200
H	-0.46235900	-3.04531100	-1.81595900
C	0.94764000	-0.83911100	-3.19319200
H	0.91529700	-0.98762500	-4.28302300
H	1.73289400	-0.10349700	-2.98975400
H	1.24826800	-1.77662900	-2.72672400
C	-0.13058900	2.09674500	-3.58828800
H	-0.13297600	1.99727400	-4.68328400
H	-0.54917200	3.07856900	-3.34792400
H	0.91656700	2.09161700	-3.27122100
C	-3.21924700	2.13335600	-2.71012600
H	-3.79873000	1.98350700	-3.63268400
H	-3.93709200	2.18349100	-1.88799200
H	-2.72081200	3.10277200	-2.79141300
C	-4.01243700	-0.76227500	-1.72703300
H	-4.02689900	-1.68124500	-1.13611900
H	-4.56694100	-0.00046400	-1.16943800
H	-4.56402600	-0.95758100	-2.65820500
C	4.55088400	-2.98350900	-2.14634600
C	3.00632000	-2.45435700	-0.52193700
C	4.04640100	-2.25450000	0.44053900
C	5.38829400	-2.43601600	0.03085400
C	5.64755000	-2.80358800	-1.27006900
H	4.73511900	-3.27814700	-3.17951900
C	3.65929900	-1.89212500	1.75607200
H	6.19200300	-2.28548900	0.74822700
H	6.66084100	-2.95650300	-1.62852700
C	2.32730800	-1.76676200	2.04567900
C	1.34690400	-1.99141200	1.02373000
H	4.42103700	-1.71835200	2.51277700
H	1.99577900	-1.48659000	3.03971000
N	3.28607000	-2.81984100	-1.80538900
N	1.68656400	-2.31035200	-0.21635800
C	-0.09195400	-1.97877700	1.41741200
C	-0.40491700	-2.83290100	2.49100400
C	-1.69695600	-2.99323700	2.98656100
C	-2.71833300	-2.24468500	2.39753800
C	-2.41608500	-1.37213600	1.34433500
C	-1.12708400	-1.21225400	0.81321800
H	0.39255700	-3.41966200	2.94152500
H	-1.88389800	-3.68113800	3.80335400
H	-3.23989300	-0.78374400	0.95650100
Ir	-0.99769800	0.19476700	-0.68683100
O	-4.03208300	-2.28436100	2.77743900
C	-4.38953400	-3.13789400	3.85310200
H	-3.87333200	-2.86077300	4.78229500
H	-5.46608800	-3.01269600	3.99316100
H	-4.17910700	-4.19180800	3.62532900
C	-1.58437000	3.20281400	2.71611100
H	-0.50102100	3.34962100	2.79830000
H	-1.88041300	2.43812600	3.44234400
H	-2.07631700	4.14625000	2.98449300
C	-1.96668900	2.75959200	1.29142500

C	-3.48324600	2.54017300	1.21527200
H	-3.79536300	1.79404800	1.95388700
H	-4.03082000	3.47032900	1.41194000
H	-3.77479500	2.17702600	0.22508400
C	-1.51362300	3.82851300	0.28294100
H	-0.44054000	4.02866800	0.38674300
H	-1.70264200	3.50372400	-0.74481700
H	-2.04424900	4.77560700	0.44279100
O	-1.31148200	1.50906100	1.07146900
C	3.66059100	2.87133400	2.84738000
C	2.80027900	2.16465700	2.00221500
C	2.61479400	2.57080200	0.67609500
C	3.30936700	3.69758400	0.21240300
C	4.17418800	4.39956600	1.05234400
C	4.35167700	3.98878100	2.37662200
H	3.78902900	2.54642200	3.87716600
H	2.26319900	1.29517500	2.36571500
H	4.70308000	5.27201100	0.67641700
H	5.01940000	4.53848700	3.03509600
C	1.69740300	1.81487300	-0.27264000
H	1.13797500	2.54099800	-0.88194100
H	2.30507700	1.22042700	-0.96916800
O	0.82082700	0.92928600	0.39743800
H	-0.05688300	1.45534600	0.95269800
H	3.16941600	4.02952200	-0.81513900

70

Ir-O2 SCF Done: E(RM06) = -1603.27002002

C	-1.91456300	-1.96744900	-1.29717600
C	-0.96814300	-2.80755200	-0.61803000
C	0.26983400	-2.81592700	-1.40417300
C	-1.20004900	-1.27722100	-2.35812700
C	0.12953000	-1.89219400	-2.44597400
C	-1.27669800	-3.76808400	0.49557200
H	-2.11222700	-3.41503500	1.10716800
H	-1.54744900	-4.75845400	0.10111100
H	-0.41656200	-3.90476400	1.15887400
C	1.43122200	-3.72981900	-1.16063800
H	1.25302400	-4.70335700	-1.64150600
H	2.36140100	-3.32467900	-1.56811100
H	1.58775800	-3.91326300	-0.09517100
C	1.12621800	-1.60575500	-3.52758300
H	0.89941300	-2.20874500	-4.41899500
H	1.10102000	-0.55666900	-3.83513500
H	2.14867500	-1.84278300	-3.21953900
C	-1.81940700	-0.43933800	-3.44127400
H	-2.13286200	-1.05959200	-4.29432400
H	-2.70548100	0.09316000	-3.08269600
H	-1.11886800	0.31227700	-3.81940700
C	-3.39409300	-1.93967000	-1.05045800
H	-3.63707800	-2.11716900	0.00129200
H	-3.84582300	-0.98791900	-1.33726800
H	-3.87967900	-2.72805900	-1.64318000
C	3.66250300	-1.98102100	1.22293400
C	1.71332200	-0.96728500	1.89092600
C	2.21625700	-0.80401300	3.21990100
C	3.51319200	-1.28366200	3.50436000
C	4.25016200	-1.87486900	2.50106200
H	4.22217100	-2.44221600	0.41037300
C	1.39350200	-0.15155900	4.17545500
H	3.91282700	-1.16988900	4.50905000
H	5.25380900	-2.24968800	2.67394000
C	0.17390800	0.32821500	3.79212200
C	-0.27793400	0.15467100	2.45018900
H	1.75289200	-0.02372800	5.19335800
H	-0.45361500	0.85659200	4.49941400
N	2.44601500	-1.55732700	0.92017500
N	0.46273600	-0.52059300	1.54912900
C	-1.50977800	0.71945600	1.93207100
C	-2.39835200	1.49297600	2.69845400
C	-3.49795800	2.11086300	2.11720100
C	-3.69680200	1.96945400	0.73218600
C	-2.81987000	1.18836000	-0.03514000
C	-1.73583600	0.52133200	0.53818700
H	-2.23680700	1.63257900	3.76390800
H	-4.17005300	2.70279400	2.72709700
H	-3.01256200	1.13563800	-1.10135900
Ir	-0.41796500	-0.65963300	-0.41506700
O	-4.71788400	2.55242700	0.04617500
C	-5.62020000	3.39530100	0.75118100
H	-5.10261500	4.24688600	1.21113100
H	-6.32677500	3.76659500	0.00549900
H	-6.17164100	2.84385900	1.52411100
C	1.57003100	1.25436400	-1.59521300
O	0.61732800	1.13259900	-0.59998500
H	1.12144300	1.27780100	-2.61274500
H	2.29099800	0.41203100	-1.60965800
C	2.37860700	2.54094800	-1.44160200
C	3.46206500	2.79517600	-2.29550700
C	2.05681700	3.49102400	-0.46729700
C	4.20635000	3.96940100	-2.18027000
H	3.72482500	2.06302700	-3.05837000
C	2.80075000	4.66904200	-0.34848700
H	1.21790700	3.28753500	0.18964500
C	3.87731400	4.91415700	-1.20272400
H	5.04413700	4.14800700	-2.85082700
H	2.53685600	5.39818300	0.41474700
H	4.45604300	5.83008600	-1.10968100

70

TS7 SCF Done: E(RM06) = -1603.22749750

C	-1.45744300	0.70661000	2.39596000
C	-0.12445800	0.13192000	2.55090900
C	-0.22893200	-1.27623800	2.27496900
C	-2.34763600	-0.36993200	2.02546800
C	-1.59523100	-1.59339400	1.96326600
C	1.08085100	0.84345300	3.08899700
H	1.02385000	1.91820500	2.89399700
H	1.14968600	0.70493200	4.17799200
H	1.99965600	0.47585500	2.62768800
C	0.88694300	-2.27180900	2.35670000
H	1.86663300	-1.79922000	2.25813200
H	0.85243300	-2.78700700	3.32785800
H	0.79996600	-3.03660400	1.57801700
C	-2.14722600	-2.96552700	1.71228300
H	-2.40790400	-3.46081900	2.65902500

H	-3.05295400	-2.93439100	1.09997200
H	-1.42335700	-3.60579000	1.19962000
C	-3.83092400	-0.24915400	1.82742700
H	-4.35754700	-0.45134400	2.77046900
H	-4.11340400	0.75497700	1.49902600
H	-4.19855100	-0.96184300	1.08305000
C	-1.86539300	2.08655100	2.82032900
H	-1.09327400	2.82551900	2.59274400
H	-2.78806400	2.40809000	2.32828500
H	-2.04339000	2.11112500	3.90493100
C	5.06735000	-2.11422100	1.27171400
C	3.74079300	-0.63984200	0.10000300
C	4.44964100	-0.92066700	-1.11108700
C	5.51072700	-1.85490800	-1.06928000
C	5.82657500	-2.45721300	0.12784900
H	5.30125300	-2.58149000	2.22828500
C	4.03706800	-0.23420000	-2.28228800
H	6.06022300	-2.08291200	-1.98010400
H	6.63409400	-3.17848400	0.20780300
C	3.00262000	0.65865500	-2.20091300
C	2.35195500	0.88765700	-0.94403000
H	4.54078000	-0.42996500	-3.22639000
H	2.65267200	1.18695600	-3.08138800
N	4.06956200	-1.24945300	1.27551100
N	2.71660300	0.25182300	0.15882500
C	1.29969200	1.94370600	-0.87130600
C	1.66943800	3.19293800	-1.39890600
C	0.82529300	4.30257000	-1.38950500
C	-0.45221500	4.14857600	-0.84689500
C	-0.84025900	2.90176700	-0.33505800
C	-0.00195600	1.77639300	-0.31367500
H	2.66961400	3.31403100	-1.80928400
H	1.16852900	5.24801400	-1.79401200
H	-1.85314300	2.83316800	0.04962100
Ir	-0.79465500	0.01101200	0.44394400
O	-1.39373700	5.13806200	-0.77683400
C	-1.05860700	6.41659600	-1.29414600
H	-0.83187300	6.37512200	-2.36819300
H	-1.93953000	7.04465700	-1.14037000
H	-0.20582100	6.86071900	-0.76309200
C	-4.07902700	-2.60879300	-2.92371100
C	-3.23502800	-1.55039800	-2.58975900
C	-1.96214800	-1.79745200	-2.05658500
C	-1.53748700	-3.11983300	-1.88141200
C	-2.38005800	-4.17966500	-2.22288500
C	-3.65309500	-3.92830200	-2.74013600
H	-5.06570200	-2.40612800	-3.33233700
H	-2.03945400	-5.20359200	-2.09052600
H	-4.30742500	-4.75430100	-3.00643300
C	-1.02907400	-0.65479600	-1.75955700
H	-1.82846600	0.23830200	-0.87711200
H	-1.06616600	0.13489800	-2.53045200
O	0.16252100	-0.89653200	-1.24632200
H	-0.54071500	-3.29989200	-1.49170300
H	-3.56415800	-0.52387400	-2.74233000

70

IN9 SCF Done: E(RM06) = -1603.24039221

C	-1.37496500	1.01855100	2.28527400
C	-0.18263400	0.21779500	2.54185400
C	-0.49964300	-1.13331800	2.24657900
C	-2.44921100	0.09257500	1.92621200
C	-1.89645600	-1.21533900	1.86651600
C	1.11532000	0.73139900	3.09044600
H	1.23764500	1.79881100	2.88615100
H	1.14684200	0.59746700	4.18149000
H	1.96778000	0.21024500	2.64862400
C	0.40405500	-2.31797300	2.38958400
H	1.45595000	-2.03005700	2.43473200
H	0.15700000	-2.86772800	3.31001700
H	0.28304800	-3.01752100	1.55554400
C	-2.64480500	-2.49152000	1.62327600
H	-2.88956700	-2.97919400	2.57813000
H	-3.58362000	-2.31945900	1.09082300
H	-2.05650600	-3.20319800	1.03731900
C	-3.89873100	0.45167700	1.76599900
H	-4.42788500	0.32743900	2.72130400
H	-4.02426200	1.49121200	1.45180100
H	-4.39446600	-0.18329400	1.02626900
C	-1.56820200	2.44315000	2.72290100
H	-0.66941800	3.04296700	2.55867500
H	-2.38921800	2.92360100	2.18252500
H	-1.80707300	2.48465300	3.79493400
C	4.47126700	-3.12492700	1.46837300
C	3.48668400	-1.46179300	0.21571700
C	4.10098800	-1.95729500	-0.97775600
C	4.92906000	-3.10027300	-0.88391700
C	5.11929800	-3.69102500	0.34509300
H	4.61063800	-3.57710400	2.45031200
C	3.84147400	-1.25795500	-2.18510000
H	5.40351900	-3.49348000	-1.78039300
H	5.74812700	-4.56782800	0.46521200
C	3.04039900	-0.14857900	-2.15257600
C	2.47325500	0.28614600	-0.91022500
H	4.27899700	-1.61160700	-3.11609200
H	2.81503500	0.40504700	-3.05786700
N	3.68844300	-2.06239700	1.42323300
N	2.68644500	-0.36110200	0.22430400
C	1.73025900	1.58144000	-0.87969900
C	2.46301900	2.67600800	-1.36998100
C	1.98003800	3.98316400	-1.36477400
C	0.69807200	4.20066900	-0.85754900
C	-0.05579000	3.11438200	-0.39150600
C	0.41136000	1.78943700	-0.37910100
H	3.46968600	2.50368500	-1.74440600
H	2.59661300	4.79110100	-1.74173900
H	-1.05648800	3.34476200	-0.04094400
Ir	-0.88222400	0.31291600	0.29784600
O	0.08499500	5.42075600	-0.78516100
C	0.79177700	6.55207200	-1.27013700
H	1.02599400	6.45834300	-2.33924300
H	0.12781500	7.40764000	-1.12476400
H	1.72198500	6.72230900	-0.71132700
C	-4.52931100	-1.94280900	-2.72179600
C	-3.51034300	-1.03559400	-2.43674100
C	-2.26943700	-1.48380200	-1.95623100

C	-2.06038000	-2.86125800	-1.79281600
C	-3.07755000	-3.76970200	-2.08918700
C	-4.31636400	-3.31462300	-2.54800900
H	-5.48761100	-1.58254800	-3.08746600
H	-2.90096400	-4.83569900	-1.96799900
H	-5.10781100	-4.02294400	-2.77873400
C	-1.16844000	-0.50981800	-1.71608600
H	-1.89960900	1.31152900	-0.38470600
H	-1.16401500	0.34443100	-2.40589700
O	-0.01552000	-0.89005800	-1.23406400
H	-1.08884900	-3.20504400	-1.45140100
H	-3.67248800	0.03065200	-2.58182600

14

2a-1 SCF Done: E(RM06) = -345.423119235

C	1.73382700	1.06318400	-0.00000200
C	0.35821100	1.29187600	0.00000000
C	-0.53501500	0.21114300	0.00000200
C	-0.04223300	-1.10401800	0.00000300
C	1.33015300	-1.33072000	0.00000100
C	2.21814800	-0.24748400	-0.00000200
H	2.42612100	1.90049500	-0.00000400
H	-0.02996900	2.30830800	-0.00000100
H	-0.74978800	-1.92775700	0.00000600
H	1.71417200	-2.34712800	0.00000100
H	3.28995300	-0.42767400	-0.00000400
C	-1.99084900	0.46758200	0.00000500
H	-2.27077200	1.54384100	0.00001500
O	-2.85164600	-0.39493200	-0.00000700

87

TS8 SCF Done: E(RM06) = -2079.15228870

C	0.18037600	-0.71195000	-2.86115800
C	0.53254900	-2.09412700	-2.57964400
C	1.88976600	-2.11073100	-2.13813100
C	1.35647000	0.10123800	-2.58083800
C	2.39293100	-0.75996500	-2.12351800
C	-0.32205600	-3.28934100	-2.88702200
H	-1.38061100	-3.08563000	-2.70223500
H	-0.21810300	-3.57671700	-3.94261900
H	-0.04053400	-4.15600100	-2.28111600
C	2.66946100	-3.33458300	-1.77017600
H	3.22796100	-3.70877700	-2.64011800
H	3.39232200	-3.12535900	-0.97830300
H	2.01479700	-4.14296600	-1.43067300
C	3.81203700	-0.35379400	-1.86265100
H	4.35662900	-0.25523900	-2.81298700
H	3.86836000	0.60725600	-1.34612600
H	4.33658200	-1.09430200	-1.25599900
C	1.47195400	1.56101600	-2.89000800
H	1.61578700	1.70053700	-3.97151900
H	0.56522000	2.09868800	-2.59992500
H	2.32070200	2.01753300	-2.37691600
C	-0.97252300	-0.25191200	-3.70763700
H	-1.79613500	-0.96986300	-3.70143900
H	-1.34885600	0.71851500	-3.37472900
H	-0.64035100	-0.14921000	-4.75018600
C	4.24804100	-1.42938800	1.70801000
C	1.98672400	-1.80936400	1.83594900
C	2.12200500	-2.34245000	3.15535700
C	3.41037200	-2.38117000	3.73114200
C	4.48781300	-1.91684800	3.00864500
H	5.08015300	-1.06437200	1.10790500
C	0.95763000	-2.81102400	3.81913500
H	3.52945300	-2.77709700	4.73644100
H	5.49448100	-1.92414600	3.41324000
C	-0.24763600	-2.74607200	3.18399000
C	-0.33864200	-2.19647700	1.87037500
H	1.04089600	-3.21338500	4.82531400
H	-1.14696400	-3.09127800	3.67842400
N	3.05518800	-1.37622100	1.13405700
N	0.75467000	-1.73792800	1.22807700
C	-1.58148600	-2.07558700	1.14119600
C	-2.80798600	-2.58868100	1.60067100
C	-3.97279000	-2.44758000	0.86192600
C	-3.91177300	-1.76781400	-0.36708000
C	-2.69239700	-1.25165700	-0.83191100
C	-1.50874700	-1.39308800	-0.10674700
H	-2.86404900	-3.11595300	2.54821700
H	-4.90435400	-2.85141700	1.23941800
H	-2.71730100	-0.71178400	-1.76974000
Ir	0.33213900	-0.76065000	-0.68341300
H	-0.75209800	0.47089000	-0.96170600
O	-4.98007300	-1.55769100	-1.17728800
C	-6.26488700	-1.99570700	-0.74560500
H	-6.55714600	-1.51506800	0.19604700
H	-6.96011300	-1.69645100	-1.53262600
H	-6.30182300	-3.08642400	-0.62906200
C	2.92640100	2.22695800	0.76089100
C	1.67709000	2.82729400	0.41997700
C	1.68237200	4.23458900	0.11050000
C	2.90050800	4.94016500	0.09781000
C	4.10660500	4.32965800	0.40193200
C	4.09866700	2.96170700	0.74238300
H	2.92703100	1.17766700	1.02383700
H	2.85325500	5.99821900	-0.14527900
H	5.03485200	4.89402500	0.39655100
H	5.03451800	2.46902000	1.00233900
C	0.45003100	4.98472400	-0.13791900
H	-0.49222800	4.43890600	0.05964400
O	0.40278800	6.15513500	-0.51091600
N	0.50746700	2.13504800	0.40830700
O	0.57333100	0.83587600	0.80630800
C	-3.17349500	1.88662600	2.82609800
C	-2.61796500	1.80082200	1.54527300
C	-3.24043300	2.43239700	0.46269600
C	-4.42378300	3.15150100	0.69155400
C	-4.98143900	3.23080600	1.96714600
C	-4.35492200	2.59618100	3.04375000
H	-2.67196200	1.39748700	3.65806600
H	-1.69382800	1.25329600	1.39441100
H	-4.91129800	3.65784300	-0.14024500
H	-5.89758000	3.79574500	2.12301300
H	-4.78085400	2.66249700	4.04197700
C	-2.71102000	2.34685000	-0.96242700
H	-3.35835000	1.66188700	-1.53143700

H -2.83073300 3.33642400 -1.43431900
O -1.37610000 1.91802100 -1.13457300
H -0.59824000 2.24867000 -0.37769600

87
IN10 SCF Done: E(RM06) = -2079.22221171

C	1.55973900	0.08200900	-2.73516300
C	2.04341100	-1.27863400	-2.58720800
C	3.23698600	-1.22118600	-1.79234200
C	2.59234800	0.96788100	-2.19199600
C	3.61172000	0.18564000	-1.63401000
C	1.48873000	-2.50431900	-3.25078200
H	0.41206700	-2.43179200	-3.41940100
H	1.96880100	-2.64581600	-4.22945200
H	1.67008300	-3.40484800	-2.65685300
C	4.12225500	-2.38239700	-1.43858400
H	4.91543700	-2.51454100	-2.18866200
H	4.61066900	-2.23474100	-0.47002000
H	3.55717300	-3.31761300	-1.38590800
C	4.90725500	0.66738800	-1.05688800
H	5.68448200	0.68845400	-1.83548800
H	4.82009700	1.67817200	-0.64937300
H	5.26116600	0.01427200	-0.25627100
C	2.53026100	2.46208400	-2.26070000
H	2.78787300	2.80966800	-3.27168200
H	1.52375000	2.82666800	-2.03840800
H	3.22233700	2.93475400	-1.55862600
C	0.44887500	0.54252400	-3.63461700
H	-0.28977300	-0.24632400	-3.80486000
H	-0.07659800	1.40152500	-3.20679200
H	0.83709700	0.84338600	-4.61874200
C	4.43954400	0.89942500	2.59468200
C	2.71348100	-0.58097400	2.26793500
C	2.80390300	-1.03251600	3.62218700
C	3.78930700	-0.45538000	4.45227800
C	4.61657700	0.52166500	3.94203800
H	5.07651700	1.67014500	2.16297600
C	1.88890200	-2.02286300	4.06636700
H	3.87363000	-0.78703000	5.48420100
H	5.38296600	0.99532200	4.54696500
C	0.93814300	-2.48834700	3.20447600
C	0.87925100	-2.00172700	1.86519600
H	1.94422000	-2.38334300	5.09032400
H	0.21201700	-3.22159900	3.53336200
N	3.53550800	0.37519300	1.78251100
N	1.77666700	-1.10190700	1.40893700
C	-0.14755800	-2.38397600	0.91616200
C	-1.17992400	-3.29314100	1.20502600
C	-2.19814300	-3.53590700	0.29249600
C	-2.19420300	-2.83311500	-0.92605800
C	-1.15865500	-1.93448900	-1.22521700
C	-0.10104300	-1.71041300	-0.34060000
H	-1.20576300	-3.82111600	2.15443300
H	-2.98468300	-4.24036000	0.53521400
H	-1.21919000	-1.40777800	-2.17125200
Ir	1.46651200	-0.48696300	-0.62547000
H	-4.02983200	1.85637200	-0.64643100
O	-3.15734300	-2.96168700	-1.87927000
C	-4.23072900	-3.86608800	-1.64542100
H	-4.81165600	-3.58595700	-0.75802100
H	-4.87095100	-3.80274200	-2.52816800
H	-3.87222800	-4.89791600	-1.53620500
C	0.66409100	3.55629300	0.90515100
C	-0.36848600	3.10111800	0.04962100
C	-1.30631600	4.05896100	-0.45087400
C	-1.22829200	5.39748800	-0.01899500
C	-0.22746300	5.82518500	0.83812000
C	0.72502800	4.88909700	1.28198800
H	1.38214200	2.83073000	1.26608000
H	-1.96884800	6.09033000	-0.40902500
H	-0.16965200	6.86369900	1.15099400
H	1.52464900	5.21148700	1.94593300
C	-2.30421200	3.70251900	-1.45998000
H	-2.10259000	2.76438900	-2.01582300
O	-3.29372000	4.37045500	-1.74994800
N	-0.45669700	1.77281900	-0.30227900
O	0.30691200	0.89061300	0.45628400
C	-7.12725500	-0.96574400	0.13772600
C	-5.82085400	-0.48436900	0.03799600
C	-5.26264200	0.27627400	1.07514800
C	-6.03737000	0.54623500	2.20955400
C	-7.34149200	0.05731200	2.31479500
C	-7.89071400	-0.69934600	1.27779400
H	-7.55161600	-1.54769100	-0.67681200
H	-5.22577300	-0.68693500	-0.84810100
H	-5.61822100	1.14680900	3.01425700
H	-7.93118600	0.27781400	3.20103300
H	-8.90856300	-1.07306900	1.35378600
C	-3.83503600	0.77555000	0.98190100
H	-3.68107600	1.61718000	1.67219900
H	-3.13187500	-0.01347700	1.27001400
O	-3.45067600	1.13363600	-0.34934400
H	-1.40476200	1.41449400	-0.42137400

71
TS8' SCF Done: E(RM06) = -1732.49020995

C	-0.36403200	-2.83276800	0.36507000
C	-1.51296100	-2.75012300	-0.52149100
C	-2.61513800	-2.25711800	0.23191400
C	-0.79311900	-2.35392100	1.67051700
C	-2.16222300	-1.95356300	1.55561100
C	-1.55342700	-3.23757600	-1.94093900
H	-0.55722600	-3.23875200	-2.39212500
H	-1.94090700	-4.26485400	-1.99770100
H	-2.19782500	-2.61137700	-2.56765300
C	-4.00688100	-2.06479100	-0.28787100
H	-4.62717600	-2.94675900	-0.07209000
H	-4.49557800	-1.20136600	0.17131900
H	-4.01857400	-1.92316100	-1.37346900
C	-2.96293600	-1.39225000	2.69099400
H	-2.98358100	-2.08949700	3.53922600
H	-2.52051100	-0.45332000	3.04392400
H	-3.99790800	-1.19378500	2.40057700
C	-0.03524300	-2.46139800	2.95982200
H	-0.39695800	-3.32564100	3.53542200
H	1.03559600	-2.59526100	2.79190300

H -0.16036200 -1.56518900 3.57213400
C 0.84184500 -3.69543400 0.12118500
H 1.15968400 -3.67481100 -0.92357800
H 1.69230700 -3.39780500 0.74058100
H 0.59881900 -4.73775400 0.37127700
C -4.14262600 1.90555400 1.08664300
C -2.48737900 1.65731400 -0.47812300
C -2.99257200 2.75207100 -1.24353400
C -4.13236100 3.42882500 -0.75711300
C -4.71012900 3.01374700 0.42338300
H -4.58793600 1.54540800 2.01249400
C -2.32818900 3.09683500 -2.45102400
H -4.53373200 4.26707900 -1.32063700
H -5.58149300 3.51013100 0.83764500
C -1.22225100 2.39568900 -2.83706000
C -0.72631600 1.33380700 -2.02551200
H -2.70497500 3.92076100 -3.05140500
H -0.69869800 2.65266500 -3.74993100
N -3.08092100 1.24022500 0.65803400
N -1.36147400 0.97899000 -0.88678300
C 0.48013000 0.59264000 -2.30351500
C 1.30507000 0.82344400 -3.41871800
C 2.49703900 0.13528800 -3.58554800
C 2.88359600 -0.79461900 -2.60239700
C 2.06158100 -1.03827600 -1.49099700
C 0.84480600 -0.37719500 -1.32227200
H 1.02418600 1.55815900 -4.16749400
H 3.11928100 0.33184300 -4.45021000
H 2.42429000 -1.74976900 -0.75922800
Ir -0.46735700 -0.62972300 0.19621100
H 0.97690700 -0.68176200 1.01162600
O 4.03433600 -1.50996900 -2.64276800
C 4.96151100 -1.26478200 -3.69596200
H 5.29901200 -0.22110500 -3.69842300
H 5.81404600 -1.91740400 -3.49764300
H 4.53580100 -1.51675000 -4.67552000
C 1.62735400 2.72559600 1.20315800
C 1.97495900 1.51821100 1.88296700
C 3.34192600 1.38725900 2.32543000
C 4.26977200 2.40904800 2.05147600
C 3.91003300 3.56353400 1.37506300
C 2.57016700 3.70759000 0.96037100
H 0.59798400 2.85712600 0.89625900
H 5.28682400 2.25845900 2.40397900
H 4.63785200 4.34578200 1.17800600
H 2.26524900 4.61382200 0.43891300
C 3.79122600 0.22321000 3.08601200
H 2.99676100 -0.50127300 3.35006300
O 4.94898900 0.01629800 3.44831500
N 1.09659300 0.51402300 2.15942300
O -0.20000500 0.75956000 1.64615100

71

IN10' SCF Done: E(RM06) = -1732.58402243

C 1.38757400 -2.88021300 0.15082000
C 2.55816000 -2.10278800 0.45296200
C 3.06751100 -1.54902900 -0.80247900
C 1.06058600 -2.65393000 -1.24330100
C 2.15187600 -1.86677000 -1.82030500
C 3.30719400 -2.10534300 1.75473700
H 2.64092200 -2.29748900 2.60082100
H 4.08341600 -2.88448900 1.75880500
H 3.80537300 -1.14738500 1.93485100
C 4.39149800 -0.86576700 -0.95840500
H 5.18813200 -1.61603000 -1.07249600
H 4.41511100 -0.21775300 -1.83777800
H 4.63729200 -0.25313600 -0.08870200
C 2.17396400 -1.41509800 -3.24710600
H 2.09379600 -2.26710600 -3.93398500
H 1.32240300 -0.74677800 -3.43113900
H 3.09064400 -0.87005400 -3.48785500
C 0.01536400 -3.36583000 -2.05535900
H 0.44377900 -4.23436800 -2.57621600
H -0.80223800 -3.73757800 -1.43045100
H -0.41654800 -2.70605200 -2.81507900
C 0.71855300 -3.84122400 1.08765100
H 0.69733900 -3.46143300 2.11303500
H -0.31025100 -4.05821300 0.79274400
H 1.26802600 -4.79304400 1.09318700
C 3.87097600 2.68104400 -0.36024400
C 2.01688300 1.98515600 0.80333000
C 2.05515600 3.15541600 1.62513700
C 3.08503000 4.09437000 1.39819200
C 4.00208900 3.86454400 0.39563000
H 4.57975400 2.47111600 -1.16001100
C 1.04992600 3.32282800 2.61273500
H 3.13084200 4.98935000 2.01379200
H 4.80401500 4.56396500 0.18266700
C 0.06278000 2.38636700 2.72383400
C 0.05353600 1.24476700 1.87168600
H 1.06784800 4.20034200 3.25383700
H -0.72763600 2.50590200 3.45468800
N 2.92844500 1.77180500 -0.16922500
N 1.03470800 1.03526500 0.96494200
C -1.00062000 0.25065000 1.87067500
C -2.15558700 0.32695300 2.66777200
C -3.18184200 -0.59954000 2.53794900
C -3.05158600 -1.62332500 1.58278100
C -1.89155300 -1.71361000 0.79580000
C -0.84164900 -0.79825900 0.91925200
H -2.27874600 1.12915800 3.38989300
H -4.06863300 -0.51330900 3.15422100
H -1.85255300 -2.52170800 0.07219100
Ir 0.85859400 -0.77077700 -0.16460300
H -1.83058000 -0.47040500 -1.49874900
O -3.99418500 -2.57185400 1.34743900
C -5.25442200 -2.46151200 2.00312500
H -5.74991900 -1.51516900 1.75528100
H -5.85719300 -3.29152900 1.62871500
H -5.15357900 -2.55393100 3.09200400
C -1.43892900 2.79249200 -1.76882900
C -2.11964200 1.54836100 -1.70151200
C -3.54916500 1.56717700 -1.57641800
C -4.21753200 2.80833300 -1.51739400
C -3.53904400 4.01176800 -1.58086100
C -2.13648900 3.98541200 -1.70771100

H -0.36273400 2.77626800 -1.88122500
H -5.29904000 2.77783500 -1.42087400
H -4.07390400 4.95615700 -1.53943000
H -1.58319900 4.92074400 -1.76337500
C -4.36062200 0.35242600 -1.50457900
H -3.82403300 -0.61012800 -1.63620500
O -5.57517000 0.32408800 -1.32726100
N -1.41045100 0.38729700 -1.83286600
O -0.03045200 0.40408400 -1.67518800

71
TS8" SCF Done: E(RM06) = -1732.48776846
C 0.16026100 -2.89291400 0.30272900
C -0.38328300 -3.01992400 -1.05874900
C -1.74164100 -2.70931600 -1.00978200
C -0.94043300 -2.65069400 1.19733900
C -2.09249000 -2.40641200 0.38175400
C 0.43626500 -3.38420800 -2.25834000
H 0.78419500 -4.42480500 -2.19828900
H -0.13280800 -3.27941200 -3.18669600
H 1.32820100 -2.75132300 -2.34051700
C -2.71948600 -2.66467300 -2.14572900
H -3.45389400 -3.47868400 -2.06570500
H -3.28740900 -1.72687600 -2.15546900
H -2.22346000 -2.76356900 -3.11565900
C -3.50069800 -2.29444900 0.88819200
H -3.53676300 -1.82149000 1.87270400
H -4.12971500 -1.71164800 0.21169800
H -3.94558000 -3.29691200 0.97843800
C -0.92308200 -2.76477900 2.69307800
H -1.35322200 -1.87263700 3.15482800
H -1.48430100 -3.65413400 3.01231800
H 0.09936700 -2.86074700 3.06657800
C 1.49294600 -3.44487600 0.72959600
H 1.91924300 -2.89017200 1.56978300
H 1.38203500 -4.49101600 1.04956300
H 2.21968700 -3.43787600 -0.08696500
C -4.57071000 1.30453000 0.46279100
C -2.52931800 1.54749700 -0.56842400
C -2.96982000 2.73804000 -1.22517300
C -4.28990200 3.17977600 -0.98747300
C -5.10264800 2.46521800 -0.13453300
H -5.18259500 0.71472500 1.14318200
C -2.06231400 3.42430900 -2.07320400
H -4.64032400 4.08371600 -1.47909400
H -6.12038500 2.77460000 0.07962400
C -0.79652300 2.93830700 -2.22129700
C -0.38927200 1.75645000 -1.53779300
H -2.38298900 4.32765600 -2.58524200
H -0.08398000 3.44939400 -2.85644100
N -3.34291800 0.85695500 0.25385200
N -1.24182700 1.07195000 -0.73928000
C 0.94112000 1.19981500 -1.63038200
C 1.97400900 1.75865400 -2.40240200
C 3.23266700 1.17466700 -2.44437500
C 3.46560400 0.01287700 -1.68952300
C 2.43756300 -0.53859400 -0.90382400
C 1.16395300 0.02495500 -0.86051700
H 1.80965400 2.66072300 -2.98445300
H 4.01437400 1.62113800 -3.04740100
H 2.69026300 -1.41454500 -0.32115300
Ir -0.47796800 -0.71789500 0.13436700
H -1.51860400 -0.14640100 1.26208200
O 4.64706200 -0.65134500 -1.65785500
C 5.75456500 -0.11610100 -2.37413800
H 6.01405900 0.88916300 -2.01967300
H 6.58952800 -0.79186700 -2.17841800
H 5.56019300 -0.08620100 -3.45408100
C 0.41737300 2.56977200 2.06181600
C 1.04305300 1.30945900 2.02449100
C 2.45978600 1.25659300 2.03032800
C 3.20321700 2.45000000 1.98545100
C 2.57177400 3.68489500 1.96415100
C 1.16846300 3.73661300 2.01442500
H -0.66245200 2.59338700 2.13686800
H 4.28639600 2.37034500 1.99666900
H 3.15454400 4.60182900 1.93539700
H 0.66352100 4.70038700 2.02251200
C 3.18468400 -0.01594300 2.20524600
H 2.54340200 -0.88603800 2.42988500
O 4.40309600 -0.13041400 2.16296300
N 0.29487100 0.08629100 2.12052500
O -0.88286300 0.33095300 2.72824100

71
IN10" SCF Done: E(RM06) = -1732.58822788
C 0.24210200 -2.88626700 0.72325600
C 0.14286500 -2.88927800 -0.72440700
C -1.20943500 -2.57293200 -1.06826700
C -1.11516300 -2.74847300 1.24672900
C -1.99739400 -2.55127200 0.16871300
C 1.22256500 -3.27915200 -1.68876900
H 1.15658100 -4.35750000 -1.89209700
H 1.13085400 -2.75436800 -2.64403600
H 2.22111200 -3.07987100 -1.29471700
C -1.78325400 -2.55199600 -2.45588100
H -2.12451200 -3.55485100 -2.75130500
H -2.64577700 -1.88236800 -2.52906800
H -1.04326200 -2.22457100 -3.19201500
C -3.49293600 -2.46210500 0.22882200
H -3.84031500 -2.09556400 1.19823700
H -3.89314400 -1.79731000 -0.54077100
H -3.93972200 -3.45442900 0.06930000
C -1.45034100 -2.81450400 2.70453600
H -2.48513200 -2.52571500 2.90055200
H -1.30575500 -3.83888800 3.07590700
H -0.80533800 -2.14789000 3.28244300
C 1.41902000 -3.34217100 1.53686500
H 1.43185900 -2.86419600 2.52084600
H 1.38538700 -4.42993800 1.69740800
H 2.36934400 -3.11573400 1.04503500
C -4.46034300 1.18839300 0.34924400
C -2.44256000 1.34556100 -0.76057400
C -2.95805900 2.37654700 -1.60538100
C -4.30756000 2.75581700 -1.44632800
C -5.07466300 2.15375700 -0.46997500

H	-5.01958700	0.72427600	1.15912300
C	-2.07389200	2.98671400	-2.53464700
H	-4.71873700	3.53022900	-2.08890500
H	-6.11374700	2.42279500	-0.31234600
C	-0.76883000	2.59131700	-2.56810000
C	-0.30696100	1.53693200	-1.72353400
H	-2.44124700	3.77362200	-3.18786900
H	-0.07212900	3.06606300	-3.24771400
N	-3.20211000	0.79111000	0.21194000
N	-1.14957300	0.89602700	-0.87901200
C	1.06088300	1.07429100	-1.69560200
C	2.09965600	1.66307500	-2.44061300
C	3.40525400	1.20899000	-2.33385300
C	3.68000500	0.14534600	-1.45396800
C	2.64857100	-0.44300300	-0.70621900
C	1.32382600	-0.01325200	-0.80864600
H	1.90060700	2.49482700	-3.11019200
H	4.19100000	1.67839100	-2.91357800
H	2.93160000	-1.24149000	-0.03023500
Ir	-0.28855400	-0.85605500	0.07423800
H	-2.09606800	0.38411900	1.69126100
O	4.91420400	-0.38776100	-1.27147800
C	6.02374600	0.20582700	-1.93551200
H	6.15063600	1.25643900	-1.64605500
H	6.90007800	-0.36178300	-1.61547800
H	5.92803300	0.13468000	-3.02685300
C	-0.32932400	2.71683400	2.14692000
C	0.43276100	1.52528400	2.02139400
C	1.85381500	1.65250900	2.11879200
C	2.44789400	2.92630700	2.19056000
C	1.68410600	4.08186500	2.23053700
C	0.28393000	3.95782300	2.23419400
H	-1.40917700	2.64200700	2.16933500
H	3.53176700	2.96460100	2.25768200
H	2.15378200	5.05963700	2.29466600
H	-0.33649500	4.84961000	2.30374800
C	2.71174700	0.47938900	2.32806000
H	2.16557400	-0.47074200	2.47172400
O	3.93518400	0.51049100	2.41933700
N	-0.14413700	0.26704000	1.88217100
O	-1.47949300	0.29525200	2.45387600

86

TS8-1 SCF Done: E(RM06) = -1966.10951799

C	0.80064400	-0.56136300	-2.57507800
C	1.15213700	0.83850800	-2.74972400
C	-0.06525000	1.59690300	-2.76198300
C	-0.63843100	-0.62467500	-2.43208600
C	-1.16264300	0.70839900	-2.52957600
C	2.51930700	1.36227000	-3.08053600
H	3.30267700	0.76457800	-2.60701400
H	2.69173400	1.33816100	-4.16563500
H	2.64764900	2.39782300	-2.74977000
C	-0.15329000	3.07457700	-2.98488400
H	-0.22468500	3.29354100	-4.05985900
H	-1.03057700	3.50430000	-2.49768000
H	0.73452100	3.59274800	-2.60874500
C	-2.62169900	1.05335600	-2.55741100
H	-3.08767700	0.65706000	-3.47068900
H	-3.15485600	0.62934200	-1.70265400
H	-2.77685800	2.13400500	-2.54919800
C	-1.44720500	-1.88310500	-2.42363000
H	-1.62486200	-2.20824000	-3.45944500
H	-0.92777300	-2.68880300	-1.90104200
H	-2.41869000	-1.73783700	-1.94838000
C	1.66494000	-1.74716300	-2.90028300
H	2.72718500	-1.49193000	-2.90430000
H	1.50502100	-2.57043900	-2.19872000
H	1.41634800	-2.11188400	-3.90658200
C	-2.80070000	3.58637500	0.17962900
C	-0.74140600	2.88103900	0.90447000
C	-0.74197900	3.85362100	1.95167800
C	-1.87042000	4.69269800	2.08115200
C	-2.91666900	4.55816300	1.19539600
H	-3.60483100	3.46613300	-0.54447500
C	0.39117500	3.93547000	2.80242500
H	-1.89289300	5.43157800	2.87815800
H	-3.80473100	5.17819100	1.25988000
C	1.44779800	3.09792300	2.59351000
C	1.39952300	2.13131700	1.54616900
H	0.40442200	4.66485400	3.60807900
H	2.32019000	3.14419900	3.23329300
N	-1.76205300	2.77922900	0.02882300
N	0.32236100	2.02218700	0.74442200
C	2.47189000	1.20583900	1.25693500
C	3.73103800	1.25745600	1.88051600
C	4.74468600	0.37470700	1.53726200
C	4.48509200	-0.59119400	0.55013800
C	3.22594900	-0.65740000	-0.06627700
C	2.19641200	0.22863400	0.25575600
H	3.93797500	2.00545300	2.64007200
H	5.70794300	0.43901200	2.02847900
H	3.08349400	-1.44631200	-0.79207300
Ir	0.36188900	0.33471500	-0.62239900
H	0.64029700	-1.28607000	-0.06588300
O	5.38815700	-1.51452000	0.12804800
C	6.68501400	-1.52415000	0.71683600
H	6.63635400	-1.71593500	1.79602900
H	7.22526300	-2.33967800	0.23173300
H	7.21670100	-0.58156100	0.53494200
C	-3.75532400	-0.07415200	1.03323300
C	-3.05855900	-1.30350400	0.78399800
C	-3.87662700	-2.48492900	0.60262100
C	-5.27727200	-2.39586900	0.74600200
C	-5.91880400	-1.20067900	1.01451200
C	-5.13015800	-0.03505400	1.14270300
H	-3.16183100	0.82048900	1.15782100
H	-5.83894400	-3.31712600	0.61493800
H	-6.99931800	-1.15437800	1.11766800
H	-5.61486400	0.91933000	1.34506000
C	-3.31232700	-3.77460500	0.22546400
H	-2.22378100	-3.77962800	0.03094800
O	-3.94890500	-4.82153700	0.09256300
N	-1.71094100	-1.38570600	0.77847500
O	-1.04699100	-0.18505700	0.91716300
C	1.19450400	-2.41479500	2.61687400

H	0.26780100	-1.90838400	2.90506100
H	1.92866600	-1.65332000	2.33960200
H	1.57745800	-2.95723600	3.49042800
C	0.93925800	-3.38328500	1.45369100
C	2.23951300	-4.03296700	0.97247000
H	2.97629900	-3.28095200	0.67564800
H	2.68335600	-4.64196100	1.76941700
H	2.04511600	-4.68589400	0.11377100
C	-0.05792600	-4.47092000	1.88436600
H	-0.99551800	-4.02814600	2.23711900
H	-0.28609000	-5.14383800	1.05045300
H	0.35863300	-5.07102700	2.70263000
O	0.35915800	-2.68019300	0.32007200
H	-0.64188000	-2.31750400	0.57277000

87

IN11 SCF Done: E(RM06) = -2079.18976561

C	-0.59292800	-2.48153700	-2.06560900
C	0.85917500	-2.40624300	-1.98943200
C	1.25539800	-1.22100100	-2.70348700
C	-1.04914600	-1.34481600	-2.85413500
C	0.08804400	-0.58057400	-3.26726900
C	1.77160300	-3.45882000	-1.43437800
H	1.28236600	-4.02049200	-0.63342800
H	2.05091300	-4.17350900	-2.22227400
H	2.68191400	-3.02132600	-1.01975600
C	2.67014400	-0.78844800	-2.93063400
H	3.04478100	-1.25475800	-3.85354300
H	2.75145300	0.29487300	-3.06270400
H	3.32804900	-1.09133100	-2.11252400
C	0.07064100	0.62294900	-4.16446500
H	0.04279600	0.32678500	-5.22280900
H	-0.80545500	1.25261700	-3.97909000
H	0.96195100	1.24191200	-4.02695200
C	-2.47875200	-1.06290700	-3.19907000
H	-2.77104400	-1.63669200	-4.08945900
H	-3.14828100	-1.34598800	-2.38237300
H	-2.64253200	-0.00381400	-3.41190700
C	-1.43189200	-3.65574600	-1.65956400
H	-1.04799700	-4.13421100	-0.75528000
H	-2.47172900	-3.37109800	-1.47427700
H	-1.43309300	-4.40692000	-2.46217600
C	6.16243200	-0.87302900	-0.33521700
C	4.16690200	-0.75126900	0.81098400
C	4.74477600	0.12988800	1.77945400
C	6.10495900	0.49106000	1.63496900
C	6.82373700	-0.01435100	0.57484200
H	6.71249300	-1.27870400	-1.18409100
C	3.90218100	0.59092900	2.82398000
H	6.56228200	1.15880700	2.36178500
H	7.87061600	0.23169600	0.42650000
C	2.60322600	0.16135700	2.87178800
C	2.11109100	-0.74703500	1.87655100
H	4.29390500	1.28265800	3.56632100
H	1.93174800	0.51414300	3.64689600
N	4.89532100	-1.23212900	-0.23796700
N	2.87462500	-1.16717800	0.87770500
C	0.73428900	-1.29801900	2.02617500
C	0.42100200	-1.76710500	3.31490200
C	-0.79321700	-2.37194400	3.63399900
C	-1.74005300	-2.50882000	2.61707800
C	-1.44422900	-2.03581600	1.32918300
C	-0.23288900	-1.41638900	0.98115300
H	1.16997500	-1.68715100	4.09921100
H	-0.97434800	-2.72750800	4.64184800
H	-2.22526900	-2.15270400	0.58511300
Ir	-0.10162000	-0.71339900	-0.97098900
H	-2.64166900	0.10324700	1.04789300
O	-2.97518800	-3.07077700	2.77084200
C	-3.34420200	-3.53805200	4.06175300
H	-3.34462400	-2.72669600	4.80137400
H	-4.35916000	-3.92865700	3.96078700
H	-2.68087300	-4.34270300	4.40578600
C	-3.68817700	2.36202800	-1.07592100
C	-3.79479000	1.37014200	-0.07358300
C	-5.00064300	1.30952300	0.69299600
C	-6.02585200	2.24020800	0.43743500
C	-5.90648500	3.21073100	-0.54429300
C	-4.72421500	3.25742300	-1.30039600
H	-2.77515800	2.40867400	-1.65472600
H	-6.92328500	2.16260600	1.04410000
H	-6.70975900	3.91821100	-0.72731700
H	-4.60879000	4.01171000	-2.07583000
C	-5.23604500	0.29797200	1.73161500
H	-4.44784800	-0.47041700	1.86735000
O	-6.23980600	0.24123600	2.43207400
N	-2.77529300	0.45579300	0.10614500
O	-1.54522500	0.79961300	-0.45757600
C	1.37786000	2.20682500	-0.83149700
O	0.80459700	1.17766600	0.00875100
H	-0.15124800	1.42051200	0.18816400
H	2.33890000	1.81593300	-1.17029200
H	0.73320900	2.35629500	-1.70593300
C	1.55884000	3.50145900	-0.07459000
C	2.82393300	3.90594500	0.36887600
C	0.45097600	4.31608500	0.20318500
C	2.98190700	5.10071300	1.07453300
H	3.68984600	3.28286600	0.15810100
C	0.60457400	5.50585200	0.91489300
H	-0.53749700	4.01663500	-0.13940200
C	1.87216400	5.90173100	1.35069700
H	3.97058700	5.40537800	1.40816200
H	-0.26270800	6.12653300	1.12383400
H	1.99371400	6.83157800	1.90013000

87

TS9 SCF Done: E(RM06) = -2079.18347504

C	0.08890000	-3.36008800	-1.13985500
C	1.45623800	-2.87952800	-1.03730900
C	1.71387800	-2.04079800	-2.20911300
C	-0.50143200	-2.70955400	-2.28045700
C	0.52527200	-1.93746200	-2.97357100
C	2.49938800	-3.41210800	-0.10332700
H	2.05263400	-3.73387500	0.84138500
H	2.99266700	-4.28437100	-0.55797600
H	3.25639100	-2.66195400	0.13225100

C 3.04889900 -1.44421000 -2.53028800
H 3.65747800 -2.18791500 -3.06434400
H 2.96568400 -0.56466800 -3.17446500
H 3.59451500 -1.16916600 -1.62319700
C 0.31973300 -1.19152600 -4.25882700
H 0.27948300 -1.88398400 -5.11122300
H -0.61868300 -0.62753900 -4.25072400
H 1.13267300 -0.48493100 -4.44905800
C -1.90249600 -2.89565700 -2.77779200
H -1.92745500 -3.69111200 -3.53579200
H -2.58364500 -3.18059300 -1.97180500
H -2.28853100 -1.98186500 -3.23685300
C -0.52710000 -4.43504100 -0.29660100
H -0.21918900 -4.35593300 0.74903700
H -1.61950100 -4.40464700 -0.32701100
H -0.21353200 -5.42111700 -0.66659100
C 5.91408300 -0.13401800 0.86421000
C 3.72780800 0.02977000 1.57445300
C 4.02584800 1.22216400 2.30748100
C 5.35352800 1.70943200 2.29075500
C 6.30850400 1.02734200 1.57010200
H 6.65379600 -0.68079300 0.27965700
C 2.95248800 1.85080300 2.98957900
H 5.59966200 2.61354200 2.84325200
H 7.34031200 1.36241300 1.53065200
C 1.70595300 1.28748700 2.93693600
C 1.50156800 0.06775900 2.21023400
H 3.12836100 2.77764100 3.53064900
H 0.86347600 1.76653600 3.42322800
N 4.68705700 -0.62264800 0.85385100
N 2.48696600 -0.52154800 1.54889100
C 0.16375200 -0.58647600 2.26552300
C -0.44947900 -0.60778400 3.53102400
C -1.68976500 -1.19656400 3.77154200
C -2.34893800 -1.79776200 2.69769000
C -1.74421000 -1.79579100 1.42977300
C -0.50161200 -1.19889500 1.15872800
H 0.07274200 -0.16608200 4.37590000
H -2.11128000 -1.18427400 4.77003600
H -2.30894600 -2.26474800 0.62967000
Ir 0.10325700 -1.22118800 -0.82228900
H -2.54181400 0.20757700 0.46610500
O -3.57329300 -2.39592600 2.76706800
C -4.28502100 -2.33018100 3.99783100
H -4.47683500 -1.29177400 4.29616600
H -5.23836000 -2.83225100 3.81890100
H -3.75087200 -2.85119600 4.80323700
C -3.39083900 2.35309800 -1.86248100
C -3.56096900 1.55459200 -0.71150700
C -4.62164500 1.87445800 0.18911800
C -5.43078800 2.99514800 -0.07147500
C -5.24232900 3.78555100 -1.19518400
C -4.21833000 3.44581000 -2.09164600
H -2.60305200 2.09815700 -2.55992600
H -6.22181600 3.21017100 0.64089700
H -5.87983600 4.64424800 -1.38317800
H -4.06012000 4.04584500 -2.98478900
C -4.93271000 1.04601600 1.36348400
H -4.37464400 0.09327600 1.45530100
O -5.77329400 1.32553300 2.20932800
N -2.75938500 0.44635900 -0.49608400
O -1.59510100 0.35863200 -1.28352300
C 1.37845400 1.57455700 -1.41163000
O 0.52673900 0.88749600 -0.49686700
H -0.65947400 0.93895600 -0.84572800
H 2.37063600 1.11375000 -1.38929200
H 0.99123000 1.46410000 -2.43854500
C 1.50534400 3.04757100 -1.07156000
C 2.76648300 3.62450600 -0.87954600
C 0.37146500 3.86803100 -0.97667200
C 2.89764500 4.98794800 -0.60247000
H 3.65498900 3.00033300 -0.94516300
C 0.49726300 5.22754300 -0.69141100
H -0.61798500 3.44362900 -1.12745500
C 1.76220700 5.79326300 -0.50545200
H 3.88595700 5.41761900 -0.45803300
H -0.39286800 5.84738200 -0.61874900
H 1.85986200 6.85358100 -0.28659300

17
1a-3 SCF Done: E(RM06) = -475.918672462
C -1.25635900 -1.13751100 -0.04698200
C -0.65583400 0.12972000 -0.02729600
C 0.76166800 0.22292700 -0.00250400
C 1.52658500 -0.95610400 0.03599200
C 0.92677900 -2.20727400 0.02847500
C -0.47040600 -2.28647000 -0.01978100
H -2.33651600 -1.20595200 -0.06169600
H 2.60680700 -0.84983000 0.05981700
H 1.53020600 -3.10962000 0.05354100
H -0.95854200 -3.25774700 -0.02749900
C 1.46815100 1.51562300 -0.06162700
H 0.83576900 2.41352400 -0.22015000
O 2.67975600 1.64534900 0.02643300
N -1.43287300 1.30456200 -0.09655900
H -1.13781200 2.01863700 0.56302300
O -2.81425400 1.08637100 0.16716500
H -3.23732600 1.31982300 -0.67757000

70
TS10 SCF Done: E(RM06) = -1603.22749750
C -1.45744300 0.70661000 2.39596000
C -0.12445800 0.13192000 2.55090900
C -0.22893200 -1.27623800 2.27496900
C -2.34763600 -0.36993200 2.02546800
C -1.59523100 -1.59339400 1.96326600
C 1.08085100 0.84345300 3.08899700
H 1.02385000 1.91820500 2.89399700
H 1.14968600 0.70493200 4.17799200
H 1.99965600 0.47585500 2.62768800
C 0.88694300 -2.27180900 2.35670000
H 1.86663300 -1.79922000 2.25813200
H 0.85243300 -2.78700700 3.32785800
H 0.79996600 -3.03660400 1.57801700
C -2.14722600 -2.96552700 1.71228300
H -2.40790400 -3.46081900 2.65902500

H	-3.05295400	-2.93439100	1.09997200
H	-1.42335700	-3.60579000	1.19962000
C	-3.83092400	-0.24915400	1.82742700
H	-4.35754700	-0.45134400	2.77046900
H	-4.11340400	0.75497700	1.49902600
H	-4.19855100	-0.96184300	1.08305000
C	-1.86539300	2.08655100	2.82032900
H	-1.09327400	2.82551900	2.59274400
H	-2.78806400	2.40809000	2.32828500
H	-2.04339000	2.11112500	3.90493100
C	5.06735000	-2.11422100	1.27171400
C	3.74079300	-0.63984200	0.10000300
C	4.44964100	-0.92066700	-1.11108700
C	5.51072700	-1.85490800	-1.06928000
C	5.82657500	-2.45721300	0.12784900
H	5.30125300	-2.58149000	2.22828500
C	4.03706800	-0.23420000	-2.28228800
H	6.06022300	-2.08291200	-1.98010400
H	6.63409400	-3.17848400	0.20780300
C	3.00262000	0.65865500	-2.20091300
C	2.35195500	0.88765700	-0.94403000
H	4.54078000	-0.42996500	-3.22639000
H	2.65267200	1.18695600	-3.08138800
N	4.06956200	-1.24945300	1.27551100
N	2.71660300	0.25182300	0.15882500
C	1.29969200	1.94370600	-0.87130600
C	1.66943800	3.19293800	-1.39890600
C	0.82529300	4.30257000	-1.38950500
C	-0.45221500	4.14857600	-0.84689500
C	-0.84025900	2.90176700	-0.33505800
C	-0.00195600	1.77639300	-0.31367500
H	2.66961400	3.31403100	-1.80928400
H	1.16852900	5.24801400	-1.79401200
H	-1.85314300	2.83316800	0.04962100
Ir	-0.79465500	0.01101200	0.44394400
O	-1.39373700	5.13806200	-0.77683400
C	-1.05860700	6.41659600	-1.29414600
H	-0.83187300	6.37512200	-2.36819300
H	-1.93953000	7.04465700	-1.14037000
H	-0.20582100	6.86071900	-0.76309200
C	-4.07902700	-2.60879300	-2.92371100
C	-3.23502800	-1.55039800	-2.58975900
C	-1.96214800	-1.79745200	-2.05658500
C	-1.53748700	-3.11983300	-1.88141200
C	-2.38005800	-4.17966500	-2.22288500
C	-3.65309500	-3.92830200	-2.74013600
H	-5.06570200	-2.40612800	-3.33233700
H	-2.03945400	-5.20359200	-2.09052600
H	-4.30742500	-4.75430100	-3.00643300
C	-1.02907400	-0.65479600	-1.75955700
H	-1.82846600	0.23830200	-0.87711200
H	-1.06616600	0.13489800	-2.53045200
O	0.16252100	-0.89653200	-1.24632200
H	-0.54071500	-3.29989200	-1.49170300
H	-3.56415800	-0.52387400	-2.74233000

73

IN12 SCF Done: E(RM06) = -1733.74794620

C	0.77871700	2.97355300	1.19831900
C	-0.62981600	2.58037500	1.15139000
C	-1.12373900	2.89285100	-0.17772300
C	1.15260200	3.39735800	-0.09466200
C	-0.01780300	3.34363300	-0.96730900
C	-1.47361200	2.28285000	2.35707700
H	-0.90166400	1.73832400	3.11458500
H	-1.82790800	3.21825800	2.81631200
H	-2.33921500	1.66911900	2.10156300
C	-2.56370000	2.86448200	-0.59946900
H	-3.03157000	3.84011900	-0.40128100
H	-2.67025900	2.65766100	-1.66912900
H	-3.12723300	2.10706000	-0.04898900
C	-0.10014500	3.94346700	-2.34646900
H	-0.33482800	5.01747100	-2.29736900
H	0.84656300	3.86231800	-2.89434900
H	-0.88065400	3.47076300	-2.95135900
C	2.50482600	3.88353500	-0.53291100
H	2.49580300	4.97120800	-0.68947200
H	3.27702500	3.66489700	0.21036500
H	2.81309500	3.42329100	-1.47799100
C	1.62520200	2.96152600	2.43682500
H	1.46067900	2.05716900	3.03005000
H	2.69347300	3.01740700	2.20573200
H	1.38323700	3.82138400	3.07816500
C	-5.80963600	-0.09481200	0.78576700
C	-3.68584200	-0.98194100	0.84624800
C	-4.15431400	-2.21810100	0.29713300
C	-5.53271400	-2.34441500	0.00206800
C	-6.36953300	-1.28014200	0.24934800
H	-6.45674500	0.76016500	0.98124000
C	-3.19373300	-3.23822600	0.07895900
H	-5.90919000	-3.27618600	-0.41450600
H	-7.43380500	-1.33135900	0.04104800
C	-1.88328300	-3.00401000	0.40602600
C	-1.50165300	-1.74135900	0.96769100
H	-3.50563800	-4.18699200	-0.35244200
H	-1.12293300	-3.75686700	0.22772000
N	-4.53197800	0.06465100	1.07525600
N	-2.38493400	-0.77477900	1.17320400
C	-0.08206600	-1.54078800	1.38113900
C	0.46313900	-2.56128200	2.17834100
C	1.77288500	-2.53772200	2.65718200
C	2.57473400	-1.44728600	2.31475500
C	2.04175100	-0.42532000	1.51257700
C	0.72662900	-0.42174900	1.01064900
H	-0.16581500	-3.40406100	2.45678800
H	2.13914200	-3.34922200	3.27544100
H	2.71563200	0.39798600	1.28224100
Ir	0.26336000	1.16213400	-0.24476600
O	3.87607400	-1.28466100	2.69527400
C	4.50059500	-2.34340100	3.40869400
H	4.49532500	-3.27569000	2.82941000
H	5.53408400	-2.02821000	3.57112700
H	4.02284400	-2.51579800	4.38236600
H	-0.80199000	0.16820200	-0.87490200
C	0.25381000	-1.22719000	-3.06456300
C	1.35470000	-1.13166600	-2.20912800

C 2.11385900 -2.27872500 -1.90172600
C 1.71398100 -3.51613900 -2.44298500
C 0.61998000 -3.61075900 -3.28925400
C -0.10607100 -2.45561400 -3.60738000
H -0.32204500 -0.33673700 -3.28724600
H 2.30511100 -4.38934900 -2.18667400
H 0.33157900 -4.57136300 -3.70612600
H -0.96312600 -2.51438500 -4.27232200
C 3.34205400 -2.27111000 -1.06892800
H 3.73465000 -1.28639100 -0.75192000
O 3.94681700 -3.28031700 -0.75080400
N 1.66354700 0.17488500 -1.65305900
O 1.97728500 1.07526000 -2.74939200
H 2.54073400 0.12986100 -1.13865000
H 1.22824500 1.69879400 -2.69701900

73
TS11 SCF Done: E(RM06) = -1733.71965832
C -1.16676400 0.32320400 2.58041800
C 0.14907100 -0.27639500 2.58307800
C -0.00264800 -1.63788500 2.14025800
C -2.11650400 -0.67405800 2.15516500
C -1.39928200 -1.88690800 1.89278900
C 1.39803400 0.33239500 3.15216600
H 1.35378400 1.42506000 3.12888500
H 1.52714800 0.02776900 4.20066500
H 2.28275300 0.02826500 2.58893200
C 1.08611300 -2.66979200 2.10159700
H 1.12242300 -3.20794700 3.05990400
H 0.91346900 -3.41169800 1.31605400
H 2.06729700 -2.21831900 1.93366600
C -2.00779400 -3.22281400 1.58522600
H -2.25984100 -3.74471400 2.52004300
H -2.92967000 -3.13292100 1.00463300
H -1.32495600 -3.86637200 1.02624000
C -3.60688300 -0.50788500 2.10971800
H -3.89667600 0.51927900 1.86941800
H -4.06762700 -1.16790600 1.36948100
H -4.04966800 -0.75253200 3.08612000
C -1.50572300 1.67226700 3.14058500
H -0.70989600 2.39863300 2.96051300
H -2.43038800 2.07591000 2.71747000
H -1.64816400 1.59789400 4.22837700
C 5.39457000 -2.30816700 0.72190700
C 4.07344700 -0.61960900 -0.11075000
C 4.79562200 -0.61841300 -1.34557700
C 5.86669100 -1.53146400 -1.49818600
C 6.17236200 -2.38061100 -0.45970100
H 5.62047800 -2.97725000 1.55172700
C 4.37890100 0.29702500 -2.34475900
H 6.42810300 -1.54917100 -2.42946500
H 6.98496700 -3.09692400 -0.53024300
C 3.32754500 1.13887700 -2.08680400
C 2.66882400 1.08395600 -0.81634100
H 4.88766000 0.31267600 -3.30576400
H 2.97048400 1.83025600 -2.84204000
N 4.38870600 -1.47327700 0.90480400
N 3.03614200 0.22620900 0.12581200
C 1.56327000 2.04936400 -0.54443800
C 1.85948600 3.39295800 -0.83573500
C 0.94848300 4.43231800 -0.66221800
C -0.33136900 4.11358600 -0.20406500
C -0.64811700 2.77562000 0.07504000
C 0.26401500 1.71993200 -0.05744500
H 2.85507000 3.64134000 -1.19503700
H 1.24081100 5.45140500 -0.88763600
H -1.66298600 2.58082000 0.40727300
Ir -0.50999900 -0.14387800 0.42199600
O -1.33580700 5.01423700 -0.00230200
C -1.07557600 6.38229900 -0.28737200
H -0.82060200 6.53441800 -1.34440400
H -2.00094200 6.91747400 -0.06229100
H -0.26848400 6.78094400 0.34131800
C -2.79673700 -1.98568100 -1.81687400
C -2.60418600 -0.59601900 -1.66976100
C -3.68753400 0.27744100 -1.84309200
C -4.96084700 -0.22711700 -2.10515500
C -5.16709600 -1.60684300 -2.21095100
C -4.08638700 -2.47330100 -2.07502100
H -5.79309600 0.46090400 -2.22756800
H -6.15957300 -1.99739500 -2.41716800
H -4.20817600 -3.54603700 -2.19312700
N -1.34967800 -0.03545000 -1.30179700
H -0.97455900 0.58699300 -2.03259800
O -0.18150300 -0.51367100 -3.09872200
H 0.61671700 -0.62918400 -2.55396100
H 0.88577600 -0.57286600 -0.16438800
H -3.52429600 1.34729200 -1.74798300
C -1.64980700 -2.93251700 -1.82885000
H -0.67076300 -2.46489400 -2.01634300
O -1.78418200 -4.14301200 -1.71962900

73
IN13 SCF Done: E(RM06) = -1733.78227305
C -0.90059000 -2.52363600 1.57523300
C 0.50600200 -2.53087000 1.28780100
C 0.65267900 -2.94256700 -0.08301100
C -1.61708100 -3.03214200 0.41953100
C -0.66538500 -3.28142700 -0.60378700
C 1.61004000 -2.32003500 2.28153300
H 1.31099100 -1.62137300 3.06855500
H 1.86854500 -3.27316600 2.76586300
H 2.50740700 -1.91672800 1.80988700
C 1.95512600 -3.21145200 -0.77892000
H 2.25259700 -4.25860900 -0.62322700
H 1.87749600 -3.04979800 -1.85844000
H 2.75568100 -2.57284000 -0.39596000
C -0.95978600 -3.86613900 -1.95402500
H -1.09440700 -4.95459100 -1.87651100
H -1.87537800 -3.44283000 -2.38050000
H -0.13911600 -3.68859100 -2.65541100
C -3.08988600 -3.30293900 0.33654100
H -3.29696700 -4.35788800 0.56891200
H -3.65435500 -2.69809300 1.05258700
H -3.48246600 -3.09829900 -0.66386100
C -1.50831700 -2.25603200 2.91913700

H	-0.94391500	-1.50417900	3.47588200
H	-2.54339000	-1.91111300	2.84364300
H	-1.51373300	-3.17843900	3.51728800
C	5.81644100	-0.86937200	-0.26527200
C	3.97188300	0.41837800	0.22016400
C	4.63943200	1.60090100	-0.22883400
C	5.96678500	1.48379500	-0.70554600
C	6.56351100	0.24357800	-0.72171000
H	6.27126700	-1.85948500	-0.27676900
C	3.91330000	2.81808400	-0.17126200
H	6.49438800	2.37060900	-1.04938000
H	7.58059200	0.10540800	-1.07497300
C	2.63524800	2.81512100	0.32317800
C	2.05069100	1.58896400	0.78447000
H	4.37416400	3.73598500	-0.52905200
H	2.05112800	3.72812800	0.34857600
N	4.57784700	-0.80320700	0.18668500
N	2.70691900	0.43903900	0.71456400
C	0.69018200	1.62664000	1.39089200
C	0.45486300	2.69601900	2.27476100
C	-0.75777500	2.90087800	2.92777800
C	-1.80024500	2.00640100	2.67638000
C	-1.58518800	0.93200900	1.79994700
C	-0.36746600	0.69876600	1.14758400
H	1.26561400	3.38871300	2.48436900
H	-0.87032700	3.73668500	3.60873700
H	-2.43297100	0.27628200	1.62849500
Ir	-0.43310100	-0.93463000	-0.12001400
O	-3.04387100	2.08134300	3.22512800
C	-3.33519600	3.17735800	4.08289400
H	-3.22233700	4.13755500	3.56307300
H	-4.37788000	3.05349000	4.38346400
H	-2.69951100	3.17139900	4.97824700
H	0.89160400	-0.24908100	-0.60088300
C	-0.04420500	1.48839700	-2.63996100
C	-1.28084100	1.09768600	-2.06708200
C	-2.39251900	1.98437700	-2.22637700
C	-2.22508100	3.20804300	-2.89148300
C	-1.00358400	3.56815900	-3.44632000
C	0.08309700	2.69038300	-3.32459200
H	0.79656000	0.80795800	-2.55426800
H	-3.08905600	3.86227300	-2.96290300
H	-0.89177600	4.51349600	-3.96949500
H	1.04428000	2.95267700	-3.76045200
C	-3.72345700	1.64636700	-1.68418800
H	-3.81891300	0.63739200	-1.24452200
O	-4.68848700	2.39680800	-1.72511400
N	-1.45112700	-0.12431400	-1.44970900
O	-3.52663000	-1.73792400	-2.59120000
H	-2.79672200	-1.18990800	-2.20961300
H	-3.52992400	-1.51111600	-3.53430800

73

TS11' SCF Done: E(RM06) = -1733.67223992

C	0.51144400	2.74310500	1.48971500
C	-0.87353600	2.36576600	1.21476800
C	-1.17752500	2.75764500	-0.15037900
C	1.05073100	3.26142000	0.28777600
C	0.00256100	3.34976200	-0.70931800
C	-1.87724300	1.98489600	2.26178700
H	-1.40827100	1.41630400	3.07070900
H	-2.32149600	2.88887800	2.70371800
H	-2.67495700	1.36924500	1.84229600
C	-2.54155700	2.75645700	-0.77776500
H	-3.03300400	3.72594200	-0.61005300
H	-2.48657900	2.59534500	-1.85896600
H	-3.18276300	1.98008200	-0.35537900
C	0.18401700	3.84523100	-2.10003400
H	0.79779400	4.75371400	-2.13520900
H	0.71358300	3.07192400	-2.69346900
H	-0.77276300	4.06123500	-2.58514800
C	2.45867900	3.72878200	0.06072900
H	2.51763100	4.82493200	0.11069900
H	3.14800800	3.33102700	0.81182200
H	2.82018500	3.43269300	-0.93014500
C	1.17395600	2.67879000	2.83220800
H	0.90717700	1.76787800	3.37653500
H	2.26542200	2.71114700	2.75235500
H	0.86834000	3.53218400	3.45562500
C	-6.07570900	0.07008100	-0.83127800
C	-4.08744200	-0.88581800	-0.17604000
C	-4.42874300	-2.11425300	-0.82605500
C	-5.67091300	-2.19715400	-1.50008700
C	-6.50293800	-1.10147700	-1.50358200
H	-6.72169800	0.94778800	-0.82528600
C	-3.49060100	-3.17383600	-0.74995200
H	-5.94861900	-3.12123700	-2.00219200
H	-7.46549000	-1.11833500	-2.00521500
C	-2.32225800	-2.98280200	-0.05822000
C	-2.06099800	-1.72139700	0.57175000
H	-3.70611400	-4.11994900	-1.24167500
H	-1.58134400	-3.77245800	0.00940700
N	-4.92640800	0.18944500	-0.19425500
N	-2.92053500	-0.71546500	0.49896500
C	-0.80770700	-1.58142800	1.37075800
C	-0.58253500	-2.60264500	2.30867700
C	0.55242200	-2.66307200	3.11747500
C	1.52350800	-1.67287100	2.95883700
C	1.31956600	-0.65277200	2.01702800
C	0.17394100	-0.55512200	1.20843000
H	-1.33467500	-3.37928700	2.42787100
H	0.66022200	-3.46394400	3.84000400
H	2.11378600	0.08219900	1.92689100
Ir	0.22155000	1.00575600	-0.11338400
O	2.69121500	-1.60606000	3.66239100
C	2.95468500	-2.62290800	4.61888800
H	3.00305200	-3.61584700	4.15243100
H	3.92894000	-2.38381400	5.05164400
H	2.20082900	-2.63453500	5.41735000
H	-0.67456600	0.03044200	-0.99641800
C	1.37984800	-2.14435400	-1.65015800
C	2.39691800	-1.16368500	-1.50868000
C	3.70539700	-1.45887500	-2.02357700
C	3.95464100	-2.72759300	-2.56812600
C	2.95045700	-3.68164000	-2.67433100
C	1.65456700	-3.37349500	-2.22445600

H 0.39280200 -1.91004400 -1.27142900
H 4.95940600 -2.93112000 -2.92567000
H 3.16278600 -4.65394100 -3.10911400
H 0.86096300 -4.11073000 -2.31341900
C 4.80653200 -0.48145200 -2.03157100
H 4.57253600 0.53972800 -1.66803900
O 5.93895500 -0.72286900 -2.42600900
N 2.09280100 0.01557600 -0.89620700
O 1.54305300 1.21454400 -2.31229700
H 2.89609000 0.61758400 -0.76008500
H 0.83162900 0.68025900 -2.71536600

73
IN13' SCF Done: E(RM06) = -1733.77494719
C 0.68379400 2.78410500 1.18380100
C -0.72829500 2.41575100 1.16500900
C -1.25532900 2.84003800 -0.09442300
C 0.99762800 3.48088700 -0.02593400
C -0.18181000 3.52947600 -0.80904700
C -1.51370100 1.97467300 2.36276700
H -0.91996200 1.33455100 3.02053800
H -1.81978700 2.85795000 2.94167800
H -2.40399200 1.41606400 2.07354300
C -2.68816900 2.81839600 -0.53569000
H -3.16271000 3.79168500 -0.34519500
H -2.75766200 2.61389600 -1.60836700
H -3.26002600 2.05043500 -0.00851200
C -0.34329100 4.11507700 -2.16431400
H 0.54044900 4.67066600 -2.48902400
H -0.53106900 3.28127500 -2.86214500
H -1.21156000 4.78353000 -2.20519300
C 2.33485200 4.05963200 -0.38856900
H 2.42454200 5.09769500 -0.04008500
H 3.15547300 3.49626400 0.06671600
H 2.49429700 4.06224700 -1.47090700
C 1.55863600 2.72712100 2.39629600
H 1.34345400 1.85844600 3.02073400
H 2.62314100 2.71513900 2.14577200
H 1.37707300 3.62574600 3.00407200
C -5.87472900 -0.42055400 0.30920800
C -3.72331700 -1.16631200 0.62992300
C -4.09501500 -2.49942300 0.27096400
C -5.44318000 -2.74819900 -0.08146800
C -6.34089000 -1.70548100 -0.06222300
H -6.57279400 0.41589800 0.32905200
C -3.07629600 -3.48596100 0.28559300
H -5.74836500 -3.75406400 -0.36073600
H -7.38481800 -1.84809100 -0.32345800
C -1.80278900 -3.13283000 0.65035000
C -1.51813600 -1.77615300 1.01339200
H -3.31284800 -4.50713700 -0.00435800
H -1.00010100 -3.86140700 0.63805800
N -4.62753300 -0.14582200 0.64260200
N -2.45471100 -0.83841100 0.98888000
C -0.13534900 -1.42873800 1.45767200
C 0.43438700 -2.32730800 2.37949600
C 1.72557400 -2.19737900 2.88146600
C 2.50342100 -1.13320300 2.42110700
C 1.95510100 -0.23415500 1.49532000
C 0.64696200 -0.32291900 1.00791400
H -0.16799900 -3.15907600 2.73479300
H 2.10238500 -2.91677200 3.59922900
H 2.61842300 0.55223200 1.15322400
Ir 0.10654800 1.09654500 -0.45534600
O 3.78716500 -0.87953000 2.79883200
C 4.42368800 -1.80196800 3.67505400
H 4.47044200 -2.80698000 3.23677600
H 5.43915300 -1.42654400 3.81875700
H 3.91738400 -1.85120900 4.64815000
H -0.98502900 -0.00037600 -0.51954400
C 0.52700800 -1.83684900 -2.34427500
C 1.70866200 -1.19595300 -1.87563800
C 2.92048000 -1.96454800 -1.91342900
C 2.90695500 -3.27819300 -2.43416300
C 1.74639600 -3.87083400 -2.89179100
C 0.55001500 -3.12884700 -2.83568400
H -0.39334900 -1.26416900 -2.34796400
H 3.85369500 -3.81061800 -2.44814600
H 1.75360400 -4.88294600 -3.28624200
H -0.37725500 -3.57106700 -3.19425300
C 4.19992300 -1.46213900 -1.41073200
H 4.19051900 -0.45132700 -0.94985800
O 5.26451400 -2.07040400 -1.45024600
N 1.65660600 0.12196900 -1.48433000
O -0.54685800 1.18853900 -2.37911100
H 2.55022600 0.45472400 -1.14108400
H 0.27265600 0.96311000 -2.85796400

86
IN14 SCF Done: E(RM06) = -2003.98849307
C -1.21366300 0.27128100 2.81043500
C 0.11273600 0.81232700 2.84211700
C 1.05969800 -0.28386200 2.91186900
C -1.07831100 -1.16175000 2.71043500
C 0.33171200 -1.49629500 2.80868000
C 0.48799000 2.25656400 2.99126200
H -0.33017900 2.91696800 2.69365000
H 0.73495900 2.47988900 4.03900400
H 1.35991200 2.49866100 2.37717000
C 2.52274600 -0.13043500 3.19359800
H 2.66644100 0.17971500 4.23955100
H 3.06012000 -1.07361100 3.06041700
H 2.99881900 0.62812000 2.56385900
C 0.89039200 -2.88617300 2.88629500
H 1.00296900 -3.20277700 3.93290600
H 0.23440000 -3.61106200 2.39566000
H 1.87377100 -2.95564800 2.41227900
C -2.20809000 -2.15050700 2.75969900
H -2.42063300 -2.43261400 3.80081300
H -3.12483500 -1.73622600 2.33260200
H -1.97078900 -3.06691100 2.21129300
C -2.49520500 1.01809900 3.04545100
H -2.42650600 2.05857400 2.71919700
H -3.33985200 0.55927100 2.52252900
H -2.73703700 1.02414000 4.11798600
C 5.87741200 1.42105700 0.73192200

C	3.82924300	1.67423400	-0.28789400
C	4.43906700	1.67460600	-1.58184400
C	5.84617000	1.54648100	-1.66198800
C	6.57415100	1.42383000	-0.50040700
H	6.43586800	1.31623600	1.66188800
C	3.58404700	1.78904400	-2.70726300
H	6.32921700	1.54421100	-2.63630400
H	7.65505700	1.32470300	-0.51289900
C	2.23472500	1.92400100	-2.51021600
C	1.70889800	1.94939900	-1.17830700
H	4.00632400	1.76368200	-3.70909700
H	1.55197800	2.00101900	-3.34926300
N	4.56747000	1.53699300	0.85007600
N	2.48538100	1.80361900	-0.11300900
C	0.26346900	2.26441000	-0.99036900
C	-0.16017800	3.44306200	-1.63012100
C	-1.45249800	3.94926800	-1.51963400
C	-2.37128100	3.23221500	-0.75190800
C	-1.97448700	2.03815100	-0.13328200
C	-0.67576800	1.52412000	-0.22042400
H	0.56269100	4.00669600	-2.21466800
H	-1.71789700	4.87220600	-2.02196800
H	-2.74195100	1.49727900	0.40450700
Ir	-0.30032300	-0.26982200	0.76998500
O	-3.67389000	3.59547800	-0.55497800
C	-4.13807700	4.78201100	-1.18509600
H	-4.06315300	4.71622500	-2.27849300
H	-5.18912800	4.88033800	-0.90410500
H	-3.58969100	5.66750000	-0.83711700
H	-1.71332600	-0.52056300	0.13714000
C	2.63503700	-1.65883800	-0.06663100
C	1.46931900	-1.89617000	-0.84172000
C	1.49463000	-3.02943200	-1.71788100
C	2.58622500	-3.90934700	-1.70523300
C	3.70237000	-3.66445500	-0.91368800
C	3.72835600	-2.51510400	-0.11173800
H	2.67065000	-0.76550400	0.54377500
H	2.54282700	-4.77378700	-2.36133200
H	4.55184300	-4.34139000	-0.93425000
H	4.60789100	-2.28698200	0.48565900
C	0.41526400	-3.28540600	-2.69533600
H	-0.24992000	-2.43352800	-2.90295500
O	0.27920500	-4.34401600	-3.29554300
N	0.36303100	-1.06471100	-0.80792100
C	-2.87347900	-1.79309900	-2.67463900
O	-1.88210300	-0.79284200	-2.84200700
H	-1.24435800	-0.82710900	-2.09470400
H	-3.30180900	-1.95412400	-3.67367600
H	-2.43316800	-2.75497700	-2.37091600
C	-3.99443700	-1.43087500	-1.71345300
C	-4.66683600	-2.43151800	-0.99903300
C	-4.41090700	-0.10208000	-1.56463000
C	-5.73537400	-2.11391200	-0.15675200
H	-4.35217000	-3.46833800	-1.10402600
C	-5.47226700	0.22053200	-0.71615200
H	-3.88449900	0.67657900	-2.10661000
C	-6.13987200	-0.78435400	-0.01037100
H	-6.24693800	-2.90341100	0.38869700
H	-5.76967800	1.25990500	-0.60363300
H	-6.96776500	-0.53384500	0.64830600

86

TS12 SCF Done: E(RM06) = -2003.95986019

C	1.09030800	-1.30137500	2.58060000
C	-0.27281700	-1.73358700	2.58479500
C	-1.11185200	-0.63817000	3.04343500
C	1.07572100	0.11831000	2.84679900
C	-0.29475600	0.50270500	3.17662200
C	-0.76504300	-3.13284300	2.37170300
H	-0.05802400	-3.72300900	1.78353900
H	-0.90340500	-3.64015900	3.33718300
H	-1.72629200	-3.13418400	1.85011200
C	-2.56755400	-0.76526900	3.36090500
H	-2.72721700	-1.58189400	4.07819000
H	-2.95967000	0.15048500	3.81328700
H	-3.17172800	-0.98416200	2.47216500
C	-0.68779000	1.86672800	3.66264300
H	-0.30710200	2.03533400	4.67965000
H	-0.27357300	2.65862200	3.03001200
H	-1.77257500	1.99657900	3.68968600
C	2.28361900	0.97630200	3.08817200
H	2.58657500	0.91046000	4.14344800
H	3.13299800	0.66274700	2.47629300
H	2.08192800	2.02825400	2.86702100
C	2.31148700	-2.17368300	2.56196000
H	2.14649600	-3.10132200	2.00967300
H	3.17336400	-1.66561400	2.11995600
H	2.58398400	-2.44544800	3.59181100
C	-5.86290900	-0.24422700	0.58906200
C	-3.95110200	-0.87974400	-0.52774200
C	-4.58589400	-0.66554900	-1.79165800
C	-5.92815100	-0.21900700	-1.80562900
C	-6.57641400	-0.00879400	-0.60999300
H	-6.35726200	-0.08192600	1.54667500
C	-3.81620400	-0.90342900	-2.95742400
H	-6.42549500	-0.04862000	-2.75758300
H	-7.60643300	0.33138100	-0.57153200
C	-2.52725600	-1.34740300	-2.82680300
C	-1.97464600	-1.56775400	-1.52263300
H	-4.25143800	-0.72174200	-3.93725000
H	-1.90809300	-1.51246100	-3.70112200
N	-4.60980100	-0.65598200	0.64393800
N	-2.66596000	-1.31559500	-0.41792300
C	-0.60357200	-2.14028700	-1.42952600
C	-0.32664900	-3.20087000	-2.31265000
C	0.90546000	-3.84462200	-2.37271300
C	1.92816700	-3.38679700	-1.53888900
C	1.67879900	-2.32129600	-0.66164900
C	0.43275300	-1.69954500	-0.55772200
H	-1.11986500	-3.55619000	-2.96482600
H	1.05164200	-4.66984200	-3.05989800
H	2.51775100	-1.97566300	-0.07178000
Ir	0.21326700	-0.16813100	0.81995600
O	3.19045800	-3.89578800	-1.50211300
C	3.52283000	-4.93486200	-2.41390700
H	3.39623800	-4.61365500	-3.45583100

H	4.57559900	-5.16449400	-2.23533200
H	2.92359100	-5.83767300	-2.23582500
H	1.60450000	0.13546700	0.11069400
C	-2.23703400	2.20085400	0.46934400
C	-1.18216000	2.17773300	-0.47587800
C	-1.17768400	3.18698600	-1.49147700
C	-2.17483500	4.17388100	-1.48961900
C	-3.17978400	4.19348200	-0.53115600
C	-3.20885400	3.18987000	0.44801200
H	-2.28519100	1.41208300	1.21070300
H	-2.13580400	4.92066400	-2.27662100
H	-3.94321800	4.96609600	-0.54993500
H	-4.00307400	3.17520500	1.19000700
C	-0.17979100	3.23706000	-2.59744000
H	0.62249200	2.47948000	-2.59441000
O	-0.23364400	4.07734500	-3.48603700
N	-0.21338100	1.18870700	-0.45338500
C	2.78246000	2.32910100	-0.95141400
O	2.07408000	1.26460200	-1.46753200
H	0.73831700	1.30908900	-1.08812900
H	2.69263100	3.23700900	-1.59265900
H	2.40051900	2.66076800	0.04349900
C	4.27705200	2.05762400	-0.78688600
C	5.10823400	2.98111700	-0.13525200
C	4.84940500	0.88859700	-1.29893600
C	6.47612500	2.74326600	0.00222400
H	4.67761500	3.89784000	0.26670700
C	6.21962000	0.64534600	-1.16236900
H	4.19863800	0.18313100	-1.80533700
C	7.03877600	1.57015000	-0.51181200
H	7.10458600	3.47119500	0.51094600
H	6.64832100	-0.26887600	-1.56780300
H	8.10456500	1.38195200	-0.40556000

86

IN15 SCF Done: E(RM06) = -2004.03664376

C	-1.39447900	-2.04740300	-2.07294600
C	0.05676600	-2.13927300	-2.19354800
C	0.50583900	-0.91766200	-2.77829800
C	-1.80969300	-0.80321100	-2.72141100
C	-0.65242100	-0.10653300	-3.14084200
C	0.90071200	-3.33237900	-1.85883200
H	0.43474300	-3.94312800	-1.08044000
H	1.02771000	-3.96580100	-2.74849000
H	1.88654700	-3.03101000	-1.49855100
C	1.91188300	-0.58108900	-3.16550900
H	2.06561000	-0.83312400	-4.22550800
H	2.11521300	0.48972300	-3.06033100
H	2.65081000	-1.12734300	-2.57729200
C	-0.59338300	1.16684300	-3.93332600
H	-0.43530800	0.95068000	-4.99974400
H	-1.52068200	1.74042700	-3.85279100
H	0.23222000	1.81137800	-3.61402200
C	-3.23720800	-0.41281400	-2.95076600
H	-3.61677700	-0.92517400	-3.84615200
H	-3.87647500	-0.70370500	-2.11229800
H	-3.35599000	0.66179400	-3.10331200
C	-2.31781700	-3.17703800	-1.72402100
H	-1.87453500	-3.85059900	-0.98677100
H	-3.26823600	-2.81531100	-1.31971900
H	-2.54485200	-3.76862100	-2.62259100
C	5.87496400	-0.96814000	-1.49107500
C	4.11010100	-0.97153000	-0.01036900
C	4.88535200	-0.25927100	0.95906300
C	6.21654400	0.09138500	0.63150700
C	6.71967900	-0.26478500	-0.59944100
H	6.25448900	-1.25865900	-2.47053500
C	4.25852000	0.05183100	2.19369800
H	6.82203500	0.63203000	1.35571700
H	7.73599500	-0.02036000	-0.89246300
C	2.97046700	-0.35900200	2.40894700
C	2.27392700	-1.09659100	1.39464400
H	4.80476800	0.61200700	2.94936300
H	2.46021500	-0.12429900	3.33666700
N	4.62813100	-1.31164900	-1.22525000
N	2.82869000	-1.36612900	0.22214600
C	0.93815200	-1.67083600	1.72774400
C	0.90737200	-2.39016200	2.93673000
C	-0.22352200	-3.05586600	3.40481900
C	-1.38614100	-2.98358200	2.63629400
C	-1.37725100	-2.24666800	1.44192000
C	-0.24758700	-1.57767000	0.93840900
H	1.81823300	-2.46075200	3.52663600
H	-0.17984600	-3.61104500	4.33479900
H	-2.31704600	-2.21210100	0.89938900
Ir	-0.57473600	-0.52252300	-0.82509600
H	-2.04890000	0.08733600	1.27706400
O	-2.57119600	-3.58019000	2.95510400
C	-2.64375900	-4.32141300	4.16509600
H	-2.44832000	-3.68853100	5.04087400
H	-3.66536900	-4.70392400	4.22257200
H	-1.94292200	-5.16697000	4.16560800
C	-2.70108500	2.39091100	-0.97207800
C	-2.86150000	1.49051700	0.11743300
C	-4.07417000	1.61315000	0.87502700
C	-5.00834400	2.62218000	0.55608000
C	-4.81287500	3.49665800	-0.49706600
C	-3.64187600	3.36345900	-1.26631900
H	-1.79615400	2.31176300	-1.56579200
H	-5.90196100	2.67579900	1.17143200
H	-5.54449900	4.26497000	-0.72905700
H	-3.46406700	4.03768300	-2.10172100
C	-4.42283200	0.70431100	1.97365200
H	-3.73356600	-0.14553800	2.15469400
O	-5.42206700	0.80867500	2.67670400
N	-1.84632500	0.61514000	0.43483200
C	1.18152200	2.34042900	-0.46864000
O	0.69511200	1.17544400	0.23538800
H	-0.17870400	1.38726200	0.66249000
H	2.08222900	2.01085600	-0.99061400
H	0.44313500	2.65504900	-1.21588200
C	1.48674600	3.47941700	0.47594500
C	0.47284200	4.37181200	0.85340700
C	2.77205900	3.65203500	1.00640300
C	0.73590600	5.40955300	1.74883000
H	-0.52765100	4.25480800	0.44209300

C 3.03914500 4.69210200 1.89810500
H 3.56585400 2.96844300 0.71612700
C 2.02054600 5.57165100 2.27293500
H -0.05977200 6.09328200 2.03256700
H 4.04182700 4.81790700 2.29866100
H 2.22781100 6.38211800 2.96685400

86
TS13 SCF Done: E(RM06) = -2004.03045835
C -1.65257800 -1.59177800 -2.24718800
C -0.22235700 -1.82832100 -2.37252100
C 0.37688500 -0.59953600 -2.80941500
C -1.90952300 -0.23589400 -2.71580600
C -0.67607500 0.37648800 -3.05652600
C 0.48752700 -3.13396200 -2.17426500
H -0.07838700 -3.79695500 -1.51375500
H 0.61260400 -3.64771000 -3.13808300
H 1.47238500 -2.97864700 -1.72736200
C 1.81444300 -0.40247600 -3.17654500
H 1.93200000 -0.54218900 -4.26151300
H 2.15993200 0.61044000 -2.94515000
H 2.47435600 -1.10920200 -2.67099600
C -0.47798800 1.73003000 -3.67403000
H -0.43810900 1.65613700 -4.77048600
H -1.29336600 2.41540300 -3.42480600
H 0.45987800 2.19097100 -3.34948900
C -3.27452700 0.36240500 -2.86452200
H -3.70054000 0.06728900 -3.83382100
H -3.95949700 0.01088700 -2.08781800
H -3.25517000 1.45386000 -2.82671300
C -2.70097600 -2.64092700 -2.01949400
H -2.34564800 -3.42649100 -1.34821700
H -3.61287500 -2.21777000 -1.58609600
H -2.97942000 -3.11645000 -2.97088400
C 5.74242600 -1.33565400 -1.65518200
C 3.99091700 -1.29995000 -0.15998000
C 4.81428900 -0.68398900 0.83513700
C 6.16215100 -0.40053900 0.51149600
C 6.63433200 -0.72803800 -0.73967700
H 6.09702100 -1.60206700 -2.65079000
C 4.21725300 -0.39444900 2.08937200
H 6.80399700 0.06867000 1.25393300
H 7.66170600 -0.53189400 -1.03071100
C 2.90651100 -0.73039600 2.29604500
C 2.15694200 -1.36291100 1.25098500
H 4.80321600 0.09369300 2.86471500
H 2.41736400 -0.51084900 3.23875700
N 4.47826400 -1.61373800 -1.39430300
N 2.68612800 -1.61768500 0.06337400
C 0.78473500 -1.86094300 1.55921700
C 0.70791000 -2.68952700 2.69200900
C -0.47015900 -3.30935200 3.10734900
C -1.62715200 -3.07685400 2.36263000
C -1.56869300 -2.22894700 1.24483900
C -0.39304800 -1.60057800 0.79934900
H 1.61458300 -2.88482700 3.26025500
H -0.46552100 -3.95343600 3.97917000
H -2.50324700 -2.07301200 0.71451400
Ir -0.62088200 -0.32540100 -0.82627800
H -1.92979500 0.25952700 1.43987100
O -2.85336000 -3.61127200 2.63647900
C -2.97512900 -4.46566700 3.76467600
H -2.72409700 -3.94452900 4.69812700
H -4.02326200 -4.77174500 3.79837600
H -2.34321100 -5.35870100 3.66779500
C -2.50004500 2.89555200 -0.43135300
C -2.75693100 1.78634300 0.40339800
C -4.06071100 1.67241500 0.96359400
C -5.03040200 2.65824900 0.68759300
C -4.75407000 3.74167500 -0.12887900
C -3.47235700 3.85226200 -0.68966900
H -1.51035600 3.00075000 -0.86412800
H -6.00864300 2.53220200 1.14174200
H -5.51217300 4.49290800 -0.32968600
H -3.22899900 4.69900600 -1.32701700
C -4.47228000 0.55149100 1.83071000
H -3.72171200 -0.24077700 2.01731400
O -5.58150100 0.44590800 2.33511700
N -1.71314900 0.88278200 0.66676000
C 1.31640800 2.14682900 -0.23991600
O 0.64640600 1.05901300 0.38076000
H -0.48285700 1.27912300 0.74674400
H 2.09809000 1.74900700 -0.89626500
H 0.62168500 2.71483800 -0.88127600
C 1.93972600 3.08520000 0.77723200
C 1.15188000 3.70450500 1.75887700
C 3.30899700 3.37355400 0.73833800
C 1.72023300 4.58429000 2.67975600
H 0.08532300 3.49727300 1.80408500
C 3.88171600 4.26014400 1.65451700
H 3.93330100 2.89635200 -0.01397700
C 3.08863300 4.86673600 2.62941300
H 1.09475500 5.05388300 3.43485900
H 4.94704900 4.47245800 1.60792500
H 3.53102600 5.55487600 3.34520000

102
IN16 SCF Done: E(RM06) = -2350.69258635
C 0.57823700 -2.93450100 -0.48780100
C 1.85507400 -3.17118300 0.16966900
C 2.91283700 -2.80427300 -0.78271900
C 0.85539900 -2.22430900 -1.69840300
C 2.31271500 -2.20265600 -1.89977200
C 2.06128100 -4.01248100 1.39629400
H 1.22152000 -3.91435200 2.08996500
H 2.15116400 -5.07539700 1.12996100
H 2.97214800 -3.72762900 1.93270300
C 4.36659400 -3.08737900 -0.56301200
H 4.56499100 -4.14904800 -0.76842700
H 5.00810200 -2.49301100 -1.21540200
H 4.66802000 -2.90112400 0.47210400
C 2.98684700 -1.61559900 -3.10358100
H 2.73391400 -2.18655600 -4.00706200
H 2.67194900 -0.58014700 -3.27539200
H 4.07473000 -1.61992500 -2.99921800
C -0.15611700 -1.77545300 -2.70944700

H	-0.36552100	-2.59452500	-3.41338100
H	-1.10204900	-1.48136500	-2.22717700
H	0.22043000	-0.93285800	-3.29948400
C	-0.76080500	-3.43681700	-0.04613300
H	-0.83436000	-3.50047100	1.04287900
H	-1.57659300	-2.80575400	-0.41949500
H	-0.90078300	-4.45256200	-0.44494100
C	5.82361400	0.53871300	-0.64763600
C	4.18844400	0.42159600	0.96175300
C	5.03503000	1.06836100	1.91497500
C	6.34026500	1.42216200	1.51240500
C	6.74575600	1.15622800	0.22109900
H	6.11107800	0.33214100	-1.67774000
C	4.50933200	1.34071700	3.20704400
H	7.00274000	1.90715400	2.22486500
H	7.73914100	1.41515900	-0.13040400
C	3.21176100	1.02199500	3.48596400
C	2.39909000	0.38248900	2.50183500
H	5.14017500	1.82261800	3.94944000
H	2.78516200	1.25840800	4.45316100
N	4.59529200	0.17608600	-0.30494300
N	2.91296000	0.03993700	1.29856100
C	0.98843800	0.10870100	2.66296900
C	0.25086200	0.46881900	3.80473600
C	-1.12755700	0.31832000	3.83712700
C	-1.78153100	-0.18377600	2.69654400
C	-1.05287900	-0.56389400	1.55296800
C	0.33648600	-0.45614600	1.52283000
H	0.74445900	0.88380800	4.67892900
H	-1.68133900	0.60510100	4.72306900
H	-1.62771800	-0.91597300	0.70017400
Ir	1.57839200	-1.04038300	0.02059700
O	-3.12423700	-0.34854900	2.61320500
C	-3.94806400	0.10797700	3.68133900
H	-3.82213900	1.18421700	3.84999800
H	-4.97454700	-0.08899400	3.36673500
H	-3.74476200	-0.43954200	4.61080300
C	-0.96929100	4.13905200	-3.58809700
C	-0.89296600	3.10233700	-2.67905900
C	0.36295200	2.57934400	-2.27025900
C	1.57485800	3.12517700	-2.81461400
C	1.46325900	4.18612300	-3.74404400
C	0.22229000	4.67545600	-4.11430300
H	-1.93204100	4.53456900	-3.89556900
H	2.37133000	4.61125300	-4.16506200
H	0.17341900	5.49167500	-4.83134900
H	-1.78948400	2.65142200	-2.25259100
C	0.34767100	1.48653500	-1.34390200
H	-0.63242000	1.12210000	-1.01399000
O	1.39917200	0.94489500	-0.90538100
C	-6.12445100	-3.14408500	0.62244900
C	-5.06597000	-2.45417500	0.02218600
C	-4.97728500	-2.35194200	-1.36954200
C	-5.97521000	-2.95688800	-2.14862600
C	-7.03368700	-3.64533600	-1.55532300
C	-7.11255000	-3.74277800	-0.16162500
H	-6.17731200	-3.21519500	1.70746500
H	-4.29005500	-1.98028400	0.61553000
H	-5.91931000	-2.88741000	-3.23500500
H	-7.79733500	-4.10827000	-2.17730200
H	-7.93552900	-4.28015600	0.30427600
C	-3.81739600	-1.59263500	-2.02627800
H	-3.39452300	-2.28036000	-2.79745800
H	-4.27926200	-0.77230200	-2.62542400
O	-2.85691900	-1.12851900	-1.16034300
H	-2.92995600	0.29997200	-1.07000200
C	-5.19997000	4.84029500	1.56229900
C	-5.25555700	3.68190700	0.78540500
C	-4.09448000	2.95396100	0.48692600
C	-2.87110600	3.42184200	0.98216600
C	-2.80967600	4.58334300	1.75626100
C	-3.97323500	5.29757600	2.05163000
H	-6.11270900	5.39137900	1.77780600
H	-6.21347900	3.33819500	0.39825700
H	-1.96898800	2.86808700	0.74439700
H	-1.84861800	4.93312600	2.12735600
H	-3.92518400	6.20376400	2.65080500
C	-4.18120900	1.66938600	-0.32021700
H	-4.46435500	0.84988000	0.36307000
H	-5.02640400	1.77171100	-1.02855500
O	-2.98634100	1.35883700	-0.98217900
N	2.79672100	2.65047400	-2.46112700
H	3.62698500	3.01809700	-2.90190700
H	2.87231600	1.86742500	-1.82414600

100

IN17 SCF Done: E(RM06) = -2295.33599550

C	-0.74249800	-2.89916900	0.39712600
C	-2.04360400	-3.04647800	-0.23843700
C	-3.06308600	-2.71613100	0.76780400
C	-0.96364300	-2.26139600	1.65841500
C	-2.41406200	-2.20598500	1.90195100
C	-2.31166600	-3.78463700	-1.51847700
H	-1.48998200	-3.65504600	-2.22842100
H	-2.42750900	-4.86207100	-1.33322300
H	-3.22809900	-3.42935700	-2.00047600
C	-4.52866700	-2.94839000	0.57249500
H	-4.73874600	-4.02408300	0.65955900
H	-5.13337600	-2.42616300	1.31536300
H	-4.86335300	-2.63573300	-0.42075000
C	-3.03775700	-1.69340200	3.16467900
H	-2.87413200	-2.39718900	3.99243300
H	-2.60248100	-0.73434000	3.46421800
H	-4.11524200	-1.54888100	3.05434900
C	0.09212400	-1.92884300	2.66904200
H	0.29273300	-2.80992700	3.29648800
H	1.03387400	-1.63117900	2.18085200
H	-0.23448200	-1.12685500	3.34030400
C	0.56441400	-3.42149500	-0.11232800
H	0.60776500	-3.41893700	-1.20484700
H	1.41397500	-2.84729800	0.27669500
H	0.67421000	-4.46516000	0.21818900
C	-5.91303700	0.63106600	0.83166400
C	-4.28249000	0.62144600	-0.78458000
C	-5.10825900	1.38417800	-1.66816700
C	-6.39751700	1.74892700	-1.22458000

C -6.80889100 1.37569800 0.03716700
H -6.21024900 0.32645700 1.83412900
C -4.58283600 1.74904700 -2.93635000
H -7.04207200 2.32601400 -1.88278400
H -7.78887900 1.64056400 0.42042300
C -3.30306200 1.40081500 -3.25854900
C -2.50643200 0.65444500 -2.33988000
H -5.19939300 2.31879200 -3.62675200
H -2.87651700 1.69874800 -4.20846600
N -4.70260600 0.25593900 0.44629900
N -3.01625800 0.23848300 -1.15863800
C -1.10896800 0.34298800 -2.54786100
C -0.38058600 0.75349800 -3.67786900
C 0.99095800 0.55702200 -3.75006700
C 1.64761100 -0.04241000 -2.65993500
C 0.92649000 -0.47293000 -1.52883200
C -0.45758500 -0.32080900 -1.46338700
H -0.87566000 1.24313200 -4.51172600
H 1.53782700 0.88275700 -4.62687300
H 1.50346300 -0.90223200 -0.71365100
Ir -1.68763600 -0.94493700 0.03218800
O 2.98497400 -0.25797700 -2.61583900
C 3.80260200 0.22834100 -3.67536500
H 3.71667700 1.31672400 -3.77853600
H 4.82703100 -0.02639500 -3.39773800
H 3.55690800 -0.25544000 -4.62958400
C 1.17856700 4.07902600 3.65398000
C 1.07485300 2.99427100 2.78336000
C -0.19676800 2.58414500 2.33985400
C -1.35531100 3.26279700 2.76936500
C -1.23846100 4.34187500 3.63496000
C 0.02887600 4.74912000 4.07754000
H 2.15611200 4.40263700 3.99973800
H -2.32735200 2.93057700 2.41797500
H -2.12639800 4.87018400 3.97109900
H 0.11451300 5.59391400 4.75632600
H 1.95594200 2.45887200 2.42802700
C -0.27882900 1.43074400 1.45132400
H 0.66342700 0.94605400 1.16657200
O -1.36797000 1.00022200 1.02754100
C 5.93961200 -3.20655600 -0.95455500
C 4.91408800 -2.55226900 -0.26390900
C 4.85607500 -2.59003200 1.13274700
C 5.84896300 -3.30126100 1.82348200
C 6.87401100 -3.95560100 1.13955000
C 6.92390700 -3.91048800 -0.25811100
H 5.96936200 -3.16744100 -2.04207500
H 4.14162400 -1.99940000 -0.78939600
H 5.81658700 -3.34205400 2.91225900
H 7.63449500 -4.50193400 1.69401100
H 7.72124100 -4.41976700 -0.79474800
C 3.73647800 -1.86371300 1.88916200
H 3.29959800 -2.61167800 2.59279100
H 4.23834800 -1.12668000 2.55959100
O 2.77930200 -1.27723600 1.09547800
H 2.95248800 0.13779400 1.11021600
C 5.35075600 4.79697200 -1.17685400
C 5.38245900 3.58553800 -0.48329400
C 4.21052600 2.84858800 -0.26097100
C 3.00074300 3.36103000 -0.74618600
C 2.96298400 4.57450700 -1.43706700
C 4.13787800 5.29781500 -1.65727700
H 6.27139700 5.35453000 -1.33404800
H 6.32961900 3.20728000 -0.10215200
H 2.09040900 2.79923000 -0.56478200
H 2.01242400 4.95784900 -1.80192200
H 4.10863300 6.24461900 -2.19128000
C 4.26563800 1.50920600 0.45522400
H 4.50597100 0.72901700 -0.28782500
H 5.12614300 1.53221800 1.15182500
O 3.07138800 1.19746300 1.11763600

16
1a-4 SCF Done: E(RM06) = -400.767869235
H -0.70556100 2.98650300 0.18566300
C -1.76681400 0.68457300 -0.01979800
C -0.38824400 0.98502300 -0.00199700
C 0.53467800 -0.09692900 0.00350600
C 0.05185200 -1.42045800 0.02392700
C -1.30459500 -1.69959600 0.01552000
C -2.21280000 -0.62931400 -0.01405500
H -2.48223900 1.50376100 -0.03185600
H 0.78949100 -2.21728200 0.03585600
H -1.66061100 -2.72522800 0.02615500
H -3.28218700 -0.82492500 -0.02454600
C 1.98837500 0.11250900 -0.04979100
H 2.32784700 1.16148200 -0.18150500
O 2.82422100 -0.77786900 0.01571800
N 0.01515600 2.31135300 -0.03899000
H 0.89868900 2.54432600 0.39354500

4
NH3 SCF Done: E(RM06) = -56.5395147280
N 0.00000000 0.00000000 0.12054200
H 0.00000000 0.93751400 -0.28126500
H -0.81191100 -0.46875700 -0.28126500
H 0.81191100 -0.46875700 -0.28126500

73
TS14 SCF Done: E(RM06) = -1659.07548630
C 1.41923100 -0.92533400 2.45367400
C 0.57348700 -2.05527500 2.15298200
C -0.80409400 -1.69633100 2.51748000
C 0.55288700 0.18810200 2.75635000
C -0.81906600 -0.33531200 2.84769300
C 1.04339700 -3.45219100 1.86840300
H 1.99829000 -3.45270700 1.33528900
H 1.18209500 -4.01216000 2.80374300
H 0.31885600 -4.00218700 1.26012600
C -1.94231400 -2.66456400 2.58760300
H -1.88797200 -3.22264200 3.53347000
H -2.91091400 -2.16086500 2.55416300
H -1.91128600 -3.39349900 1.77495700
C -2.00401800 0.49789700 3.22677300
H -1.98781500 0.72467600 4.30148000
H -2.00492500 1.45416700 2.69287600

H	-2.94586900	-0.00927700	3.00334700
C	0.98155500	1.53264600	3.26987700
H	1.12315700	1.50625100	4.35938300
H	1.92879700	1.85414300	2.82580800
H	0.22985200	2.30059200	3.06073000
C	2.91367700	-0.96282700	2.55844800
H	3.36355100	-1.60547000	1.79699800
H	3.36253300	0.02872100	2.47214300
H	3.19069800	-1.36555700	3.54238900
C	-3.70379300	-2.42047900	-0.62787700
C	-1.56836400	-2.02854100	-1.36702300
C	-1.82727300	-2.71595100	-2.59322000
C	-3.11145900	-3.27533200	-2.78185000
C	-4.06223100	-3.12645300	-1.79652500
H	-4.43716400	-2.28698700	0.16557000
C	-0.79071700	-2.78758000	-3.55946200
H	-3.32951700	-3.80759500	-3.70406200
H	-5.06193900	-3.53469200	-1.90157100
C	0.40382700	-2.17130600	-3.31024200
C	0.61142500	-1.48673400	-2.07929400
H	-0.96432000	-3.31656700	-4.49283600
H	1.19882800	-2.19493900	-4.04555300
N	-2.50879400	-1.89506400	-0.40756800
N	-0.33930300	-1.46318400	-1.12359800
C	1.80929100	-0.72607400	-1.76562700
C	2.89262300	-0.57168400	-2.64572200
C	3.96494700	0.25538600	-2.33144700
C	3.94423900	0.95909900	-1.11476800
C	2.86146400	0.80423500	-0.22660500
C	1.79480600	-0.05591100	-0.50684300
H	2.91034900	-1.09331400	-3.59855100
H	4.79120000	0.35303400	-3.02495600
H	2.90267800	1.36630800	0.70170800
Ir	0.21939700	-0.45697700	0.69459100
O	4.91607700	1.81098700	-0.70719300
C	6.06039100	2.00103200	-1.53915800
H	5.78409700	2.43051100	-2.51007200
H	6.70153700	2.70422600	-1.00446900
H	6.60345500	1.06123800	-1.69335600
N	0.54153200	2.90673200	-1.49780800
H	0.09707000	2.66156000	-2.38351900
H	0.79066400	3.89703400	-1.51784900
H	1.38521000	2.33880900	-1.38277600
C	-2.75106700	5.56321700	-0.09876100
C	-1.70645700	4.66546000	0.12195800
C	-1.79677300	3.34783800	-0.34711400
C	-2.94570500	2.93295300	-1.03290700
C	-3.98822500	3.83239700	-1.25411600
C	-3.89223900	5.14756600	-0.78997300
H	-2.67835500	6.58188000	0.27179500
H	-3.01552500	1.90501800	-1.37451300
H	-4.87930300	3.50722500	-1.78415600
H	-4.70754400	5.84520800	-0.96054200
C	-0.66843100	2.39136600	-0.09716500
H	0.02337000	2.71479300	0.69092600
O	-0.88620100	1.14113600	-0.25396300
H	-0.82052100	4.98599400	0.66720000

73

IN18 SCF Done: E(RM06) = -1659.07752042

C	1.29494200	-0.80364200	2.53848300
C	0.45769600	-1.94666800	2.27374600
C	-0.93073000	-1.55721100	2.54974100
C	0.42481700	0.33106200	2.73146300
C	-0.95374600	-0.17894800	2.79567400
C	0.92829300	-3.36285100	2.10663000
H	1.92389400	-3.40464000	1.65558600
H	0.97992200	-3.87199300	3.07923300
H	0.24849900	-3.93870000	1.47076600
C	-2.07311200	-2.52003100	2.63010300
H	-2.05294300	-3.02594200	3.60622100
H	-3.03982600	-2.02171500	2.53311500
H	-2.01129500	-3.29283300	1.86068500
C	-2.15036100	0.67742900	3.07441700
H	-2.15854200	1.00024700	4.12433600
H	-2.14821800	1.58080900	2.45545300
H	-3.08447000	0.14511800	2.87891600
C	0.84570900	1.69849200	3.18893900
H	0.97749500	1.72000200	4.27980800
H	1.79580100	2.00432000	2.73972100
H	0.09384300	2.45411000	2.94034300
C	2.78085400	-0.83847200	2.73051300
H	3.27374700	-1.51522500	2.02769500
H	3.23707300	0.14853700	2.62936800
H	2.99796300	-1.19588700	3.74652700
C	-3.75894800	-2.36447000	-0.61264300
C	-1.60368300	-2.04849100	-1.33095600
C	-1.86343400	-2.76487200	-2.54048200
C	-3.15874700	-3.29744500	-2.73130500
C	-4.11891700	-3.09646200	-1.76468200
H	-4.49941300	-2.18900600	0.16588000
C	-0.81686200	-2.89099900	-3.48996500
H	-3.37746700	-3.85089400	-3.64080600
H	-5.12685300	-3.48331300	-1.87202200
C	0.39129600	-2.30299400	-3.23862700
C	0.60087000	-1.59275600	-2.02265200
H	-0.99286700	-3.43914200	-4.41175800
H	1.19581500	-2.36786200	-3.96096500
N	-2.55439400	-1.86274300	-0.39094200
N	-0.36347200	-1.50856000	-1.08477800
C	1.82058200	-0.86758200	-1.70882700
C	2.92756700	-0.79142100	-2.56888200
C	4.02925600	-0.00090500	-2.26184100
C	4.01482400	0.74571000	-1.07149300
C	2.90741600	0.67077200	-0.20242600
C	1.80764500	-0.15470800	-0.47205000
H	2.94217900	-1.34803800	-3.50180200
H	4.87302400	0.03482600	-2.94014600
H	2.96189600	1.25835900	0.70916700
Ir	0.18201100	-0.43108300	0.69432500
O	5.01534500	1.56770000	-0.67266600
C	6.19126300	1.66131500	-1.47706800
H	5.96543500	2.06186100	-2.47305500
H	6.85410100	2.35231100	-0.95308800
H	6.68649800	0.68805100	-1.57535800
N	0.61627300	2.72276600	-1.49732300

H 0.18866800 2.45044000 -2.38673200
H 0.90029000 3.70531100 -1.55360100
H 1.44151000 2.12961800 -1.33554700
C -2.45264700 5.67839100 -0.35581400
C -1.43625200 4.74864300 -0.13167800
C -1.60533900 3.41061600 -0.51162200
C -2.80487000 3.00789000 -1.10914200
C -3.82090700 3.93836100 -1.33223900
C -3.64619300 5.27353400 -0.95912100
H -2.31794300 6.71288800 -0.05223500
H -2.93498500 1.96415700 -1.37621800
H -4.75277800 3.62031600 -1.79193900
H -4.44074800 5.99486600 -1.12912000
C -0.49210600 2.40661900 -0.27301100
H 0.12802100 2.69721500 0.58840100
O -0.83208700 1.14277100 -0.36011000
H -0.51417000 5.06383700 0.35459000

75
TS14' SCF Done: E(RM06) = -1714.41743282
C 1.55945800 -0.18579000 2.54091400
C 1.15700800 -1.55815500 2.37444600
C -0.28263600 -1.63966900 2.64812400
C 0.35581500 0.60345900 2.67832500
C -0.76801100 -0.33449800 2.80310600
C 2.07843200 -2.73985300 2.27868700
H 3.02135100 -2.47296100 1.79281100
H 2.31519900 -3.12968500 3.27847600
H 1.62672800 -3.55631700 1.70661100
C -1.04467200 -2.91682200 2.81682800
H -0.91018600 -3.29125600 3.84204000
H -2.11578200 -2.78054700 2.65171900
H -0.69430700 -3.69584700 2.13635300
C -2.18450000 0.08022500 3.05700600
H -2.32063400 0.35216300 4.11288800
H -2.46152700 0.95467100 2.45963800
H -2.89054300 -0.72028400 2.82248000
C 0.29524900 2.06001600 3.04100200
H 0.37404600 2.19292400 4.12929000
H 1.11308300 2.62618600 2.58427800
H -0.64718400 2.51975400 2.72647800
C 2.97075800 0.29793700 2.68302800
H 3.66073000 -0.25476500 2.03985200
H 3.07222800 1.36067100 2.45287100
H 3.29479300 0.15392100 3.72293400
C -2.49030600 -3.70466000 -0.49026600
C -0.56851800 -2.66794900 -1.19352300
C -0.47901300 -3.52768200 -2.33207700
C -1.47306100 -4.51902300 -2.49566100
C -2.49116000 -4.61092200 -1.57275300
H -3.28523300 -3.75296900 0.25226100
C 0.58887500 -3.33084200 -3.24507200
H -1.42176500 -5.18732500 -3.35131900
H -3.27779900 -5.35287300 -1.66235800
C 1.47186300 -2.30873800 -3.03469800
C 1.33323500 -1.46821600 -1.89345700
H 0.68119100 -3.98289600 -4.10970100
H 2.27745800 -2.12615700 -3.73557600
N -1.56994200 -2.77368900 -0.29427700
N 0.36743700 -1.68444400 -0.97804900
C 2.15919500 -0.29842500 -1.64168800
C 3.19298900 0.12901200 -2.48948600
C 3.88369900 1.31189700 -2.24956800
C 3.51809500 2.09589800 -1.14284600
C 2.48434900 1.66921100 -0.28481600
C 1.80853800 0.45826100 -0.48334700
H 3.47263700 -0.45668900 -3.36049000
H 4.68208300 1.61428000 -2.91621200
H 2.26073500 2.30557800 0.56702600
Ir 0.38856500 -0.34512400 0.70528900
O 4.09803900 3.27692100 -0.81733600
C 5.17730300 3.75732800 -1.61876100
H 4.85962500 3.93889100 -2.65295600
H 5.48492200 4.70142200 -1.16557100
H 6.02299100 3.05956000 -1.61036200
N -0.42541100 2.65034300 -1.54986400
H -0.68202000 2.33728900 -2.49025600
H -0.46876100 3.67126000 -1.50404600
H 0.54120600 2.35192900 -1.35733400
C -4.71776900 3.76453200 -0.67546300
C -3.34775100 3.53016400 -0.49591600
C -2.82826900 2.24674100 -0.76533500
C -3.68211400 1.24746200 -1.24893400
C -5.03877000 1.49770500 -1.44752200
C -5.56022100 2.75718500 -1.14349600
H -5.11835900 4.75116600 -0.45255200
H -5.68934000 0.70964700 -1.81609000
H -6.61955800 2.95955800 -1.27645400
C -1.39088200 1.88707300 -0.42426300
H -1.05847800 2.43309300 0.46848900
O -1.11335200 0.60072300 -0.48565000
H -3.26720700 0.26064400 -1.42410000
N -2.47337700 4.55655000 -0.00596600
H -2.03804100 5.06747200 -0.77530800
H -3.00549900 5.25343100 0.51283700

75
IN18' SCF Done: E(RM06) = -1714.42821239
C 1.75865400 -0.56590700 2.41150300
C 1.34759800 -1.90649600 2.08791000
C -0.06450900 -2.04550600 2.46157000
C 0.56870700 0.17909400 2.75844700
C -0.53844500 -0.78385000 2.84411900
C 2.25879800 -3.05076500 1.75135400
H 3.16370100 -2.70573300 1.24320800
H 2.56876000 -3.58101900 2.66274000
H 1.76483500 -3.77894800 1.10047500
C -0.80560200 -3.34540500 2.49918600
H -0.56871500 -3.87618700 3.43269000
H -1.88749300 -3.20113600 2.46312700
H -0.52492700 -3.99947100 1.67082500
C -1.93080200 -0.42192100 3.25996500
H -1.96527600 -0.18868600 4.33277600
H -2.29009300 0.46116200 2.72120600
H -2.63517500 -1.23551200 3.06997700
C 0.52769600 1.56972800 3.32535800

H	0.65124200	1.55010600	4.41736600
H	1.32634900	2.19913900	2.91965900
H	-0.42897400	2.06022500	3.11732900
C	3.17719800	-0.09118700	2.50228800
H	3.80470300	-0.51588900	1.71416900
H	3.25735500	0.99642100	2.44885100
H	3.59665400	-0.40371100	3.46845600
C	-2.59961700	-3.62966900	-0.71057700
C	-0.74273000	-2.49516200	-1.43646400
C	-0.77316000	-3.17795900	-2.69224400
C	-1.79139700	-4.13504200	-2.90387300
C	-2.71789900	-4.36330100	-1.91100700
H	-3.31856400	-3.79161800	0.09086100
C	0.20644900	-2.84956300	-3.66414700
H	-1.83013000	-4.66938800	-3.84953200
H	-3.51974900	-5.08398300	-2.03311100
C	1.11859100	-1.86834400	-3.39244400
C	1.09877900	-1.20262100	-2.13395900
H	0.20920100	-3.36873700	-4.61884600
H	1.85767100	-1.58729800	-4.13263900
N	-1.65257700	-2.73714400	-0.46848800
N	0.21939400	-1.54978700	-1.17215000
C	1.96189700	-0.08211200	-1.79583400
C	2.91146100	0.47028600	-2.67078800
C	3.63762600	1.60573900	-2.32814000
C	3.39415800	2.21452900	-1.08515700
C	2.44052700	1.66652000	-0.20438000
C	1.73382100	0.49629900	-0.51070800
H	3.09550900	0.02173800	-3.64291200
H	4.36898500	2.00722200	-3.01907400
H	2.30410300	2.17302200	0.74660900
Ir	0.42444900	-0.47160400	0.67958700
O	4.02259300	3.33050600	-0.64276900
C	5.02952200	3.92493800	-1.46145000
H	4.61715600	4.26905500	-2.41802900
H	5.39910500	4.78421500	-0.89879200
H	5.85732200	3.22983000	-1.64589700
N	-0.55424600	2.64055700	-1.29527800
H	-0.86704500	2.22928100	-2.17891300
H	-0.75966900	3.65728000	-1.27719700
H	0.45178300	2.45835600	-1.18431600
C	-4.43027700	4.18339300	-0.45804000
C	-3.10493500	3.78029700	-0.25869600
C	-2.79317600	2.40348500	-0.24123100
C	-3.81873900	1.46653500	-0.39019000
C	-5.14003400	1.87394900	-0.57955900
C	-5.44107200	3.23606600	-0.62291400
H	-4.66570900	5.24534800	-0.47715700
H	-5.92653900	1.13414700	-0.69702700
H	-6.46494900	3.56711500	-0.77359500
C	-1.35281500	1.92511400	-0.08532100
H	-0.86304800	2.40820800	0.77896600
O	-1.20331500	0.61212200	-0.16830600
H	-3.55368200	0.41510100	-0.36106000
N	-2.05227100	4.73890300	-0.16330600
H	-2.40480500	5.69047600	-0.23757000
H	-1.53640300	4.66769000	0.71382400

77

IN19 SCF Done: E(RM06) = -1715.63825136

C	-1.70138500	0.68806100	2.46481400
C	-1.14638500	1.97890700	2.15326400
C	0.27116800	1.96063900	2.53540800
C	-0.60030700	-0.18466700	2.80477400
C	0.60396600	0.65268600	2.90838100
C	-1.92597200	3.22110900	1.83038100
H	-2.86624500	2.98552700	1.32357600
H	-2.17045800	3.77660300	2.74669400
H	-1.35555400	3.89319000	1.18149300
C	1.15033300	3.17161500	2.58003900
H	0.97671400	3.71724900	3.51882600
H	2.20980400	2.91055500	2.53632300
H	0.93861900	3.86007600	1.75874800
C	1.94881800	0.13782400	3.31919000
H	1.93852900	-0.17762100	4.37102200
H	2.24164600	-0.72946600	2.71786100
H	2.72486300	0.89872100	3.20402200
C	-0.71053000	-1.57973900	3.35178500
H	-0.88133800	-1.56073900	4.43750700
H	-1.54203900	-2.13159700	2.90144500
H	0.20661300	-2.15177600	3.17889400
C	-3.16263400	0.36835300	2.55769700
H	-3.74496900	0.85925700	1.77353600
H	-3.35864200	-0.70448500	2.50330800
H	-3.54262000	0.72112700	3.52641800
C	3.02971400	3.21656700	-0.60683300
C	1.04723300	2.33967300	-1.35749700
C	1.17543700	3.02564300	-2.60546700
C	2.31005000	3.84596700	-2.79918900
C	3.25002000	3.94323100	-1.79721600
H	3.75775100	3.27425600	0.20077300
C	0.16997700	2.83246000	-3.58825700
H	2.42445000	4.38141300	-3.73818500
H	4.13792200	4.55734600	-1.90499300
C	-0.86466800	1.97655900	-3.33108100
C	-0.94624400	1.30831000	-2.07526100
H	0.24316000	3.35320200	-4.53939800
H	-1.62426200	1.79542900	-4.08200800
N	1.97427300	2.45048800	-0.38171700
N	-0.03140700	1.52598000	-1.10796200
C	-1.95356900	0.31357800	-1.74733200
C	-2.98175600	-0.08353900	-2.61817800
C	-3.84560000	-1.12239900	-2.29019900
C	-3.66321200	-1.79360600	-1.06821500
C	-2.64282900	-1.39169800	-0.18559600
C	-1.79528600	-0.31515300	-0.47386000
H	-3.12352600	0.41659100	-3.57202100
H	-4.63493600	-1.40499700	-2.97647600
H	-2.55782000	-1.93622200	0.74983900
Ir	-0.38179400	0.46577100	0.73396200
O	-4.41905800	-2.83829900	-0.64977700
C	-5.50331200	-3.27404300	-1.46823600
H	-5.15202500	-3.63293400	-2.44376000
H	-5.96939500	-4.09986700	-0.92770600
H	-6.24049000	-2.47537500	-1.61387200
N	0.30174500	-2.67367600	-1.30521500

H 0.84227900 -2.38037900 -2.17711200
H 0.19575700 -3.69283600 -1.31055400
H -0.63313100 -2.24784400 -1.31627700
C 3.72458200 -4.85109700 0.37416600
C 2.48920600 -4.20057000 0.37123300
C 2.40032100 -2.84636600 0.02484800
C 3.56570500 -2.14395000 -0.30235000
C 4.80179600 -2.79400200 -0.29806600
C 4.88359100 -4.14845000 0.03579700
H 3.78340000 -5.90077400 0.64901500
H 3.48866300 -1.08710300 -0.53611400
H 5.70387400 -2.24080400 -0.54677200
H 5.84650500 -4.65175500 0.04300100
C 1.04484000 -2.14533400 0.00205700
H 0.40263200 -2.55186100 0.80045600
O 1.11761000 -0.81563600 -0.05806100
H 1.59197100 -4.75072600 0.65208000
H 2.82481300 -2.08313600 -3.28363800
N 1.85503300 -1.95979100 -3.57614000
H 1.73516600 -2.50313600 -4.43112300
H 1.75615700 -0.97846800 -3.83592600

79
IN19' SCF Done: E(RM06) = -1770.98835794
C 1.09729400 1.42282200 2.52302500
C 2.25477500 0.62689300 2.21072700
C 1.94541300 -0.76972000 2.54283300
C 0.01224100 0.50883800 2.80930800
C 0.58824500 -0.84217400 2.87875400
C 3.63329900 1.15047400 1.92840200
H 3.59702300 2.12215500 1.42766900
H 4.20177700 1.27609900 2.86069800
H 4.19939800 0.46567800 1.28923800
C 2.95382100 -1.87463100 2.58208700
H 3.50332200 -1.83796000 3.53390000
H 2.48552100 -2.85867100 2.50638400
H 3.68721900 -1.78730400 1.77740700
C -0.20058500 -2.06524800 3.23123600
H -0.52244800 -2.03029200 4.28070200
H -1.10132900 -2.14414400 2.61328200
H 0.38229500 -2.97899400 3.08888200
C -1.34052200 0.87609800 3.34947300
H -1.31312500 0.96822600 4.44451100
H -1.69131800 1.83373800 2.95202500
H -2.08864900 0.11394700 3.10758400
C 1.07693200 2.91585700 2.65802600
H 1.70985000 3.40494700 1.91232000
H 0.06948600 3.32666900 2.56520700
H 1.45541000 3.19195500 3.65185400
C 2.85834000 -3.48710700 -0.72206300
C 2.30195900 -1.36388400 -1.39272700
C 2.98255100 -1.54217100 -2.63768900
C 3.62570300 -2.77825100 -2.87292800
C 3.56343200 -3.76415800 -1.91308900
H 2.79338600 -4.24833600 0.05373200
C 2.96502800 -0.47788500 -3.57584100
H 4.15368700 -2.93281900 -3.81040000
H 4.03888700 -4.72926900 -2.05390700
C 2.27318900 0.66358600 -3.28145800
C 1.59872500 0.79138800 -2.03327700
H 3.48846200 -0.58769500 -4.52191400
H 2.23205800 1.47830800 -3.99439000
N 2.25552900 -2.33839400 -0.45907300
N 1.65757900 -0.18396400 -1.10312700
C 0.76803600 1.92715400 -1.67232600
C 0.52799900 3.02467000 -2.51610400
C -0.36031600 4.03131500 -2.15559200
C -1.03946800 3.92654900 -0.92881300
C -0.79796300 2.83320800 -0.07646800
C 0.12528800 1.83177800 -0.39985500
H 1.03031400 3.10620300 -3.47590600
H -0.52462900 4.87056000 -2.82055200
H -1.34531400 2.81086700 0.86092300
Ir 0.64152700 0.25273900 0.73761300
O -1.94980600 4.82622700 -0.48062000
C -2.23541800 5.97277700 -1.28024300
H -2.66165800 5.68930800 -2.25069300
H -2.97254500 6.54972500 -0.71871400
H -1.33971600 6.58559900 -1.43673400
N -2.54585100 0.21478500 -1.44078900
H -2.31316200 -0.39183700 -2.28418200
H -3.55380400 0.44056300 -1.43483500
H -1.99549600 1.07877900 -1.46972100
C -5.41075800 -2.63329200 -0.04776200
C -4.54722500 -1.53045000 -0.04315400
C -3.15096600 -1.74355900 -0.06226700
C -2.65872700 -3.05083900 -0.04978300
C -3.52398100 -4.14695300 -0.04589400
C -4.90309200 -3.93252300 -0.05544300
H -6.48550800 -2.46499700 -0.03786700
H -1.58205100 -3.18446800 -0.04879500
H -3.12535200 -5.15740800 -0.03781200
H -5.58988200 -4.77474600 -0.05459400
C -2.17892700 -0.56446000 -0.11992900
H -2.42633100 0.17416200 0.66189100
O -0.89039000 -0.92107200 -0.15077300
H -1.54439200 -2.34982200 -3.11821000
N -1.95522000 -1.54643800 -3.59421900
H -2.76965500 -1.89496900 -4.09993500
H -1.27921000 -1.24813400 -4.29702800
N -5.05798400 -0.20454300 -0.10598900
H -6.07427300 -0.18634500 -0.07480200
H -4.70854000 0.39184800 0.64306500

77
TS15 SCF Done: E(RM06) = -1715.63381546
C 0.27872100 1.05393200 2.73551000
C 1.54471300 0.38834200 2.57165100
C 1.30337500 -1.05982400 2.63538600
C -0.75166300 0.04331400 2.65829800
C -0.08168200 -1.26553200 2.66442900
C 2.89397900 1.03923200 2.67216600
H 2.87344700 2.06039600 2.28105500
H 3.22459900 1.08837800 3.71919000
H 3.65375600 0.48168600 2.11608100
C 2.37895400 -2.09679000 2.73566300

H 2.72082400 -2.17285600 3.77796200
H 2.02627800 -3.08471900 2.43032000
H 3.25047800 -1.84549900 2.12674100
C -0.80917500 -2.57397600 2.68342000
H -1.29489700 -2.73070200 3.65605800
H -1.59097200 -2.60245200 1.91799600
H -0.13519000 -3.41590900 2.50559300
C -2.21272600 0.25148200 2.93656600
H -2.41425500 0.19287300 4.01566300
H -2.55363800 1.23377500 2.59511700
H -2.82978300 -0.50898200 2.44851500
C 0.08785600 2.50393000 3.06219500
H 0.83360700 3.13869500 2.57595200
H -0.90203100 2.86703300 2.77763300
H 0.18936300 2.64112200 4.14775000
C 3.14233000 -3.18460600 -0.74291000
C 2.60141100 -0.98682400 -1.12752300
C 3.64493200 -0.86245400 -2.09678000
C 4.46529900 -1.98787100 -2.33679400
C 4.21775500 -3.16060400 -1.65659200
H 2.91381800 -4.10242600 -0.20388200
C 3.78494800 0.37435800 -2.78120700
H 5.27344300 -1.91282000 -3.05990900
H 4.82001800 -4.04922400 -1.81362100
C 2.90288500 1.38742800 -2.52608800
C 1.87645100 1.21936700 -1.54978900
H 4.58167100 0.49628200 -3.51023600
H 2.98666300 2.33049600 -3.05250100
N 2.36290000 -2.14679500 -0.47739600
N 1.77091200 0.07236500 -0.84065400
C 0.85649300 2.20885000 -1.26075000
C 0.71347600 3.41766400 -1.96475300
C -0.34491000 4.27897700 -1.70725400
C -1.29193800 3.91712700 -0.73136700
C -1.15325400 2.70944000 -0.02243900
C -0.07628100 1.84688200 -0.24150700
H 1.42467700 3.69854700 -2.73641000
H -0.43343300 5.20543900 -2.26141200
H -1.91327200 2.48269000 0.71932800
Ir 0.31681000 0.13522000 0.75574900
O -2.37190300 4.66578000 -0.40359200
C -2.57071000 5.91709000 -1.06047300
H -2.71128200 5.78562800 -2.14046500
H -3.48067000 6.33655700 -0.62777800
H -1.73450100 6.60276400 -0.87838100
N -2.42694800 0.19638000 -2.06076700
H -1.18474700 -0.70408100 -3.23202400
H -3.40570700 0.24877200 -2.34043500
H -2.11965200 1.15105800 -1.86954800
C -5.42125600 -2.78429500 -1.10387000
C -4.58311400 -1.67390100 -1.00150100
C -3.19217900 -1.83004000 -0.89830500
C -2.66129200 -3.12441800 -0.88109700
C -3.49936100 -4.23911600 -0.98395400
C -4.87993300 -4.07299900 -1.09976900
H -6.49628600 -2.64441200 -1.18057400
H -1.58951800 -3.24868000 -0.76653000
H -3.07197300 -5.23860400 -0.96704700
H -5.53170600 -4.93905100 -1.17640200
C -2.28966900 -0.59538300 -0.79877900
H -2.62843800 0.01016700 0.05368100
O -0.94772000 -0.94594700 -0.60451000
H -5.01942300 -0.67616200 -0.99171900
H -0.39519600 -1.29039900 -1.94475200
N -0.34441800 -1.30687500 -3.05732300
H -0.46252200 -2.24079600 -3.45460300
H 0.50530200 -0.87340600 -3.42199800

77

IN20 SCF Done: E(RM06) = -1715.64321931

C 0.22472500 1.06196500 2.74923100
C 1.55292100 0.52447100 2.54841500
C 1.45723600 -0.94216100 2.61899200
C -0.69485400 -0.04134900 2.67589500
C 0.10003500 -1.28100900 2.65878200
C 2.83356400 1.30123700 2.64726900
H 2.70859300 2.32730100 2.29041000
H 3.17503200 1.34842300 3.69070200
H 3.63146300 0.83567300 2.06113300
C 2.63026700 -1.86696800 2.70515800
H 2.98976200 -1.90554000 3.74351500
H 2.37284500 -2.88509800 2.40473500
H 3.46401100 -1.53024400 2.08507600
C -0.49637000 -2.65364100 2.68104800
H -0.90518800 -2.87664800 3.67596800
H -1.31914800 -2.74196800 1.96433300
H 0.24465900 -3.42092800 2.44296100
C -2.17254700 0.01818500 2.93515900
H -2.38081200 -0.08904500 4.00898500
H -2.60190200 0.97216600 2.61341800
H -2.70506500 -0.78546400 2.41849200
C -0.09492000 2.48451500 3.09050700
H 0.54781500 3.19160300 2.56009500
H -1.13368900 2.74089900 2.87351700
H 0.06275200 2.63252100 4.16785400
C 3.33983600 -2.99015000 -0.75974200
C 2.66050100 -0.83459200 -1.15662500
C 3.65836100 -0.66650600 -2.16540400
C 4.53188000 -1.74793700 -2.42043400
C 4.37585000 -2.92169900 -1.71555900
H 3.18483700 -3.91010700 -0.19873700
C 3.70363800 0.56527300 -2.87068500
H 5.30648900 -1.63919700 -3.17526400
H 5.02052000 -3.77787300 -1.88366800
C 2.77563500 1.53073300 -2.59764600
C 1.79611600 1.31852600 -1.58467100
H 4.46361800 0.71822700 -3.63242400
H 2.78128700 2.46601400 -3.14404300
N 2.51411800 -1.99318400 -0.47922600
N 1.78078900 0.17953600 -0.85755500
C 0.72907800 2.25225600 -1.27715200
C 0.48700900 3.43523100 -1.99535400
C -0.62720200 4.22220600 -1.72989400
C -1.52677700 3.81081000 -0.73056500
C -1.28158700 2.63232100 0.00056500
C -0.15214100 1.84753200 -0.23286800

H 1.15986200 3.75303700 -2.78635600
H -0.79398700 5.12887900 -2.29895300
H -2.00545900 2.36739100 0.76523300
Ir 0.36627700 0.16503400 0.76759400
O -2.65567800 4.48030300 -0.39671100
C -2.96993300 5.68853300 -1.08827200
H -3.11412000 5.50958600 -2.16083500
H -3.90605300 6.04322100 -0.65340700
H -2.19239000 6.44769100 -0.94185900
N -2.40935700 0.05036300 -2.14246500
H -0.84523800 -1.31121500 -3.48848900
H -3.33286500 -0.05387800 -2.55431100
H -2.22960000 1.04298300 -2.00261900
C -5.43176600 -2.86172800 -1.02476800
C -4.58880800 -1.75078700 -1.00763800
C -3.19900300 -1.90662800 -0.89021900
C -2.67194400 -3.19759700 -0.77593600
C -3.51671200 -4.31175200 -0.79374700
C -4.89639000 -4.14839000 -0.92027100
H -6.50567500 -2.72279400 -1.11511000
H -1.60101100 -3.32989700 -0.66696200
H -3.09272500 -5.30868300 -0.70575800
H -5.55165300 -5.01501700 -0.93184700
C -2.31674900 -0.66045500 -0.87581300
H -2.60146200 -0.03393400 -0.02293600
O -0.93962600 -0.99331600 -0.61483900
H -5.01635900 -0.75264200 -1.08095100
H -0.51235900 -1.34145900 -1.48570600
N -0.15489400 -1.89116200 -3.00782800
H -0.39001000 -2.86574100 -3.19342900
H 0.75405200 -1.71328400 -3.43212100

79
TS15' SCF Done: E(RM06) = -1770.98129215
C 0.51892100 1.02458300 2.74133000
C 1.73903700 0.28390000 2.54066400
C 1.41445100 -1.14827100 2.62128300
C -0.56972200 0.07581800 2.68611100
C 0.02214400 -1.27136500 2.68533800
C 3.12767600 0.85083500 2.60930700
H 3.16108300 1.87300700 2.22175600
H 3.48539800 0.87482700 3.64824900
H 3.83817700 0.24972500 2.03373500
C 2.42872700 -2.24572200 2.70390600
H 2.77764900 -2.34642800 3.74183700
H 2.01592200 -3.21010000 2.39892100
H 3.30534900 -2.04123500 2.08514900
C -0.77516000 -2.53693500 2.75912000
H -1.15146200 -2.69004600 3.78018800
H -1.64444000 -2.50707900 2.09528300
H -0.17725700 -3.41180000 2.48981500
C -2.00882200 0.36451700 3.00452800
H -2.18610000 0.30746500 4.08788800
H -2.30246800 1.36769100 2.67965900
H -2.67847400 -0.35776500 2.52756900
C 0.42725700 2.48169400 3.07961700
H 1.18533200 3.07371200 2.55997200
H -0.55138100 2.90375900 2.84187000
H 0.58763400 2.60694700 4.15945900
C 3.06959600 -3.34031600 -0.77572700
C 2.62275800 -1.12525200 -1.18119600
C 3.60402400 -1.08090800 -2.21979400
C 4.34254100 -2.25603700 -2.48611500
C 4.07851300 -3.39756500 -1.76078700
H 2.83032700 -4.23105300 -0.19744200
C 3.76638400 0.13088900 -2.94218600
H 5.10158300 -2.24289100 -3.26411800
H 4.61861400 -4.32221300 -1.93531900
C 2.97015100 1.20198000 -2.64635200
C 2.00724200 1.11600100 -1.59778100
H 4.51181100 0.18893000 -3.73099900
H 3.06816700 2.12495800 -3.20444600
N 2.36737000 -2.25502100 -0.48630100
N 1.86955800 -0.01562200 -0.87124000
C 1.07577500 2.17544300 -1.25945800
C 1.00046400 3.40309500 -1.94109100
C 0.00910900 4.32964800 -1.64625300
C -0.93990200 4.01603600 -0.65614400
C -0.85781000 2.79741400 0.04251500
C 0.15402100 1.86968000 -0.21281700
H 1.71567000 3.65160300 -2.72001700
H -0.02746500 5.26977100 -2.18290900
H -1.60773500 2.61527300 0.80611400
Ir 0.45923700 0.12723000 0.75821200
O -1.96779200 4.82425000 -0.30215400
C -2.11495900 6.08178500 -0.96143000
H -2.27915700 5.95285300 -2.03838000
H -2.99570200 6.54788400 -0.51605900
H -1.24259100 6.72534200 -0.79587600
N -2.44042100 0.35647700 -1.99133900
H -1.23317000 -0.66108200 -3.29823700
H -3.43933800 0.44927500 -2.18300300
H -2.07901500 1.29743600 -1.83001600
C -5.41704100 -2.60015100 -0.87171900
C -4.58382200 -1.47316100 -0.80303100
C -3.17944400 -1.64902200 -0.78027500
C -2.66438100 -2.94856300 -0.79394200
C -3.49789300 -4.06758300 -0.86252600
C -4.88037600 -3.88550500 -0.91009700
H -6.49541500 -2.45672600 -0.89106600
H -1.58724600 -3.06927300 -0.73889300
H -3.07241700 -5.06710800 -0.87380600
H -5.54717200 -4.74227400 -0.96128700
C -2.24838600 -0.43374000 -0.74707900
H -2.49338600 0.17704100 0.13603600
O -0.89630100 -0.81691500 -0.63937200
H -0.47377100 -1.12817800 -1.88955300
N -0.40641600 -1.24084400 -3.05935800
H -0.52762400 -2.19934500 -3.39036400
H 0.44002600 -0.83944900 -3.46452300
N -5.14631700 -0.18071800 -0.83084200
H -6.14861000 -0.18274100 -0.66746200
H -4.70269900 0.48463600 -0.20377800

79
IN20' SCF Done: E(RM06) = -1770.99030775

C	0.52219600	1.09342600	2.74138600
C	1.81188500	0.48796800	2.48914700
C	1.64971600	-0.97110100	2.59030200
C	-0.45276300	0.03461400	2.72671600
C	0.28107500	-1.24218800	2.69759100
C	3.13120600	1.20335200	2.51322100
H	3.03926400	2.22757200	2.14151000
H	3.52190300	1.25280300	3.53913500
H	3.87897600	0.68973300	1.90170000
C	2.78009400	-1.95058600	2.63834000
H	3.17256700	-2.00692300	3.66389500
H	2.46559700	-2.95538900	2.34733400
H	3.60707300	-1.65388900	1.98947800
C	-0.38040400	-2.58274900	2.77573900
H	-0.74779900	-2.76699100	3.79454300
H	-1.24262800	-2.64203900	2.10397800
H	0.30704800	-3.39170100	2.51634100
C	-1.91084900	0.17045700	3.05993100
H	-2.06857700	0.09301000	4.14484700
H	-2.30912000	1.13914800	2.74189400
H	-2.50835600	-0.61529100	2.58765800
C	0.28696900	2.53383600	3.07731700
H	0.95411200	3.20084300	2.52540700
H	-0.74144600	2.84498500	2.88261000
H	0.47875300	2.68143500	4.14922100
C	3.30489100	-3.10371900	-0.84111000
C	2.68816200	-0.92981300	-1.23795100
C	3.62899500	-0.81849700	-2.30740500
C	4.43984800	-1.93962400	-2.59596400
C	4.27962700	-3.09451500	-1.86173900
H	3.14946100	-4.00676300	-0.25345300
C	3.68331500	0.40068500	-3.03331200
H	5.16914600	-1.87582700	-3.39945100
H	4.87542700	-3.98034900	-2.05497500
C	2.81224600	1.40763700	-2.72365500
C	1.87877200	1.24685300	-1.65904900
H	4.40450200	0.51253900	-3.83867900
H	2.82805400	2.33520300	-3.28286300
N	2.53930600	-2.06898000	-0.52893400
N	1.86368600	0.12143200	-0.91113400
C	0.85606600	2.21885500	-1.31673000
C	0.61402700	3.39733500	-2.04253600
C	-0.45797900	4.22605700	-1.73292900
C	-1.31682100	3.86118300	-0.68145500
C	-1.07445100	2.68478900	0.05334800
C	0.01420700	1.85826200	-0.22444000
H	1.25214100	3.67854600	-2.87504300
H	-0.62438400	5.12870500	-2.30838300
H	-1.76637900	2.45516200	0.85792100
Ir	0.52262200	0.17068100	0.76992700
O	-2.40304900	4.57441200	-0.29893300
C	-2.70357200	5.79093200	-0.98261200
H	-2.91148500	5.61227500	-2.04456000
H	-3.59966300	6.18695900	-0.50156200
H	-1.88833900	6.51756000	-0.88320000
N	-2.37529700	0.09683700	-2.04623000
H	-0.99658700	-1.32175800	-3.38058100
H	-3.35144100	0.10818500	-2.33966700
H	-2.07040200	1.06474700	-1.94911500
C	-5.41294900	-2.73921800	-0.72815300
C	-4.57274400	-1.61480200	-0.74953500
C	-3.16983300	-1.80014500	-0.70743600
C	-2.66228200	-3.10005400	-0.61845200
C	-3.50400400	-4.21397400	-0.59587000
C	-4.88522300	-4.02612700	-0.65858900
H	-6.49004600	-2.59056600	-0.76501600
H	-1.58715800	-3.23373000	-0.56402400
H	-3.08478100	-5.21367500	-0.52878400
H	-5.55775400	-4.87967100	-0.64168600
C	-2.25000600	-0.58377400	-0.76253100
H	-2.47066800	0.07660800	0.08778000
O	-0.87448500	-0.95186500	-0.55820000
H	-0.49584600	-1.32467600	-1.44333400
N	-0.24509600	-1.88473700	-2.97520300
H	-0.46809000	-2.86552400	-3.14180900
H	0.61270300	-1.67751400	-3.48424000
N	-5.12778100	-0.32907000	-0.89062700
H	-6.12779100	-0.30656100	-0.71568800
H	-4.66980600	0.39947900	-0.35102800

73

TS15-1 SCF Done: E(RM06) = -1659.04212823

C	0.01394400	-0.80279100	2.73605200
C	-1.36540500	-0.40560900	2.57226900
C	-1.40446000	1.06333100	2.51597800
C	0.82389700	0.36972400	2.51894300
C	-0.08549700	1.52615800	2.44325200
C	-2.56336500	-1.28087300	2.80189200
H	-2.35486600	-2.32186100	2.53915900
H	-2.86345300	-1.25584800	3.85876900
H	-3.42182500	-0.94995000	2.20914800
C	-2.65654800	1.87828500	2.59373700
H	-2.96668300	1.96998100	3.64470500
H	-2.51679600	2.88657600	2.19850900
H	-3.48151200	1.41199700	2.05073600
C	0.37357100	2.94614000	2.31484200
H	0.81395300	3.29490200	3.25884000
H	1.13941500	3.04973300	1.53958100
H	-0.45170500	3.61616600	2.06083600
C	2.30854900	0.47184200	2.72080600
H	2.53958700	0.70818400	3.76899200
H	2.81795700	-0.46627100	2.48021100
H	2.74768800	1.26380700	2.10648600
C	0.48222700	-2.15828700	3.16881200
H	-0.11031200	-2.95925200	2.71934400
H	1.53135500	-2.33436000	2.92268900
H	0.38204200	-2.23852700	4.25998100
C	-3.57400800	2.68562400	-0.88150600
C	-2.72615500	0.57583100	-1.18162000
C	-3.72145400	0.27524900	-2.16218400
C	-4.67537700	1.27246300	-2.46640500
C	-4.60351300	2.49061300	-1.82731700
H	-3.49245400	3.64004400	-0.36428800
C	-3.68976500	-0.99757000	-2.78897100
H	-5.44464600	1.06381400	-3.20526200
H	-5.31077900	3.28651100	-2.03528600

C	-2.69341600	-1.87858900	-2.47280700
C	-1.71303600	-1.53090500	-1.50038000
H	-4.44772500	-1.25046000	-3.52548000
H	-2.64057500	-2.84520900	-2.95862400
N	-2.67241200	1.77159700	-0.55711300
N	-1.76784300	-0.35130000	-0.84448600
C	-0.56399000	-2.35717500	-1.17027800
C	-0.26266500	-3.57273800	-1.80658100
C	0.91772100	-4.25475700	-1.53414700
C	1.82601600	-3.69841700	-0.61622500
C	1.52346000	-2.48519600	0.03180200
C	0.32486700	-1.80639000	-0.19951600
H	-0.94427000	-4.00071500	-2.53617300
H	1.12530300	-5.19252300	-2.03525000
H	2.25525700	-2.11406100	0.74325600
Ir	-0.29061900	-0.10179500	0.69556100
O	3.01709700	-4.25385000	-0.28646300
C	3.38232100	-5.50398300	-0.87036100
H	3.47268900	-5.42652900	-1.96092600
H	4.35586300	-5.75584200	-0.44567600
H	2.66191900	-6.29035300	-0.61545400
N	1.98060800	0.16008300	-2.45128700
H	0.79855900	0.43223300	-2.06842200
H	2.41211700	0.63255700	-3.24771000
H	2.22756600	-0.83154200	-2.43782300
C	4.99017300	3.36880700	-1.11699300
C	4.27548200	2.17688900	-0.99244600
C	2.89048300	2.15777400	-1.20711500
C	2.22569300	3.34501900	-1.53965400
C	2.94314300	4.53617000	-1.66247500
C	4.32441200	4.55003200	-1.45381800
H	6.06285700	3.37642100	-0.94505100
H	1.15006200	3.33007600	-1.68290800
H	2.42196400	5.45515100	-1.91643100
H	4.87964800	5.47923300	-1.54680900
C	2.13926200	0.85153100	-1.10743800
H	2.61496200	0.16200300	-0.40732800
O	0.73754700	0.98397800	-0.89482000
H	4.79543700	1.25925000	-0.72492800

88

TS15-2 SCF Done: E(RM06) = -1892.67026538

C	-0.82436800	-3.13213900	-0.01685000
C	-0.05361400	-2.97582000	1.19143000
C	1.36310300	-2.87352300	0.81755900
C	0.07113000	-2.88151700	-1.12628000
C	1.43248900	-2.80827700	-0.57734700
C	-0.55098000	-3.23870000	2.58378800
H	-1.59884800	-2.94820900	2.70112100
H	-0.47292000	-4.30888200	2.82069600
H	0.03489100	-2.69678800	3.33162800
C	2.51433600	-2.93136300	1.77332400
H	2.78557700	-3.97888900	1.96942900
H	3.40019100	-2.42992900	1.37499900
H	2.27131400	-2.47146600	2.73345200
C	2.67840200	-2.74624800	-1.40497500
H	2.99053700	-3.76545200	-1.67480200
H	2.52543400	-2.19965300	-2.33817000
H	3.50696800	-2.27455800	-0.87138000
C	-0.25142500	-3.12334100	-2.57393300
H	-0.08949000	-4.17863900	-2.83627700
H	-1.29551800	-2.88933200	-2.80094100
H	0.37905600	-2.52686900	-3.24105100
C	-2.24281000	-3.60938100	-0.09645800
H	-2.83241300	-3.27680800	0.76192300
H	-2.74660300	-3.26589400	-1.00241900
H	-2.25343700	-4.70818100	-0.10441200
C	2.95614500	0.53123100	3.11442900
C	0.71319200	0.62044800	2.63689400
C	0.45696600	1.39145400	3.81276200
C	1.55030800	1.70775000	4.65053900
C	2.81266400	1.28021300	4.30203100
H	3.94255000	0.18237300	2.81354300
C	-0.87598900	1.80556300	4.07068400
H	1.37733500	2.28798600	5.55316500
H	3.68059000	1.50532300	4.91286700
C	-1.86183000	1.49706200	3.17468300
C	-1.55709700	0.74153100	2.00559700
H	-1.09546300	2.37495900	4.97001300
H	-2.88091200	1.82119000	3.34732800
N	1.95561800	0.20224200	2.31118800
N	-0.30715800	0.28097800	1.77958900
C	-2.51467000	0.44567600	0.95370900
C	-3.82741300	0.94768600	0.92139600
C	-4.64950700	0.73545500	-0.17884900
C	-4.14157000	0.02080400	-1.27831500
C	-2.83361000	-0.49661500	-1.24472200
C	-2.00858400	-0.32404100	-0.13458900
H	-4.22283500	1.52486300	1.75245700
H	-5.65783800	1.13135700	-0.18292200
H	-2.50072400	-1.05310700	-2.11603500
Ir	-0.15468700	-1.08074100	0.11785100
O	-4.83785700	-0.22549900	-2.41497500
C	-6.18124900	0.24384100	-2.51573700
H	-6.23035000	1.33782100	-2.45234500
H	-6.53349000	-0.07532200	-3.49837700
H	-6.82040600	-0.19882900	-1.74207700
N	0.46392400	1.72633800	-3.05964600
H	0.53732900	2.80595300	-2.05480200
H	0.98301200	1.87060500	-3.92500700
H	-0.50886300	1.52529600	-3.29799800
C	4.59001000	0.50530300	-3.66832900
C	3.20109400	0.42358700	-3.56314100
C	2.56073400	0.66909900	-2.33904200
C	3.33335600	0.98434900	-1.21791300
C	4.72468100	1.06657200	-1.32318300
C	5.35598800	0.83067400	-2.54570500
H	5.07348900	0.31031500	-4.62185400
H	2.84005400	1.14405900	-0.26550900
H	5.31679300	1.31450700	-0.44587100
H	6.43793200	0.89353700	-2.62416400
C	1.03659300	0.58622200	-2.25106200
H	0.69691600	-0.35496400	-2.69853900
O	0.52701500	0.69868000	-0.95452200
H	2.61551500	0.15386200	-4.44125300
C	-1.75644800	3.83431700	-1.09415500

H	-2.09721400	2.90941300	-0.61877400
H	-1.80837800	3.70794900	-2.18160700
H	-2.45466900	4.63312000	-0.82082700
C	-0.34165400	4.20563600	-0.64617300
C	0.17986900	5.43844200	-1.38575900
H	0.16251500	5.28619800	-2.47123600
H	-0.44703700	6.30714600	-1.15835100
H	1.20803200	5.66520900	-1.08592200
C	-0.24820200	4.38541700	0.86584700
H	-0.63058100	3.50726100	1.39526800
H	0.78909600	4.55696000	1.17131800
H	-0.84498500	5.24993300	1.17602800
O	0.59642100	3.11846600	-1.00334800
H	0.44716800	2.06738000	-0.67490500

77

IN21 SCF Done: E(RM06) = -1715.66456230

C	2.04550100	-0.59560500	2.24878600
C	1.64017300	-1.90413100	1.80756200
C	0.28300900	-2.13729200	2.30823000
C	0.88514100	0.05421900	2.81264500
C	-0.17549700	-0.95784900	2.91355100
C	2.54040800	-2.97089200	1.25336400
H	3.38089800	-2.54457200	0.69826000
H	2.95580000	-3.58471800	2.06452000
H	1.99990600	-3.64273600	0.57942500
C	-0.43409300	-3.45185600	2.28205800
H	-0.11467200	-4.05965500	3.14101600
H	-1.51732400	-3.32674300	2.35119400
H	-0.21220500	-4.02755100	1.38013100
C	-1.49436800	-0.78403400	3.61275600
H	-1.54784800	-1.44990200	4.48394200
H	-1.62113400	0.23698900	3.98177800
H	-2.34621200	-1.02057300	2.96638400
C	0.87457200	1.38705300	3.50508900
H	1.10883600	1.27548800	4.57275100
H	1.61626300	2.06791500	3.07661100
H	-0.10391300	1.87340900	3.43699300
C	3.44351300	-0.05832800	2.22001000
H	4.00153900	-0.40568500	1.34684500
H	3.46990000	1.03339900	2.22657000
H	3.97488700	-0.40638400	3.11659400
C	-2.67763300	-3.39918100	-0.74026100
C	-0.79739400	-2.28407700	-1.46688500
C	-0.79279100	-3.02170900	-2.68967000
C	-1.78386700	-4.01151900	-2.87190500
C	-2.73133700	-4.21112700	-1.89039700
H	-3.43421800	-3.50326700	0.03454400
C	0.19286800	-2.70460100	-3.66191000
H	-1.78988300	-4.59374600	-3.78956300
H	-3.51228900	-4.95674200	-1.99401200
C	1.07432500	-1.68911300	-3.41804300
C	1.03957600	-0.99045900	-2.17686300
H	0.22218700	-3.26098100	-4.59483100
H	1.81658500	-1.41511600	-4.15770200
N	-1.74759900	-2.47770900	-0.52421800
N	0.15662300	-1.33029600	-1.21005200
C	1.88502900	0.14287000	-1.85760000
C	2.75736800	0.75651500	-2.77315100
C	3.43417900	1.92437900	-2.44452600
C	3.22348900	2.49983000	-1.17840100
C	2.36078400	1.88069500	-0.25356200
C	1.70155800	0.69154400	-0.55525400
H	2.90552300	0.33757300	-3.76464100
H	4.09979600	2.38269900	-3.16593200
H	2.24937700	2.36243700	0.71372600
Ir	0.50283000	-0.37450300	0.68895900
O	3.80862800	3.64347700	-0.75313800
C	4.71608900	4.32166900	-1.62153600
H	4.21833600	4.65219000	-2.54127700
H	5.06040300	5.19510700	-1.06494800
H	5.57682700	3.69049200	-1.87359700
N	-0.99510900	1.09572500	0.07951500
H	-1.15672700	1.08521000	-0.95278900
H	-0.56439700	2.00483700	0.26161000
H	-2.34219900	-0.86331900	0.40426100
C	-4.94635800	3.23362900	-0.98867500
C	-4.19449400	2.12245400	-0.60042600
C	-3.18654800	2.25455600	0.36243000
C	-2.95165900	3.50999000	0.93729200
C	-3.69967700	4.62186200	0.54656800
C	-4.69867200	4.48573600	-0.42000800
H	-5.73421700	3.11965000	-1.72889200
H	-2.18834200	3.61951000	1.70607000
H	-3.51076400	5.58916900	1.00412000
H	-5.28796400	5.34793500	-0.72006200
C	-2.32517700	1.07025600	0.77700500
H	-2.09286100	1.16393500	1.84632600
O	-2.98387800	-0.12199600	0.49343200
H	-4.39452300	1.14409400	-1.02388800
N	-1.53375500	1.53892400	-2.82347300
H	-2.35263200	2.14500400	-2.76992400
H	-1.76533500	0.80656600	-3.49421200
H	-0.79252500	2.09355600	-3.25159800

79

IN21' SCF Done: E(RM06) = -1771.01101043

C	1.76026100	0.06235900	2.55975700
C	1.73098600	-1.33242600	2.20928200
C	0.38181300	-1.83636300	2.49676400
C	0.40095700	0.46919500	2.83519300
C	-0.41720300	-0.75019300	2.87859600
C	2.93180800	-2.19684500	1.95013100
H	3.72791200	-1.64112900	1.44672400
H	3.34085200	-2.58043300	2.89517600
H	2.68323600	-3.06277700	1.32923400
C	-0.01175600	-3.28146700	2.50374200
H	0.26360000	-3.73270400	3.46780200
H	-1.08892700	-3.41404300	2.37514000
H	0.49691000	-3.85221600	1.72356600
C	-1.83757900	-0.81287100	3.35423900
H	-1.85474300	-1.00628900	4.43621700
H	-2.37128700	0.12787000	3.19465300
H	-2.40597200	-1.61323200	2.87152000
C	-0.02392900	1.80796800	3.36965100
H	0.04919000	1.83136800	4.46580000

H	0.60614500	2.61466500	2.98311500
H	-1.06158300	2.04225600	3.11039800
C	2.99252200	0.90029000	2.71496700
H	3.77826600	0.61325000	2.01162800
H	2.79036100	1.96454100	2.57922700
H	3.38792800	0.76518700	3.73121300
C	-1.29460600	-4.08457700	-0.93500100
C	0.31813500	-2.50486100	-1.40301500
C	0.81573500	-3.29146000	-2.48618100
C	0.21477300	-4.54619800	-2.72893900
C	-0.84108200	-4.95693200	-1.94266600
H	-2.15573700	-4.35707400	-0.32846200
C	1.87304400	-2.76046700	-3.27206100
H	0.58829000	-5.16650600	-3.53919400
H	-1.33195000	-5.91150500	-2.09835900
C	2.35919100	-1.51579400	-2.98795200
C	1.85047900	-0.78063600	-1.87790100
H	2.27121300	-3.34313500	-4.09806600
H	3.15041000	-1.08437200	-3.58869000
N	-0.74149200	-2.90835400	-0.66356400
N	0.88733100	-1.29718700	-1.08007200
C	2.28150000	0.55724800	-1.52640200
C	3.14378400	1.33853300	-2.31536700
C	3.41353500	2.66030600	-1.98695500
C	2.79743300	3.21989000	-0.85296100
C	1.94524200	2.43670600	-0.05097400
C	1.69136600	1.09998000	-0.34873800
H	3.60382600	0.92809500	-3.20979400
H	4.07712800	3.24547800	-2.61180400
H	1.50891800	2.91797300	0.81986900
Ir	0.55500700	-0.17924000	0.74031000
O	2.96751200	4.49941700	-0.44755300
C	3.81927100	5.35778000	-1.20671300
H	3.44978800	5.48219600	-2.23181300
H	3.79778700	6.32199100	-0.69588800
H	4.84930500	4.98196300	-1.22811700
N	-1.08511100	0.84743200	-0.29007800
H	-0.94997500	0.84696300	-1.32360400
H	-0.99099100	1.81800100	0.01137200
H	-2.01036400	-1.48363500	-0.42620800
C	-5.91785400	1.97687600	-0.34839700
C	-4.89637400	1.05043600	-0.08058600
C	-3.56083500	1.37677700	-0.42924600
C	-3.30329200	2.60136400	-1.05326500
C	-4.32583300	3.51353600	-1.32374100
C	-5.63492600	3.19595000	-0.95858700
H	-6.94050400	1.72685100	-0.07415400
H	-2.28762700	2.86022600	-1.33860000
H	-4.09902600	4.45908400	-1.80712600
H	-6.44324700	3.89514100	-1.15586400
C	-2.47749100	0.38753600	-0.02921000
H	-2.55647200	0.22359500	1.05103100
O	-2.69018200	-0.82986200	-0.71653400
N	-1.37657700	0.70073400	-3.24932400
H	-2.09822100	0.00032900	-3.07594500
H	-0.76962100	0.32502900	-3.97725000
H	-1.85407100	1.50331800	-3.65876200
N	-5.17095300	-0.15095700	0.57661400
H	-6.15729100	-0.38328400	0.61594600
H	-4.61447000	-0.92952200	0.23704600

77

TS16 SCF Done: E(RM06) = -1715.61588804

C	1.90677700	-0.10707100	2.43548000
C	1.27148400	-1.39672700	2.37997200
C	-0.13364500	-1.25056400	2.64899700
C	0.87310900	0.86659500	2.73479900
C	-0.37569500	0.15792200	2.84612500
C	1.99081200	-2.69858100	2.18777500
H	2.87903200	-2.58386000	1.55983200
H	2.32730900	-3.08127900	3.16117000
H	1.34735600	-3.46013600	1.74088200
C	-1.12404500	-2.35889500	2.85212100
H	-1.15058400	-2.67696300	3.90426500
H	-2.13974800	-2.04913100	2.58453100
H	-0.87528400	-3.23665900	2.24962300
C	-1.67013500	0.78139100	3.27733400
H	-1.69431100	0.86642000	4.37246100
H	-1.79415300	1.78720900	2.86622600
H	-2.53583800	0.18365500	2.98107000
C	1.10085800	2.29834500	3.11797700
H	1.30286700	2.36607600	4.19630400
H	1.95806100	2.73019700	2.59585800
H	0.22564900	2.92164400	2.91138300
C	3.38180300	0.16691600	2.37689000
H	3.90317700	-0.56180600	1.74864600
H	3.58806500	1.16163500	1.97043600
H	3.83030900	0.11899300	3.37915500
C	-0.54160400	-4.32359700	-0.71948100
C	0.77413700	-2.48594900	-1.20587500
C	1.55971900	-3.25959200	-2.11584200
C	1.27729300	-4.63790400	-2.23555600
C	0.23356300	-5.18503700	-1.51805400
H	-1.41792400	-4.70401500	-0.19878300
C	2.56149400	-2.59297800	-2.87132800
H	1.88327800	-5.24674000	-2.90148100
H	-0.01433400	-6.23889500	-1.58351700
C	2.72034300	-1.24355300	-2.72658500
C	1.95246900	-0.52646000	-1.76238300
H	3.17368700	-3.16015000	-3.56715300
H	3.45984900	-0.71253200	-3.31410800
N	-0.28835300	-3.02853400	-0.55824200
N	1.05061000	-1.16152500	-0.97357500
C	2.06133000	0.89988900	-1.53690900
C	2.78223600	1.77127300	-2.37302600
C	2.75664500	3.14414000	-2.16735400
C	1.98333000	3.65684100	-1.10895200
C	1.27126800	2.78664400	-0.26280500
C	1.31028700	1.40354300	-0.43285300
H	3.35900200	1.38810900	-3.21027000
H	3.31514600	3.79872200	-2.82575200
H	0.70204800	3.24170100	0.54177300
Ir	0.43084300	-0.00055200	0.74521900
O	1.86598900	4.97542300	-0.82140100
C	2.57788000	5.92360100	-1.61385700
H	2.25306200	5.89646300	-2.66131100

H	2.34082900	6.90113300	-1.18970700
H	3.66092200	5.75896400	-1.55974400
N	-1.31425500	0.51423800	-0.30601000
H	-1.43099500	0.01573700	-3.16870100
H	-1.41943200	1.51542900	-0.42096100
H	-1.78756900	-1.95955500	-0.62861900
C	-6.17996400	0.93904900	0.00025500
C	-4.98316600	0.27857800	0.27944500
C	-3.79631200	0.63294000	-0.37625300
C	-3.83593300	1.66290100	-1.32741900
C	-5.03218300	2.32611600	-1.60842000
C	-6.20766600	1.96578000	-0.94607900
H	-7.08896100	0.65367500	0.52275400
H	-2.92872600	1.95912200	-1.84745500
H	-5.04449400	3.12774900	-2.34222200
H	-7.13775600	2.48358700	-1.16369700
C	-2.52402000	-0.12416500	-0.04653800
H	-2.62095400	-0.58509900	0.94239600
O	-2.59692600	-1.44341900	-0.90603900
H	-4.96791600	-0.52386300	1.01324100
N	-2.18218700	-0.67160100	-3.26067800
H	-2.40896900	-1.11450600	-2.05437100
H	-1.89015300	-1.39073800	-3.92465400
H	-3.00446500	-0.20022700	-3.64190200

77

IN22 SCF Done: E(RM06) = -1715.65702672

C	1.71431500	-1.46886400	2.18272000
C	0.69446400	-2.38803600	1.76932100
C	-0.59366000	-1.89593900	2.27432800
C	1.05080500	-0.31179000	2.74849900
C	-0.37590000	-0.63677100	2.85782100
C	0.92970800	-3.76224900	1.21164300
H	1.84916300	-3.80573000	0.62105600
H	1.02318900	-4.49335500	2.02667700
H	0.10360600	-4.09100300	0.57486100
C	-1.87669300	-2.67001800	2.26931600
H	-1.93263700	-3.29490900	3.17261200
H	-2.74936800	-2.01255700	2.26064800
H	-1.94326700	-3.33819200	1.40731500
C	-1.39400500	0.22466400	3.54229700
H	-1.46502100	-0.06194100	4.60141400
H	-1.10753600	1.28031300	3.51745100
H	-2.38583900	0.12621500	3.09360100
C	1.71818300	0.83226000	3.45765600
H	1.77333900	0.63671100	4.53746700
H	2.74085300	0.98651300	3.10266200
H	1.17146200	1.77080000	3.32216700
C	3.19183200	-1.71120100	2.12281700
H	3.47005000	-2.32618300	1.26267400
H	3.76203200	-0.78139000	2.07039000
H	3.50754200	-2.24327000	3.03073300
C	-3.49116200	-2.23829700	-1.11529500
C	-1.32850300	-1.70636700	-1.70776100
C	-1.51194700	-2.31028800	-2.99221100
C	-2.75359100	-2.91715400	-3.27985500
C	-3.75314600	-2.89543400	-2.33229100
H	-4.26888200	-2.16842300	-0.35741400
C	-0.44069500	-2.26767000	-3.92129900
H	-2.89979200	-3.39017000	-4.24737300
H	-4.72009000	-3.35436500	-2.50906200
C	0.72209700	-1.64527800	-3.57116000
C	0.87339000	-1.08488400	-2.27047000
H	-0.56203000	-2.72134500	-4.90105400
H	1.54584500	-1.58692000	-4.27150100
N	-2.33514100	-1.66435900	-0.80362500
N	-0.11952500	-1.15318600	-1.35343400
C	2.07287500	-0.38742800	-1.84379900
C	3.15586500	-0.09770000	-2.69305500
C	4.22500200	0.67417800	-2.25629500
C	4.20379100	1.18739500	-0.94761100
C	3.12820700	0.88997100	-0.08944200
C	2.07169500	0.07906600	-0.49923500
H	3.17330100	-0.46078100	-3.71646200
H	5.04826800	0.88446800	-2.92838600
H	3.16483900	1.30589700	0.91240000
Ir	0.48843600	-0.47051500	0.63950900
O	5.17146800	1.97251500	-0.41937400
C	6.30643000	2.30133400	-1.21968700
H	6.01578000	2.86044100	-2.11746600
H	6.93836200	2.93173000	-0.59152500
H	6.86493200	1.40284000	-1.50862300
N	-0.21885000	1.42255700	0.11290800
H	-6.69960400	1.20087600	0.34798000
H	0.48769000	2.00640400	-0.33661600
H	-3.16379700	-0.45494500	0.61930000
C	-3.47348900	5.04483600	-0.20181000
C	-3.05027200	3.75942900	0.12946600
C	-1.71636300	3.36686600	-0.08729000
C	-0.81236900	4.29293800	-0.64326600
C	-1.23847700	5.57554400	-0.97095200
C	-2.56821300	5.95461900	-0.75129300
H	-4.50508500	5.33775700	-0.02915700
H	0.22626200	4.02302200	-0.81849900
H	-0.53461800	6.28518800	-1.39592800
H	-2.89376700	6.95895400	-1.00737100
C	-1.36174700	1.99544300	0.28748300
H	-2.15367800	1.40571600	0.75096900
O	-3.75080000	0.03570000	1.22516300
H	-3.74627100	3.04232900	0.55670800
N	-6.23310700	0.41965200	-0.11340700
H	-4.60396600	0.15847200	0.73101600
H	-6.20771600	0.65029200	-1.10649600
H	-6.86263900	-0.37718800	-0.01849000

79

TS16' SCF Done: E(RM06) = -1770.96506678

C	1.63293400	0.38930800	2.59753400
C	1.62512900	-1.03363500	2.44461000
C	0.25032900	-1.50044000	2.65649900
C	0.25365100	0.82959700	2.72395300
C	-0.57023200	-0.37420500	2.84379400
C	2.83536700	-1.92080800	2.37530500
H	3.67763600	-1.41672200	1.89261700
H	3.15885800	-2.21250100	3.38451300
H	2.63481800	-2.84297900	1.82160700

C	-0.16708600	-2.93515100	2.78176400
H	-0.08054600	-3.26257900	3.82777500
H	-1.20534900	-3.08618700	2.47435600
H	0.46123600	-3.59999200	2.18399700
C	-2.02641800	-0.37045000	3.19643400
H	-2.13923900	-0.30315000	4.28793100
H	-2.55047300	0.48926500	2.76951700
H	-2.53939700	-1.28054200	2.87256900
C	-0.20774200	2.21520900	3.07612400
H	-0.28449500	2.33940000	4.16556800
H	0.48742800	2.97621900	2.71023800
H	-1.19208000	2.42960700	2.64835100
C	2.84889700	1.25966100	2.70560600
H	3.69891000	0.84467800	2.15674100
H	2.66771200	2.26818400	2.32681900
H	3.14323900	1.34861400	3.76041900
C	-0.90690000	-4.26096300	-0.73677000
C	0.64892200	-2.61318600	-1.17013900
C	1.36903400	-3.48582600	-2.04440100
C	0.91445700	-4.81623600	-2.18112500
C	-0.22096300	-5.21849100	-1.50958200
H	-1.84052900	-4.52779200	-0.24538200
C	2.48377800	-2.95798300	-2.74871800
H	1.46293400	-5.50167700	-2.82209800
H	-0.60182800	-6.23118300	-1.58693400
C	2.80928800	-1.63936300	-2.59396500
C	2.08927400	-0.82458300	-1.67143700
H	3.05073400	-3.60240300	-3.41505800
H	3.63883500	-1.21110800	-3.14378100
N	-0.49841800	-3.00908400	-0.56429500
N	1.07961400	-1.33295700	-0.92701200
C	2.35209500	0.58597400	-1.45708300
C	3.21307700	1.35309700	-2.26211000
C	3.33073800	2.72514100	-2.08073400
C	2.56122900	3.34611500	-1.07915600
C	1.70960000	2.58092900	-0.26148200
C	1.60534000	1.19668000	-0.40493600
H	3.79128100	0.88916800	-3.05669100
H	3.99754200	3.29781000	-2.71421800
H	1.14916500	3.11021600	0.50370100
Ir	0.51213500	-0.04997600	0.74756400
O	2.57674600	4.67696600	-0.82499500
C	3.43178300	5.51623100	-1.59858000
H	3.16295500	5.49231700	-2.66204300
H	3.28369100	6.52661700	-1.21308500
H	4.48515100	5.23472400	-1.47951600
N	-1.10522300	0.62122600	-0.39544500
H	-0.98713300	0.60840500	-2.96291800
H	-1.02467100	1.55825700	-0.76694400
H	-1.93786400	-1.72964500	-0.95447000
C	-5.97116200	1.45281400	-0.59269000
C	-4.88759100	0.57477200	-0.40612700
C	-3.56225700	1.07121200	-0.54736600
C	-3.38830900	2.42331300	-0.86805100
C	-4.46860900	3.28566800	-1.06047300
C	-5.76589700	2.79024700	-0.91503700
H	-6.98249000	1.06791600	-0.47986100
H	-2.38600500	2.83647600	-0.94286800
H	-4.29692900	4.33050100	-1.30162600
H	-6.62201800	3.44560400	-1.05273600
C	-2.39598100	0.13971800	-0.26867400
H	-2.56752600	-0.38856000	0.67212700
O	-2.62323700	-1.09182200	-1.26589400
N	-1.76024100	0.08254400	-3.38663800
H	-2.17857000	-0.52564300	-2.54074200
H	-1.41236300	-0.49204000	-4.15818400
H	-2.46177200	0.73898900	-3.74014500
N	-5.11107100	-0.74805800	-0.03125600
H	-6.06984000	-1.05682200	-0.14507300
H	-4.44078500	-1.39637500	-0.43518600

79

IN22' SCF Done: E(RM06) = -1771.01136503

C	-1.67545300	-1.51502500	-2.21160700
C	-0.72762400	-2.45188100	-1.68503900
C	0.61223600	-2.02673100	-2.10011100
C	-0.92719000	-0.41033300	-2.77958100
C	0.48568800	-0.79309800	-2.76444900
C	-1.06074600	-3.78363700	-1.07672100
H	-1.99785000	-3.74760900	-0.51435800
H	-1.17295000	-4.54440700	-1.86165900
H	-0.27532500	-4.13067500	-0.39914900
C	1.87022300	-2.82594400	-1.94436500
H	2.00882500	-3.48286600	-2.81526000
H	2.74743400	-2.17878400	-1.86841400
H	1.83976100	-3.46308500	-1.05707600
C	1.58984700	-0.02074100	-3.42171800
H	1.77416000	-0.42810500	-4.42619500
H	1.32522500	1.03266300	-3.55062700
H	2.52308200	-0.07751700	-2.85439600
C	-1.49430700	0.72478200	-3.58402000
H	-1.49191100	0.48032600	-4.65531700
H	-2.52771600	0.94419900	-3.30235300
H	-0.91331400	1.64330700	-3.45456000
C	-3.16193000	-1.70266600	-2.25600400
H	-3.53084400	-2.26639700	-1.39459100
H	-3.69899100	-0.75245900	-2.28797200
H	-3.42759100	-2.26450100	-3.16205600
C	3.25370400	-2.18860400	1.61183600
C	1.03143000	-1.67250300	1.93176600
C	1.07086600	-2.24515900	3.24313900
C	2.27621900	-2.82656100	3.69068600
C	3.38020700	-2.81043900	2.86765200
H	4.11904100	-2.13070300	0.95447800
C	-0.09973500	-2.19399800	4.04314500
H	2.31322100	-3.27168500	4.68139500
H	4.32693500	-3.24512200	3.16990700
C	-1.21553300	-1.57861500	3.55601900
C	-1.21705400	-1.03503200	2.23868600
H	-0.08721900	-2.63111700	5.03778400
H	-2.11105100	-1.50896200	4.16060800
N	2.13618500	-1.64162500	1.14767800
N	-0.13268900	-1.13369900	1.43394800
C	-2.34609800	-0.31396500	1.67983500
C	-3.50793700	0.00765300	2.40574100
C	-4.50373700	0.80015700	1.85170800

C	-4.32934200	1.30160600	0.54924200
C	-3.17266700	0.97791100	-0.18354700
C	-2.18552400	0.14757500	0.34282100
H	-3.64564100	-0.34680400	3.42277500
H	-5.38839100	1.03639200	2.43044000
H	-3.08793700	1.39177100	-1.18301300
Ir	-0.52246600	-0.49435000	-0.62457800
O	-5.22053000	2.09778300	-0.08593800
C	-6.42718000	2.45749500	0.58599100
H	-6.22215900	3.03179200	1.49752700
H	-6.98083700	3.08330800	-0.11623200
H	-7.02692800	1.57340900	0.83337100
N	0.28015900	1.32181900	0.01937700
H	6.81323600	-1.15105100	-0.03956500
H	-0.23426600	1.74205400	0.79398700
H	3.07071200	-0.43066400	-0.24672600
C	3.44591100	4.99898600	0.46953900
C	3.08134500	3.71660100	-0.00531000
C	1.73005100	3.29097400	0.18339600
C	0.82424900	4.14286600	0.85604600
C	1.20641000	5.38787600	1.32255000
C	2.53114000	5.81183500	1.11645900
H	4.46976900	5.33642100	0.32669700
H	-0.21112100	3.82985400	0.97024100
H	0.49041600	6.03480600	1.81919100
H	2.84703100	6.79008700	1.46945800
C	1.31040400	2.01003800	-0.35686700
H	1.91338900	1.57859800	-1.15014400
O	3.71416900	0.04137100	-0.81191400
N	6.20837700	-1.26256700	-0.85322800
H	4.56904500	-0.46775300	-0.76641800
H	6.23132300	-2.25283500	-1.09631200
H	6.67292400	-0.77546200	-1.62043200
N	4.00691800	2.93154100	-0.64288000
H	4.96225700	3.26364900	-0.62157300
H	3.91512400	1.91084700	-0.64391100

73

TS16-1 SCF Done: E(RM06) = -1659.02031275

C	0.45732600	-0.18220500	-2.62010800
C	-0.97556400	-0.28675700	-2.62695100
C	-1.33464400	-1.62954500	-2.16575000
C	0.97900800	-1.36512200	-1.97185800
C	-0.15300900	-2.27150900	-1.75644400
C	-1.92223100	0.67918000	-3.27796100
H	-1.54321300	1.70397000	-3.23715300
H	-2.06507200	0.41842100	-4.33578500
H	-2.90751600	0.66442100	-2.80290900
C	-2.70932800	-2.22314400	-2.21938500
H	-2.89934500	-2.63763800	-3.21979200
H	-2.83059600	-3.03519200	-1.49796200
H	-3.48559600	-1.48012000	-2.02192300
C	-0.03704900	-3.66612000	-1.21880700
H	0.15419100	-4.37172300	-2.03912800
H	0.79547200	-3.75943700	-0.51566400
H	-0.95341700	-3.98928000	-0.71585400
C	2.42555200	-1.76344000	-1.91409400
H	2.71337100	-2.29919100	-2.83007800
H	3.08010100	-0.89155700	-1.82599500
H	2.63514200	-2.42099800	-1.06676900
C	1.25484900	0.89152700	-3.29533000
H	0.76722200	1.86788900	-3.23241500
H	2.25832000	0.98796900	-2.87657100
H	1.36319300	0.63940900	-4.35934000
C	-4.35161700	-1.53626900	1.16431000
C	-3.04550800	0.33320700	0.87157400
C	-4.07024700	1.17494900	1.40263700
C	-5.29086700	0.57490900	1.78220100
C	-5.44020600	-0.79035900	1.65966200
H	-4.43111500	-2.61891400	1.08589400
C	-3.79355400	2.56055200	1.54866100
H	-6.08870200	1.19779900	2.17802500
H	-6.35837600	-1.29177900	1.94661100
C	-2.55310200	3.03493900	1.22908700
C	-1.56017600	2.15583200	0.70486700
H	-4.56258000	3.22200200	1.93841200
H	-2.31335800	4.08185000	1.36988800
N	-3.19820600	-1.00852900	0.77602600
N	-1.84098500	0.85596100	0.46677000
C	-0.19191600	2.54585900	0.42808200
C	0.31507500	3.83837000	0.65444700
C	1.65437500	4.13337900	0.43828800
C	2.51033100	3.10756900	-0.00208500
C	2.00814000	1.81500300	-0.23073100
C	0.65961800	1.50321600	-0.04807900
H	-0.33369400	4.63425000	1.00881600
H	2.02186700	5.13694300	0.61499500
H	2.71360400	1.06776100	-0.57813600
Ir	-0.25866600	-0.23479700	-0.53551200
O	3.83600000	3.26754900	-0.23851300
C	4.41125600	4.56533400	-0.09623300
H	4.33468600	4.92912200	0.93562400
H	5.46454300	4.45256300	-0.36001600
H	3.94372900	5.28698700	-0.77698300
N	-0.13844600	-1.04670600	1.49336600
H	1.42934300	-0.66581500	3.88774700
H	-1.08091700	-1.43810800	1.55638300
H	0.05024200	-0.43278000	2.78378200
C	4.15814600	-3.43305800	1.65904300
C	2.78938200	-3.29699200	1.89608900
C	2.19143100	-2.02798300	1.88696000
C	2.98606200	-0.89236200	1.66580300
C	4.35520500	-1.03036300	1.44528900
C	4.94254600	-2.29968500	1.43626200
H	4.61073400	-4.42025600	1.66127400
H	2.53405800	0.09343400	1.65682200
H	4.96319100	-0.14614900	1.27611400
H	6.00967900	-2.40338500	1.26129700
C	0.73636800	-1.92847500	2.17699000
H	0.30279500	-2.88312000	2.47403600
O	0.57047600	-1.05376600	3.61447800
H	2.18306000	-4.17794200	2.09169500

88

TS16-2 SCF Done: E(RM06) = -1892.64971376

C	0.41828500	-2.86866900	1.29602600

C	1.79988600	-2.61224500	0.99975800
C	2.00478000	-2.80018700	-0.43914100
C	-0.27474400	-3.02306500	0.03362500
C	0.74707800	-3.04606300	-1.01504700
C	2.89947300	-2.48053500	2.01340100
H	2.53991400	-2.02942700	2.94245900
H	3.30727800	-3.47034800	2.26153100
H	3.72864100	-1.87216100	1.64111300
C	3.33693000	-2.84345000	-1.12357100
H	3.77314100	-3.84907200	-1.03616200
H	3.25648700	-2.61230000	-2.18914100
H	4.04705100	-2.14019600	-0.68229200
C	0.47603400	-3.35302800	-2.45700200
H	0.50467300	-4.44000200	-2.61777500
H	-0.51563500	-3.01081200	-2.76277500
H	1.21989800	-2.90480300	-3.12229900
C	-1.68768800	-3.50740200	-0.12858000
H	-1.72784000	-4.60477300	-0.06880200
H	-2.34145100	-3.11509400	0.65561000
H	-2.11436100	-3.21384900	-1.09112000
C	-0.16490700	-3.07702500	2.66083200
H	0.34835900	-2.48072700	3.42015200
H	-1.22645700	-2.82246400	2.69730600
H	-0.06606300	-4.13470600	2.94177500
C	4.21804100	0.75895100	-1.98412400
C	2.93340500	1.07782000	-0.10525100
C	3.80503500	2.10934300	0.36001900
C	4.94833700	2.40889300	-0.41347700
C	5.16375300	1.72928600	-1.59344700
H	4.34737700	0.22724100	-2.92499400
C	3.45916300	2.79239000	1.55632600
H	5.63458600	3.17916900	-0.07172200
H	6.02586300	1.93336100	-2.21954800
C	2.29130700	2.48094600	2.19416900
C	1.45320800	1.44170600	1.69205600
H	4.11437500	3.57150300	1.93639100
H	1.99422800	3.01780600	3.08720600
N	3.14739900	0.43120900	-1.27432300
N	1.81596600	0.71841900	0.61084600
C	0.15457500	1.10061800	2.23981800
C	-0.45433700	1.79513100	3.30051200
C	-1.74383400	1.48766300	3.71384000
C	-2.44216600	0.46471700	3.04617000
C	-1.83248200	-0.24204100	1.99483700
C	-0.53064700	0.03795500	1.57594300
H	0.07255400	2.59397400	3.81453300
H	-2.19393800	2.03664200	4.53225400
H	-2.41639100	-1.02612900	1.52465000
Ir	0.54359300	-0.97459600	0.19490700
O	-3.70952900	0.08600100	3.34756400
C	-4.38778300	0.74303700	4.41626400
H	-4.51292900	1.81431600	4.21480100
H	-5.37064200	0.27206600	4.47833500
H	-3.86173000	0.60732300	5.36904100
N	-0.12497600	0.43759200	-1.36966300
H	-1.88451100	1.32173300	-3.87021000
H	0.81711400	0.61659100	-1.72736300
H	-0.47801300	2.13414100	-1.14595100
C	-4.15011500	-1.71696900	-3.23666500
C	-2.82316700	-1.28979200	-3.31703000
C	-2.27286600	-0.47058800	-2.31796900
C	-3.07823200	-0.07650200	-1.24111600
C	-4.40340300	-0.50552900	-1.15965600
C	-4.94260200	-1.32492700	-2.15566500
H	-4.56146900	-2.35465300	-4.01405100
H	-2.66040200	0.54847700	-0.46212700
H	-5.01459200	-0.20191800	-0.31402900
H	-5.97480700	-1.65723700	-2.08859300
C	-0.86330200	0.01988200	-2.49372600
H	-0.31933200	-0.60344000	-3.20829000
O	-1.00685000	1.35109700	-3.44464800
H	-2.20740200	-1.59422700	-4.16089300
C	-1.60237100	5.00701700	-2.43900600
H	-2.17923300	4.50984100	-3.22772800
H	-0.63998300	5.31989800	-2.85715000
H	-2.15361100	5.90343100	-2.13704700
C	-1.39799900	4.08745000	-1.23586700
C	-0.58133800	4.77326700	-0.14155900
H	0.38813100	5.10055000	-0.53075600
H	-1.11755400	5.65177200	0.23336900
H	-0.41024200	4.09771000	0.70367400
C	-2.72462700	3.55058900	-0.69831800
H	-3.27816100	3.00787200	-1.47218000
H	-2.56525300	2.88002100	0.15330300
H	-3.35181400	4.38141100	-0.35658800
O	-0.57157800	2.97067000	-1.74123700
H	-0.90161200	2.31081000	-2.70147500

76

TS16-3 SCF Done: E(RM06) = -1735.47695655

C	0.28257400	-0.29486100	-2.73910600
C	-1.14928100	-0.41392000	-2.65430100
C	-1.46604100	-1.73986000	-2.11410000
C	0.85461400	-1.44665300	-2.07692500
C	-0.25272300	-2.35264100	-1.75747300
C	-2.13970800	0.51618000	-3.29288400
H	-1.77250400	1.54609200	-3.30690400
H	-2.32761400	0.21594200	-4.33305300
H	-3.10182100	0.50719000	-2.77259400
C	-2.83139000	-2.35388200	-2.06356700
H	-3.05357300	-2.85201300	-3.01828500
H	-2.91080000	-3.10552500	-1.27379100
H	-3.61188400	-1.60754200	-1.89769500
C	-0.09081100	-3.73499600	-1.19995200
H	-0.00409900	-4.45910600	-2.02227700
H	0.81658000	-3.82692800	-0.59754800
H	-0.94795000	-4.03689400	-0.59033900
C	2.30698000	-1.82606800	-2.08943100
H	2.55158000	-2.38688900	-3.00293100
H	2.95357800	-0.94432300	-2.06396200
H	2.57308300	-2.45140700	-1.23422500
C	1.02984900	0.76250200	-3.49394300
H	0.53772600	1.73682300	-3.43313600
H	2.05282200	0.88073900	-3.13114000
H	1.08424300	0.47941300	-4.55418700
C	-4.31908600	-1.41366200	1.31625700

C -3.01957000 0.42503100 0.84939300
C -4.03180500 1.30798300 1.33536200
C -5.24676800 0.74165600 1.78016400
C -5.39996500 -0.62896300 1.76613700
H -4.39824100 -2.49909500 1.32781900
C -3.74583500 2.69960200 1.37487900
H -6.03785000 1.39490200 2.13901400
H -6.31464500 -1.10437700 2.10400400
C -2.50858700 3.14207300 1.00082100
C -1.53084600 2.22259100 0.51690900
H -4.50461000 3.39188100 1.72959700
H -2.26012700 4.19456800 1.06347200
N -3.17278100 -0.91941100 0.86771200
N -1.82406900 0.91086600 0.37446100
C -0.16512400 2.57616500 0.19186700
C 0.37546600 3.86128200 0.37844900
C 1.72384700 4.10873400 0.16376100
C 2.55119900 3.04526400 -0.24215200
C 2.01088400 1.76461900 -0.45332000
C 0.65680800 1.49728900 -0.25442000
H -0.25158700 4.68421000 0.70968400
H 2.12179000 5.10425900 0.31924900
H 2.69517500 0.98770800 -0.77700100
Ir -0.30262700 -0.24810300 -0.62843800
O 3.88492700 3.15841000 -0.45684800
C 4.51102900 4.42366600 -0.25209800
H 4.40469600 4.76005900 0.78649500
H 5.56883100 4.27038400 -0.47413600
H 4.10696100 5.18570000 -0.92941100
N -0.02911900 -0.90692200 1.47546100
H 1.47245900 -2.33497900 3.93642200
H -0.99526300 -1.21296800 1.61583500
H 0.18390700 0.11561900 2.52528400
C 4.32532400 -3.17797800 1.30845400
C 2.93945500 -3.15580700 1.47440100
C 2.25911900 -1.93828100 1.63865300
C 2.99103400 -0.74130500 1.65977500
C 4.37508300 -0.76615400 1.49351200
C 5.04412800 -1.98097700 1.31516600
H 4.83954200 -4.12537400 1.17465600
H 2.48132900 0.20423500 1.80176300
H 4.93168100 0.16663400 1.50168100
H 6.12267300 -1.99372700 1.18538200
C 0.78354600 -1.97668100 1.87633700
H 0.36637100 -2.97130500 1.71616300
O 0.64247400 -1.97190900 3.57023500
H 2.37970700 -4.08869200 1.47593900
O 0.44331000 0.41892400 3.55520900
H 0.58559700 -0.83681800 3.79720400
H -0.35980500 0.76871900 3.98068000

15
2a-2 SCF Done: E(RM06) = -325.531677644
N -2.97963400 0.29680500 -0.00022900
H -2.67117200 1.27665600 -0.00086400
C 1.74666200 -1.06015300 -0.00009700
C 0.37005800 -1.28365600 -0.00008200
C -0.53244400 -0.20833100 0.00010500
C -0.02364600 1.10139700 0.00012000
C 1.35010900 1.32635100 0.00005000
C 2.23938600 0.24615100 -0.00005100
H 2.43287400 -1.90268100 -0.00015300
H -0.70372800 1.94980800 0.00031300
H 1.73071700 2.34413000 0.00012300
H 3.31132000 0.42455200 -0.00011900
C -1.98411900 -0.50429100 0.00020500
H -2.22314600 -1.57415200 0.00091600
H -0.01546500 -2.30075200 -0.00011900

17
2a-3 SCF Done: E(RM06) = -380.876588693
N 2.83351200 -0.49766900 -0.26729100
H 2.66285000 0.43670500 -0.65318500
C -2.02255900 -1.09673700 -0.02545200
C -0.68724000 -1.46948000 0.08067700
C 0.35362300 -0.52404800 0.08581700
C 0.02111000 0.85710600 0.04464800
C -1.33158500 1.22405500 -0.06275100
C -2.33701300 0.26341200 -0.10939200
H -2.80546300 -1.84891400 -0.04212100
H -1.58579700 2.28137300 -0.10070500
H -3.37321400 0.58091600 -0.19274400
C 1.73601100 -1.04337400 0.10923000
H 1.81303300 -2.07266500 0.48110900
H -0.42240900 -2.52293100 0.14138300
N 1.00777000 1.84769500 0.07822900
H 0.63971600 2.77697700 0.25483400
H 1.78822000 1.65276000 0.69820200

Supplemental Reference.

Chen, X. W., Zhao, H., Xiong, B., Jiang, H. F., Dixneuf, P. H. and Zhang, M. (2017). Selective synthesis of nitrogen bi-heteroarenes by a hydrogen transfer-mediated direct a, β-coupling reaction. Organic & Biomolecular Chemistry *15*, 6093-6097.

Rajendran, S., Raghunathan, R., Hevus, I., Krishnan, R., Ugrinov, A., Sibi, M. P., Webster, D. C. and Sivaguru, J., (2015). Programmed photodegradation of polymeric/oligomeric materials derived from renewable bioresources. Angew. Chem.-Int. Edit. *54*, 1159-1163.

Wang, C., Chen, H.-Y. T., Bacsa, J., Catlow, C. R. A. and Xiao, J. (2013). Synthesis and X-ray structures of cyclometalated iridium complexes including the hydrides. Dalton Transactions *42*, 935-940.

Marenich, A. V., Cramer, C. J. and Truhlar, D. G. (2009). Performance of SM6, SM8, and SMD on the SAMPL1 test set for the prediction of small-molecule solvation free energies. J. Phys. Chem. B. *113*, 4538−4543.

Becke, A. D. (1993). Density-functional thermochemistry. III. The role of exact exchange. J. Chem. Phys. *98*, 5648−5652.

Lee, C., Yang, W. and Parr, R. G. (1988). Development of the Colle-Salvetti correlation-energy formula into a functional of the electron density. Phys. Rev. B. *37*, 785−789.

Stephens, P. J., Devlin, F. J., Chabalowski, C. F. and Frisch, M. J. (1994). Ab Initio Calculation of Vibrational Absorption and Circular Dichroism Spectra Using Density Functional Force Fields. J. Phys. Chem. *98*, 11623−11627.

Fukui, K. (1970). Formulation of the reaction coordinate. J. Phys. Chem. *74*, 4161−4163.

Fukui, K. (1981). The path of chemical reactions - the IRC approach. Acc. Chem. Res. *14*, 363−368.

Hay, P. J. and Wadt, W. R. (1985). Ab initio effective core potentials for molecular calculations. Potentials for K to Au including the outermost core orbitals. J. Chem. Phys. *82*, 299−310.

Wadt, W. R. and Hay, P. J. (1985). Ab initio effective core potentials for molecular calculations. Potentials for main group elements Na to Bi. J. Chem. Phys. *82*, 284−298.

Ehlers, A. W., Böhme, M., Dapprich, S., Gobbi, A., Höllwarth, A., Jonas, V., Köhler, K. F., Stegmann, R., Veldkamp, A. and Frenking, G. (1993). A set of f-polarization functions for pseudo-potential basis sets of the transition metals Sc Cu, Y Ag and La Au. Chem. Phys. Lett. *208*, 111−114.

Krishnan, R., Binkley, J. S., Seeger R. and Pople, J. A. (1980). Self-consistent molecular orbital methods. XX. A basis set for correlated wave functions. J. Chem. Phys. *72*, 650−654.

McLean, A. D. and Chandler, G. S. (1980). Contracted Gaussian basis sets for molecular calculations. I. Second row atoms, Z=11–18. J. Chem. Phys. *72*, 5639−5648.

Zhao, Y., Schultz, N. E. and Truhlar, D. G. (2005). Exchange-correlation functional with broad accuracy for metallic and nonmetallic compounds, kinetics, and noncovalent interactions. J. Chem. Phys. *123*, 161103.

Zhao, Y. and Truhlar, D. G. (2008). Acc. Chem. Res. *41*, 157−167.

Zhao, Y. and Truhlar, D. G. (2008). The M06 suite of density functionals for main group thermochemistry, thermochemical kinetics, noncovalent interactions, excited states, and transition elements: two new functionals and systematic testing of four M06-class functionals and 12 other functionals. Theor. Chem. Acc. *120*, 215−241.

Zhao, Y. and Truhlar, D. G. (2009). Benchmark Energetic Data in a Model System for Grubbs II Metathesis Catalysis and Their Use for the Development, Assessment, and Validation of Electronic Structure Methods. J. Chem. Theory Comput. *5*, 324−333.

Roy, L. E., Hay, P. J. and Martin, R. L. (2008). Revised Basis Sets for the LANL Effective Core Potentials. J. Chem. Theor. Comput. *4*, 1029−1031.

Grimme, S., Antony, J., Ehrlich, S. and Krieg, H. (2010). A consistent and accurate ab initio parametrization of density functional dispersion correction (DFT-D) for the 94 elements H-Pu. J. Chem. Phys. *132*, 154104.

Chen, M., Zhang, M., Xiong, B., Tan, Z., Lv, W. and Jiang, H. (2014). A Novel Ruthenium-Catalyzed Dehydrogenative

Synthesis of 2-Arylquinazolines from 2-Aminoaryl Methanols and Benzonitriles. Organic Letters *16*, 6028-6031.

Ma, J., Wan, Y., Hong, C., Li, M., Hu, X., Mo, W., Hu, B., Sun, N., Jin, L. and Shen, Z. (2017). ABNO-Catalyzed Aerobic Oxidative Synthesis of 2 - Substituted 4H - 3,1 - Benzoxazines and Quinazolines. European Journal of Organic Chemistry *(23)*, 3335-3342.

Han, B., Yang, X. L., Wang, C., Bai, Y. W., Pan, T. C., Chen, X. and Yu, W. (2012). CuCl/DABCO/4-HO-TEMPO-Catalyzed Aerobic Oxidative Synthesis of 2-Substituted Quinazolines and 4H-3,1-Benzoxazines. J. Org. Chem. *77*, 1136-1142.

Yamaguchi, T., Sakairi, K., Yamaguchi, E., Tada, N. and Itoh, A. (2016). Magnesium iodide-catalyzed synthesis of 2-substituted quinazolines using molecular oxygen and visible light. Rsc Advances 6, 56892-56895.

Zhang, Z., Wang, M., Zhang, C., Zhang, Z., Lu, J. and Wang, F. (2015). The cascade synthesis of quinazolinones and quinazolines using an a-MnO2 catalyst and tert-butyl hydroperoxide (TBHP) as an oxidant. Chemical Communications *51* (44), 9205-9207.

Gopalaiah, K., Saini, A. and Devi, A. (2017). Iron-catalyzed cascade reaction of 2-aminobenzyl alcohols with benzylamines: synthesis of quinazolines by trapping of ammonia. Organic & Biomolecular Chemistry *15*, 5781-5789.

Cheng, X., Wang, H., Xiao, F. and Deng, G.-J. (2016). Lewis acid-catalyzed 2-arylquinazoline formation from N′-arylbenzimidamides and paraformaldehyde. Green Chemistry *18*, 5773-5776.

Malakar, C. C., Baskakova, A., Conrad, J. and Beifuss, U. (2012). Copper-Catalyzed Synthesis of Quinazolines in Water Starting from o-Bromobenzylbromides and Benzamidines. Chemistry-a European Journal *18*, 8882-8885.

Wang, H., Chen, H., Chen, Y. and Deng, G.-J. (2014). Palladium-catalyzed one pot 2-arylquinazoline formation via hydrogen-transfer strategy. Organic & Biomolecular Chemistry *12*, 7792-7799.

Yu, C., Guo, X., Xi, Z., Muzzio, M., Yin, Z., Shen, B., Li, J., Seto, C. T. and Sun, S. (2017). AgPd Nanoparticles Deposited on WO2.72 Nanorods as an Efficient Catalyst for One-Pot Conversion of Nitrophenol/Nitroacetophenone into Benzoxazole/Quinazoline. Journal of the American Chemical Society *139*, 5712-5715.

Gujjarappa, R., Maity, S. K., Hazra, C. K., Vodnala, N., Dhiman, S., Kumar, A., Beifuss, U. and Malakar, C. C. (2018). Divergent Synthesis of Quinazolines Using Organocatalytic Domino Strategies under Aerobic Conditions. European Journal of Organic Chemistry *(33)*, 4628-4638.

Saha, M., Mukherjee, P. and Das, A. R. (2017). A facile and versatile protocol for the one-pot PhI(OAc)2 mediated divergent synthesis of quinazolines from 2-aminobenzylamine. Tetrahedron Lett. *58*, 2044-2049.

Chen, J., Natte, K., Neumann, H. and Wu, X.-F. (2014). A convenient palladium-catalyzed carbonylative synthesis of quinazolines from 2-aminobenzylamine and aryl bromides. Rsc Advances *4*, 56502-56505.
